# Assembly-Line Biosynthesis in Living-Cell Emulsions via Tunable Supramolecular Surface Chemistry

**DOI:** 10.1038/s41467-026-74245-z

**Published:** 2026-06-24

**Authors:** Xiankun Wu, Mathias Dimde, Henrik Karring, Zhongkai Wang, Changzhu Wu

**Affiliations:** 1 Anhui Provincial Engineering Center for High Performance Biobased Nylons, School of Materials and Chemistry, Anhui Agricultural University, Hefei, Anhui China; 2 Department of Physics, Chemistry and Pharmacy, University of Southern Denmark, Odense, Denmark; 3 Department of Biology, Chemistry and Pharmacy, Institute of Chemistry and Biochemistry, Research Center of Electron Microscopy (FZEM), Free University of Berlin, Berlin, Germany; 4 Department of Green Technology, University of Southern Denmark, Odense, Denmark; 5 Danish Institute for Advanced Study (DIAS), University of Southern Denmark, Odense, Denmark

**Keywords:** Biocatalysis, Biosynthesis, Biotechnology

## Abstract

Nature organizes enzymes in micro-compartments to enable efficient biosynthesis, inspiring multienzyme cascades for producing complex molecules. However, practical implementation is often limited by enzyme incompatibility, poor substrate transfer, and inefficient catalyst recycling. Here, we report a living biocatalytic platform that operates as a factory-like assembly line within Pickering emulsions. Tunable supramolecular chemistry is used to graft an oil-derived photocatalyst onto enzyme-overexpressing *Escherichia coli* (*E. coli*) cells, generating amphiphilic “suprabacteria” that self-assemble at water–oil interfaces and stabilize emulsions. These interfacial suprabacteria accelerate chemoenzymatic and multienzyme cascades, achieving reaction rates up to 45-fold higher than conventional biphasic systems. The platform supports single-step, sequential, and one-pot cascade reactions, including gram-scale benzoin synthesis. Importantly, the dynamic supramolecular linkage enables on-demand dual recycling: either the living-cell conjugate is reused, or the synthetic catalyst is selectively recovered and reattached to fresh cells. This strategy integrates chemical and biological catalysis in a sustainable, scalable platform for future greener industrial biosynthesis.

## Abstract (web summary)

Natural enzymes are organised in micro-compartments for efficient biosynthesis. Here, the authors replicate this by modifying *E. coli* with oil-derived photocatalyst so they assemble at the interface of Pickering emulsions, stabilising them and allowing more efficient chemoenzymatic and multienzyme cascades.

## Introduction

The enzymatic cascades found in living organisms represent the pinnacle of chemical manufacturing, converting simple precursors into complex, high-value molecules with unparalleled precision and efficiency^1–3^. These biological assembly lines achieve their elegance by organizing multiple enzymes within specific cellular micro-environments, overcoming challenges of substrate diffusion, competing reactions, and catalyst degradation^4,5^. Translating this concept to industrial-scale synthesis holds the promise of a more sustainable chemical industry, offering atom-economical routes that operate under mild, aqueous conditions^6–8^. Whole-cell biocatalysis, which utilizes engineered microbes as self-contained and self-replicating micro-factories, is a particularly attractive approach as it avoids the need for costly enzyme purification and provides necessary cofactors in situ^9–12^.

However, the widespread implementation of whole-cell biocatalysis, especially for multi-step cascades involving hydrophobic substrates or products, faces significant hurdles^13–15^. A primary challenge is the poor mass transfer across the phase boundary between the aqueous cellular environment and an immiscible organic phase, leading to low reaction velocities^16–19^. Furthermore, many cascades require the synergy of biological and chemical catalysts (chemoenzymatic reactions), which often operate under incompatible conditions, while direct contact with organic solvents can compromise cell viability and enzyme stability^20–23^. Finally, for any process to be economically viable, the efficient recovery and reuse of both the biological entity and any expensive synthetic co-catalysts is not just desirable, but essential^24–26^.

To address these limitations, various strategies have been explored, including enzyme immobilization on solid supports and the use of conventional biphasic solvent systems^27–30^. While these methods have their merits, they often suffer from diffusion limitations, catalyst leaching, and the inherently low interfacial area of bulk two-phase systems, which throttles productivity^31,32^. Encouragingly, Pickering emulsions stabilized by solid particles adsorbed at the liquid-liquid interface offer a compelling alternative by creating enormous interfacial surface areas^33–36^. In many established systems, Pickering emulsions are stabilized by solid particles, such as inorganic, polymeric, and bioderivatives, which can be strongly anchored at the interface. This not only maintains a large interfacial surface area but also effectively prevents coalescence and promotes interfacial mass transfer^37–39^. Consequently, extensive insightful studies on Pickering emulsions and surfactants have been reported^40–44^. A paradigm shift would be to engineer living-cells and place them precisely at the interface between water and organic phases to perform biocatalytic reactions. However, despite these advances, most existing Pickering-based interfacial biocatalytic systems remain built on static particulate interfaces, offering limited control over interfacial assembly, reconfiguration, and catalyst recovery. These constraints hinder their broader application in complex chemoenzymatic cascades and sustainable, long-term operation.

**Fig. 1:**
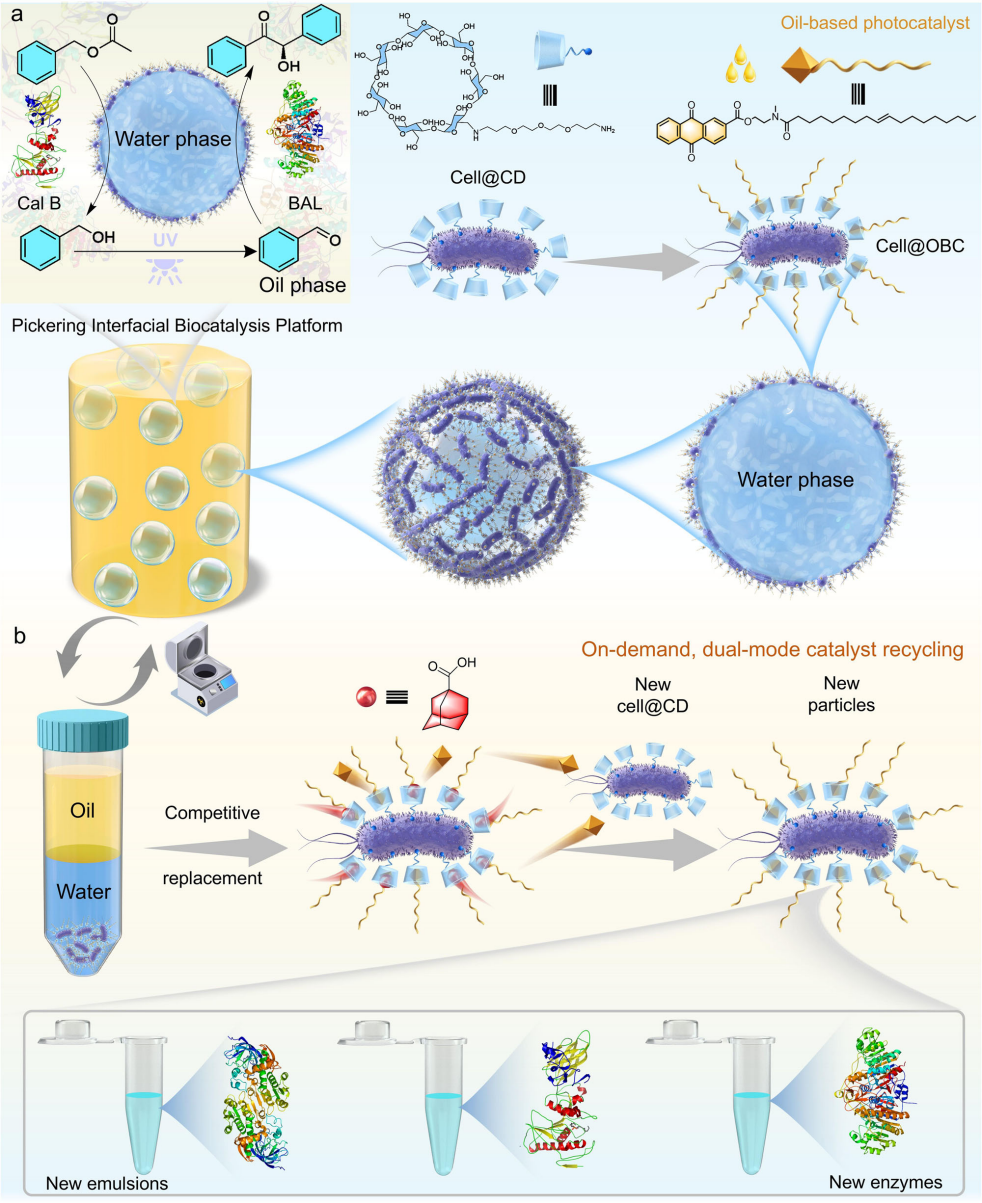
Schematic illustration of a robust Pickering interfacial biocatalysis platform and demonstration of dual recycling strategy. **a** Pickering emulsions based on living cells and oil-based catalysts (OBC) were built to realize interfacial biocatalysis. **b** The well-designed, on-demand, and dual-mode recycling strategy improves atom economy significantly, allowing for sustainable catalysis.

Here, we design and construct a living interfacial biocatalytic platform that goes beyond conventional Pickering emulsion-based systems. By integrating principles of supramolecular chemistry and interfacial engineering, we introduce an “all-in-one” strategy to transform engineered *E. coli* cells into dynamic “suprabacteria” particles that function simultaneously as emulsifiers and active catalytic microreactors. This is achieved through a two-step, modular surface modification: first, the cell membrane is decorated with β-cyclodextrin (β-CD) host molecules. Subsequently, a custom-synthesized, oil-based catalyst (OBC)—which contains a palm fatty acid (PFA) long chain for hydrophobicity and a photoactive anthraquinone head group—is attached as a guest via host-guest interaction (Fig. 1a). Unlike permanently fabricated Pickering particles, this supramolecular surface chemistry imparts tunable amphiphilicity and dynamic reconfigurability, enabling the living cells to self-assemble at the water–oil interface and stabilize robust Pickering emulsions without the need for high-shear mechanical processing that would damage the cells.

This “living emulsion” architecture provides a programmable interfacial environment for assembly-line biotransformations. We demonstrate its remarkable efficacy and versatility across a suite of single-step bioconversions and complex, multi-step enzymatic cascades, including chemo- and photoenzymatic reactions. While the enlarged interfacial area accelerates reaction rates relative to conventional biphasic systems, a defining feature of our approach is the dynamic and reversible nature of the supramolecular host–guest linkage, which enables a unique, on-demand recycling strategy. Specifically, the conjugates (cell@OBC) can be directly reused for continuous catalysis. Alternatively, when cellular activity declines, the valuable OBC photocatalyst can be selectively displaced, recycled, and reassembled onto a new batch of fresh, living cells, thus maximizing atom economy to uphold the principles of green chemistry (Fig. 1b). By separating photocatalyst recovery from the fate of the living cell-based carrier, this strategy overcomes a key limitation of conventional Pickering systems based on static materials. Finally, the practicality of this strategy is confirmed by scaling the process to produce gram-scale quantities of a high-value chemical, demonstrating a viable pathway from laboratory design to potential industrial application.

## Results and discussion

### Design and synthesis of living amphiphilic biocatalysts

Our strategy commenced with the modular synthesis of the key components: an aminated β-cyclodextrin (ACD) host and a tailored OBC guest (Supplementary Fig. 1). The successful synthesis and purity of both molecules were rigorously confirmed by ¹H NMR, TLC-MS, and FTIR spectroscopy (Supplementary Figs. 2, 3, 4, and 5). With these building blocks in hand, we proceeded to engineer the surface of living *E. coli* BL21 (DE3) cells. The construction of the “suprabacteria” particles was a two-step process executed under mild, biocompatible conditions^45,46^. First, we covalently grafted the ACD host onto the cell surface via coupling agents, 1-(3-dimethylaminopropyl)-3-ethylcarbodiimide (EDC) and *N*-hydroxy succinimide (NHS), yielding the cell@CD conjugates (Supplementary Fig. 6). Second, the OBC guest was introduced, spontaneously assembling onto the cell surface through host-guest inclusion within the cyclodextrin cavity to form the final cell@OBC particles. The successful, non-covalent attachment of the OBC was confirmed by UV-Vis spectroscopy, which revealed the characteristic absorption of the anthraquinone moiety at 330 nm for cell@OBC^47,48^, a peak absent in control mixtures of native cells and free OBC (Supplementary Fig. 7). This result provides clear evidence for a stable supramolecular linkage. Critically, we verified that the photocatalytic activity of the anthraquinone unit was unimpeded by its conjugation into the OBC structure, providing an excellent starting point for our investigations (Supplementary Fig. 8).

**Fig. 2:**
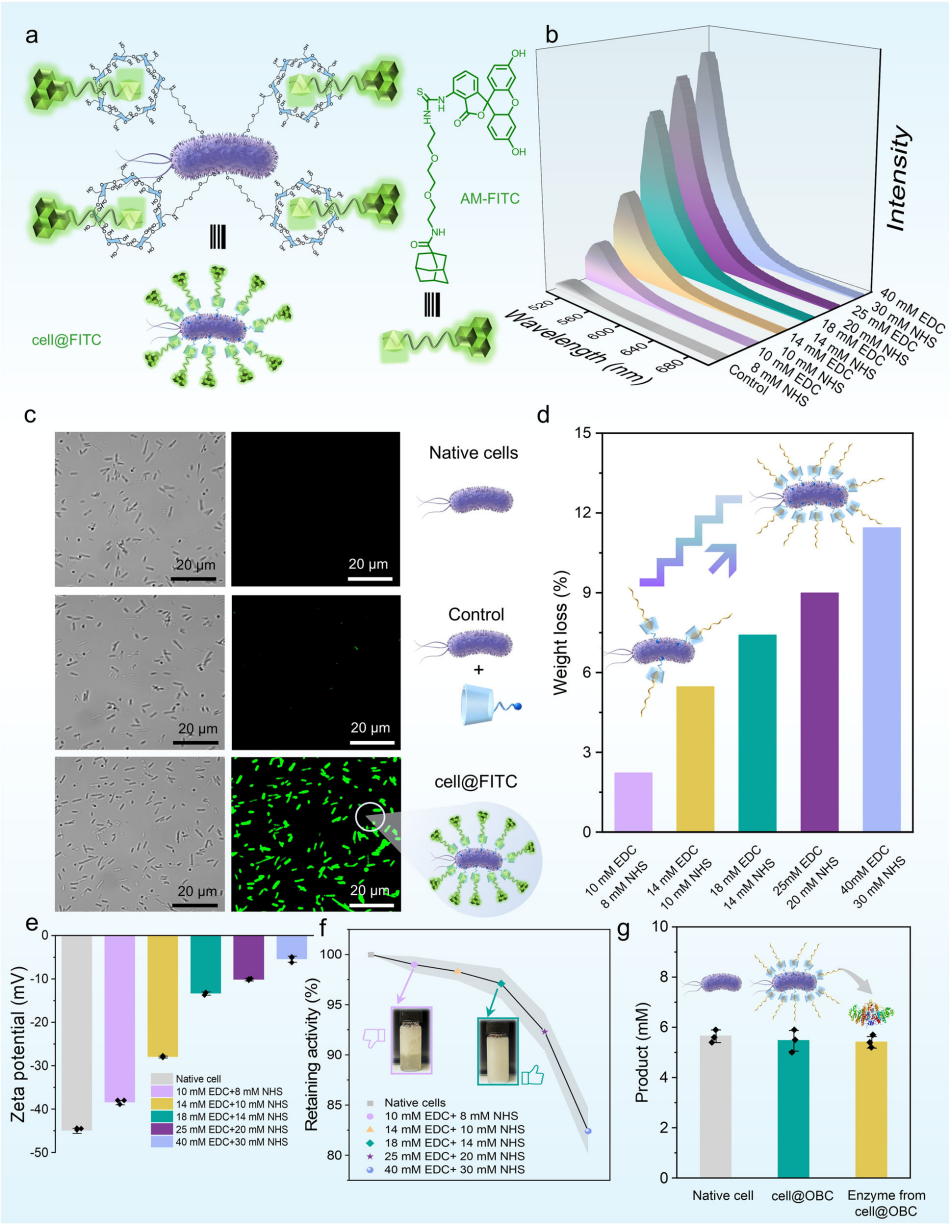
Proof of concept of modified *E. coli* cells and tunable surface. **a** Schematic of cell@CD fluorescence labeling. **b** The concentration of the coupling agent was positively correlated with the fluorescence intensity on the coupling surface, and a mixture of cells and ACD served as the control. **c** Bright-field and fluorescence images of native cells, control group (cells mixed with ACD) and cell@CD. The experiment was repeated three times independently with similar results. **d** With increasing amounts of coupling agent, the weight loss of cell@OBC increased over the 0-800 °C range compared to the native cells. **e** Zeta potential of native cells and cell@OBC modified with different amounts of coupling agent. Data are presented as mean values +/- SD based on *n* = 3 independent replicates with the error bars representing the SD. **f** Effect of the concentration of coupling agents on the BAL enzymatic retaining activity and emulsion stability. The error bars represent the standard deviations of three parallel measurements, *n* = 3. **g** The catalytic activity of native cells, cell@OBC, and enzyme lysed from cell@OBC. Data are presented as mean values +/- SD based on *n* = 3 independent replicates with the error bars representing the SD.

To visualize the surface modification and optimize its density, we fluorescently labelled the cell@CD conjugates using an adamantane-tagged fluorescein dye (AM-FITC), which binds specifically to the surface-grafted cyclodextrin cavities (Fig. 2a). A central principle of our design was to tune the surface chemistry while preserving cell viability and function. By varying the concentration of the coupling reagents (EDC/NHS), we could precisely control the density of β-CD on the cell surface, as quantified by the corresponding increase in fluorescence intensity (Fig. 2b). Meanwhile, confocal laser scanning microscopy (CLSM) revealed a distinct green fluorescent around the cell@CD conjugates, which was absent on native cells or control cells merely mixed with the dye, confirming the successful covalent attachment of the β-CD host (Fig. 2c). Furthermore, the weight loss difference between modified and native cells increased monotonically, revealing the tunability of surface grafting (Fig. 2d and Supplementary Fig. 9). Subsequently, the ζ-potential was characterized to assess surface charge, showing a gradual shift from −45 mV in native cells to −6 mV with increasing supramolecular loading (Fig. 2e and Supplementary Fig. 10). These findings indicate that the supramolecular layer on the cell surface can be precisely regulated to adapt to diverse functional requirements.

However, this functionalization revealed an inverse relationship between the degree of surface modification and the retention of intracellular enzymatic activity. We assessed the impact on intracellular enzyme function using *E. coli* overexpressing benzaldehyde lyase (BAL) and found that excessive surface functionalization, while increasing hydrophobicity, significantly diminished the enzyme’s retaining activity (Fig. 2f). This decrease is likely attributable to mass transport limitations imposed by a dense surface layer or potential chemical stress. Conversely, insufficient functionalization failed to provide the necessary amphiphilicity to stabilize an emulsion (Fig. 2f, inset). Through systematic optimization, we identified that coupling conditions of 18 mM EDC and 14 mM NHS struck the optimal balance, affording robust emulsification properties while maintaining over 97% of the intracellular enzyme activity. To further confirm that the attachment of the supramolecular layer to the cell surface does not introduce additional mass transfer barriers, we lysed the cell@OBC to release free BAL enzyme and compared its activity with the cell@OBC and native cells. The results showed nearly no difference among the three (Fig. 2g and Supplementary Fig. 11), strongly supporting our hypothesis and providing a solid foundation for subsequent research.

### Enhanced cellular robustness and biocompatibility

**Fig. 3:**
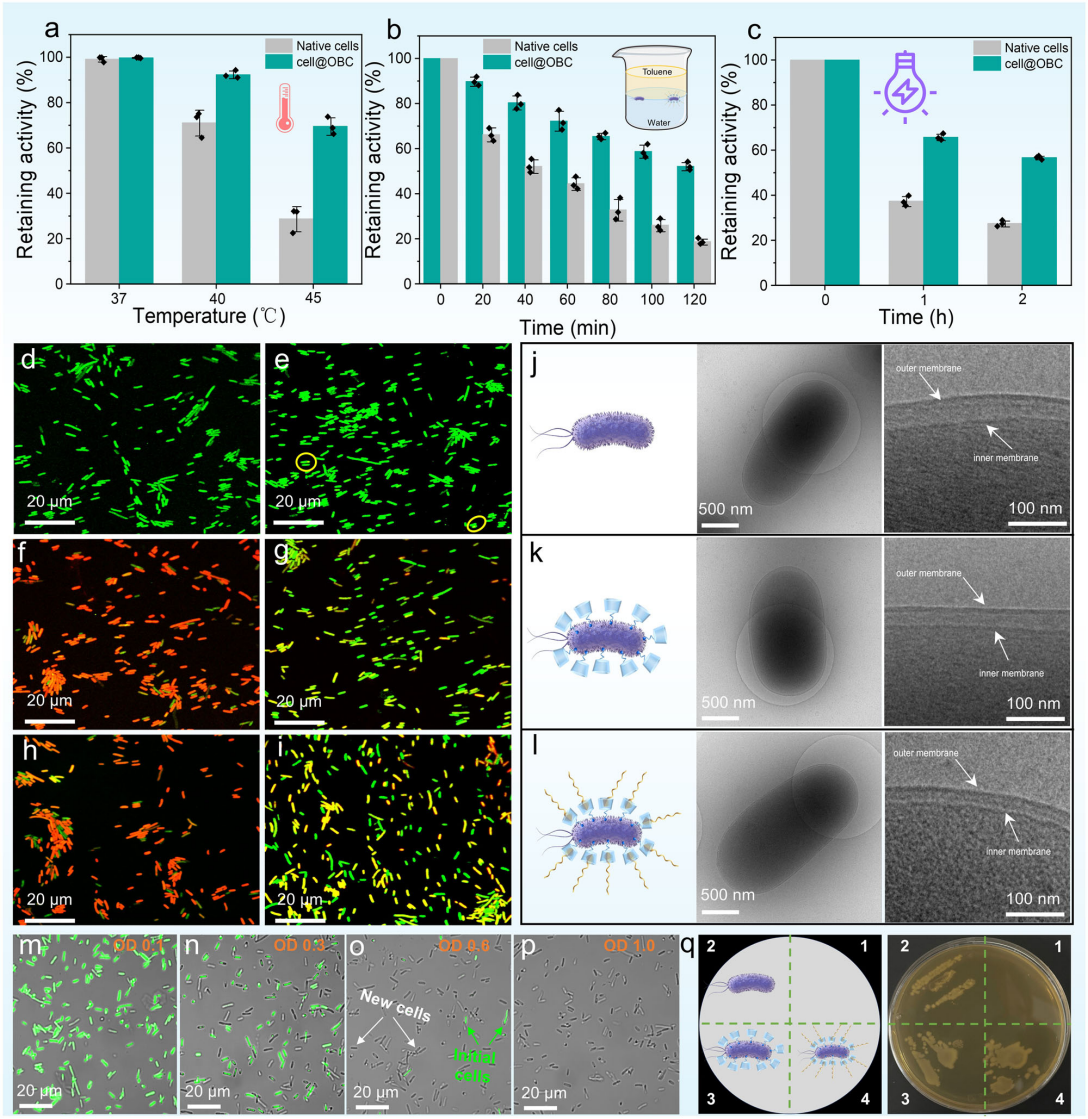
Evaluation of the viability of modified *E. coli* cells under external stress conditions. **a** Stability of live cells overexpressing BAL under heat treatment; gray columns-native cells; green columns-cell@OBC. Data are presented as mean values +/- SD based on *n* = 3 independent replicates with the error bars representing the SD. **b** Stability of live cells overexpressing BAL under interfacial stress; gray columns-native cells; green columns-cell@OBC. Data are presented as mean values +/- SD based on *n* = 3 independent replicates with the error bars representing the SD. **c** Retaining activity of native cells (gray) and cell@OBC (green) measured after 0, 1, and 2 h of continuous UV exposure. Data are presented as mean values +/- SD based on *n* = 3 independent replicates with the error bars representing the SD. Live/dead assay of (**d**) native cells and (**e**) fresh cell@OBC; green - live cells; red - dead cells; live/dead assay of heat-treated (45 °C, 2 hours) cells: (**f**) native cells and (**g**) cell@OBC; live/dead assay of (**h**) native cells and (**i**) cell@OBC after 2 hours UV treatment. The experiment was repeated three times independently with similar results. Representative cryo-TEM images of native cells (**j**), cell@CD (**k**), cell@OBC (**l**), and the enlarged details of cell structures to assess the biocompatibility of the modification strategies. Growth images for cell@CD with OD_600_ 0.1 (at the beginning of re-culture) (**m**), OD_600_ 0.3 (**n**), OD_600_ 0.6 (**o**) and OD_600_ 1.0 (**p**). **q** Schematic representation of the plating assay and the image of the plating assay, showing native cells (section 2), cell@CD (section 3) and cell@OBC (section 4), section 1 is blank control.

A key advantage of whole-cell catalysis is the protective environment the cell provides for enzymes^49–52^. We hypothesized that our surface modification would further shield the cells from the harsh conditions often encountered in processes. To test this, we subjected the cell@OBC conjugates to thermal and chemical stressors. When exposed to 45 °C for two hours, the cell@OBC retained 69.6% of their initial intracellular BAL activity, a 2.4-fold improvement over unmodified native cells (Fig. 3a). Even more strikingly, when challenged with a toluene-water interface—a condition notorious for inducing cell lysis—the cell@OBC conjugates maintained 52.2% of their activity after two hours, whereas the activity of native cells significantly dropped, demonstrating a 2.8-fold enhancement in stability (Fig. 3b). Other solvents like *N, N*-dimethylformamide (DMF), isopropanol, and dimethylsulfoxide (DMSO) were also tested to further consolidate the protectability of the polymers against organic solvents (Supplementary Fig. 12). Furthermore, after 2 hours of UV exposure, the retaining activity of cell@OBC remained 2.1 times higher than natural cells (Fig. 3c and Supplementary Fig. 13). These findings were corroborated by live/dead staining assays, which visualized the superior viability of cell@OBC conjugates under both heat and organic solvent stress compared to their native counterparts (Fig. 3d-i).

To ensure that our surface engineering did not compromise cellular integrity, we employed cryogenic transmission electron microscopy (cryo-TEM). The images revealed that native cells, cell@CD, and cell@OBC all possessed intact inner and outer membranes^53^, confirming that our mild, two-step modification strategy is biocompatible and does not cause morphological damage (Fig. 3j-l and Supplementary Fig. 14). The ultimate test of biocompatibility, however, is the ability to proliferate. We re-cultured fluorescently labelled cell@CD and monitored their growth. As the optical density (OD₆₀₀) increased, the fluorescence per cell progressively diluted, indicating that the surface-modified cells were actively dividing (Fig. 3m-p, Supplementary Figs. 15, 16). Notably, while the replicated cells no longer exhibit emulsification capabilities due to the loss of surface hydrophobicity, their recyclability remains intact (Supplementary Fig. 17). This proliferative capacity was further confirmed by plating assays, where native cells, cell@CD cells, and cell@OBC all formed healthy colonies after 48 hours of incubation (Fig. 3q). Collectively, these results establish that our supramolecular engineering approach yields robust, viable, and functionally unimpaired living catalysts poised for interfacial applications.

### Living cells as active stabilizers for Pickering emulsions

**Fig. 4:**
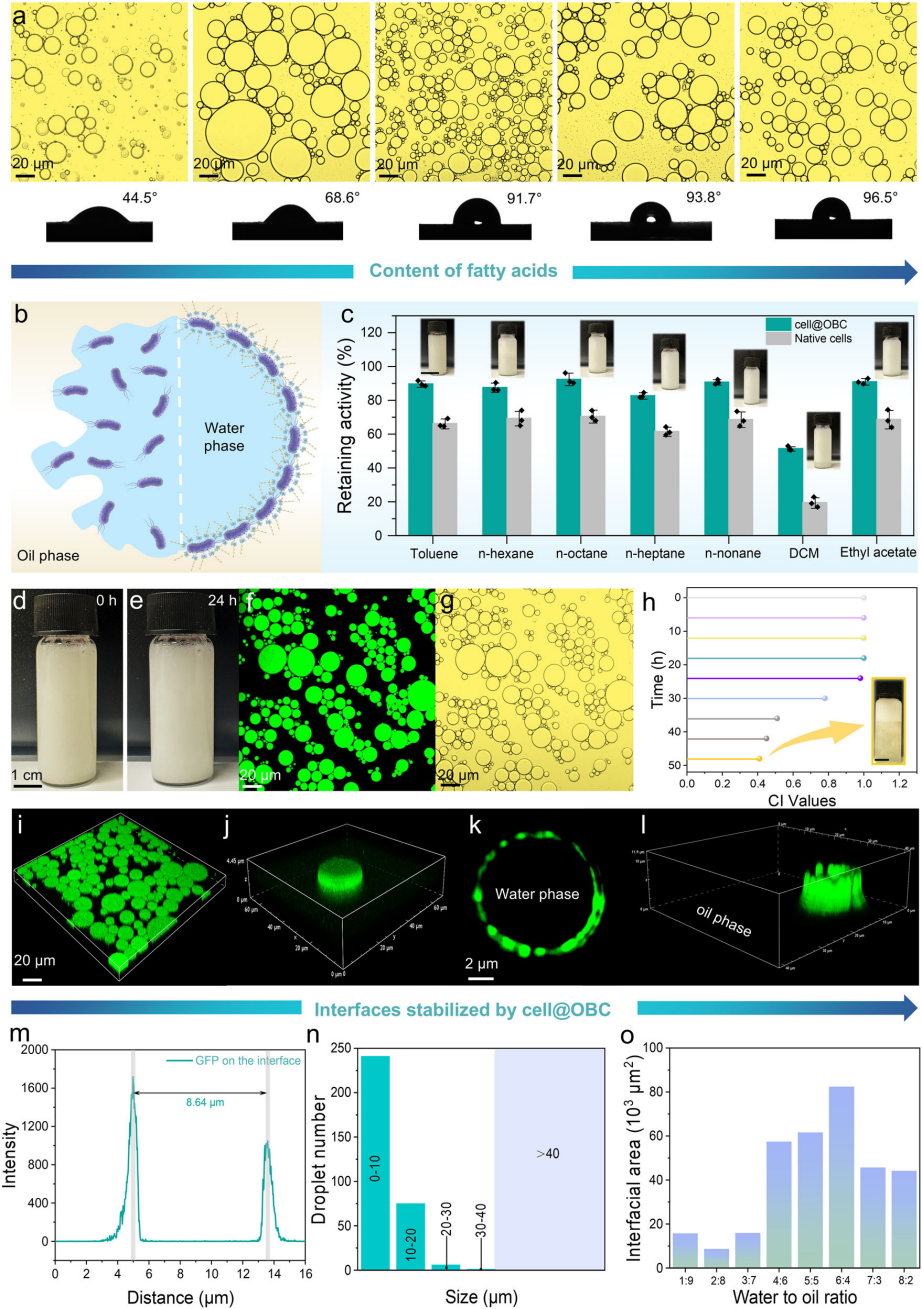
Characterization and interfacial stability of Pickering emulsions. **a** Effect of coupling agent concentration on emulsion droplet morphology and cell@OBC hydrophobicity. The experiment was repeated three times independently with similar results. **b** Scheme of native cells (left) and cell@OBC (right) under interfacial stress (i.e., toluene–water biphasic condition). **c** cell@OBC exhibits excellent resistance in a wide range of solvents; cell@OBC is compatible with a wide range of organic solvents to form stable emulsions (inset, scale bar: 1 cm). Data are presented as mean values +/- SD based on *n* = 3 independent replicates with the error bars representing the SD. Emulsion appearance at 0 h (**d**) and 24 h (**e**). **f** Confocal laser scanning microscopy (CLSM) image of the water-in-oil emulsions; green–water phase. **g** Optical microscopy image of the emulsions. **h** CI values of emulsion with different storage times; after 48 hours of storage, the emulsion tends to the final state (inset, scale bar: 0.5 cm). 3D CLSM (**i**) and magnified detail (**j**) of droplets after 24 hours of emulsion storage. **k** 2D CLSM of emulsions stabilized with cell@OBC containing GFP proteins. **l** 3D CLSM as further evidence that cell@OBC exists between the water-oil interface to stabilize emulsions. **m** Fluorescence intensity of GFP proteins at the interface as additional evidence of emulsion particle size. **n** The number and size distribution of emulsion droplets stabilized by cell@OBC (water-to-oil ratio = 6:4). **o** The interfacial area of emulsion droplets stabilized by cell@OBC with different water fractions from 10% to 80%.

Having engineered cells with the requisite amphiphilicity, we next investigated their ability to stabilize Pickering emulsions. The degree of hydrophobicity, controlled by the density of the grafted OBC, was paramount. We observed a clear correlation between the surface density of the fatty acid chains and the cells’ ability to stabilize a toluene-in-water emulsion (Supplementary Fig. 18), with a water contact angle increasing from 25.4° for native cells to 96.5° for the most densely coated cells (Fig. 4a and Supplementary Fig. 19). Based on our optimization, we proceeded with cell@OBC particles prepared under the ideal conditions (18 mM EDC, 14 mM NHS) (Fig. 4b). Remarkably, stable Pickering emulsions were formed by simple handshaking—without the need for high-energy homogenization or sonication—thereby preserving cellular integrity (Supplementary Fig. 20). The versatility of this system was demonstrated by its ability to form stable emulsions with a wide array of organic solvents, including n-hexane, n-octane, and ethyl acetate, while maintaining high intracellular enzyme activity (Fig. 4c).

We systematically optimized the emulsion formulation by varying the water-to-oil volume ratio. A ratio of 6:4 (v/v) yielded an exceptionally stable emulsion that showed no signs of creaming or phase separation even after 24 hours (Figs. 4d, 4e, and Supplementary Movie 1). Using a water-soluble fluorescent dye, we identified the emulsion type as water-in-oil (W/O) (Figs. 4f, 4g and Supplementary Fig. 21). Analysis of the creaming index (CI) over time confirmed the system reached a stable equilibrium within 48 hours (Fig. 4h and Supplementary Fig. 22). Three-dimensional (3D) CLSM reconstructions revealed a well-defined architecture of tightly packed, spherical aqueous droplets (Figs. 4i, 4j). To confirm that the cell@OBC particles were indeed acting as the interfacial stabilizers, we engineered the cells to express green fluorescent protein (GFP). The resulting CLSM images clearly showed the fluorescent cells forming a continuous, stabilizing layer at the oil-water interface (Figs. 4k, 4l). Quantitative analysis of droplet size distribution revealed that the optimal 6:4 ratio produced the highest number of droplets with the smallest mean diameter (≈ 8.64 µm), which in turn generated the largest total interfacial area (Figs. 4m, 4n, and Supplementary Fig. 23). At low water fractions, fewer aqueous droplets form and coalescence leads to larger droplets, reducing total interfacial area. At high water fractions, droplet packing/coalescence and partial loss of the W/O structure increase mean droplet size, again decreasing total interfacial area. The 6:4 ratio provides an optimal balance. This maximization of the interfacial area (Fig. 4o) is the key physical parameter that underpins the enhanced catalytic performance of the system.

### Accelerated single-step interfacial biocatalysis

**Fig. 5:**
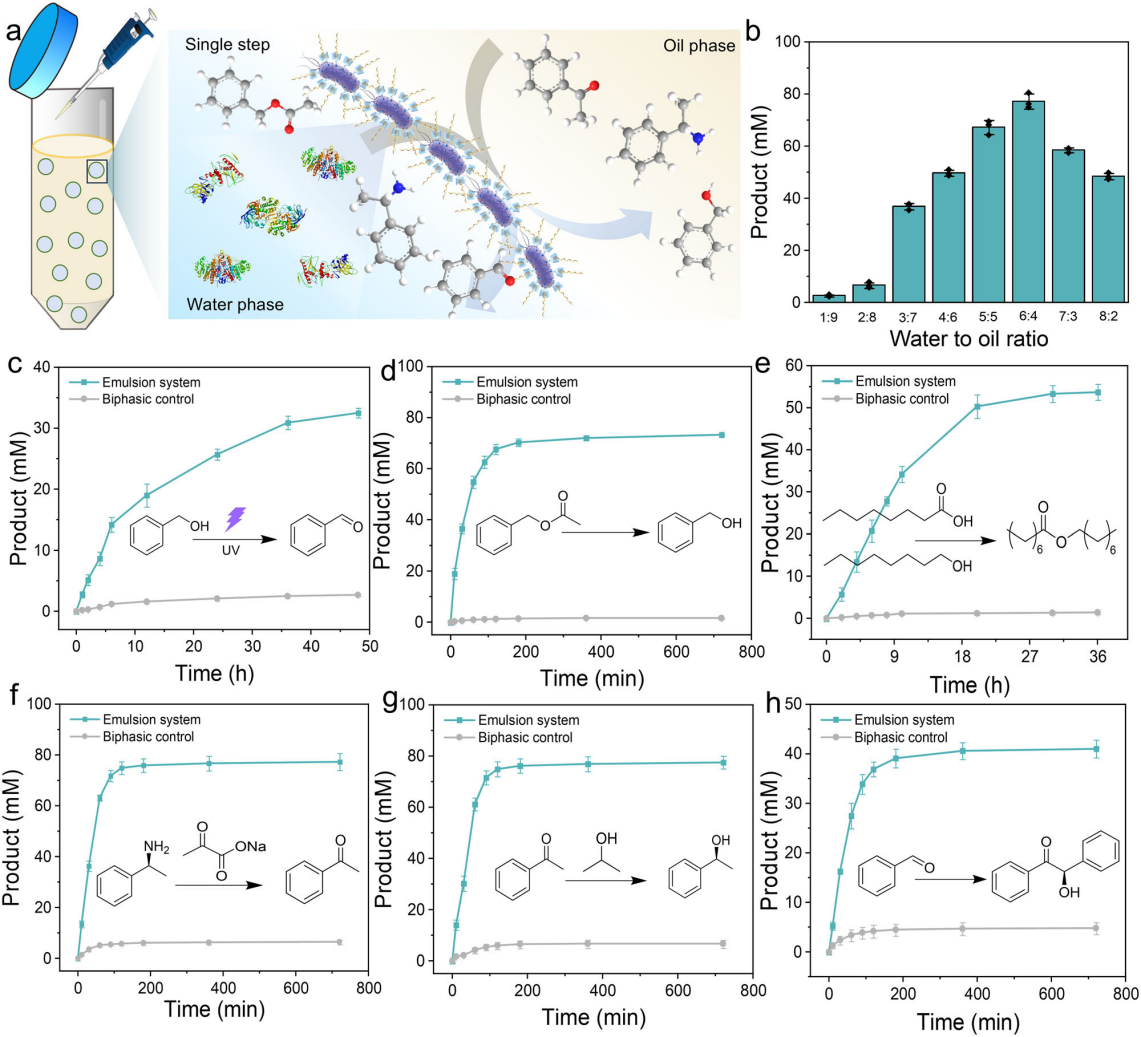
Investigation of single step interfacial biocatalysis based on Pickering emulsions. **a** Visualization of interfacial biocatalytic processes stabilized by cell@OBC. **b** BAL enzyme activity of emulsions with different water-to-oil ratios. **c** OBC as a photocatalyst for the conversion of benzyl alcohol to benzaldehyde (green), and the biphasic system was used as a negative control (gray). Cal B-catalyzed hydrolysis (**d**) and esterification reactions (**e**) demonstrate the diversity of enzymatic reactions in emulsions. Three different enzymatic reactions were extended in emulsion by overexpressing ATA (**f**), RR ADH (**g**) and BAL (**h**) inside living cells. The results in (**b**–**h**) are the average values of three parallel experiments. The error bars represent the standard deviations of three parallel measurements, *n* = 3.

With a stable and well-characterized living emulsion platform in hand, we set out to demonstrate its catalytic prowess. The system is designed to position the intracellular enzymes within the aqueous droplets, while the photocatalytic OBC resides at the interface, perfectly situated to interact with hydrophobic substrates in the continuous oil phase (Fig. 5a). The catalytic efficiency is directly proportional to the available interfacial area, and as predicted, the bioconversion of benzaldehyde by BAL peaked at the 6:4 water-to-oil ratio that provided the maximum interface (Fig. 5b).

Prior to exploring a series of chemo- and biocatalytic reactions, we quantified the loading amount of OBC in a 1 mL emulsion system as 0.23 mg via a calibration curve (Supplementary Fig. 24). Then, we consistently compared the emulsion system to a conventional biphasic system with an equivalent amount of catalyst. For the photocatalytic oxidation of benzyl alcohol to benzaldehyde, driven by the OBC at the interface, the emulsion system yielded 32 mM of product after 48 hours—a 11.8-fold increase in productivity over the biphasic control (Fig. 5c). The macroscopic color change of the emulsion from white to chartreuse provided a striking visual indicator of the reaction’s progress (Supplementary Fig. 25). Additionally, an extra photocatalytic reaction was performed under alternating UV conditions, demonstrating the unique advantage of photocatalytic controllability (Supplementary Fig. 26). In another example, the hydrolysis of benzyl acetate by *E. coli* overexpressing *Candida antarctica* lipase B (Cal B) produced 73 mM of benzyl alcohol, a remarkable 45-fold rate enhancement compared to the biphasic control (Fig. 5d). The platform was equally effective for esterification reactions (Fig. 5e). To showcase its generality, we successfully deployed the system for transformations using three other enzymes: Amine transaminase (ATA), an alcohol dehydrogenase from *Rhodococcus ruber* (RR ADH), and BAL, achieving high conversions (77-83%) in each case (Fig. 5f-h and Supplementary Fig. 27). The rapid reaction kinetics, with most conversions approaching their maximum within just 3 hours, highlight the high efficiency of this interfacial biocatalytic architecture, which effectively eliminates the mass transfer limitations that plague traditional biphasic systems.

### Assembly-line catalysis in scalable multienzyme cascades

**Fig. 6:**
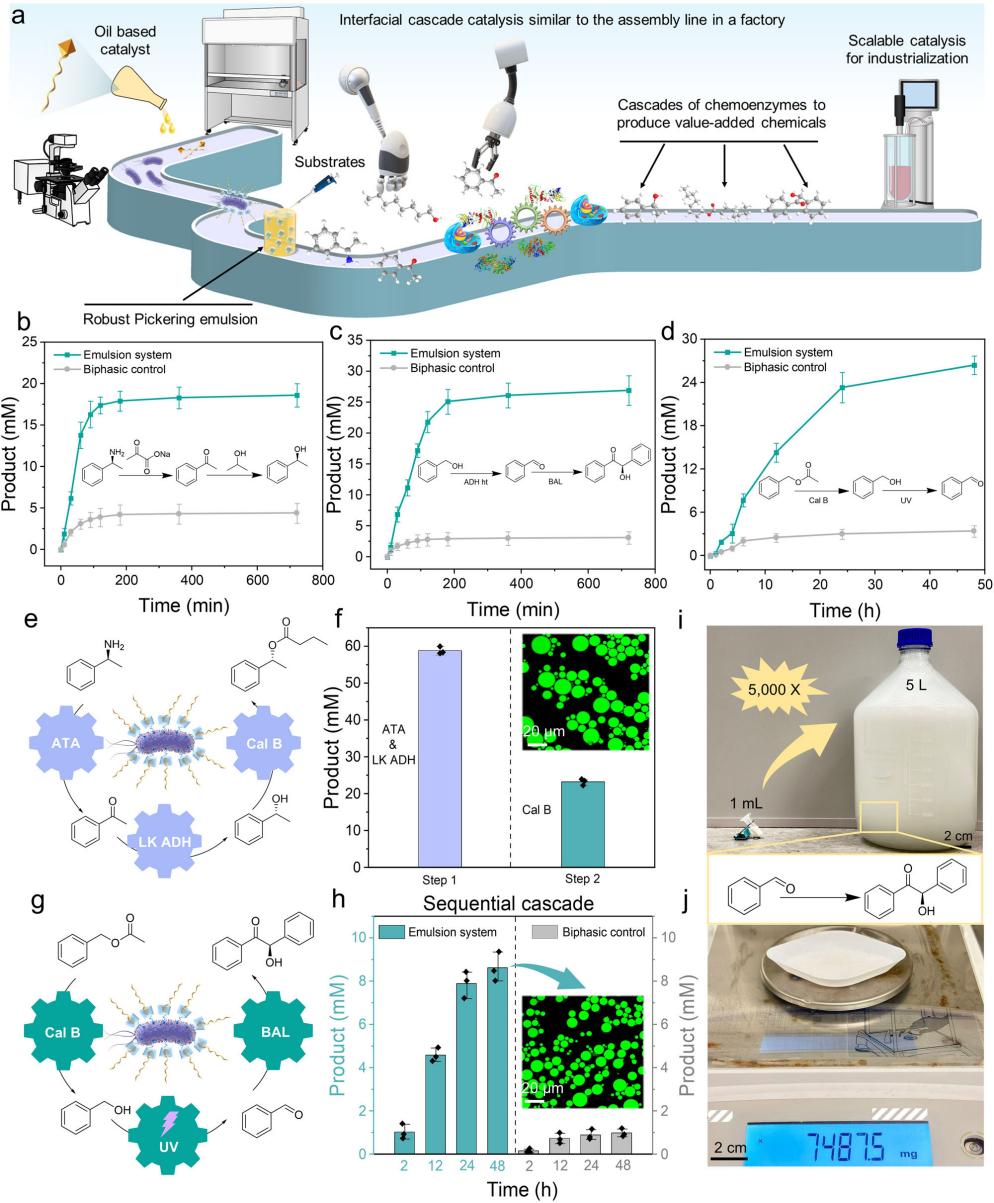
Photoenzymatic catalysis and multienzymatic catalysis in Pickering emulsions. **a** A scheme of tailored cascade catalysis assembly line built to enable conversion from simple substrates to high value-added chemicals. **b** The ATA and RR ADH were used as the first explorations of the cascade reaction. **c** The extendable multienzyme cascade catalysis protocol was validated by the combination of two enzymes, ADH ht and BAL. **d** The combination of Cal B and the photo-responsive unit on the surface of cell@OBC opens the possibility of photoenzymatic cascade catalysis. **e** A schematic representation of three-step sequential cascade catalysis based on ATA, LK ADH and Cal B. **f** Catalytic efficiency of the first and second steps in a sequential cascade; emulsion system remains stable after reaction (inset, scale bar: 20 μm). The experiment was repeated three times independently with similar results. **g** OBC, Cal B and BAL mediated one-pot cascade catalysis scheme. **h** The catalytic efficiency of one-pot cascade reactions as a function of time; CLSM image of the reacted emulsion system (inset, scale bar: 20 μm). The experiment was repeated three times independently with similar results. **i** 5 L of the enlarged emulsion was prepared and the catalysis was performed. **j** Approximately 7.5 g of purified benzoin was obtained. The results in (b–d, f, and h) are the average values of three parallel experiments. The error bars represent the standard deviations of three parallel measurements, *n* = 3.

The true potential of this platform lies in its ability to orchestrate complex, multi-step reactions, mimicking the spatial organization of metabolic pathways. We designed our system to function as a programmable, factory-like assembly line for the synthesis of value-added chemicals (Fig. 6a). We first demonstrated a two-step cascade by co-stabilizing an emulsion with two different *E. coli* populations, one overexpressing ATA and the other RR ADH. This two-enzymatic system efficiently converted (*S*)-1-phenylethylamine first to acetophenone and subsequently to (*S*)-1-phenylethanol, yielding 19 mM of the final product—a 4.3-fold increase over the biphasic control (Fig. 6b). The platform’s modularity was further shown in a cascade producing benzoin using  alcohol dehydrogenase from *Bacillus stearothermophilus* (ADH ht) and BAL, which again dramatically outperformed the control system (Fig. 6c). We then integrated chemical and biological catalysis in a photoenzymatic cascade, where benzyl acetate was first hydrolysed to benzyl alcohol by Cal B, which was then photocatalytically oxidized to benzaldehyde by the interfacial OBC, all within a single emulsion (Fig. 6d).

Encouraged by these results, we further explored a more ambitious three-step cascade. We first demonstrated a sequential cascade where an emulsion co-stabilized by ATA and *Lactobacillus kefir* ADH (LK ADH)-expressing cells converted (*S*)-1-phenylethylamine to (*R*)-1-phenylethanol (59 mM yield) (Figs. 6e, 6f). The crude product was then directly introduced into a second, fresh emulsion stabilized by Cal B-expressing cells, which performed a transesterification to yield 1-phenylethyl butyrate (23 mM). More impressively, we achieved a one-pot, three-step photoenzymatic cascade. An emulsion co-stabilized by cells expressing Cal B and BAL was used to convert benzyl acetate sequentially to benzyl alcohol (by Cal B), then to benzaldehyde (by the photocatalytic OBC), and finally to (*R*)-benzoin (by BAL) (Fig. 6g). This integrated system produced 8.6 mM of (*R*)-benzoin, whereas the product was barely detectable in the biphasic control (Fig. 6h). Throughout these complex transformations, the emulsions remained remarkably stable (Fig. 6h, inset).

Finally, to prove the application relevance of our platform, we demonstrated its scalability. The reaction rate could be precisely tuned by controlling the cell density (OD₆₀₀) (Supplementary Fig. 28). Moreover, the substrate scope was further evaluated. Two representative enzymes (ATA and RR ADH) were overexpressed in *E. coli* to perform both single-step catalysis and cascade reactions. Specifically, the ATA single-step module was validated on five structurally distinct amine substrates, giving conversions spanning 28-97% (entries 1-5, Supplementary Table 2, Supplementary Figs. 29-37), which directly demonstrates that the living cell Pickering microreactor is not limited to a single aromatic substrate but can accommodate substituent variation. Additionally, the cascade reaction was further evaluated on five substrates, delivering secondary alcohol products with high stereoselectivity in most cases (*ee* value up to 99%) together with conversions from 19-93% depending on substrate structure (entries 1-4, Supplementary table 3, Supplementary Figs. 38-51), supporting the platform’s capability for enantioselective cascade synthesis in the interfacial setting rather than only single-enzyme turnover. Next, we are interested in exploring the scalable potential of this platform. First, we pre-scaled up the BAL-catalysed synthesis of benzoin to a 600 mL emulsion system (Supplementary Figs. 52, 53 and Supplementary Movie 2). This preparative-scale reaction successfully yielded approximately 1 gram of purified benzoin powder. Subsequently, we further scaled up the system to 5 L and obtained more benzoin (7.48 g) (Figs. 6i, 6j and Supplementary Fig. 54), providing compelling evidence that this living emulsion technology is a viable and powerful platform for sustainable chemical production.

### Sustainable catalysis via on-demand dual-mode recycling

**Fig. 7:**
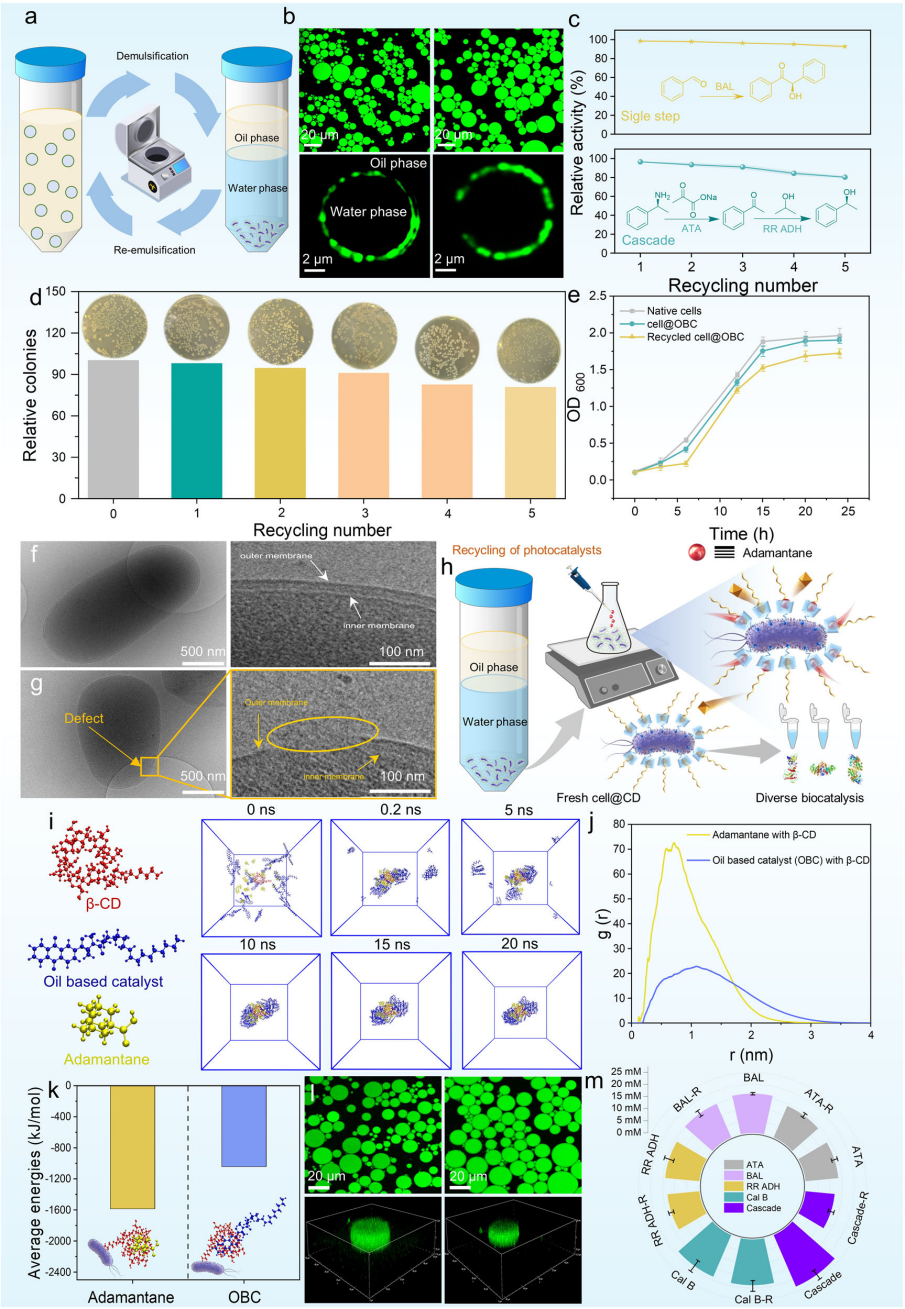
On-demand recycling strategy as a reliable candidate for sustainable continuous catalysis. **a** Scheme demonstration of whole-cell recycling. **b** Droplet morphology under CLSM of the Pickering emulsion before recycling (left side, top) and the new emulsion (right side, top, scale bar: 20 μm) formed by the cell@OBC after recycling; emulsions before and after recycling were stabilized by cell@OBC, which overexpresses GFP protein (down). The experiment was repeated three times independently with similar results. **c** The cell@OBC after 5 times of recycling still maintains the high catalytic activity in single-step and cascade catalysis. **d** Relative colonies of cell@OBC after different times of recycling; plating assays of cell@OBC after different times of recycling (inset). **e** The cell growth curves of native cells (gray), fresh cell@OBC (green) and recycled cell@OBC (yellow). Data are presented as mean values +/- SD based on *n* = 3 independent replicates with the error bars representing the SD. **f** Representative cryo-TEM images of fresh cell@OBC and cellular structure in enlarged detail. **g** Representative cryo-TEM images of recycled cell@OBC and cellular structure in enlarged detail. **h** Showcase of schemes for recycling photocatalysts from Pickering emulsion systems. **i** Molecular dynamics simulations were used to evaluate the interaction and bonding processes of the host with multiple guest molecules. **j** Radial distribution function between two guest and host molecules. **k** The average energies of two different host-guest interaction systems. **l** CLSM images of emulsion before recovery and CLSM images of new emulsion formed by OBC with fresh cells (top); 3D CLSM images of the droplets from the previous emulsion and the new emulsion. The experiment was repeated three times independently with similar results. **m** Four single-step catalysis reactions and one cascade catalysis were performed by the new emulsion after recycling to emphasize the sustainability of the catalytic strategy.

Low-energy preparation processes, eco-friendly solvents, and catalyst recycling and reuse represent the cornerstones of green chemistry^54,55^. Therefore, the emulsions were prepared using three different methods: handshaking, vortexing, and sonication. The results showed no significant difference in emulsion stability or catalytic efficiency (Supplementary Fig. 55). Furthermore, three eco-friendly solvents, represented by ethyl acetate, were used to replace toluene in emulsion preparation, also yielding positive results (Supplementary Fig. 56). This strongly demonstrates the system’s versatility and sustainability. Our platform’s design, based on dynamic supramolecular bonds, uniquely facilitates a flexible, on-demand recycling strategy. We first demonstrated the recovery of the entire living conjugate. After a catalytic run, the cell@OBC particles were easily recovered by centrifugation, washed, and redispersed to form a new, stable emulsion for a subsequent reaction cycle (Fig. 7a). CLSM images confirmed that the recycled cells not only retained the ability to stabilize the interface, but also that cell@OBC could still be reversibly attached to the interface via handshaking even after five recycling cycles. (Fig. 7b and Supplementary Fig. 57). We subjected the system to five consecutive cycles of single-step (BAL) and two-step cascade (ATA/RR ADH) reactions. Remarkably, the catalysts retained over 92% and 80% of their initial activity, respectively, showcasing their excellent operational stability (Fig. 7c). Furthermore, plating assays confirmed that the cells remained highly proliferative even after five recycling rounds, with only a minor decrease in colony formation (Fig. 7d). The growth curves of recycled cells were nearly identical to those of native cells, indicating sustained viability (Fig. 7e). Cryo-TEM analysis of the recycled cells revealed that while the majority remained morphologically intact, some minor membrane defects were observable, which likely accounts for the modest decline in catalytic activity over repeated cycles (Figs. 7f, 7g).

While recycling the entire living conjugate is effective, the finite lifespan of the cells presents a limitation. Our supramolecular design offers a more sophisticated solution: the selective recycling of the synthetic photocatalyst. We hypothesized that the OBC guest could be displaced from the β-CD host by introducing a competitive guest with a higher binding affinity, such as adamantane (Fig. 7h). Molecular dynamics simulations strongly supported this concept, showing that adamantane rapidly and preferentially occupies the β-CD cavity, expelling the OBC (Fig. 7i and Supplementary Fig. 58). The calculated radial distribution function and average energies confirmed a significantly stronger interaction for the adamantane-CD pair (-1,586 kJ mol⁻¹) compared to the OBC-CD pair (-1,054 kJ mol⁻¹), providing a thermodynamic driving force for the exchange (Figs. 7j, 7k). We validated this experimentally by treating the used cell@OBC conjugates with 1-adamantanecarboxylic acid. This process successfully liberated the OBC, which was recycled (~250 mg from a 1 L emulsion) and its chemical identity confirmed (Supplementary Fig. 59, 60 and 61). This recovered OBC was then reassembled onto fresh cell@CD conjugates expressing a variety of enzymes. These newly formed living catalysts successfully stabilized emulsions (Fig. 7l) and exhibited catalytic efficiencies in four different single-step reactions and one cascade reaction that were nearly identical to the original system (Fig. 7m). We illustrate both recycling processes in Supplementary Fig. 62 to highlight the efficiency and selectivity of catalyst reuse. This dual-mode recycling capability represents a significant advance, enabling a truly circular and sustainable catalytic process where both the biological and chemical components can be managed for maximum value and minimal waste.

In summary, we have developed a versatile and robust platform for chemoenzymatic catalysis by engineering the surface of living cells with a modular, supramolecularly-linked, oil-based catalyst. This strategy transforms the cells into active, living emulsifiers that self-assemble at the water-oil interface, creating a highly stable Pickering emulsion. This unique architecture effectively overcomes the critical mass-transfer limitations inherent in conventional biphasic systems, dramatically accelerating a wide range of single-step biotransformations and complex, multi-step enzymatic cascades. The system functions as a programmable, factory-like assembly line, enabling the efficient synthesis of high-value chemicals, which we have demonstrated with the gram-scale production of benzoin.

Crucially, the dynamic nature of the supramolecular linkage endows the platform with an elegant, on-demand, dual-mode recycling capability. The entire living catalyst can be reused for multiple cycles while cell viability is high. When the cells  lose productivity, the valuable synthetic catalyst can be selectively dispalaced, recovered, and re-assembled onto a fresh batch of engineered cells, establishing a truly sustainable and circular process. This work provides a powerful and generalizable blueprint for the seamless integration of biological and chemical catalysis within a single, compartmentalized system. We believe this approach of using tunable surface chemistry to program the assembly of living materials at interfaces will pave the way for a new generation of intelligent, adaptive, and sustainable catalytic systems with significant potential for industrial biotechnology and green chemical manufacturing.

## Methods

### Materials

Commercial chemicals were used without further purification unless otherwise mentioned. The *Escherichia coli* (BL21(DE3)) strain was obtained from Thermo Fisher Scientific. The preparation of cell cultures and the expression of enzymes were described in the supplementary information.

### Synthesis of oil-based catalysts (OBC)

#### ⅰ) Synthesis of oil-based catalysts PFA

First, palm oil (88.5 g, 0.1 mol) was poured into a flask and placed in an oil bath at 100 °C to degas for 1 hour. Subsequently, 2-(methylamino) ethanol (30.4 g, 0.4 mol) and sodium methoxide (0.6 mL, 3 mmol) were added by injection after cooling down to 60 °C. The reaction was sealed overnight. 12 hours later, sodium methoxide (0.6 mL, 3 mmol) was added additionally and the reaction was continued for another 6 hours. The crude product was cooled to room temperature and diluted with dichloromethane (DCM). It was washed with saturated sodium chloride solution until the organic phase was clarified. The organic phase was dried with magnesium sulfate and the solvent was removed by rotary evaporation to obtain a pure PFA.

#### ⅱ) Synthesis of oil-based catalysts OBC

Dissolve PFA (1.5 g, 4.4 mmol), anthraquinone-2-carboxylic acid (1.0 g, 4 mmol), and 4-(dimethylamino)pyridine (DMAP, 0.5 g, 4.4 mmol) in 20 mL of DCM. Allow the dissolution to complete and subsequently dropwise *N*,*N'*-dicyclohexylcarbodiimide (DCC, 0.9 g, 4.4 mmol) was added to the reaction overnight in an ice bath. The product was filtered and the crude product (organic phase) was further purified by column chromatography (ethyl acetate: petroleum ether = 1:3) after removal of the solvent by rotary evaporation. The pure product (OBC) was a pale yellow solid.

### Synthesis of aminated CD (ACD)

First, β-cyclodextrin (β-CD, 25.0 g, 22.0 mmol) was dissolved in 250 mL of a 0.4 M sodium hydroxide solution and cooled to below 5 °C on ice bath. Subsequently, *p*-toluenesulfonyl chloride (17.5 g, 92 mmol) was gradually added to the vigorously stirred solution over a period of 5 min. The reaction mixture was maintained at 5 °C under continuous stirring for 30 min, followed by filtration. The resulting filtrate was neutralized with concentrated hydrochloric acid and further stirred for 1 h. The precipitate obtained was collected by filtration, washed thoroughly with water (three times), and dried overnight at 60 °C, yielding 7.1 g of a white solid, namely OTs-CD.

Second, 4,7,10-trioxa-1,13-tridecanediamine (3.4 g, 15.4 mmol) and OTs-CD (4.0 g, 3.1 mmol) were dissolved in 10 mL of anhydrous *N*-methyl-2-pyrrolidinone (NMP), followed by the addition of potassium iodide (KI, 50 mg, 0.3 mmol). The reaction mixture was stirred under an inert and anhydrous atmosphere at 70 °C overnight. Upon completion, the resulting yellow solution was cooled to room temperature and subsequently diluted with 200 mL of ethanol. The precipitate formed was collected by filtration, washed sequentially with ethanol and diethyl ether, and further purified via column chromatography (acetonitrile: H_2_O: aqueous ammonia = 1:1:0.2). Finally, the purified product was dried under vacuum at 50 °C overnight, yielding 2.6 g of white solid, namely aminated CD (ACD).

### Preparation of conjugates (cell@OBC)

#### ⅰ) Preparation of conjugates (cell@CD)

The freshly collected *E. coli* was dispersed in 40 mL of potassium phosphate buffer (KPI buffer, pH 8.0, 0.1 M) with a final OD_600_ of 2.0. ACD (10 mM), EDC (18 mM), and NHS (14 mM) were added together to the *E. coli* dispersion and the reaction was carried out for 12 hours at 4 °C to obtain the crude product. At the end of the reaction, the excess free ACD and unreacted reactants were removed by washing three times with KPI buffer (pH 8.0, 0.1 M). The product was collected by centrifugation and the final conjugates (cell@CD) were suspended in KPI buffer (pH 8.0, 0.1 M) with a final OD_600_ of 2.0 for further use.

#### ⅱ) Preparation of conjugates (cell@OBC)

The collected cell@CD was dispersed in 40 mL of KPI buffer (pH 8.0, 0.1 M) with a final OD_600_ of 2.0. Subsequently, OBC (10 mM) dissolved in 4 mL of DMSO was added to the above buffer and reacted overnight at 4 °C. At the end of the reaction, excess free OBC was removed by washing 3 times with KPI buffer (pH 8.0, 0.1 M) containing 10% DMSO. The product (cell@OBC) was collected by centrifugation.

### Interfacial catalysis with different *E. coli*

A solution of 0.6 mL of cell@OBC overexpressing different enzymes was mixed with 0.4 mL of organic solution and the emulsion was made by handshaking. Then, 100 mM substrates were added in emulsions to initiate the reaction. At each time interval, a 20 μL mixture was taken and extracted with 180 μL ethyl acetate, followed by drying with anhydrous magnesium sulfate. After centrifugation, 150 μL supernatant was withdrawn for gas chromatography measurements. All the experiments were carried out in triplicate.

### Reporting summary

Further information on research design is available in the Nature Portfolio Reporting Summary linked to this article.

## Supplementary information

Supplementary Information

Description of Additional Supplementary Files

Supplementary Movie 1

Supplementary Movie 2

Reporting Summary

Transparent Peer Review file

## Source data

Source Data

## Data Availability

All data are available in the main text and the supplementary information or available from the authors upon reasonable request. Source data are provided with this paper. Source data is available for Figs. 2, 4, 5, 6 and 7. Source data are provided with this paper.
