# Supplementary material for: Assembly-Line Biosynthesis in Living-Cell Emulsions via Tunable Supramolecular Surface Chemistry
Source: Nat Commun. 2026 Jun 24;17:7948. doi: 10.1038/s41467-026-74245-z (PMC13448702; doi:10.1038/s41467-026-74245-z)
Supplement: Supplementary file 1 [file 41467_2026_74245_MOESM1_ESM.pdf]

Supplementary Information

Assembly-Line Biotransformation in Living-Cell Emulsions via Tunable Supramolecular Surface Chemistry

Xiankun Wu [1,2], Mathias Dimde [3], Henrik Karring [4], Zhongkai Wang [1*], Changzhu Wu [2,5*]

[1] Anhui Provincial Engineering Center for High Performance Biobased Nylons, School of Materials and Chemistry, Anhui Agricultural University, Hefei, Anhui 230036, China

[2] Department of Physics, Chemistry and Pharmacy, University of Southern Denmark, Campusvej 55, 5230 Odense, Denmark

[3] Department of Biology, Chemistry and Pharmacy, Institute of Chemistry and Biochemistry, Research Center of Electron Microscopy (FZEM), Free University of Berlin, Berlin, Germany

[4] Department of Green Technology, University of Southern Denmark, Campusvej 55, 5230 Odense, Denmark

[5] Danish Institute for Advanced Study (DIAS), University of Southern Denmark, Campusvej 55, 5230 Odense, Denmark

* Correspondence:

Email: wu@sdu.dk (C.W.), wangzk6@ahau.edu.cn (Z.W).

Table of Contents

Materials......3

Instruments......3

Synthesis......4

Preparation of conjugates......6

Enzyme expression......6

Expression of *benzaldehyde lyase* (BAL)......6

Expression of *Candida antarctica* Lipase B (Cal B)......7

Expression of green fluorescence protein (GFP)......7

Expression of alcohol dehydrogenase from *Rhodococcus ruber* (RR ADH)......7

Expression of alcohol dehydrogenase from *Bacillus stearothermophilus* (ADH ht)......8

Expression of *amine transaminase* (ATA)......8

Expression of *Lactobacillus kefir* (LK ADH)......8

Viability assay of *E. coli* cells......9

Protectability against external stresses......9

Water contact angle experiments......10

Plating assay of *E. coli* cells......10

Emulsions with different water-to-oil ratios......10

Calculation of the CI value of emulsions......11

Catalytic performance......11

Single step interfacial catalysis with different *E. coli*......11

Multienzyme cascade catalysis......11

Re-culturing and growth curve of *E. coli* cells......13

600 mL-scale interfacial biocatalysis......13

5 L-scale interfacial biocatalysis......13

Recycle experiments......14

Molecular dynamics simulation......14

Supplementary Table......16

Supplementary Figures......19

Supplementary Reference......72

Materials

All the chemicals and reagents were used without further purification unless mentioned. Anthraquinone-2-carboxylic acid, palm oil, 1-(3-dimethylaminopropyl)-3-ethylcarbodiimide (EDC), *N*-hydroxy succinimide (NHS), (*S*)-1-phenylethylamine, sodium pyruvate, isopropyl alcohol, Eosin Y, sodium methoxide, Fluorescein isothiocyanate isomer I (FITC) were purchased from Sigma Aldrich. Sodium chloride, Sodium bicarbonate, *N*-methyl-2-pyrrolidinone, *N*, *N*'dicyclohexylcarbodiimid (DCC), 4,7,10-trioxa-1,13-tridecanediamine, vinyl butyrate, 4-dimethylaminopyridine (DMAP), Dichloromethane (DCM), magnesium sulfate, 4-dimethylaminopyridine and 2-(methylamino)ethanol were purchased from VWR. Propidium iodide, potassium iodide, SYTO 9, β-cyclodextrin (β-CD), p-toluenesulfonyl chloride, and 1-adamantanecarboxylic acid were purchased from Thermo Fisher Scientific. All other reagents were of analytical grade.

Instruments

Fourier-transform infrared spectroscopy (FTIR) was recorded on a Cary 630 FTIR from Agilent Technologies. Gas chromatography (GC) analysis was carried out on a Shimadzu Nexis™ GC-2030 gas chromatography machine, equipped with an AOC-20i/s autoinjector/autosampler. ^{1}H NMR spectra were recorded on a Bruker AVANCE III 400 MHz and Hz equipped with a tunable multinuclear probe and an auto-sampler. Samples were measured at 25 °C using 5-mm NMR tubes.

Thermal gravimetric analysis (TGA) was performed with a PerkinElmer Thermogravimetric Analyzer TGA 4000. The temperature was raised from 25 to 100 °C at a rate of 10 °C/min, kept at 100 °C for 10 min, and then raised to 800 °C at a rate of 10 °C/min. Holding for 5 min at 800 °C, and cooling from 800 °C to 25 °C at 100 °C /min. Zeta potential analysis was performed using a DelsaMax PRO zeta potential dynamic light scattering analyzer (Beckman Coulter). UV-visible spectra were recorded on a Cary 60 UV-Vis (Agilent Technologies) spectrophotometer at room temperature using a 2-mm quartz cuvette. Fluorescence spectra were recorded on a Varian Cary Eclipse Fluorescence Spectrophotometer at room temperature. High-performance liquid chromatography (HPLC) was performed on a Thermo Scientific Vanquish ™ Core system equipped with a normal-phase chromatography kit and a

variable-wavelength detector(VWD). Enantiomeric purity was determined using a Daicel Chiral OJ-H or IB N-5 column (250 ×4.6 mm i.d.) with hexane/isopropanol as the mobile phase. Data were analyzed using Chromeleon™software (Thermo Scientific). Gas chromatography–mass spectrometry (GC-MS) analysis was conducted using a Shimadzu Nexis™ GC-2030AM (Shimadzu, Japan) equipped with a Daicel chiral column OJ-H (250 ×4.6 mm i.d.). Data acquisition and processing were performed with LabSolutionsTM GC-MS software for advanced mass spectrometric analysis.

Surface morphology of cells using cryotransmission electron microscopy (Cryo-TEM). First, perforated carbon film-covered microscopical 200 mesh grids (R1.2/1.3 batch of Quantifoil, MicroTools GmbH, Jena, Germany) were cleaned with chloroform and hydrophilized by 60 s glow discharging at 10 mA in a Safematic CCU-010 device (safematic GmbH, Zizers, Switzerland). Subsequently, 4 µl aliquots of the sample solution were applied to the grids. The samples were vitrified by automatic blotting and plunge freezing with an FEI Vitrobot Mark IV (Thermo Fisher Scientific Inc., Waltham, Massachusetts, USA) using liquid ethane as a cryogen.

The vitrified specimens were transferred to the autoloader of a FEI TALOS ARCTICA electron microscope (Thermo Fisher Scientific Inc., Waltham, Massachusetts, USA). This microscope is equipped with a high-brightness field-emission gun (XFEG) operated at an acceleration voltage of 200 kV. Micrographs were acquired on an FEI Falcon 3 direct electron detector (Thermo Fisher Scientific Inc., Waltham, Massachusetts, USA) using a 100 µm objective aperture.

Fluorescent images of cells were acquired using a Nikon A1 confocal microscope with NIS-Elements AR 5.30 software. Channels were set up in the 407 nm (DAPI) and 561 nm (TRITC) range using a CoolLED “pE-300 white” light source, standard DAPI and TRITC filter cubes and an Andor Zyla monochrome camera. The contrast was adjusted to the desired levels in the ImageJ version. 1.53 v.

Synthesis

Synthesis of oil-based catalysts (OBC)

i) Synthesis of oil-based catalysts PFA

First, palm oil (88.5 g, 0.1mol) was poured into a flask and placed in an oil bath at 100 °C to degas for 1 hour. Subsequently, 2-(methylamino) ethanol (30.4 g, 0.4 mol)

and sodium methoxide (0.6 mL, 3 mmol) were added by injection after cooling down to 60 ℃. The reaction was sealed overnight. 12 hours later, sodium methoxide (0.6 mL, 3 mmol) was added additionally and the reaction was continued for another 6 hours. The crude product was cooled to room temperature and diluted with DCM. It was washed with saturated sodium chloride solution until the organic phase was clarified. The organic phase was dried with magnesium sulfate and the solvent was removed by rotary evaporation to obtain a pure hydroxylated fatty acid (**PFA**)[1].

ii) Synthesis of oil-based catalysts OBC

Dissolve PFA (1.5 g, 4.4 mmol), Anthraquinone-2-carboxylic acid (1.0 g, 4 mmol), and DMAP (0.5 g, 4.4 mmol) in 20 ml of DCM. Allow the dissolution to complete and subsequently, dropwise DCC (0.9 g, 4.4 mmol) was added to the reaction overnight in an ice bath. The product was filtered and the crude product (organic phase) was further purified by column chromatography (ethyl acetate: petroleum ether = 1:3) after removal of the solvent by rotary evaporation. The pure product (OBC) was a pale yellow solid.

Synthesis of Aminated CD (ACD)

First, β-cyclodextrin (β-CD, 25.0 g, 22.0 mmol) was dissolved in 250 mL of a 0.4 M sodium hydroxide solution and cooled to below 5 ℃ on ice bath. Subsequently, p-toluenesulfonyl chloride (17.5 g, 92 mmol) was gradually added to the vigorously stirred solution over a period of 5 min. The reaction mixture was maintained at 5 ℃ under continuous stirring for 30 min, followed by filtration. The resulting filtrate was neutralized with concentrated hydrochloric acid and further stirred for 1 h. The precipitate obtained was collected by filtration, washed thoroughly with water (three times), and dried overnight at 60 ℃, yielding 7.1 g of a white solid, namely **OTs-CD**.

Second, 4,7,10-trioxa-1,13-tridecanediamine (3.4 g, 15.5 mmol) and OTs-CD (4.0 g, 3.1 mmol) were dissolved in 10 mL of anhydrous *N*-methyl-2-pyrrolidinone (NMP), followed by the addition of potassium iodide (KI, 50 mg, 0.3 mmol). The reaction mixture was stirred under an inert and anhydrous atmosphere at 70 ℃ overnight. Upon completion, the resulting yellow solution was cooled to room temperature and subsequently diluted with 200 mL of ethanol. The precipitate formed was collected by filtration, washed sequentially with ethanol and diethyl ether, and further purified via

column chromatography (acetonitrile: H_2O: aqueous ammonia = 1:1:0.2). Finally, the purified product was dried under vacuum at 50 °C overnight, yielding 2.6 g of a white solid (**ACD**).

Preparation of conjugates (cell@OBC)

i) Preparation of conjugates (cell@CD)

The freshly collected *E. coli* was dispersed in 40 mL of KPI buffer (pH 8.0, 0.1 M) with a final OD_{600} of 2.0. ACD (10 mM), EDC (18 mM), and NHS (14 mM) were added together to the *E. coli* dispersion and the reaction was carried out for 12 hours at 4 °C to obtain the crude product. At the end of the reaction, the excess free ACD and unreacted substrate were removed by washing three times with KPI buffer (pH 8.0, 0.1 M). The product was collected by centrifugation and the final conjugates (cell@CD) were suspended in KPI buffer (pH 8.0, 0.1 M) with a final OD_{600} of 2.0 for further use[2].

ii) Preparation of conjugates (cell@OBC)

The collected cell@CD was dispersed in 40 mL of KPI buffer (pH 8.0, 0.1 M) with a final OD_{600} of 2.0. Subsequently, OBC (10 mM) dissolved in 4 mL of DMSO was added to the above buffer and reacted overnight at 4 °C. At the end of the reaction, excess free OBC was removed by washing 3 times with KPI buffer (pH 8.0, 0.1 M) containing 10% DMSO. The product (cell@OBC) was collected by centrifugation[3].

Enzyme expression

Expression of benzaldehyde lyase (BAL)

The BAL enzyme was expressed in a recombinant *E. coli* BL21 (DE3) strain containing the pET28a plasmid. For preculture preparation, the cells were grown overnight at 37 °C in Luria-Bertani (LB) medium supplemented with kanamycin. The resulting preculture was inoculated into fresh medium and cultivated at 20 °C until the optical density at 600 nm (OD_{600}) reached approximately 0.6. Protein expression was then induced by adding isopropyl β-D-1-thiogalactopyranoside (IPTG) to a final concentration of 1 mM. After induction, the culture was further incubated for 16 hours. Finally, the cells were collected by centrifugation at 2,490 x g for 10 min at 4 °C[4].

Expression of Candida antarctica* Lipase B *(Cal B)

Cal B expression was carried out using *E. coli* BL21 (DE3) containing the pET22b_Cal B-His construct, in which the expressed enzyme is targeted to the periplasmic space. For preculture preparation, the strain was cultivated overnight at 37 °C in LB medium supplemented with 100 μg mL^{-1} ampicillin. Subsequently, the preculture was then transferred into an autoinduction medium containing the same concentration of ampicillin and incubated at 20 °C overnight for protein expression[5].

Auto-Induction Medium (2 L): 20 g Tryptone, 10 g Yeast extract, 40 mL 50 xM stock solution, 4 mL 1M $MgSO_4$, 0.4 mL 1000x Trace Metals stock solution, 40 mL 50x 5052 stock solution.

Add sterilized stocks to autoclaved Tryptone + Yeast medium right before use.

50x salt stock solution: 1.25 M Na_2HPO_4X7 H_2O, 1.25 M KH_2PO_4, 2.5 M NH_4Cl, 0.25 M Na_2SO_4.

50X5052 stock solution: 25% Glycerol, 2.5% Glucose, 10% G-Lactose.

1000x stock solution of trace metals: 50 mM $FeCl_3$, 20 mM $CaCl_2$ (x2 H_2O), 10 mM $MnCl_2$ (x4 H_2O).
10 mM $ZnSO_4$ (x7 H_2O), 2 mM $CoCl_2$ (x6 H_2O), 2 mM $CuCl_2$, 2 mM $NiCl_2$, 2 mM Na_2MoO_4, 2 mM Ma_2SeO_3, 2 mM H_3BO_3.

Expression of green fluorescence protein (GFP)

GPF in *E. coli* cells was expressed using the strain *E. coli* BL21 (DE3) containing the plasmid pET28b-GFP-His. According to previous research[2], the preculture was prepared with LB medium containing 50 μg mL^{-1} kanamycin and the recombinant strain *E. coli* was grown at 37 °C while shaking at 220 rpm overnight, and then the preculture was transferred into the main culture with 50 μg mL^{-1} kanamycin. IPTG (0.5 mM) was added to induce GFP expression at 20 °C overnight when OD_{600} reached 0.8-1.0 for the main culture. The bacteria pellets were obtained by centrifugation (4 ℃, 2,490 x g, 10 min).

Expression of alcohol dehydrogenase from Rhodococcus ruber (RR ADH)

First, a preculture was prepared by incubating *E. coli* BL21 containing pET22b+-RR ADH plasmid in LB medium with 100 µg mL^{-1} ampicillin overnight at 37 °C. Second, a main culture was prepared in terrific broth (TB) medium with 100 µg mL^{-1} ampicillin at 37 °C until reaching OD_{600} = 0.6, followed by adding 1 mM IPTG and 0.43 mM $ZnSO_4$ to induce the RR ADH expression. After further incubation overnight at 20 °C, cells were collected by centrifugation (4 °C, 2,490 x g, 10 min).

Expression of alcohol dehydrogenase from Bacillus stearothermophilus (ADH ht)

For expressing enzyme ADH-ht, in a typical procedure, the *E. coli* strain BL21 containing the plasmid pET21a+-ADH-ht was incubated overnight at 37 °C as the preculture. The preculture was then transferred to the main culture and incubated at 37 °C until reaching OD_{600} 0.7, whereby a final concentration of 1 mM IPTG was added to induce the expression. The cultures were shaken overnight at 20 °C, 120 rpm. After that, the bacteria pellets were obtained by centrifugation (4 °C, 2,490 x g, 10 min).

Expression of amine transaminase (ATA)

A preculture was first established by growing *E. coli* BL21 containing the pET22b-ATA plasmid overnight at 37 °C in TB medium supplemented with 100 µg mL^{-1} ampicillin. This culture was subsequently used to inoculate fresh TB medium containing the same antibiotic, and cells were cultivated at 37 °C until the OD_{600} reached approximately 0.6. At this point, the ATA expression was induced by the addition of IPTG to a final concentration of 1 mM. Following induction, the culture was shifted to 20 °C and incubated overnight. Cells were then collected by centrifugation at 2,490 x g for 10 min at 4 °C.

Expression of* alcohol dehydrogenase from *Lactobacillus kefir (LK ADH)

LK ADH from *Lactobacillus kefir*, subcloned into the pET-22b vector, was expressed in *E. coli* BL21 (DE3). For preculture, the strain was grown overnight at 37 °C and 220 rpm in TB medium supplemented with 100 µg mL^{-1} ampicillin. The preculture

was subsequently used to inoculate fresh medium and cultivated at 37 °C until the OD_{600} reached approximately 0.6. Protein expression was then induced by the addition of IPTG (1 mM) together with $MgCl_2$ (1 mM), followed by incubation at 20 °C overnight. The cells expressing LK ADH were obtained by centrifugation at 4 °C and 2,490 x g for 10 min.

Viability assay of *E. coli* cells

Cells (native cells, conjugate cells and conjugate cells after 5 times recycling) were respectively dispersed in a KPI buffer (pH 8, 0.1 M). Then, the cells were incubated with SYTO 9 for 20 min, followed by incubation with propidium iodide for another 20 min and washed three times with KPI buffer (pH 8, 0.1 M). After that, fluorescent microscopy was used to measure cell viability.

Protectability against external stresses

i) Interfacial stress

Equal amounts of native and conjugate cells were placed into 5 mL of KPI buffer (pH 8, 0.1 M), respectively. Subsequently, 5 mL of toluene or other organic solvent was added to the cell suspension and stirred slowly for 1 h at room temperature to form sufficient interfacial contact between the two phases. Stirring was stopped at intervals and 100 μL of the water phase was taken for live-dead assay of the cells after the interface was stratified. Meanwhile, another 100 μL of the water phase was taken for the enzyme activity assay of the cells.

ii) Thermal treatment

Equal amounts of native and conjugate cells were taken and placed in three 15 mL centrifuge tubes, followed by the addition of 10 mL of KPI buffer (pH 8, 0.1 M). The above three centrifuge tubes were placed in a water bath at 37 °C, 40 °C and 45 °C respectively to heat the cell suspension. 100 μL of the water phase was taken out at each interval for the live/dead assay. Meanwhile, another 100 μL of the water phase was taken for the enzymatic activity assay.

iii) UV treatment

Equal amounts of native cells and cell@OBC were suspended in 10 mL KPi buffer (pH 8, 0.1 M) and exposed to UV light for 2 hours. At each time interval, 1 mL of suspension was taken out and 100 mM benzaldehyde was added as substrate. After 10 minutes of reaction, 20 μL of the reaction mixture was taken, extracted with 180 μL ethyl acetate, and dried with anhydrous magnesium sulfate. After centrifugation, 150 μL of the supernatant was withdrawn for GC measurements. The experiment was carried out in triplicate.

Water contact angle experiments

The cells to be measured were lyophilized and transferred to a press. Subsequently, sheets of cells were placed on slides to measure the water contact angle. A drop of water was dropped on the cell sheet and the images were captured with Point Grey software, which calculated the angle using ImageJ software.

Plating assay of *E. coli* cells

Cells (native cells, conjugate cells and conjugate cells after 5 times recycling) were respectively suspended in 1 mL KPI buffer (pH 8, 0.1 M) to reach a final OD_{600} of 0.1. 20 μL of cell suspension was taken and put on an LB agar plate. The plates were further incubated at 37 °C for 48 h.

Emulsions with different water-to-oil ratios

To optimize interfacial biocatalysis, we prepared emulsions with different water-to-oil ratios. Typically, 2 mL conjugate cells were first taken from the stock solution, collected by centrifugation, and then used as emulsifiers. By adding different amounts of water and organic solvents to a total volume of 1 mL, emulsions were obtained with gentle shaking by hand. These different emulsions were then applied to catalysis reactions. The reactions were initiated upon providing a final concentration of 100 mM substrates. At each time interval, 20 μL emulsion was withdrawn and extracted with 180 μL ethyl acetate. The ethyl acetate phase was dried with anhydrous magnesium sulfate. After centrifugation, 150 μL supernatant was subjected to gas chromatography (GC) analysis. The experiment was repeated in triplicate.

Calculation of the CI value of emulsions

The prepared emulsion was transferred into a vial and stored statically at 25 °C. The creaming index (CI) was calculated as follows:

$$CI = \frac{H_c}{H_t} \times 100\%$$

Where, H_c represented the height of the emulsified layer; H_t represented the total height of the emulsion.

Catalytic performance

Single step interfacial catalysis with different E. coli

First, a solution of 0.6 mL of cell@OBC overexpressing different enzymes was mixed with 0.4 mL of organic solution and the emulsion was made by handshaking. Then, 100 mM substrates was added to the emulsions to initiate the reaction. At each time interval, a 20 μL mixture was taken and extracted with 180 μL ethyl acetate, followed by drying with anhydrous magnesium sulfate. After centrifugation, 150 μL supernatant was withdrawn for GC measurements. The experiment was carried out in triplicate.

Multienzyme cascade catalysis

i) Cascade reaction with ATA and RR ADH

Typically, equal amounts of cell@OBC overexpressing ATA and RR ADH were dispersed in 0.6 mL of KPI buffer (pH 8, 0.1 M), and a Pickering emulsion system was formed by hand shaking after adding 0.4 mL of toluene. Then 100 mM of (*S*)-1-phenylethylamine, sodium pyruvate (250 mM), isopropyl alcohol (100 μL) and PLP (25 μL) were added to initiate the reaction. At each time interval, a 20 μL mixture was taken and extracted with 180 μL ethyl acetate, followed by drying with anhydrous magnesium sulfate. After centrifugation, 150 μL supernatant was withdrawn for GC measurements. The experiment was carried out in triplicate.

ii) Cascade reaction with ADH ht and BAL

Equal amounts of cell@OBC overexpressing ADH ht and BAL were dispersed in 0.6 mL of KPI buffer (pH 8, 0.1 M), and a Pickering emulsion system was formed by hand shaking after adding 0.4 mL of toluene. Then 100 mM of benzyl alcohol, 250

mM 1-octanal and 1 mM NAD^+ were added to initiate the reaction. At each time interval, a 20 μL mixture was taken and extracted with 180 μL ethyl acetate, followed by drying with anhydrous magnesium sulfate. After centrifugation, 150 μL supernatant was withdrawn for GC measurements. The experiment was carried out in triplicate.

iii) Cascade reaction with Cal B and OBC

The cell@OBC overexpressing Cal B was dispersed in 0.6 mL of KPI buffer (pH 8, 0.1 M), and a Pickering emulsion system was formed by hand shaking after adding 0.4 mL of toluene. The emulsion was poured into a transparent glass vial, 100 mM of benzyl acetate was added, and the cascade reaction was initiated under a UV lamp at 365 nm. At each time interval, a 20 μL mixture was taken and extracted with 180 μL ethyl acetate, followed by drying with anhydrous magnesium sulfate. After centrifugation, 150 μL supernatant was withdrawn for GC measurements. The experiment was carried out in triplicate.

IV) Cascade reaction with Cal B, OBC and BAL

Equal amounts of cell@OBC overexpressing Cal B and BAL were dispersed in 0.6 mL of KPI buffer (pH 8, 0.1 M), 0.4 mL of toluene was added, and the Pickering emulsion system was formed by hand shaking. The emulsion was poured into a transparent glass vial, 100 mM benzyl acetate was added, and the cascade reaction was initiated under UV lamp (365 nm). At each time interval, a 20 μL mixture was taken and extracted with 180 μL ethyl acetate, followed by drying with anhydrous magnesium sulfate. After centrifugation, 150 μL supernatant was withdrawn for GC measurements. The experiment was carried out in triplicate.

V) Cascade reaction with ATA, LK ADH and Cal B

Equal amounts of cell@OBC overexpressing ATA and LK ADH were dispersed in 0.6 mL of KPI buffer (pH 8, 0.1 M), and the first Pickering emulsion system was formed by hand shaking after adding 0.4 mL of toluene. Then, (*S*)-1-phenylethylamine (100 mM), sodium pyruvate (250 mM), isopropyl alcohol (100 μL), and PLP (25 μL) were added to the emulsion for the reaction. Upon completion of the reaction, the liquid phase was collected by centrifugation. The organic phase was extracted three times

with a large amount (~ 6 mL) of ethyl acetate and the organic phase was collected, and the solvent was subsequently removed by rotary evaporation to obtain the crude product of (*R*)-1-phenylethanol.

The cell@OBC overexpressing Cal B was dispersed in 0.6 mL of KPI buffer (pH 8, 0.1 M), and the second Pickering emulsion system was formed by hand shaking after adding 0.4 mL of toluene. The emulsion was immediately poured into a round-bottomed flask containing the (*R*)-1-phenylethanol crude product and the reaction was continued overnight with the addition of vinyl butyrate (250 mM). A 20 μL mixture was taken and extracted with 180 μL hexane, followed by drying with anhydrous magnesium sulfate. After centrifugation, 150 μL supernatant was withdrawn for GC measurements. The experiment was carried out in triplicate.

The procedure for all biphasic controls is consistent with the Pickering emulsion system, but hand shaking is not required to maintain the biphasic system.

Re-culturing and growth curve of *E. coli* cells

Cells after recycling were suspended in 1 mL LB medium containing 100 μg mL^{-1} kanamycin to reach a final OD_{600} of 0.1 and incubated at 37 °C. Afterwards, UV-visible spectroscopy was then used to measure OD_{600} at different time intervals. The experiments were performed in triplicate[6].

600 mL-scale interfacial biocatalysis

The 600 mL reaction experiment was carried out under the same conditions as the small-scale reaction. The emulsion was first formed by hand shaking, followed by the addition of benzaldehyde to initiate the reaction. After 6 hours, the organic phase was collected by centrifugation and separation. The solvent was removed by rotary evaporation to obtain the crude product, and the unreacted benzaldehyde was subsequently removed using flash column chromatography (petroleum ether-EtOAc 5:1), ultimately obtaining around 1 g of pure benzoin[7].

5 L-scale interfacial biocatalysis

A 5 L reaction experiment was conducted under the same conditions as the 600 ml reaction, but with a longer handshaking time, followed by the addition of benzaldehyde to initiate the reaction. After 6 hours, the organic phase was collected

by centrifugation and separation. The solvent was removed by rotary evaporation to obtain the crude product, and the unreacted benzaldehyde was subsequently removed using flash column chromatography (petroleum ether-EtOAc 5:1), ultimately obtaining around 7.5 g of pure benzoin.

Recycle experiments

i) Recycling of conjugate cells (cell@OBC)

The conjugate cells are recovered by simple centrifugation. Specifically, the emulsion after completion of the catalytic reaction was centrifuged (2,490 x g, 5 min). After removing the supernatant, the solid phase was washed three times with KPI buffer (pH 8, 0.1 M) containing 5% DMSO to completely remove the substrate and products from the last step. The washed suspension was centrifuged again to obtain the recycled conjugate cell (cell@OBC).

ii) Recycling of oil-based catalyst (OBC)

To obtain more OBC, the recycling is conducted in a 1 L emulsion system at high concentration. First, the recycled cell@OBC was obtained by the steps of i). Subsequently, the cell@OBC was suspended in DMSO with an excess of 1-adamantanecarboxylic acid. The suspension was shaken for 6 h and centrifuged to collect the organic phase. This step was repeated one more time to displace more photocatalysts. The organic phase was collected and then the solvent was removed by rotary evaporation and vacuum chamber. The crude product obtained was washed with water (alkaline) at least three times to remove the 1-adamantanecarboxylic acid. The oil-based catalyst (OBC: light yellow solid) was obtained after the removal of residual water.

Molecular dynamics simulation

Quantum chemistry calculations were first performed to optimize molecular geometries of molecules using the Gaussian 16 package at B3LYP/6-311+G (d) level of theory. The bonded and non-bonded interactions of atoms were described with the OPLS-AA force field, and the charge used is RESP. In the system, 1 β-CD, 20 Adamantane and 20 oil-based catalysts (OBC) were inserted into a simulation box, which is x = 10.0 nm, y = 10.0 nm, and z =10.0 nm, respectively. The steepest descent

method was applied to minimize the initial energy for each system with a force tolerance of 1 kJ/(mol^{-1} nm^{-1}) and a maximum step size of 0.001 ps before MD calculations.

The MD simulations were performed in the GROMACS 2021 software package. In all three directions, periodic boundary conditions were imposed. The Leapfrog algorithm was used to integrate the Newtonian equation of motion. All MD simulations were processed in an NVT ensemble and the simulation time was 20 ns and the temperature was maintained by the V-rescale thermostat at 298.15 K. The Particle-Mesh-Ewald (PME) with a fourth-order interpolation was used to evaluate the electrostatic interactions and a cutoff of 2 nm was employed to calculate the short-range van der Waals interactions.

Supplementary Table

Supplementary Table 1. Effect of different coupling agent concentrations on the retaining activity of the enzyme in cell@OBC.

Items	Product (mM)	Retaining activity (%)
Native cells	24.62	100
EDC (10 mM) +NHS (8 mM)	24.39	99.08
EDC (14 mM) +NHS (10 mM)	24.28	98.62
EDC (18 mM) +NHS (14 mM)	24.10	97.88
EDC (25 mM) +NHS (20 mM)	22.68	92.12
EDC (40 mM) +NHS (30 mM)	21.77	88.42

Supplementary Table 2. Single step reactions for the production of ketone using enzyme ATA.

**Entry**	**Substrate**	**Product**	***Conv.* (%)**	***ee.* (%)**
1	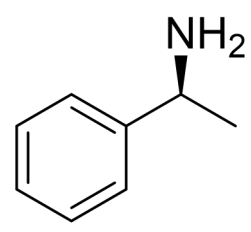	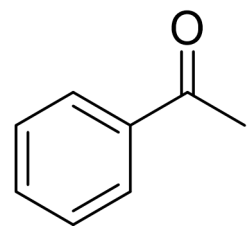	77	-
2	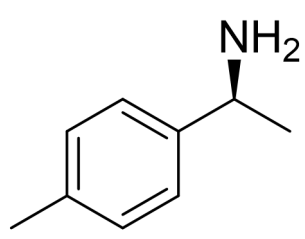	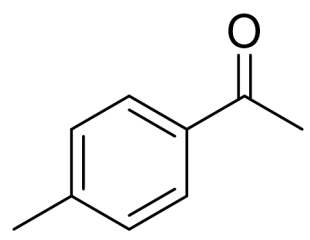	93[a]	-
3	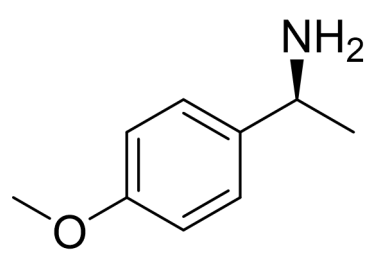	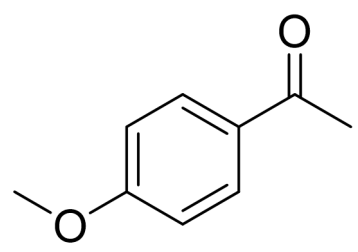	61[a]	-
4	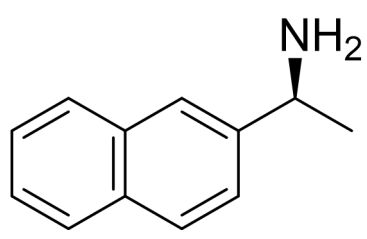	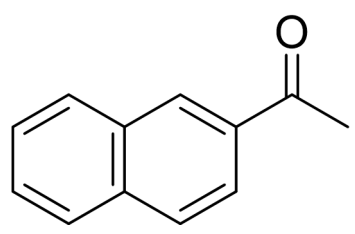	97[a]	-
5	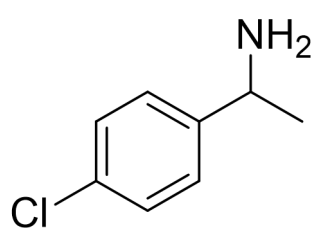	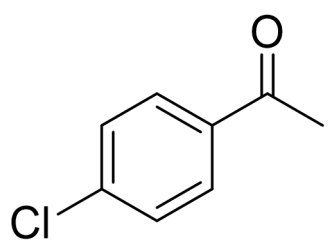	28[a]	-

[a]Conversion determined by ^{1}H NMR. The conversion of acetophenone was determined by GC.

Supplementary Table 3. Cascade reactions for the production of secondary alcohols using enzyme ATA and RR ADH.

**Entry**	**Substrate**	**Product**	***Conv.* (%)**	***ee.* (%)[b]**
1	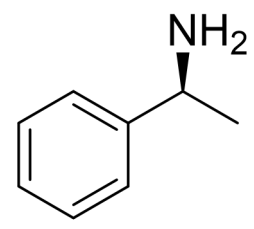	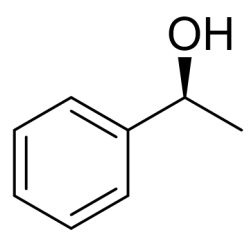	19[a]	99
2	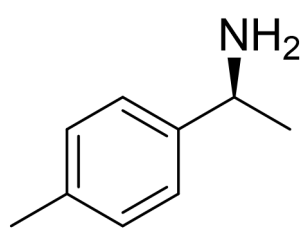	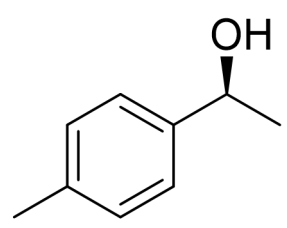	71[a]	81
3	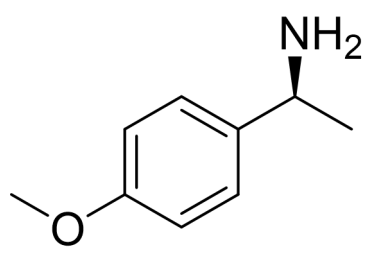	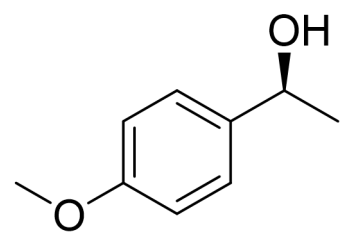	93[a]	99
4	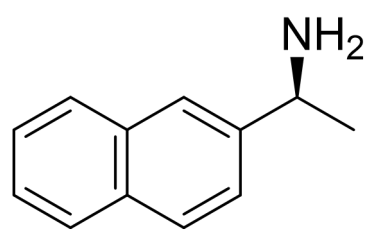	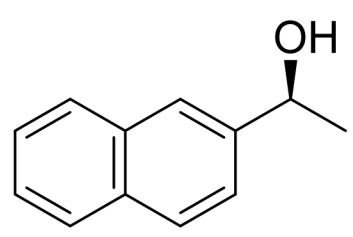	30[a]	99
5	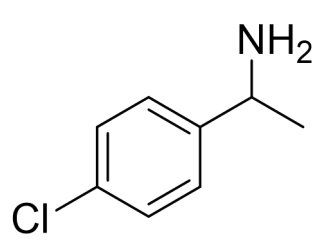	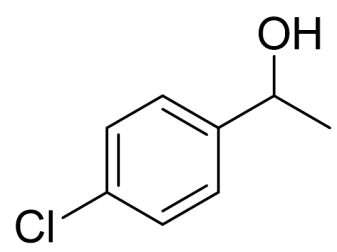	5[a]	-

[a]Conversion determined by ^{1}H NMR. The conversion of (*S*)-1-phenylethanol was determined by GC; [b]*ee* values were determined by chiral HPLC.

Supplementary Figures

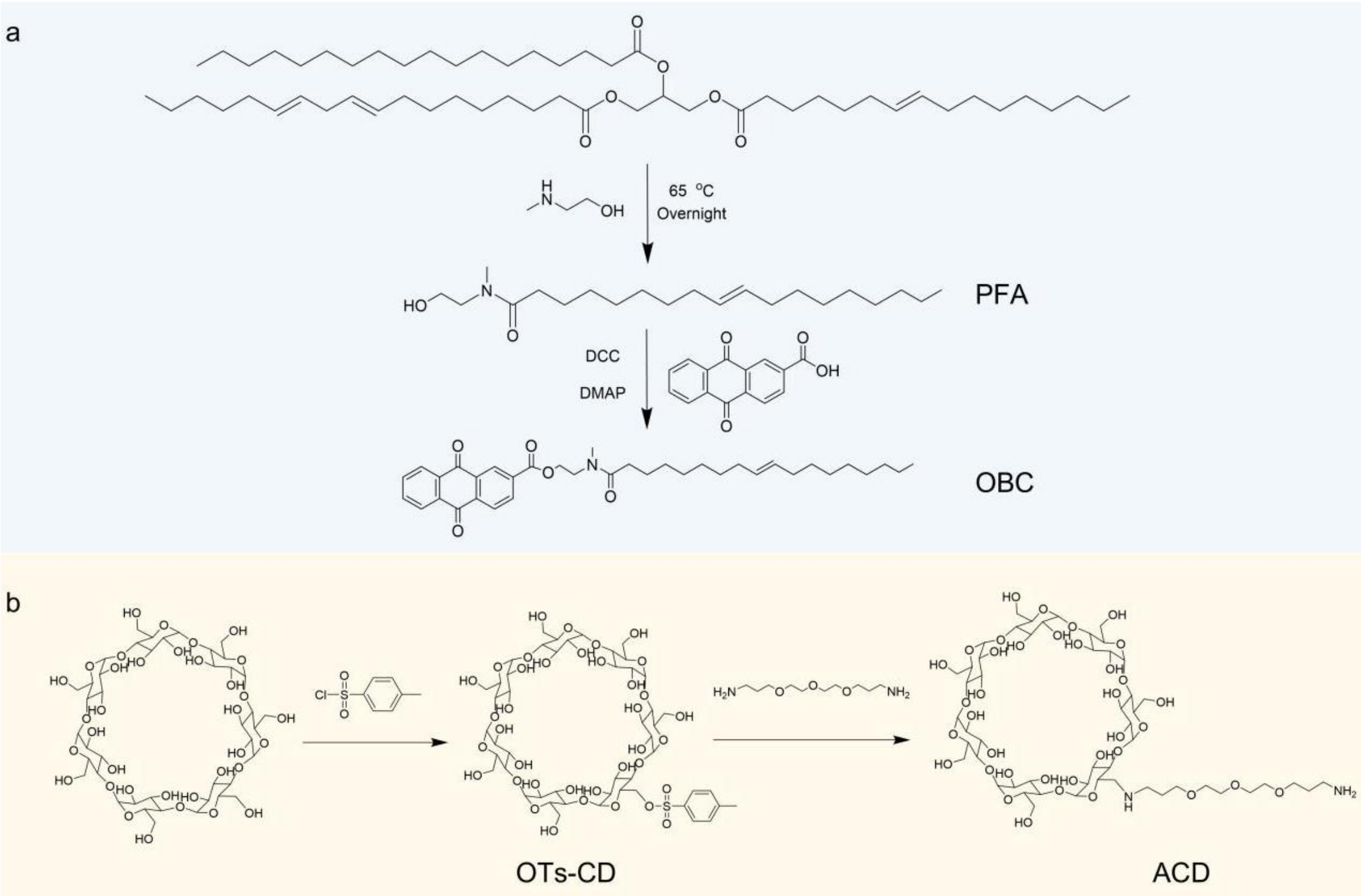

Supplementary Figure 1. (a) Synthesis route of PFA and OBC. (b) Synthesis route of OTs-CD and ACD.

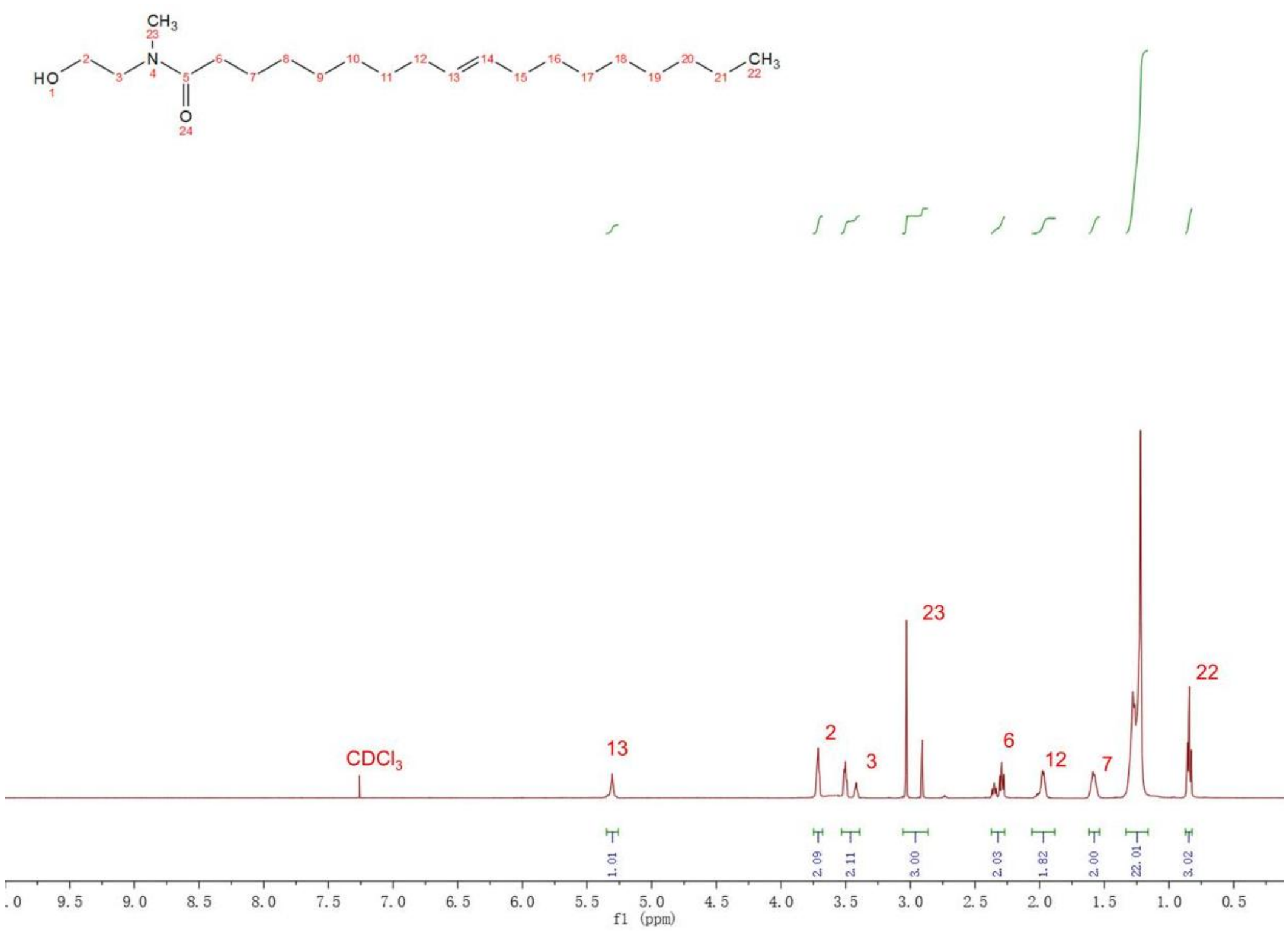

Supplementary Figure 2. ^{1}H NMR spectrum of PFA.

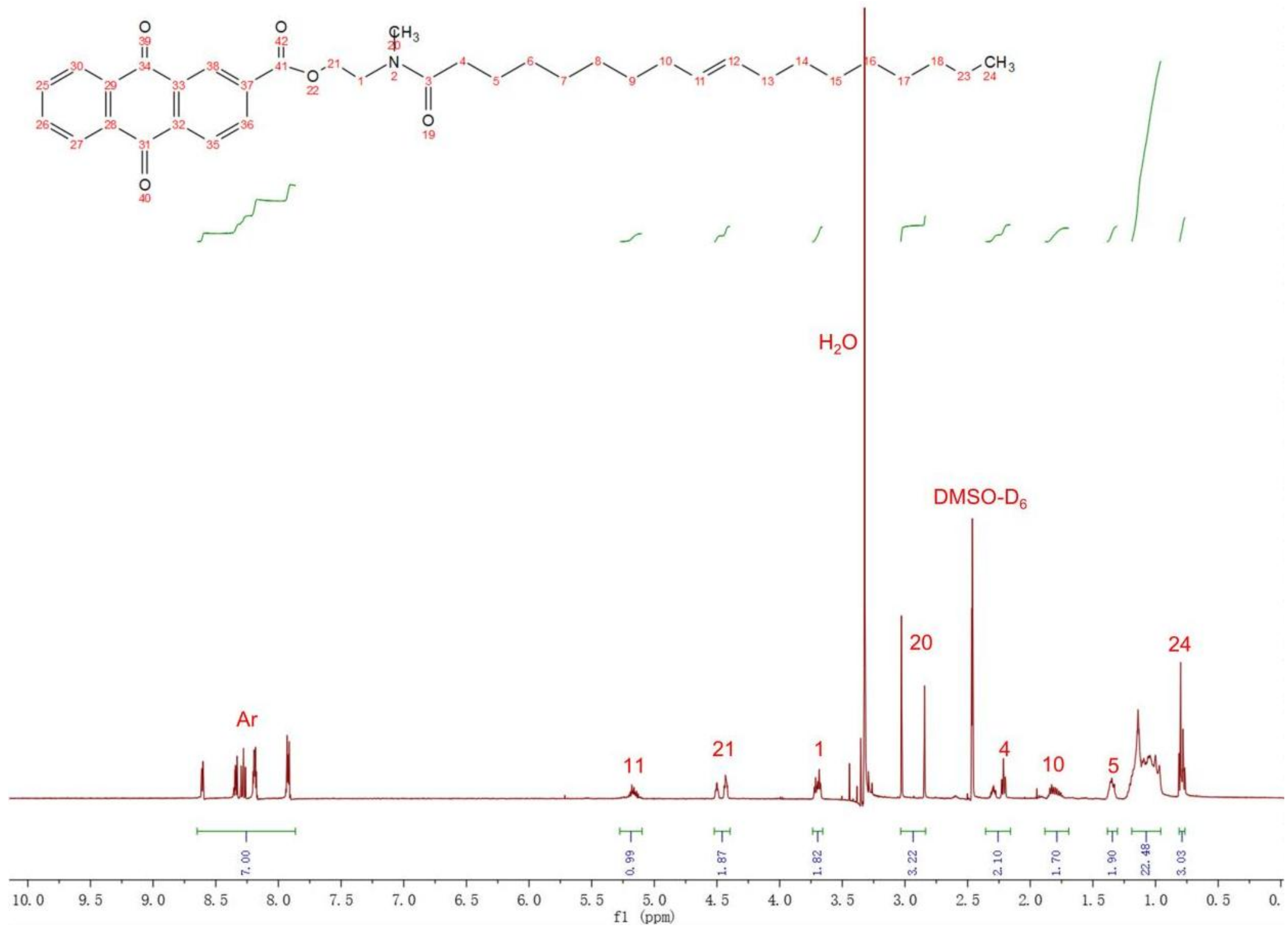

Supplementary Figure 3. ^{1}H NMR spectrum of OBC.

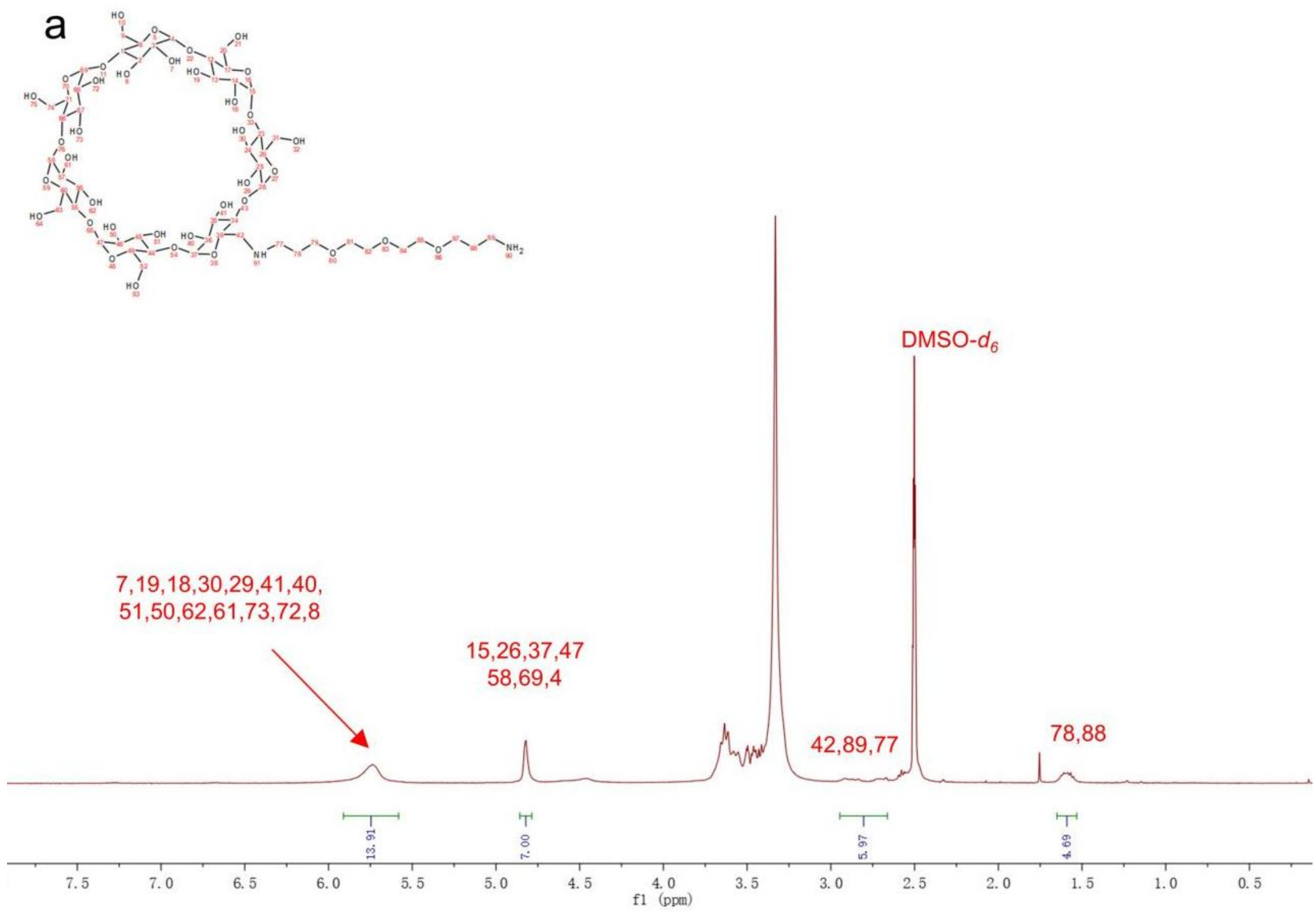

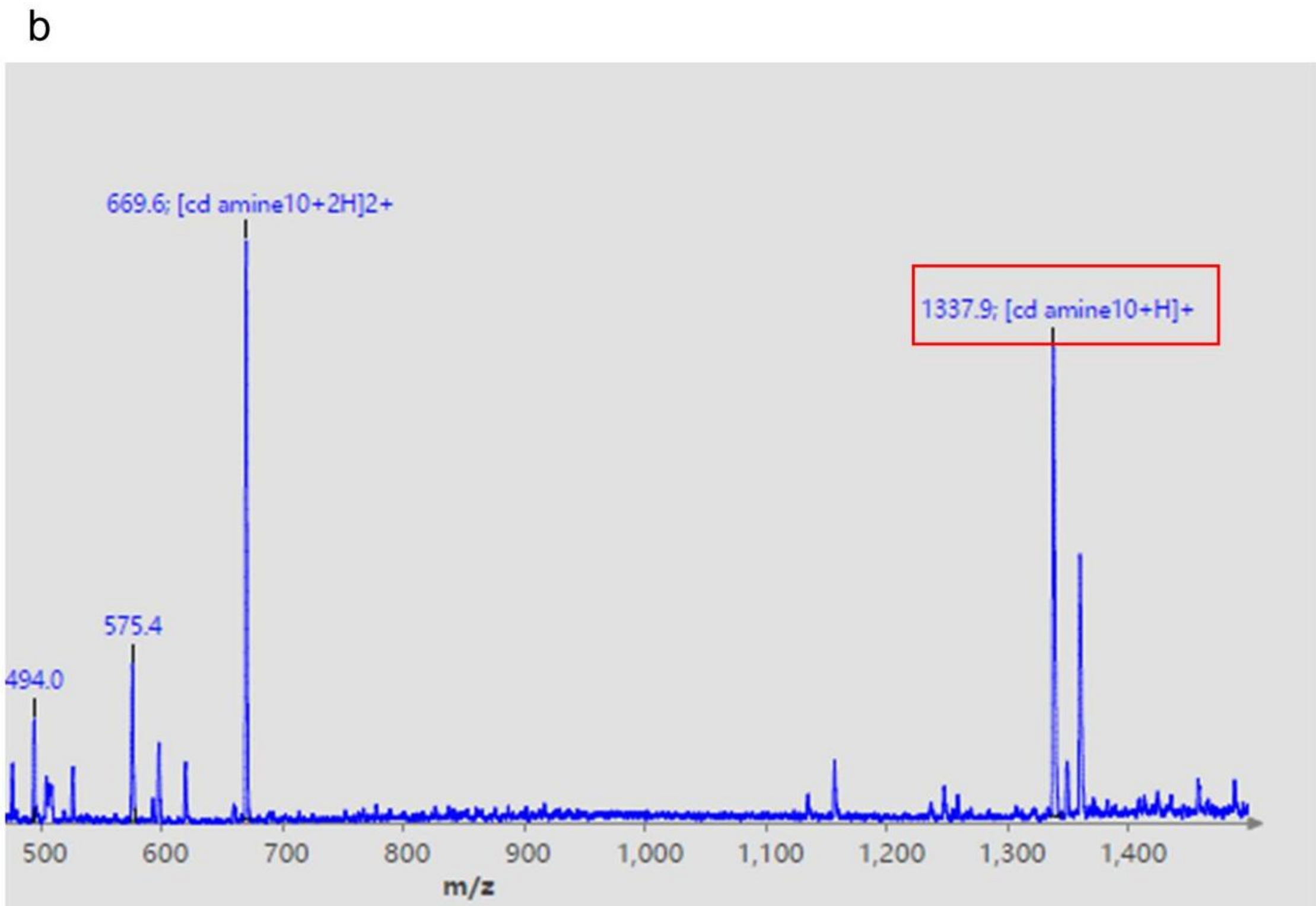

Supplementary Figure 4. (a) ^{1}H NMR spectrum of ACD. (b) TLC-MS spectrum of ACD.

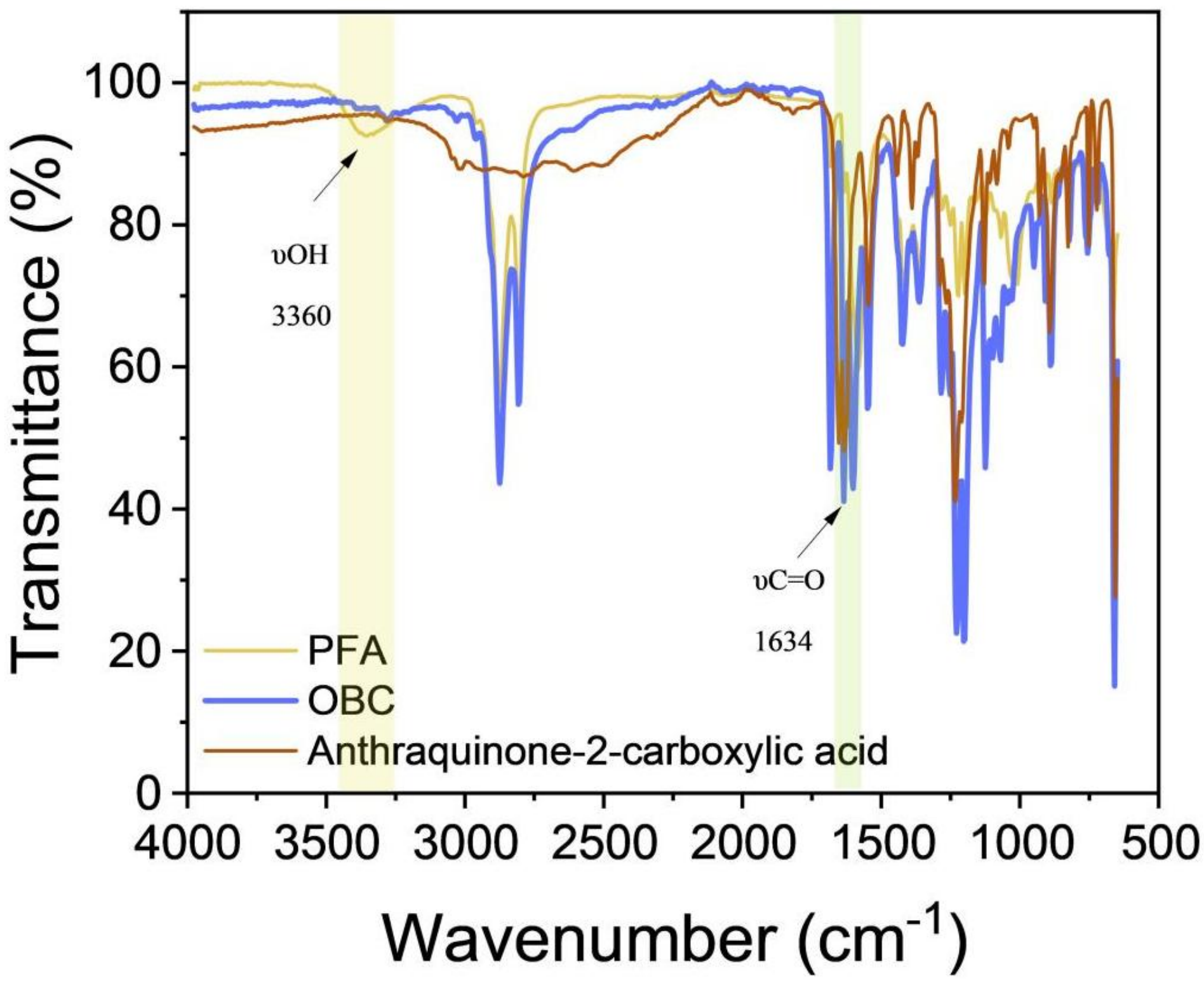

Supplementary Figure 5. FT-IR spectra of PFA, oil based catalyst monomer (OBC) and the Anthraquinone-2-carboxylic acid.

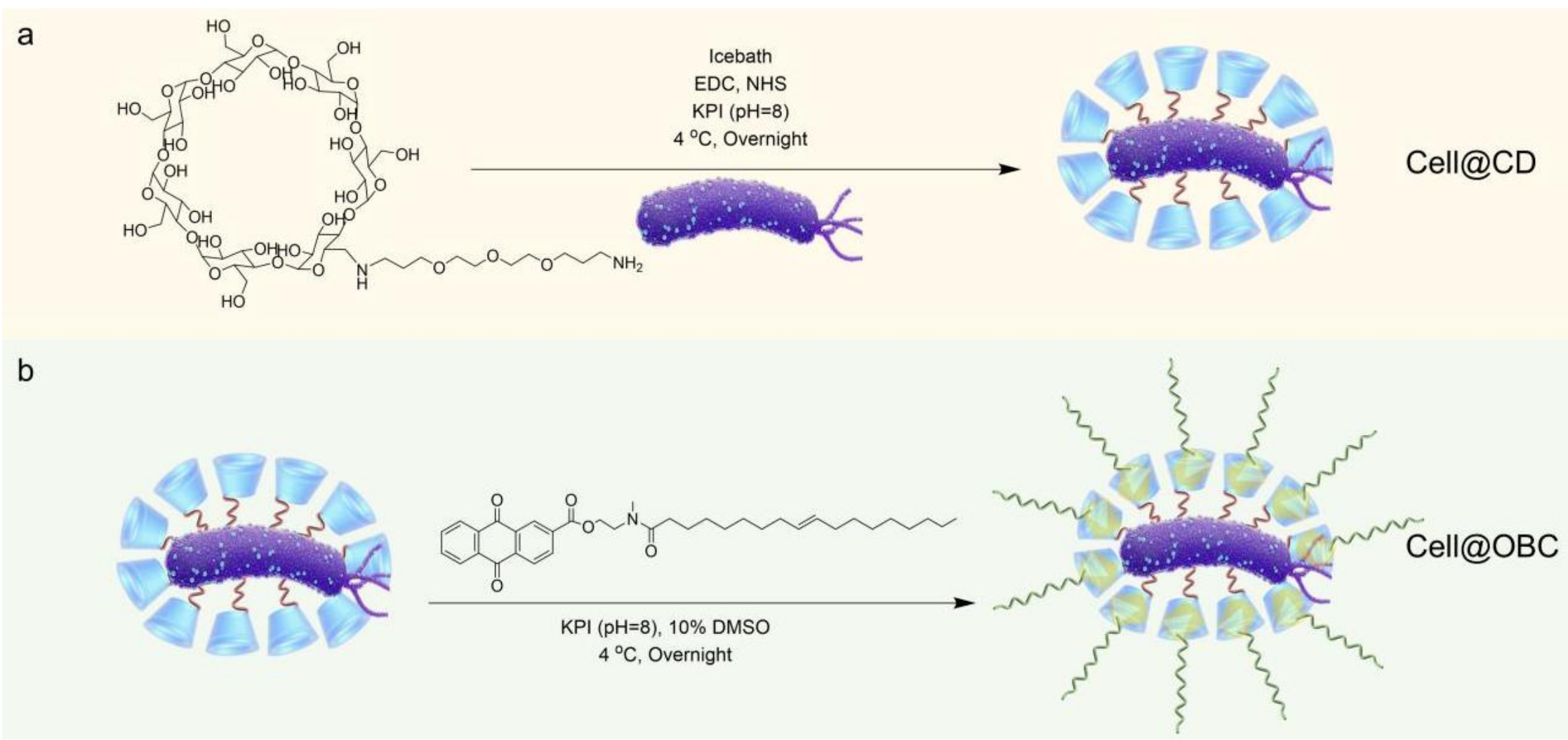

Supplementary Figure 6. (a) Preparation of cell@CD conjugates. (b) Preparation of cell@OBC conjugates.

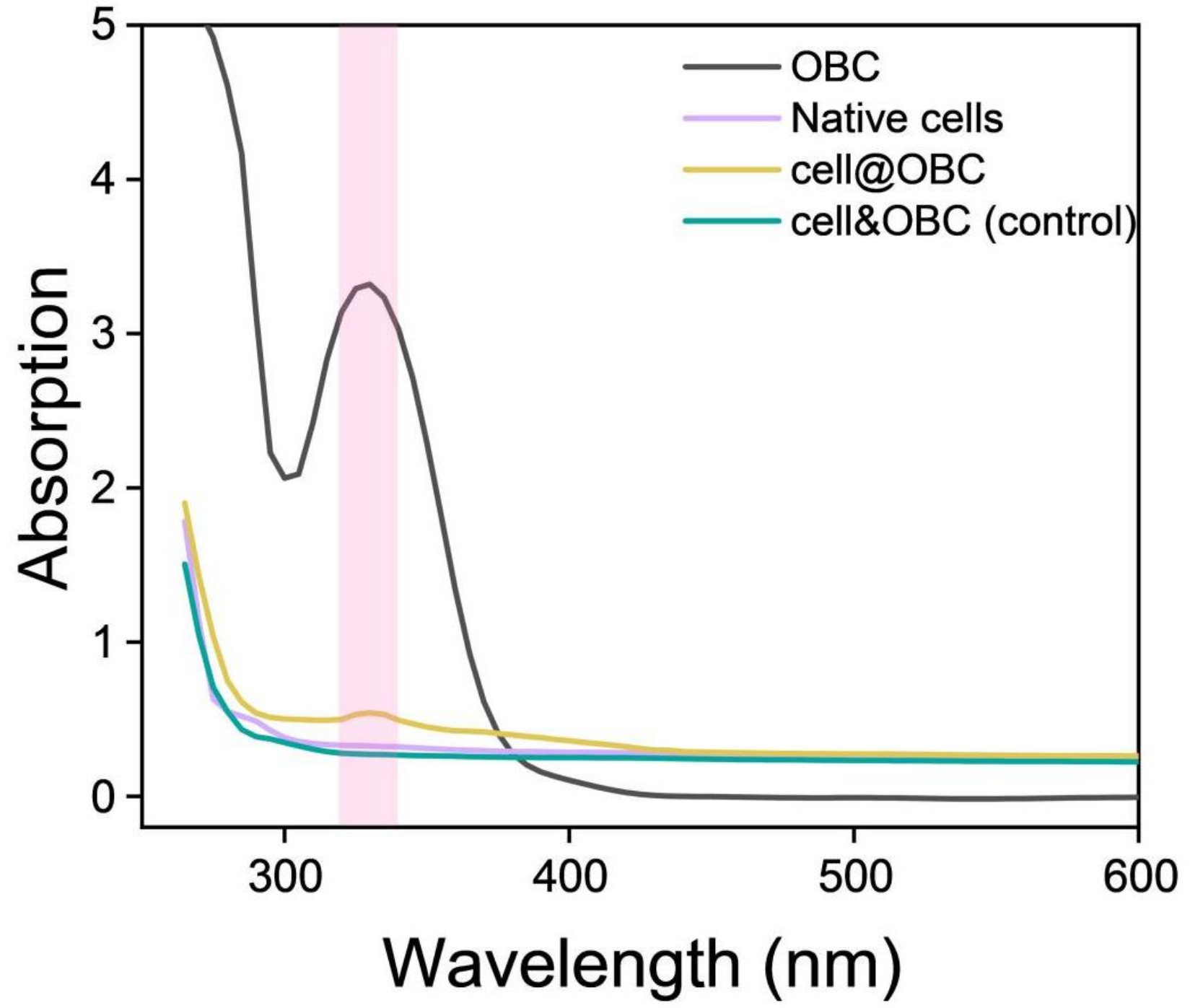

Supplementary Figure 7. UV-vis spectra of oil based catalyst monomer (OBC), native cell and the cell@OBC.

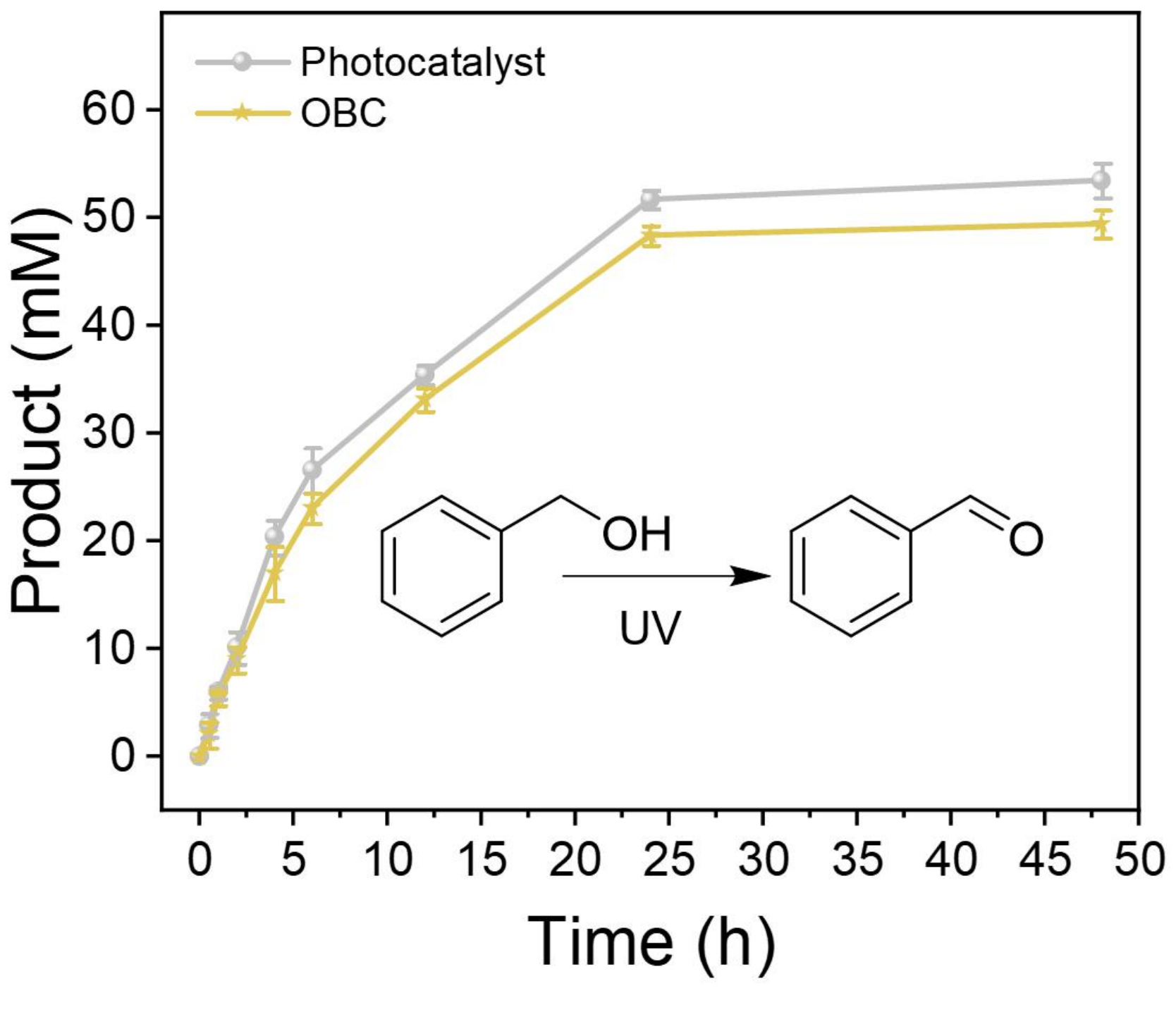

Supplementary Figure 8. The anthraquinone-2-carboxylic acid and oil-based catalyst (OBC) were used for the same catalytic reactions respectively, and there was almost no difference between them. The error bars represent the standard deviations of three parallel measurements, n=3.

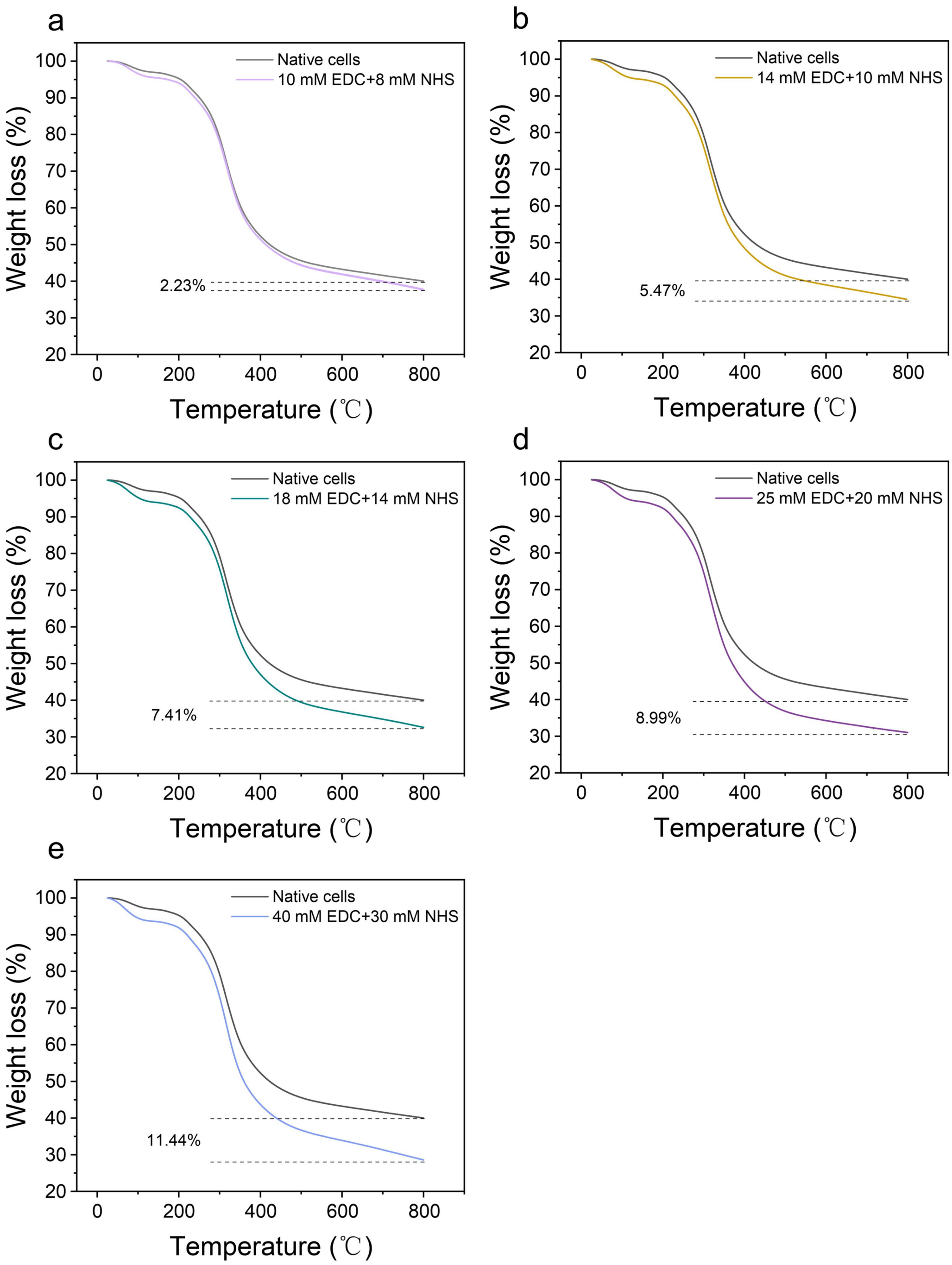

Supplementary Figure 9. TGA curves of (a) native cells and cell@OBC modified with different amounts of coupling agent (b-e).

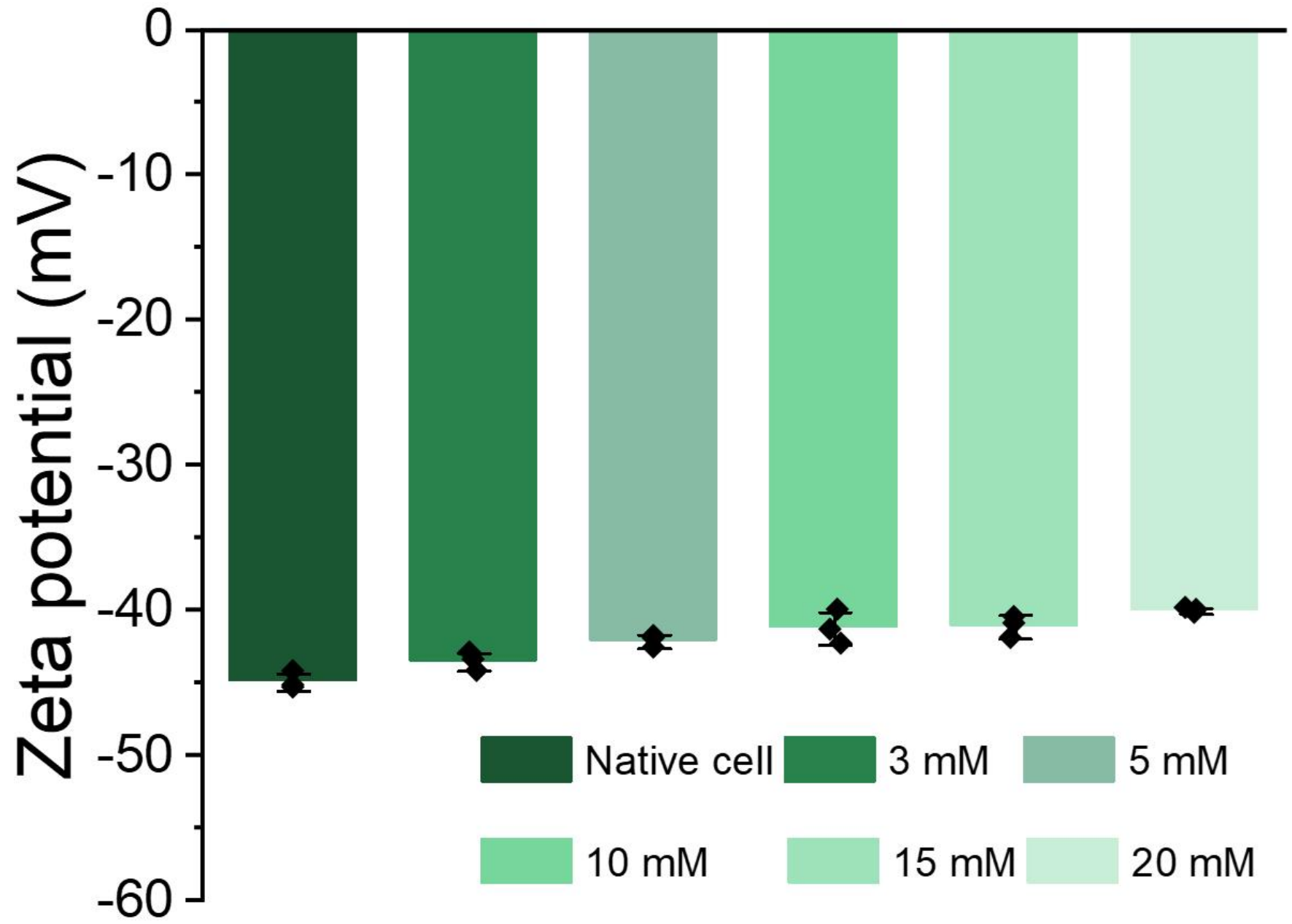

Supplementary Figure 10. Zeta potential of native cells and native cells mixed with OBC solutions at different concentrations. Data are presented as mean values +/- SD based on n=3 independent replicates with the error bars representing the SD.

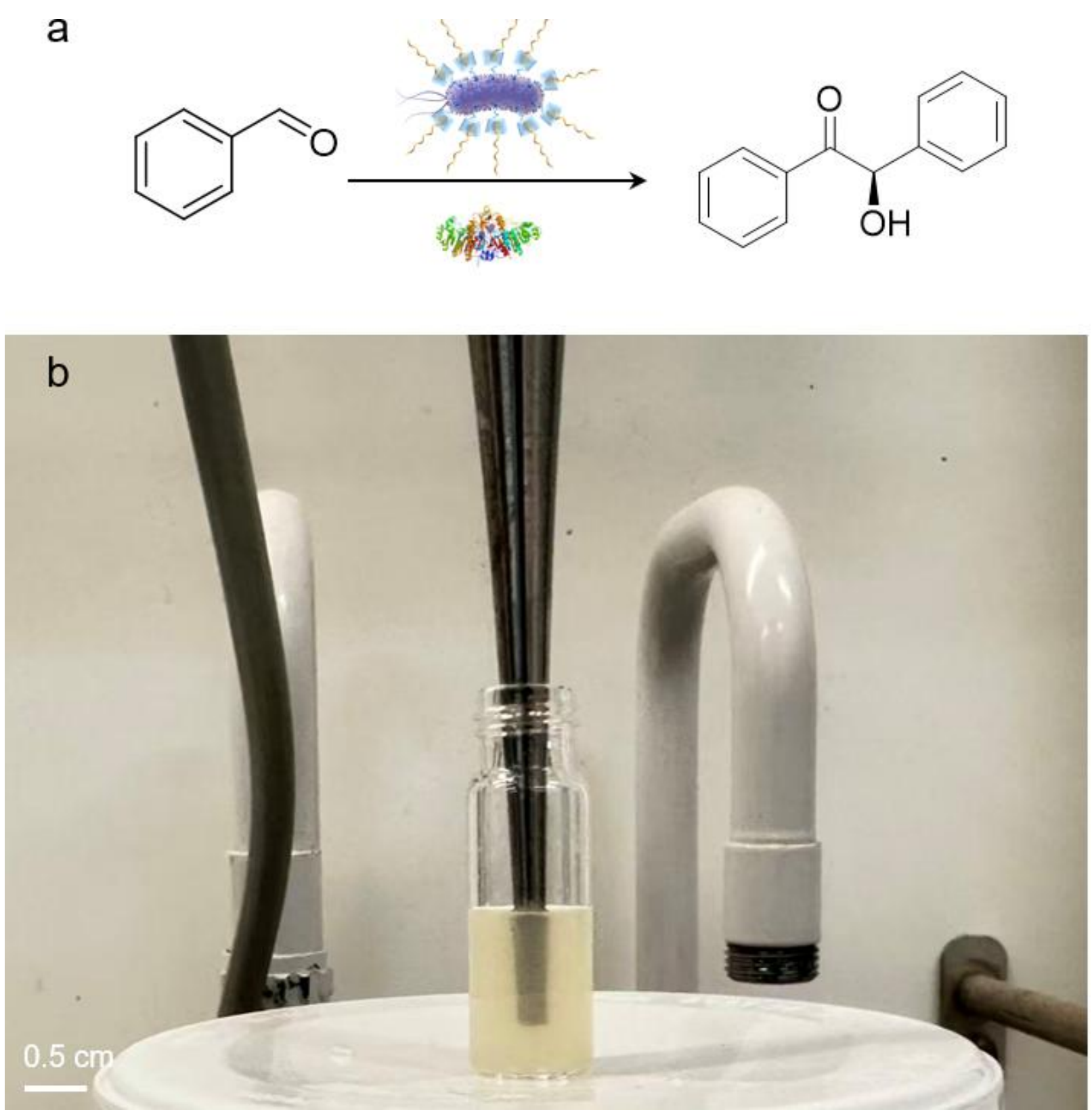

Supplementary Figure 11. (a) The catalytic activity is assessed through the BAL-induced benzoin condensation reaction. (b) Enzyme lysed is achieved by sonication.

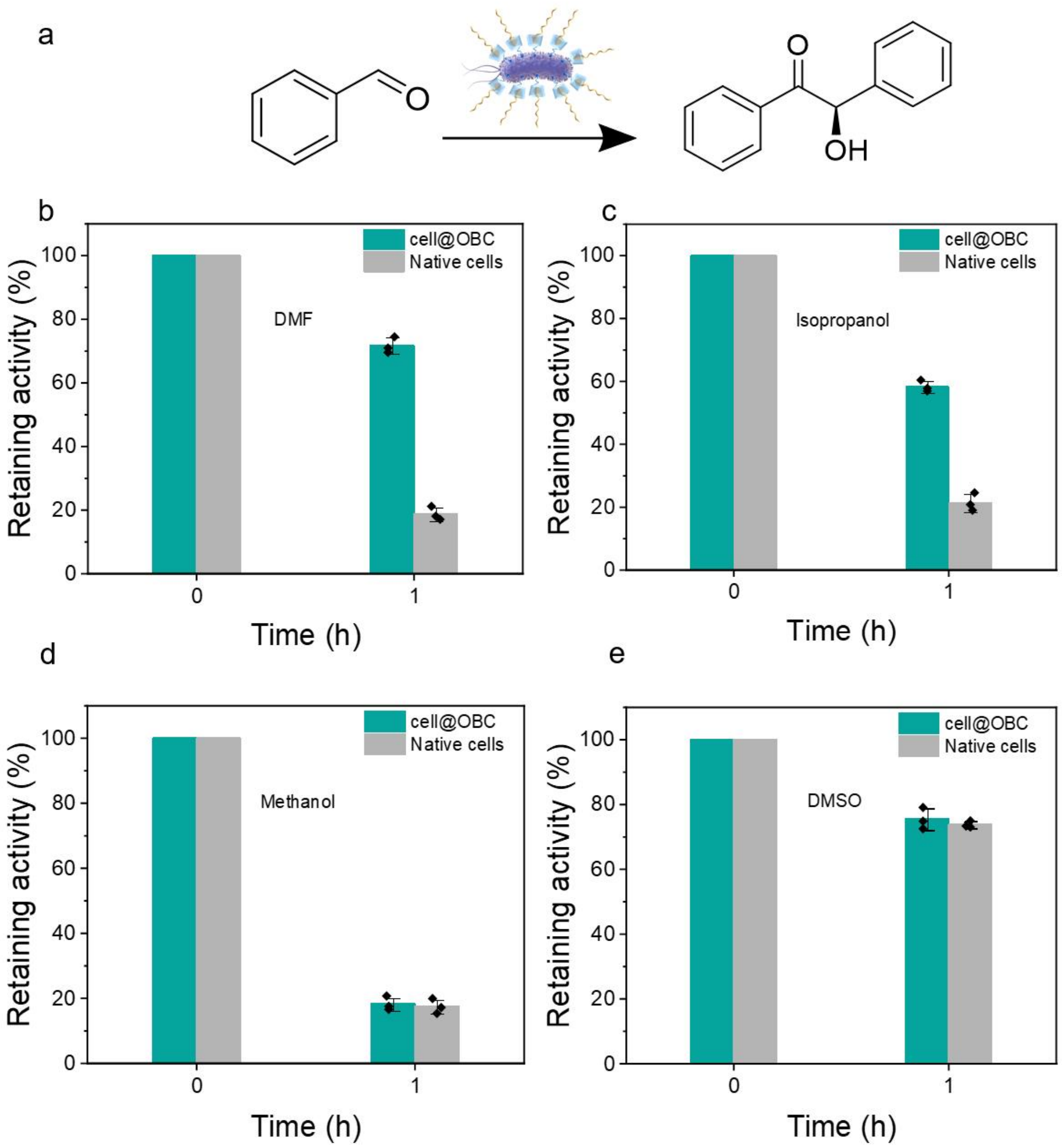

Supplementary Figure 12. General robustness of native cells and cell@OBC in different solvents. The retaining activity of intracellular enzymes is assessed through the BAL-induced benzoin condensation reaction (a). (b) Stability of the intracellular enzyme BAL in a water-DMF mixture (v/v = 50:50). (c) Stability of the intracellular enzyme BAL in a water-isopropanol mixture (v/v = 50:50). (d) Stability of the intracellular enzyme BAL in a water-methanol mixture (v/v = 50:50). (e) Stability of the intracellular enzyme BAL in a water-DMSO mixture (v/v = 50:50). Data are presented as mean values +/- SD based on n=3 independent replicates with the error bars representing the SD.

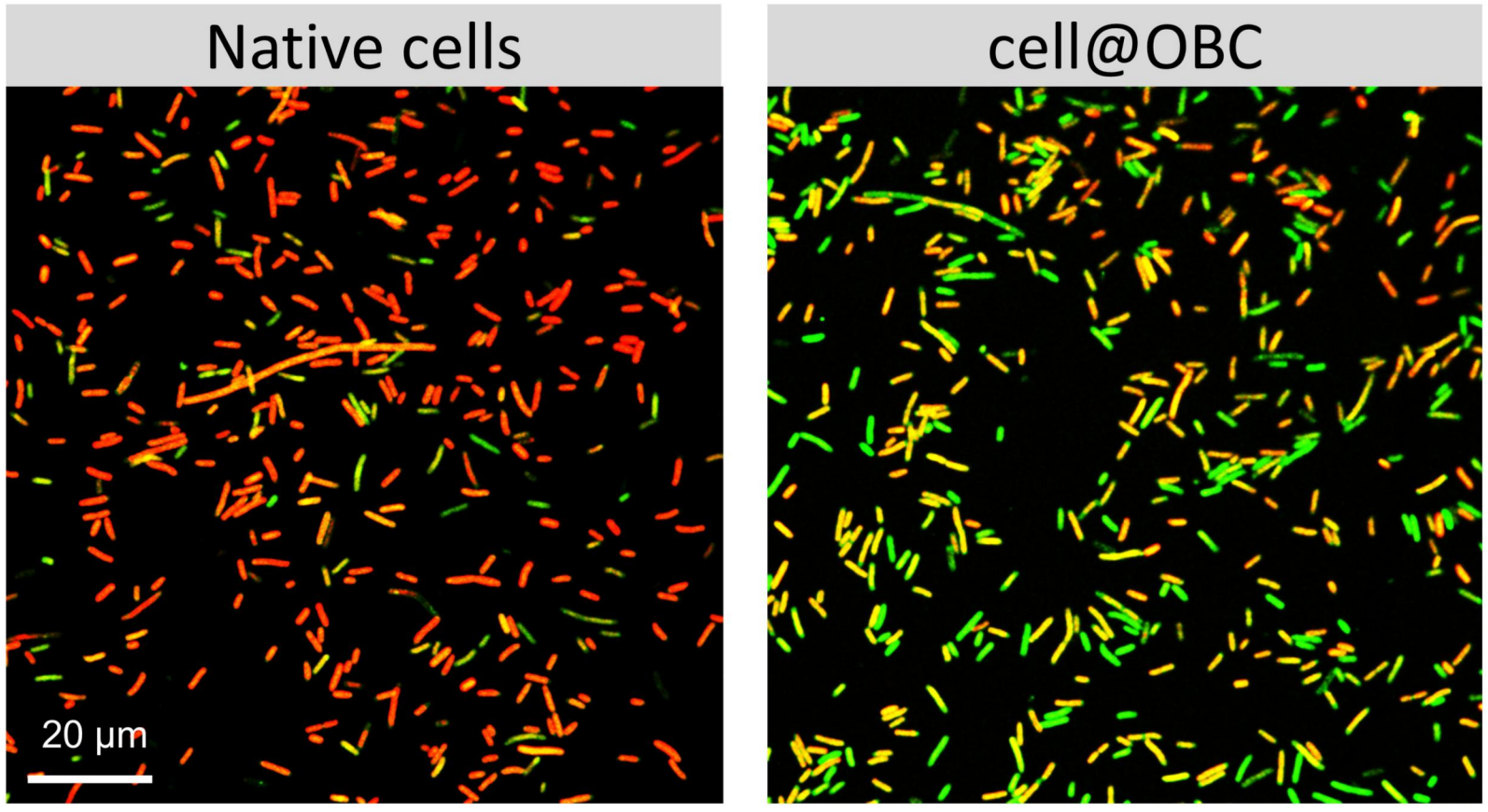

Supplementary Figure 13. Live/dead assay of native cells and cell@OBC after 2 hours of UV exposure. The experiment was repeated three times independently with similar results.

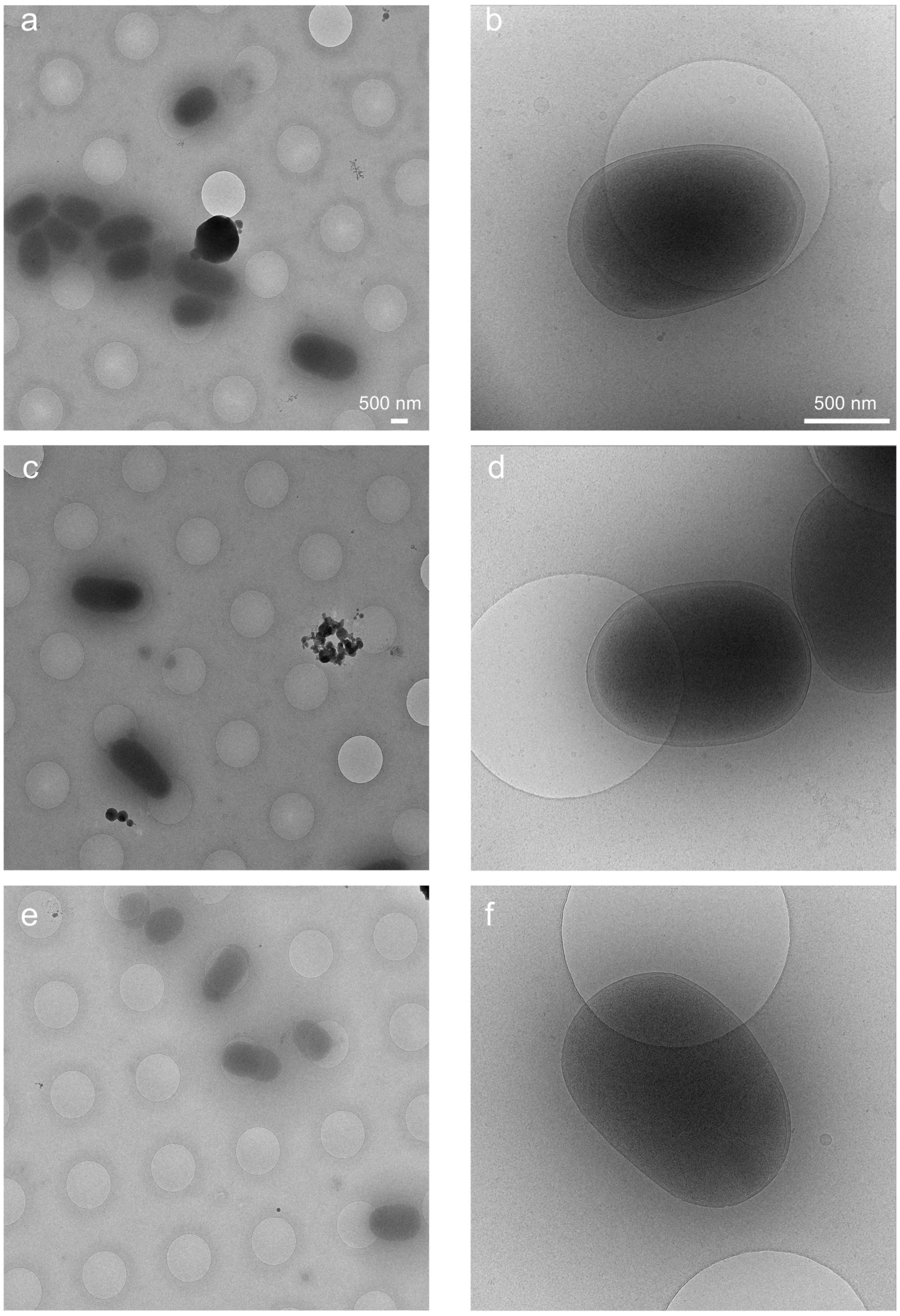

Supplementary Figure 14. Representative cryo-TEM images of (a) native cell at 3,400x magnification, (b) native cell at 17,500x magnification, (c) cell@CD at 3400x magnification, and (d) cell@CD at 17,500x magnification, (e) cell@OBC at 3,400x magnification, (f) cell@OBC at 17,500x magnification. Intact inner and outer *E.coli* membranes were observed for all measured samples.

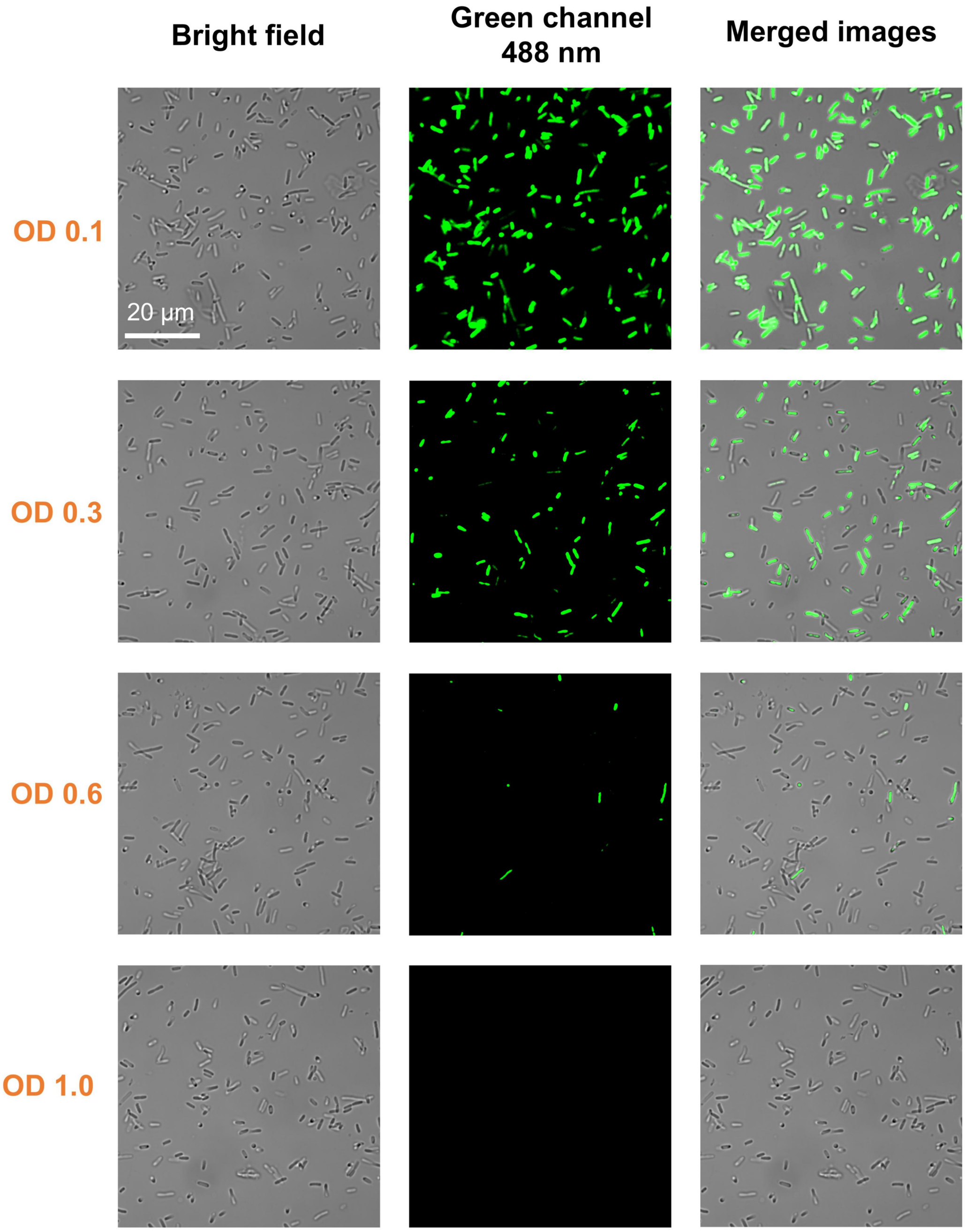

Supplementary Figure 15. Bright-field and fluorescence images of conjugate cells with different OD_{600} during regrowth. The experiment was repeated three times independently with similar results.

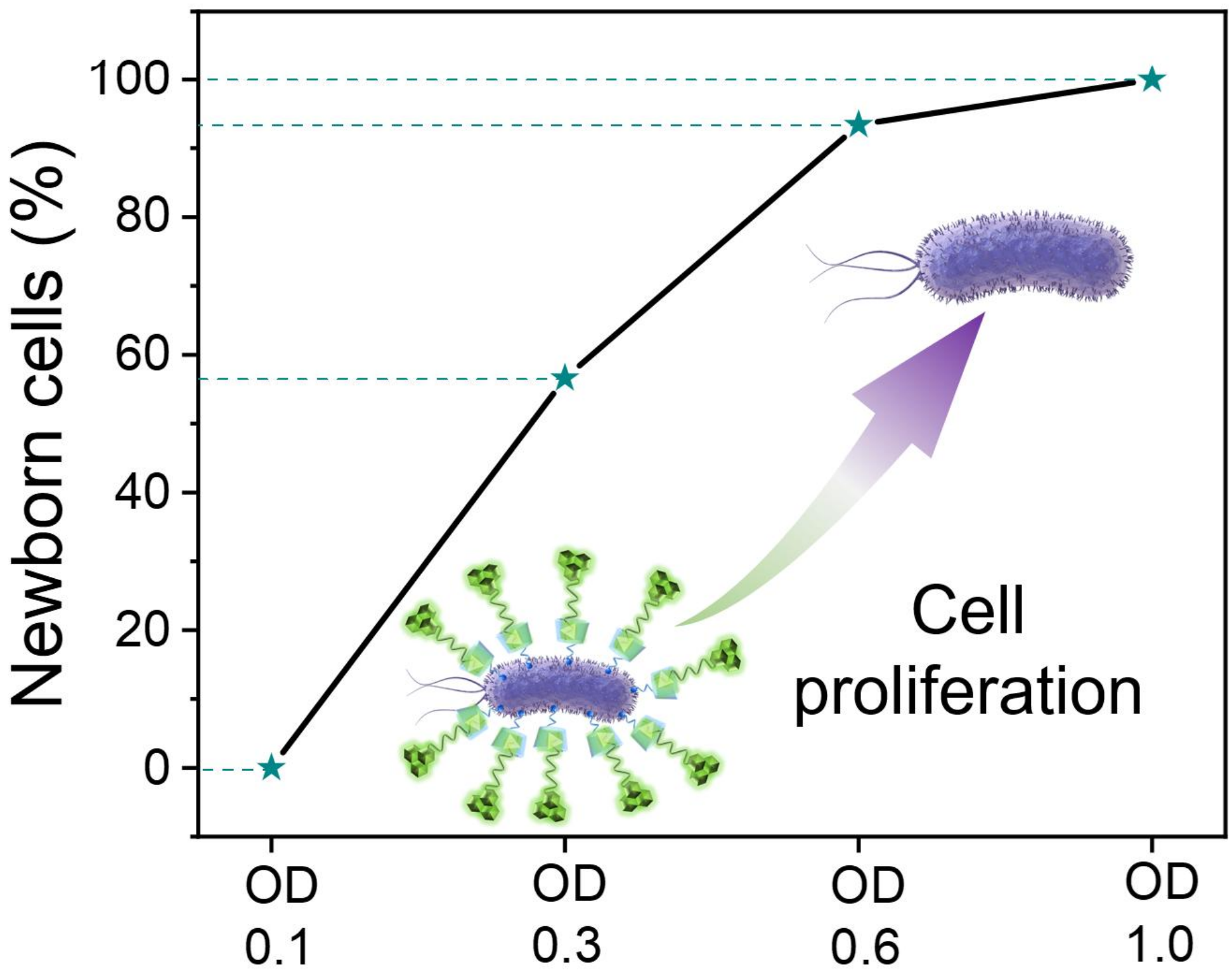

Supplementary Figure 16. New generations of cells at different OD_{600}.

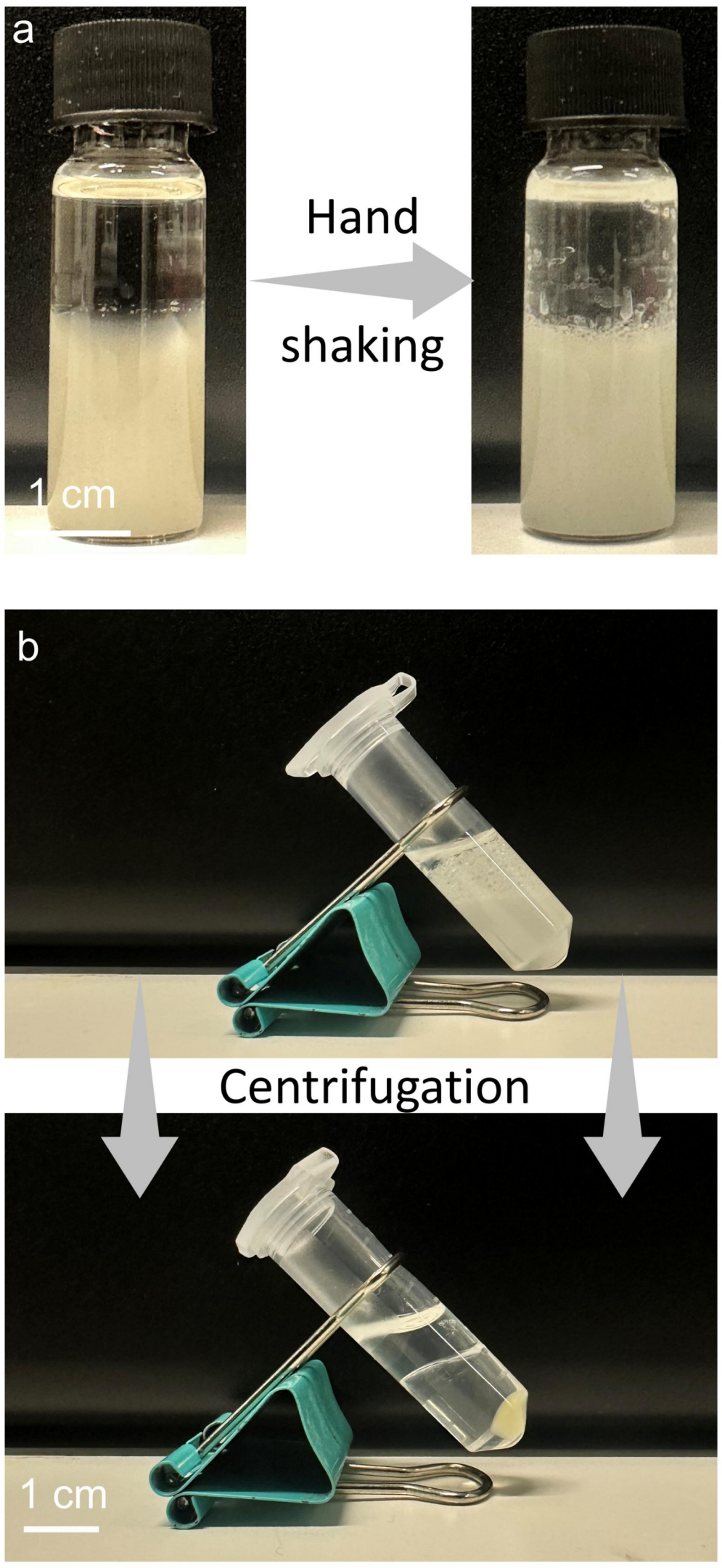

Supplementary Figure 17. (a) The replicated cells lose the ability to emulsify as the hydrophobic properties on the surface are lost. (b) The replicated cells can still be recycled by centrifugation, despite the loss of the functionalized interface.

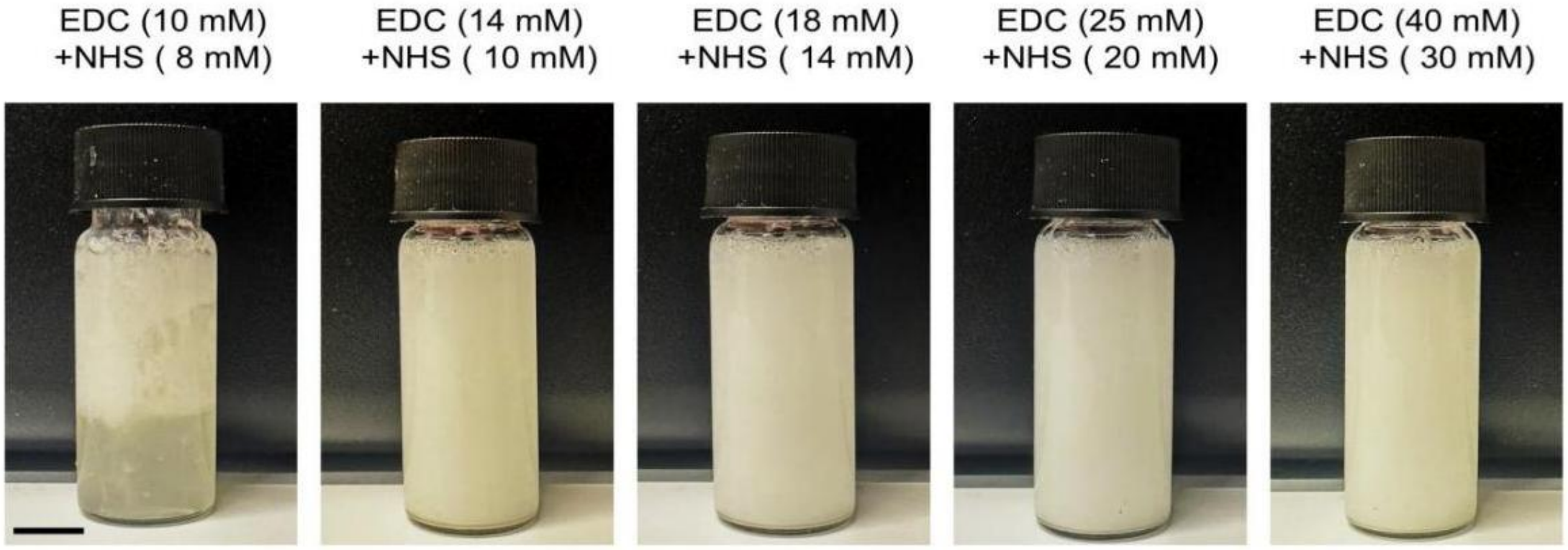

Supplementary Figure 18. Macrophotographs of emulsions formed by conjugates prepared with different amounts of coupling agents (scale bar: 0.5 cm).

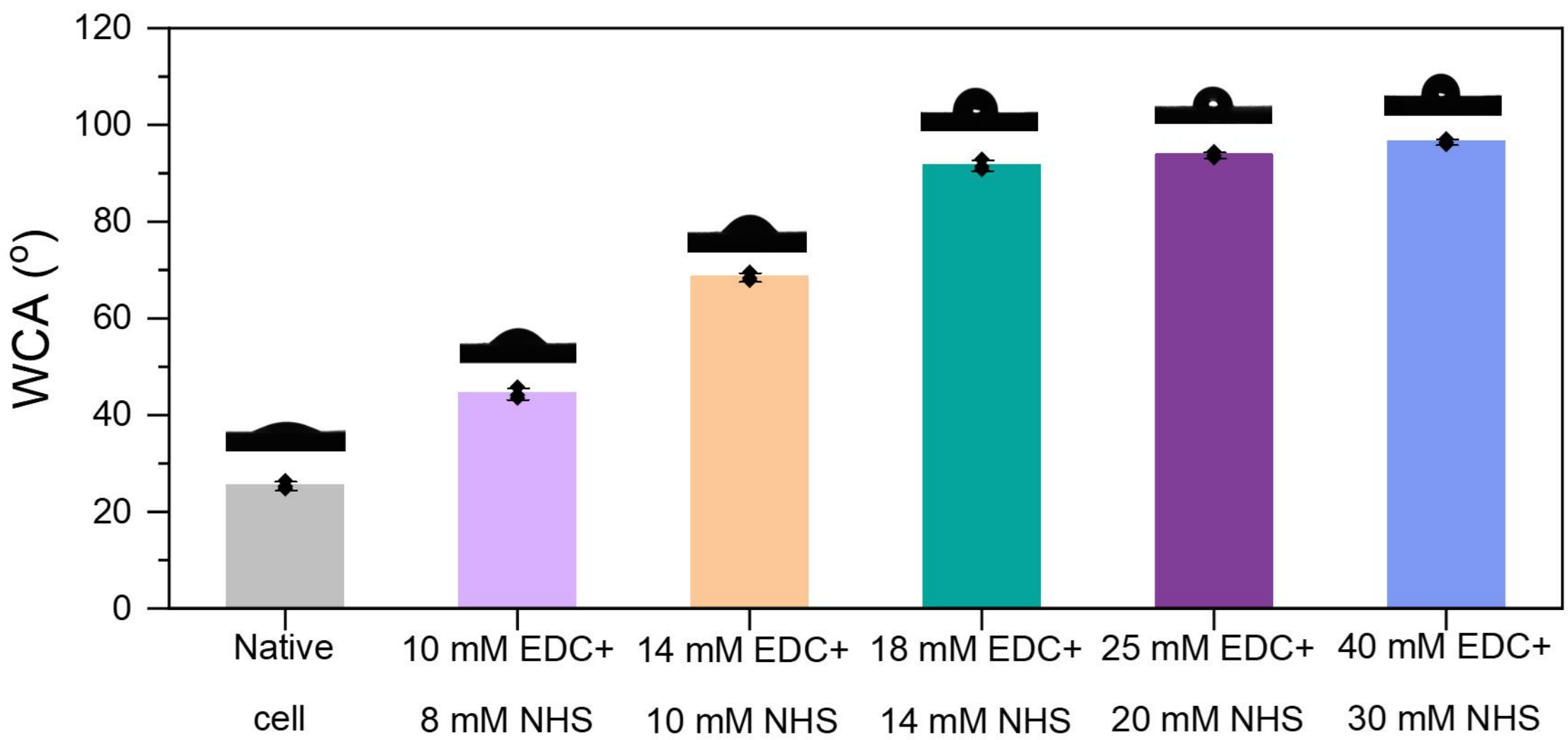

Supplementary Figure 19. Water contact angle of the native cell and all conjugates. Data are presented as mean values +/- SD based on n=3 independent replicates with the error bars representing the SD.

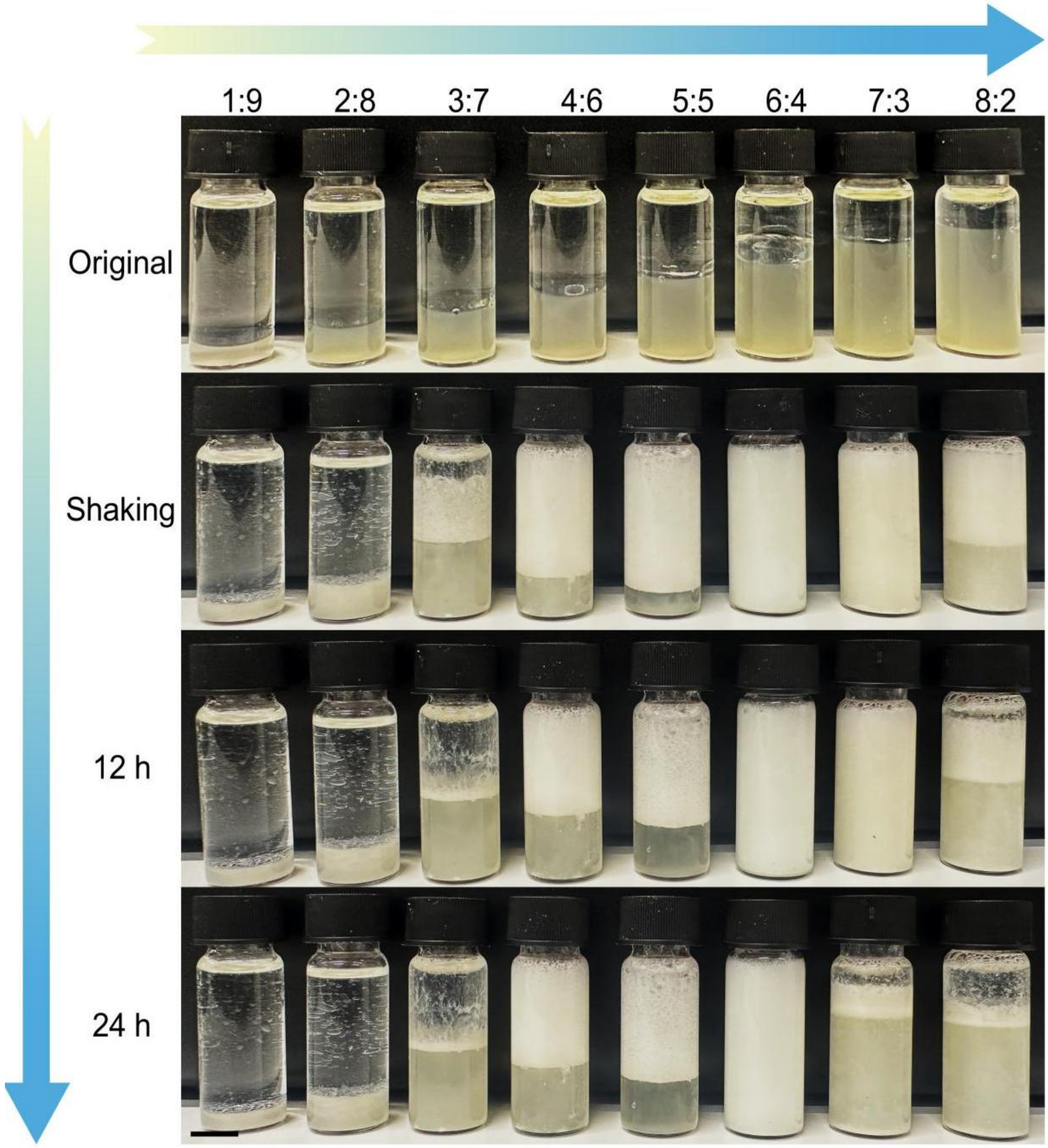

Supplementary Figure 20. Images of emulsion systems with different water-to-oil ratios after handshaking and standing for different periods (scale bar: 0.5 cm).

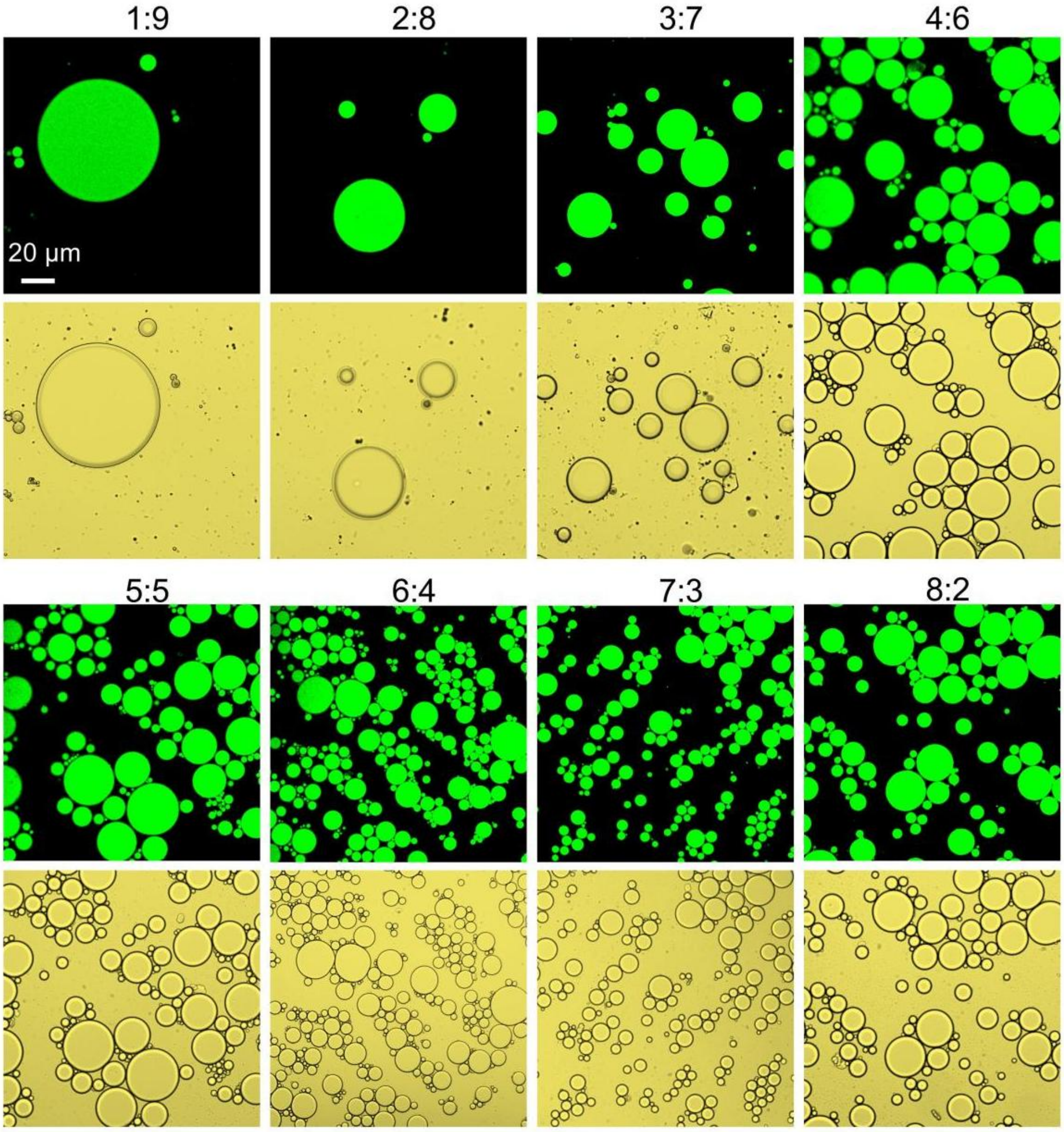

Supplementary Figure 21. The fluorescence microscopy images of emulsion droplets formed by conjugates with different water-to-oil ratios from 1:9 to 8:2. The experiment was repeated three times independently with similar results.

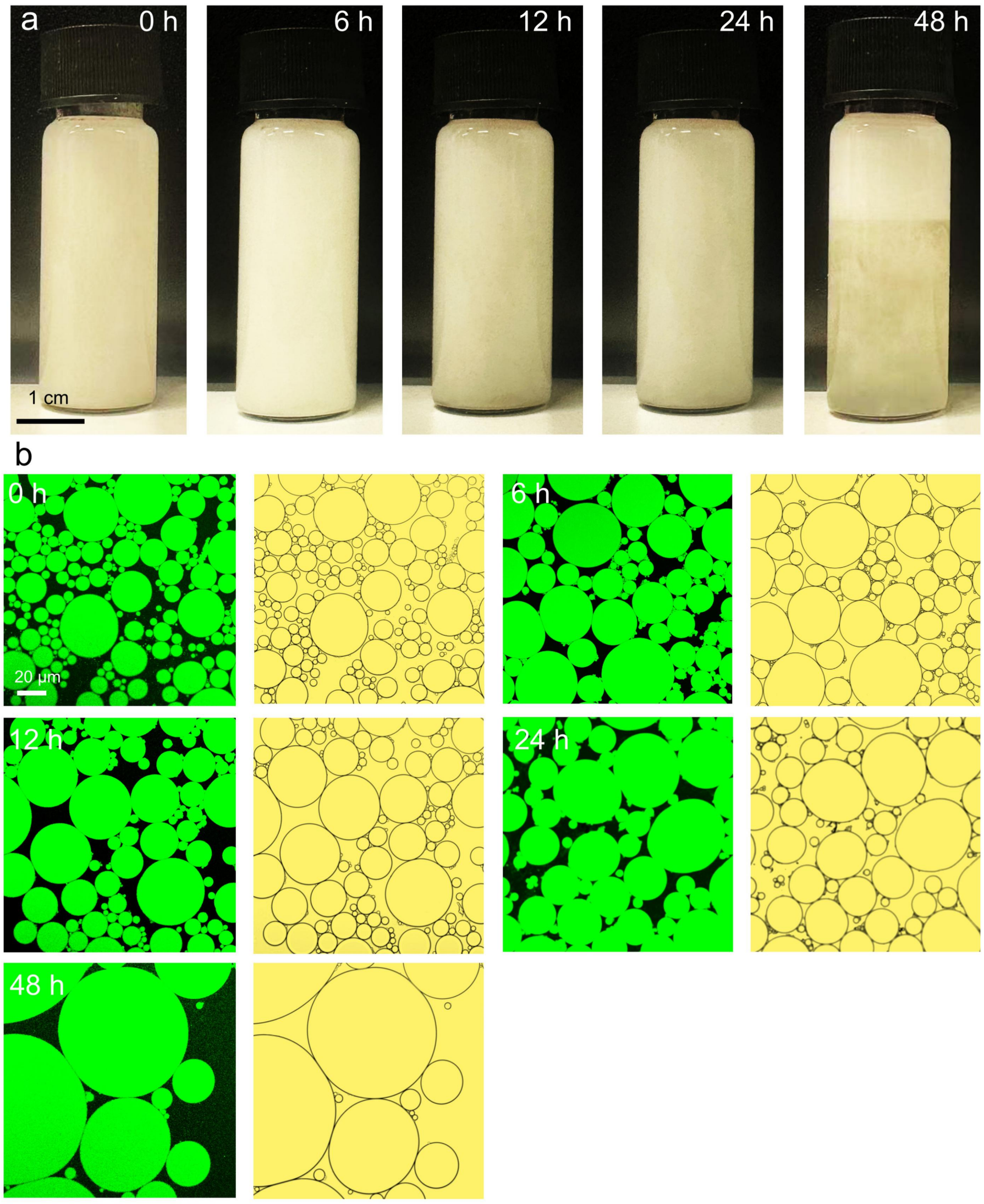

Supplementary Figure 22. (a) Macroscopic photos of the emulsion after standing for different periods of time. (b) The morphology of cell@OBC stabilized Pickering emulsions was recorded by CLSM at 0, 6, 12, 24 and 48 hours. At 48 hours, coalescence of droplets was observed. The experiment was repeated three times independently with similar results.

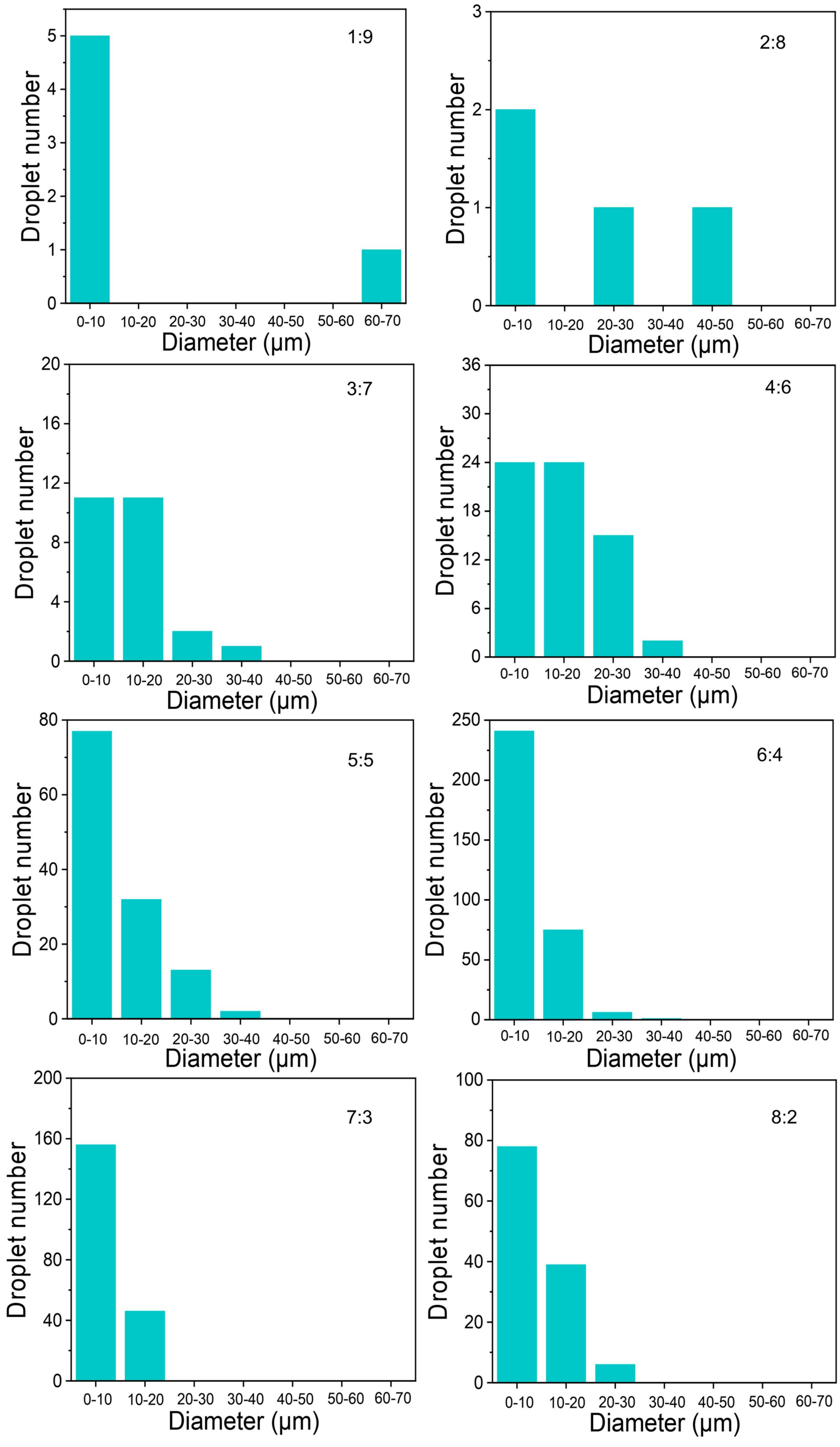

Supplementary Figure 23. The number and size distribution of emulsion droplets formed by conjugates with different water-to-oil ratios from 1:9 to 8:2.

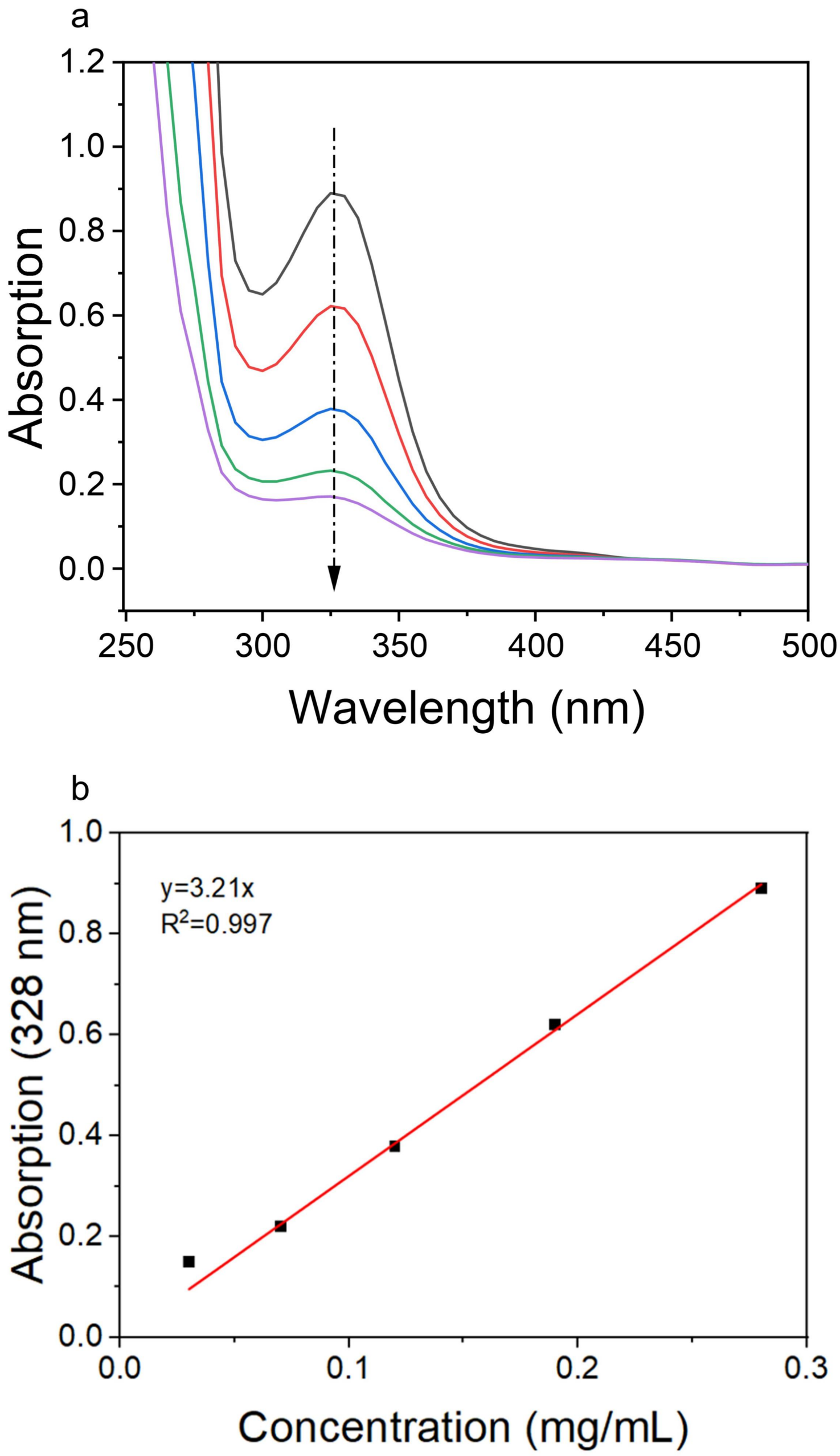

Supplementary Figure 24. UV absorption (a) and standard calibration curve (b) of the photocatalyst.

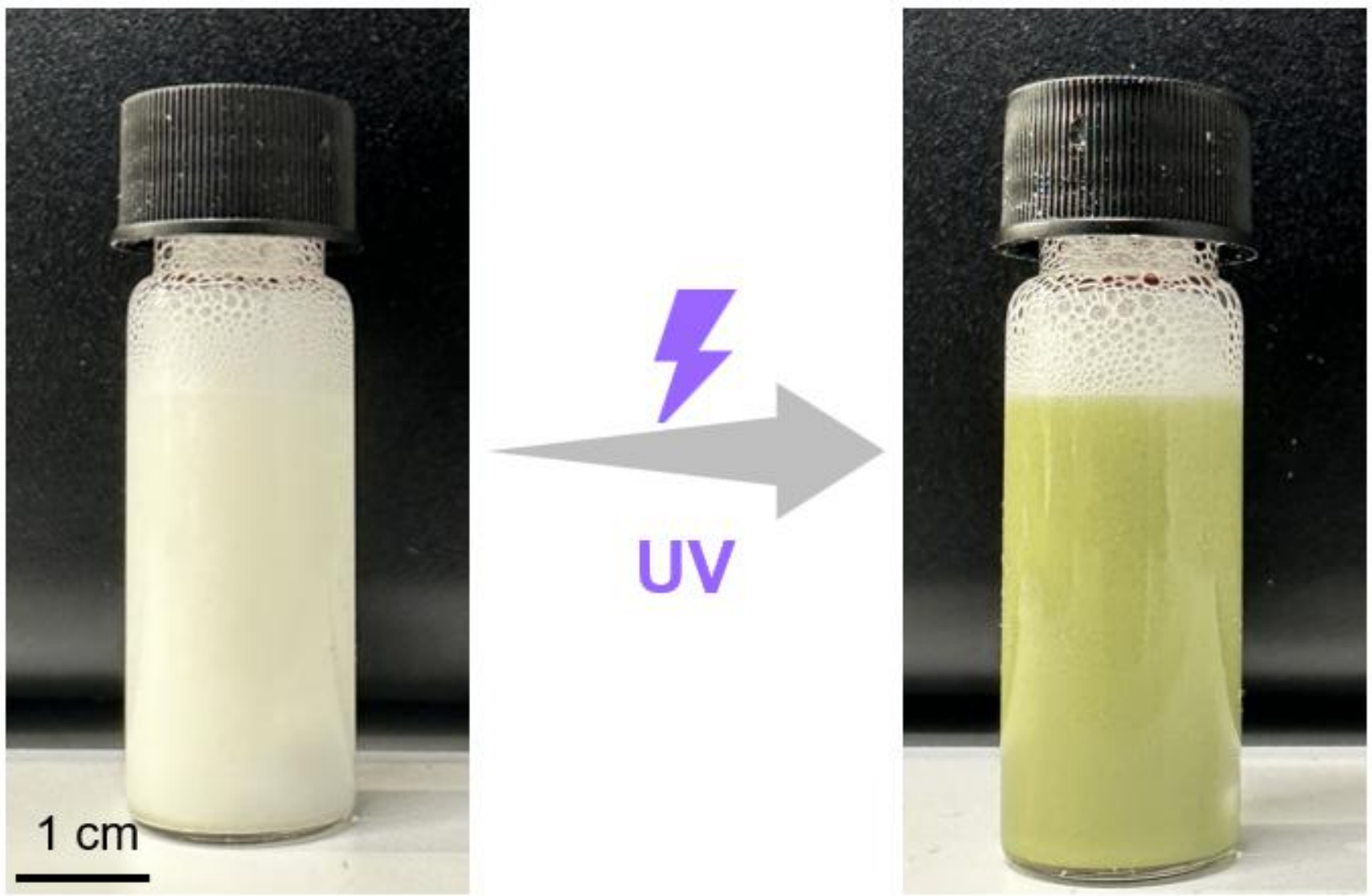

Supplementary Figure 25. Macroscopic changes in cell@OBC-stabilized emulsions before and after photocatalytic reactions.

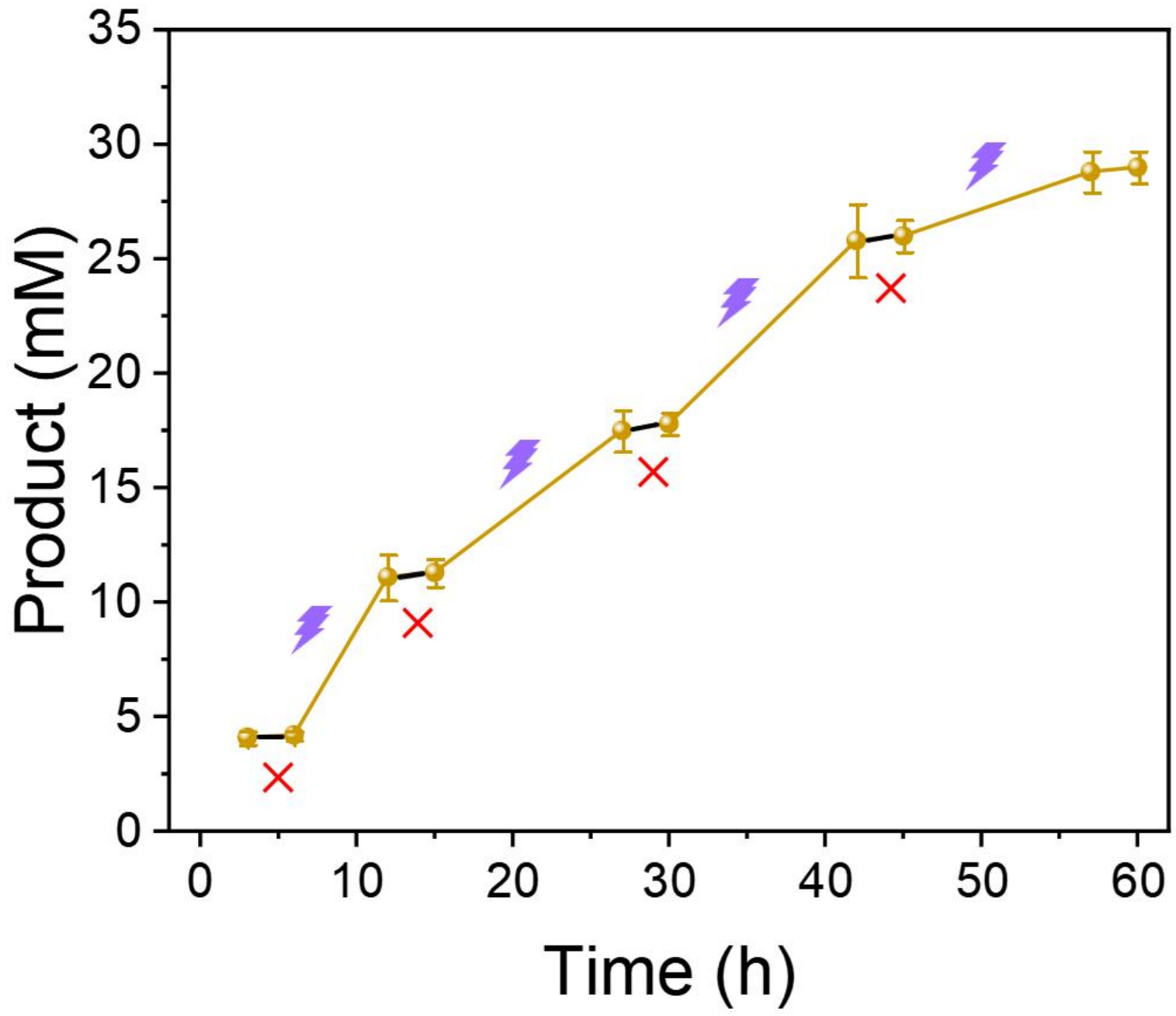

Supplementary Figure 26. Time-dependent benzaldehyde formation under alternating UV light irradiation and dark conditions. The yellow line corresponds to periods of UV irradiation, whereas the black line indicates dark intervals (3 h each). A significant difference in reaction rates is observed between light-on and light-off periods, with faster benzaldehyde accumulation under UV, confirming the controllable of the photocatalytic. Data are presented as mean values +/- SD based on n=3 independent replicates with the error bars representing the SD.

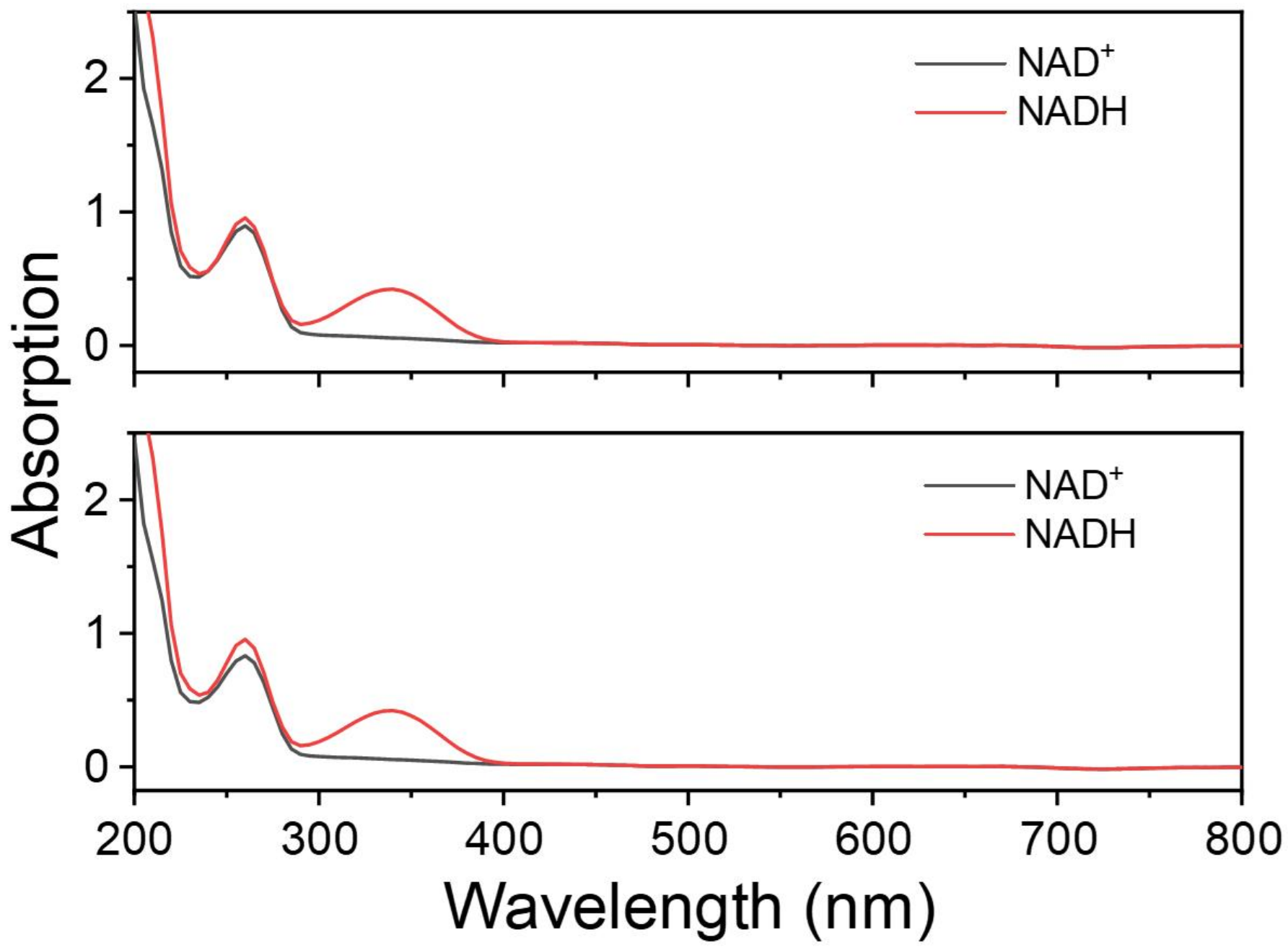

Supplementary Figure 27. The UV-Vis spectra of NAD^+ and NADH before and after 12 hours of incubation in KPI buffer confirm their stability.

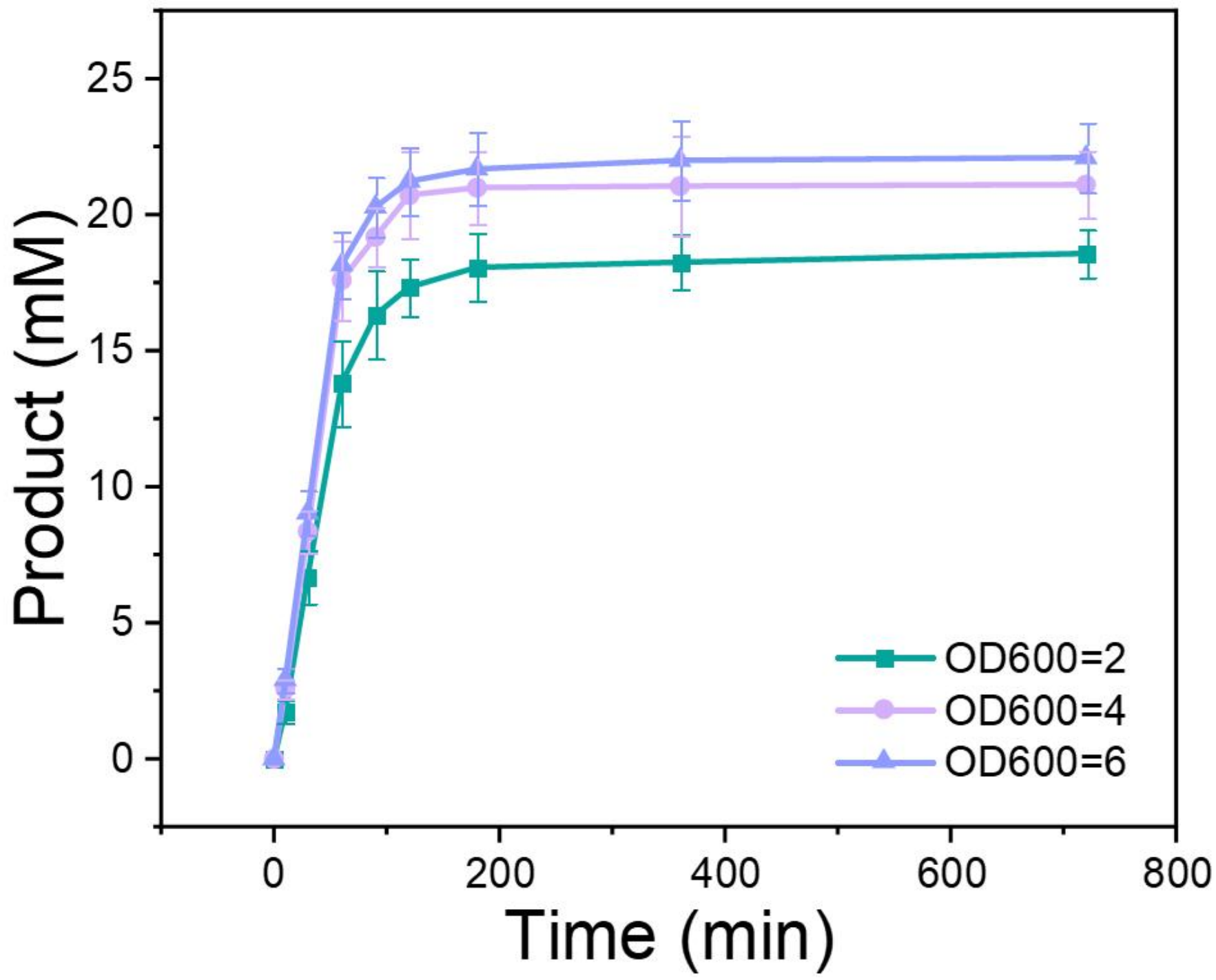

Supplementary Figure 28. Profiles of multienzymatic cascade reaction by conjugate cells with different OD_{600} values. The error bars represent the standard deviations of three parallel measurements, n=3.

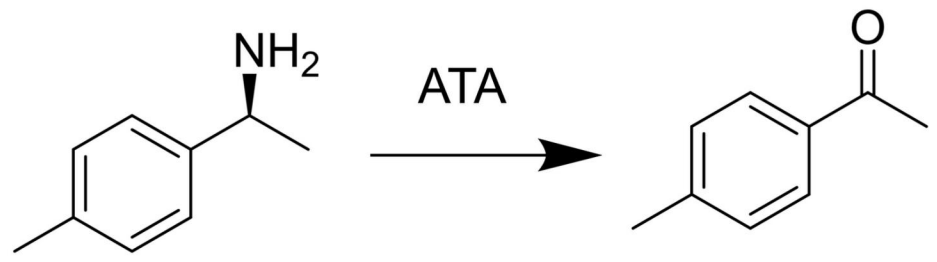

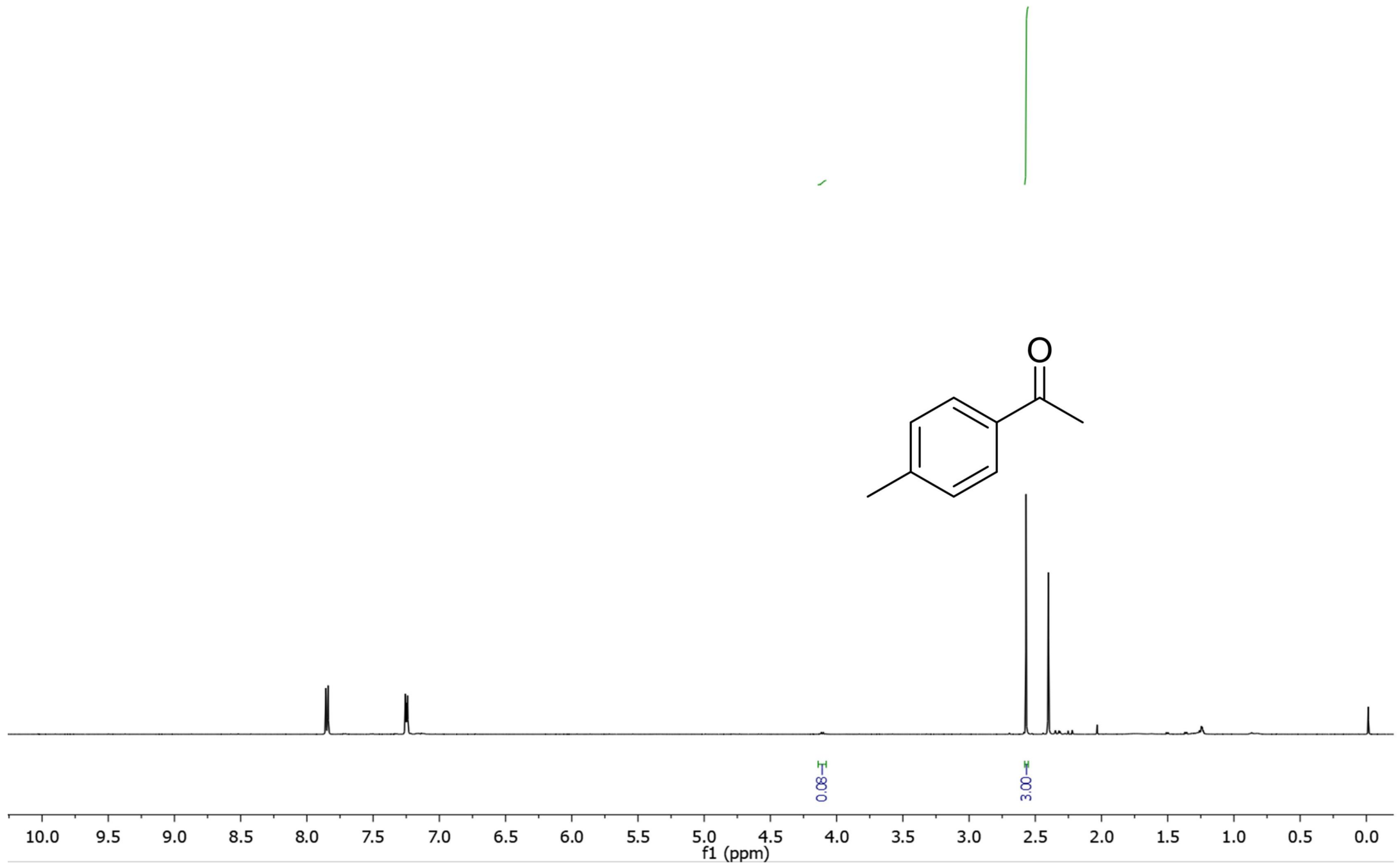

Supplementary Figure 29. ^{1}H NMR spectrum of crude 1-(p-tolyl)ethanol, reactions catalyzed by *E. coli* overexpressing ATA.

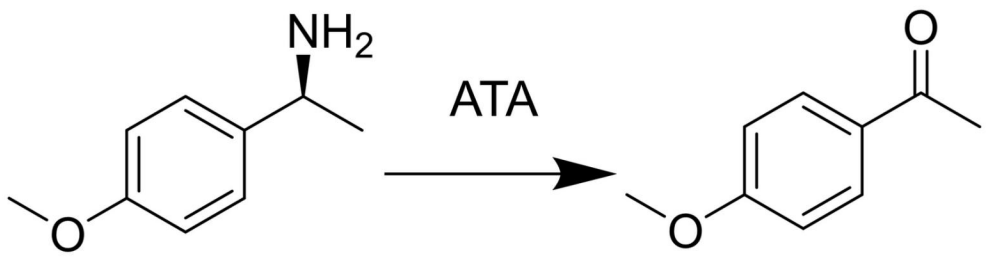

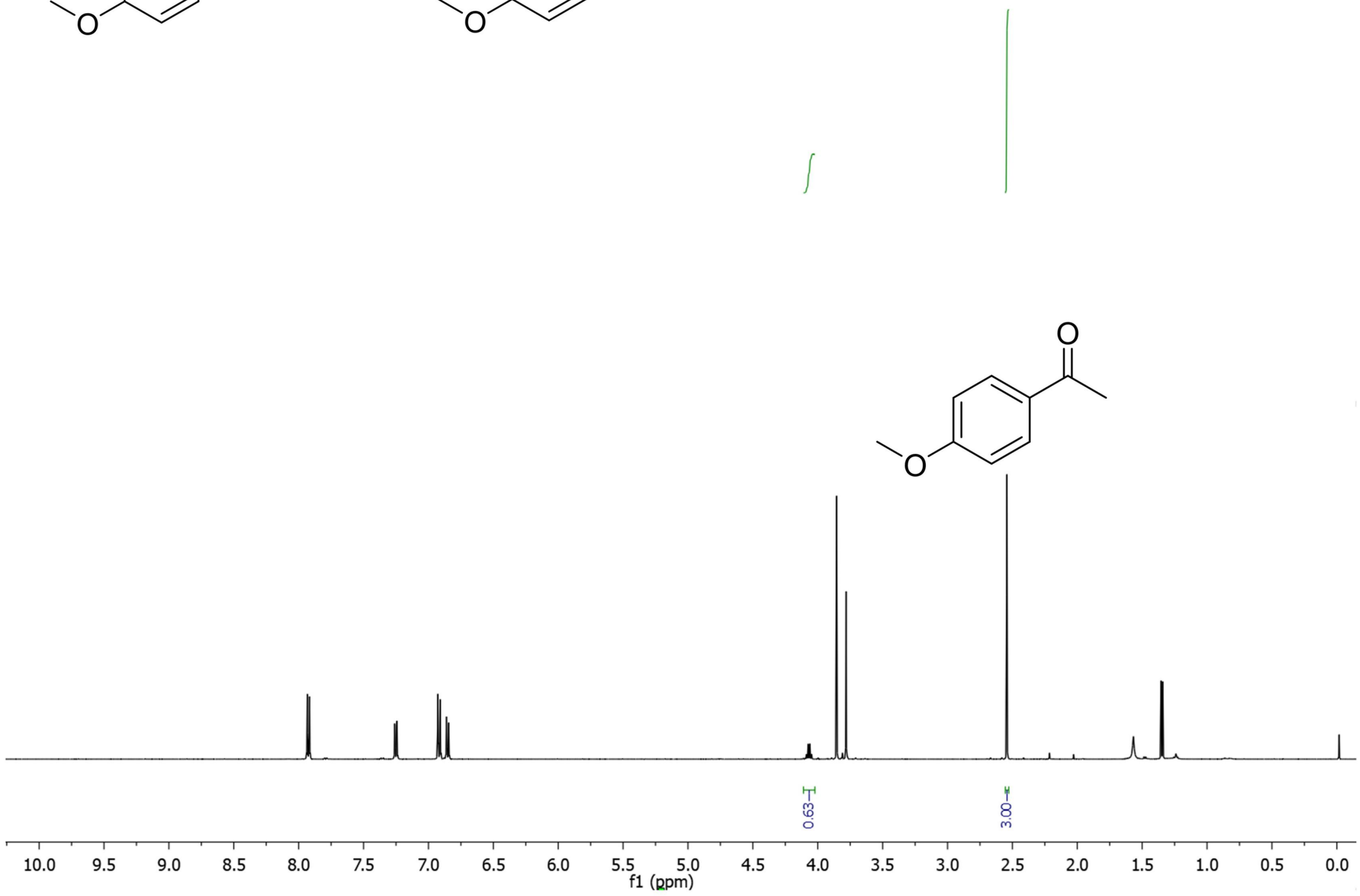

Supplementary Figure 30. ^{1}H NMR spectrum of crude 4'-methoxyacetophenone, reactions catalyzed by *E. coli* overexpressing ATA.

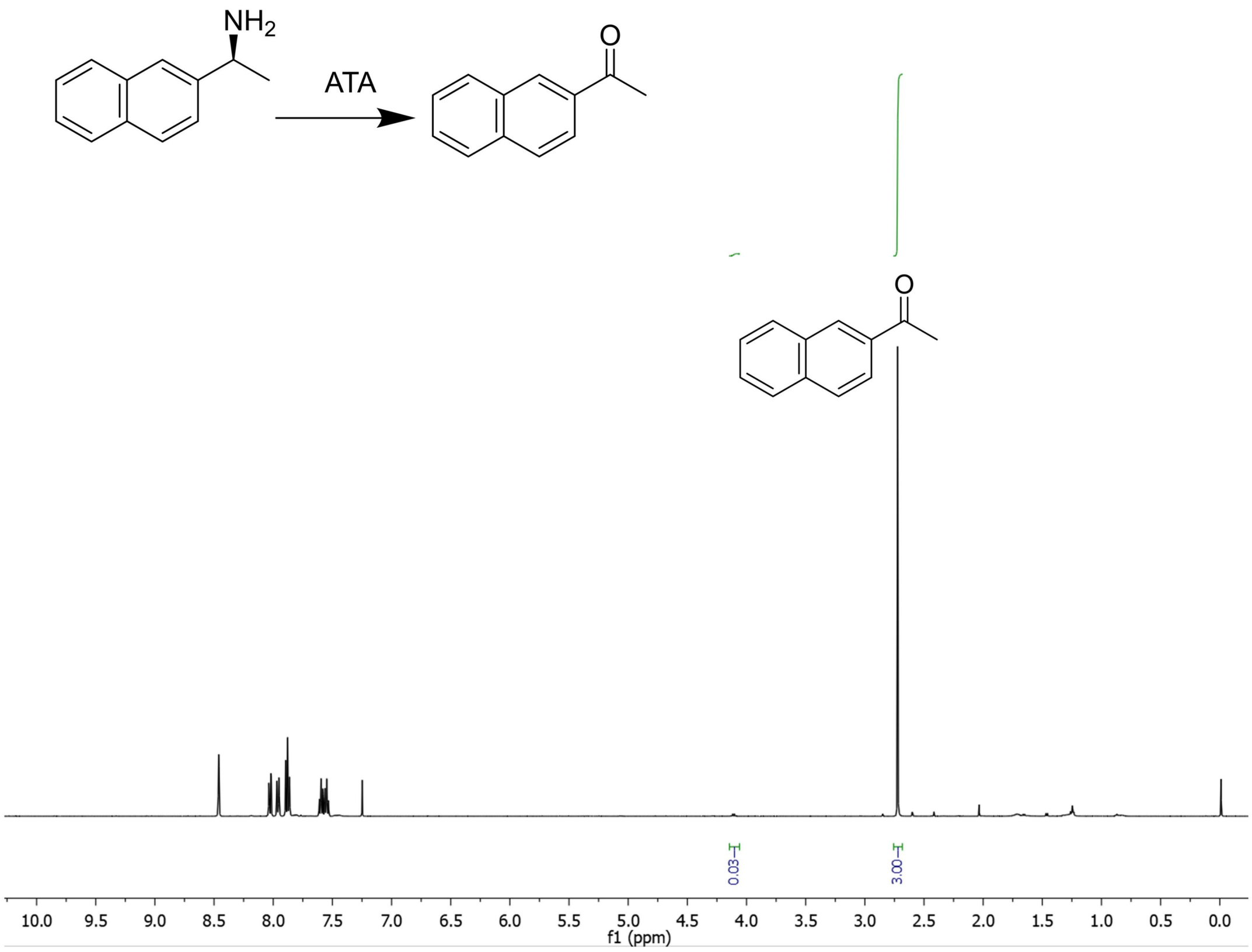

Supplementary Figure 31. ^{1}H NMR spectrum of crude 2-acetonaphthone, reactions catalyzed by *E. coli* overexpressing ATA.

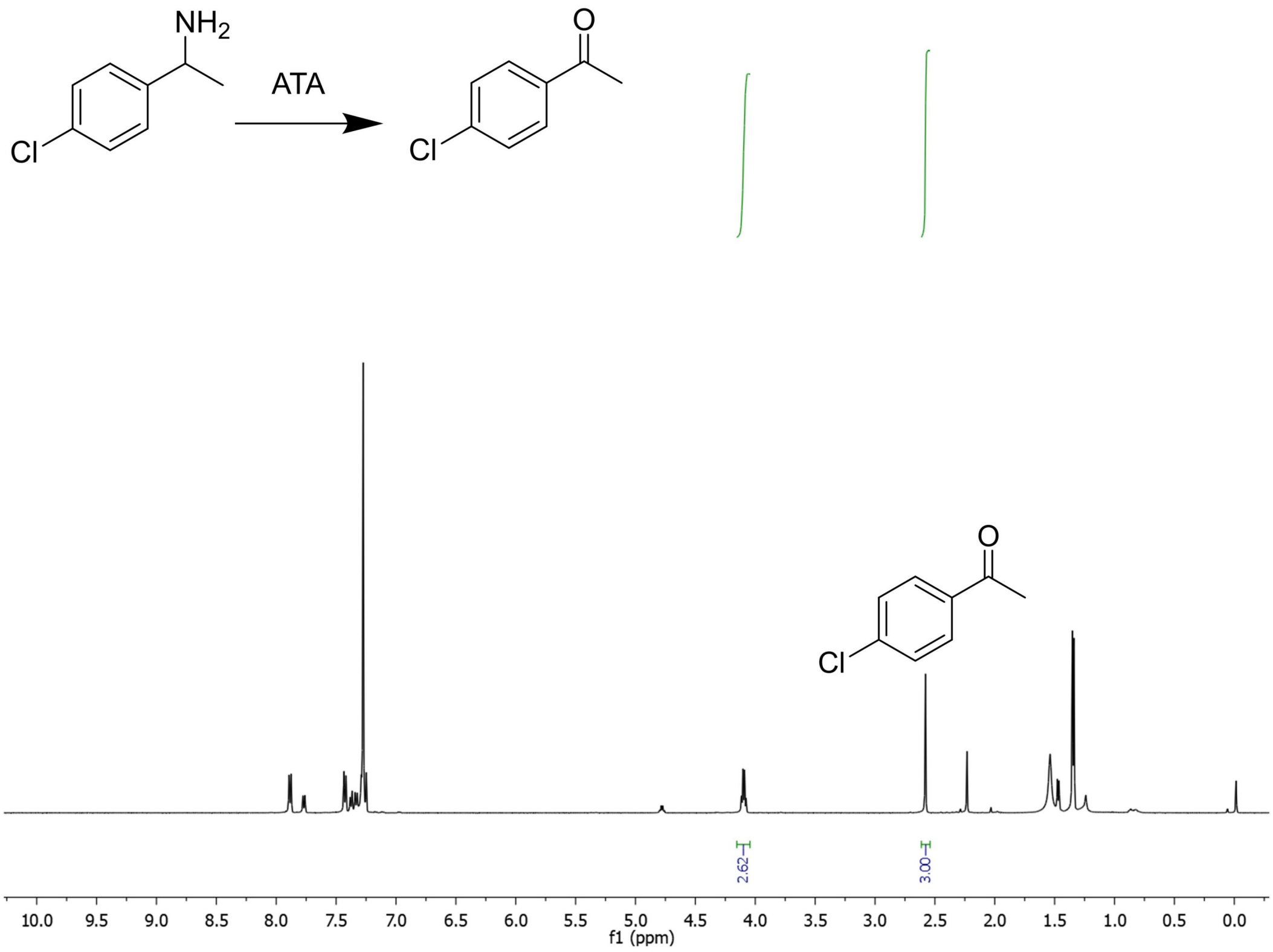

Supplementary Figure 32. ^{1}H NMR spectrum of crude 1-(4-chlorophenyl)ethan-1-one, reactions catalyzed by *E. coli* overexpressing ATA.

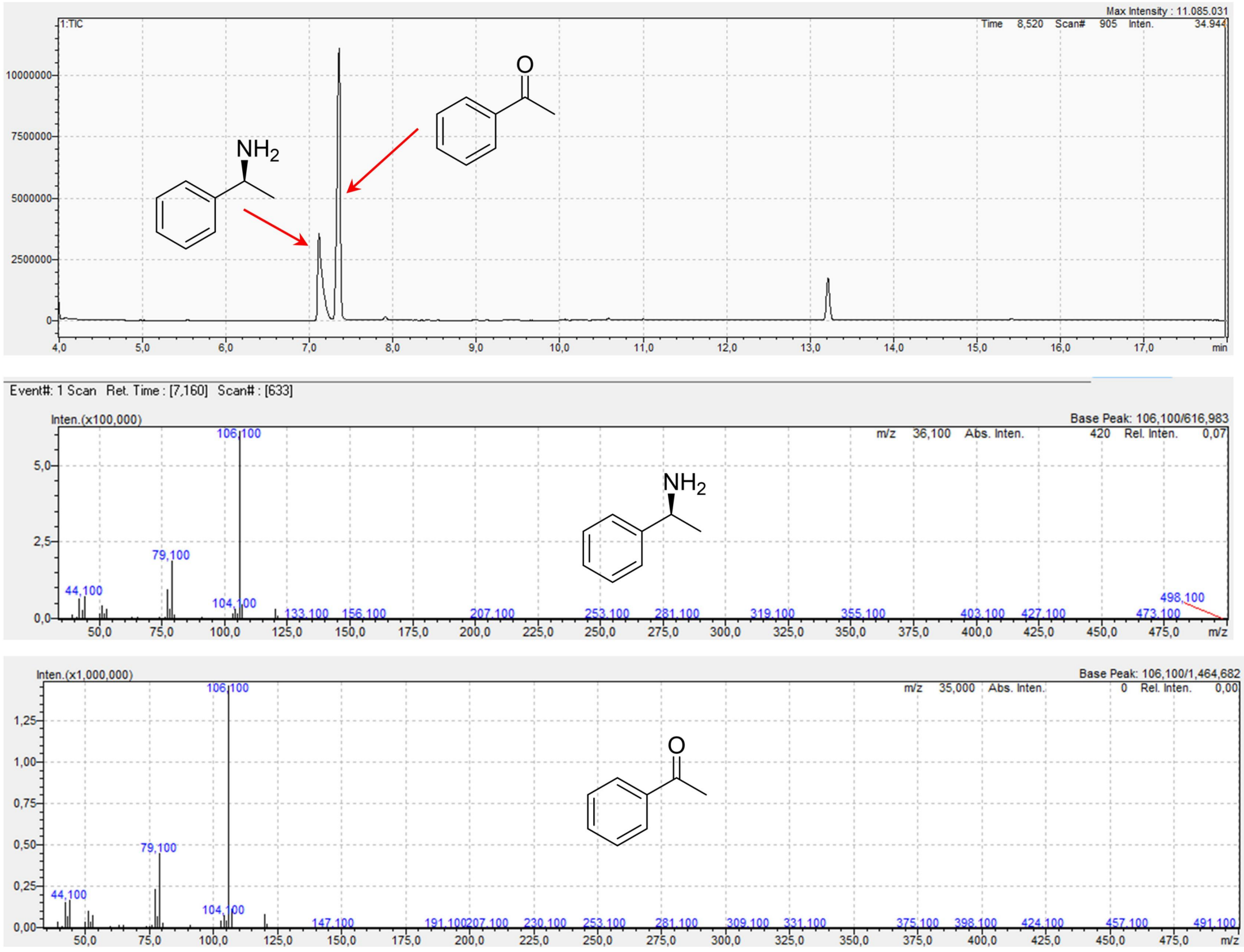

Supplementary Figure 33. GC and MS spectra of single-step catalysis from (*S*)-1-phenylethylamine to acetophenone.

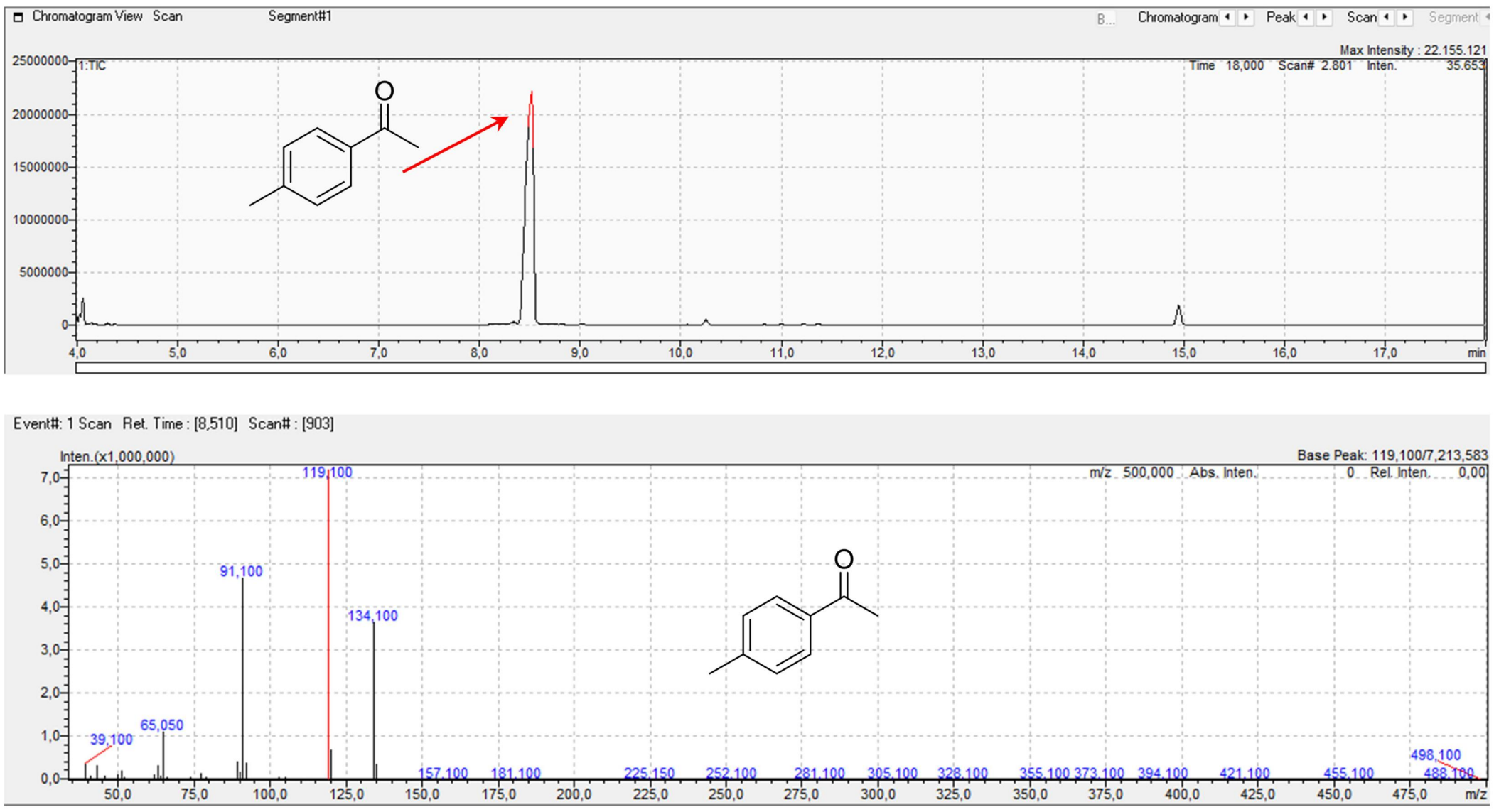

Supplementary Figure 34. GC and MS spectra of single-step catalysis from (*S*)-(-)-1-(p-tolyl)ethylamine to 1-(p-tolyl)ethanol.

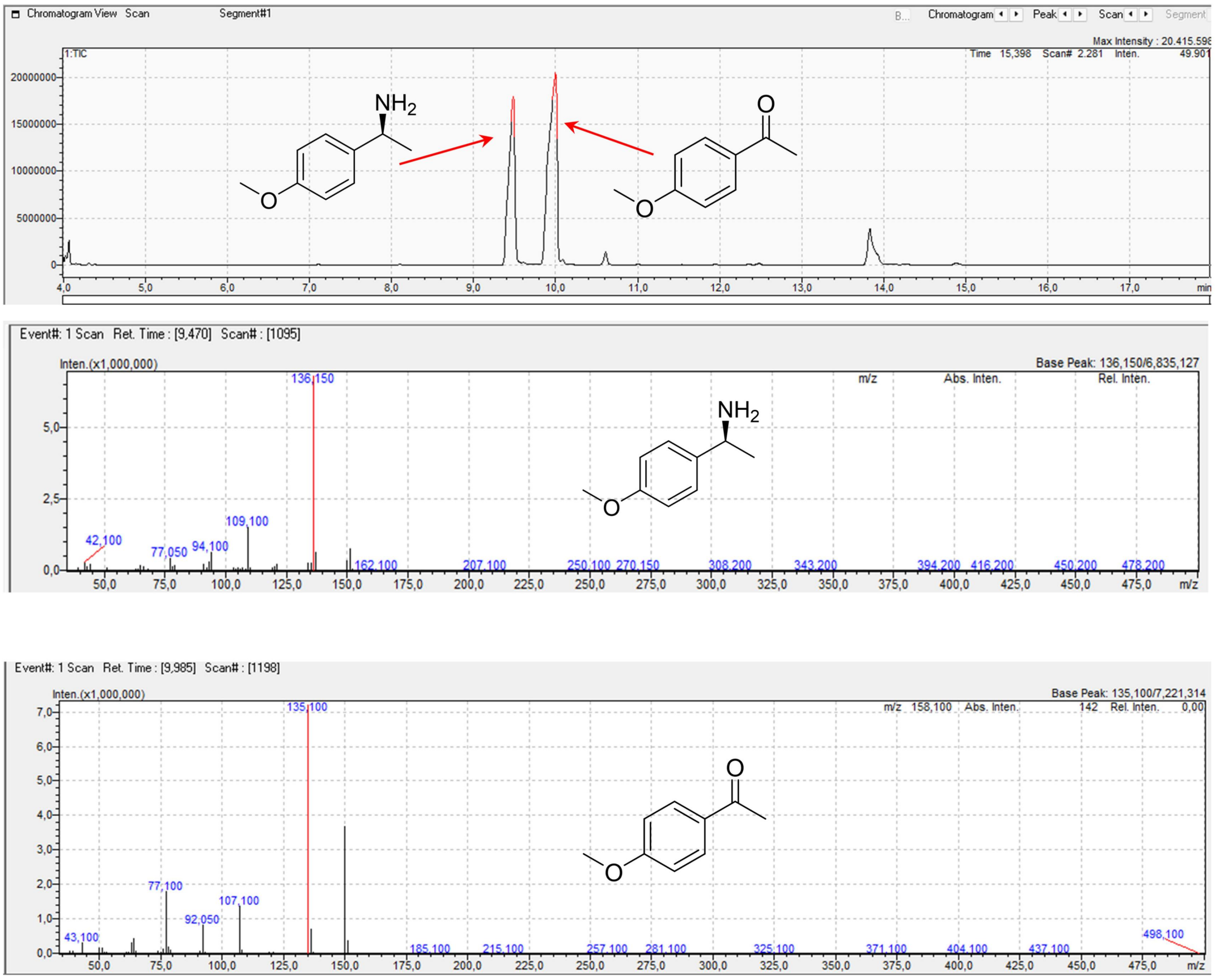

Supplementary Figure 35. GC and MS spectra of single-step catalysis from (*S*)-1-(4-methoxyphenyl)ethylamine to 4'-methoxyacetophenone.

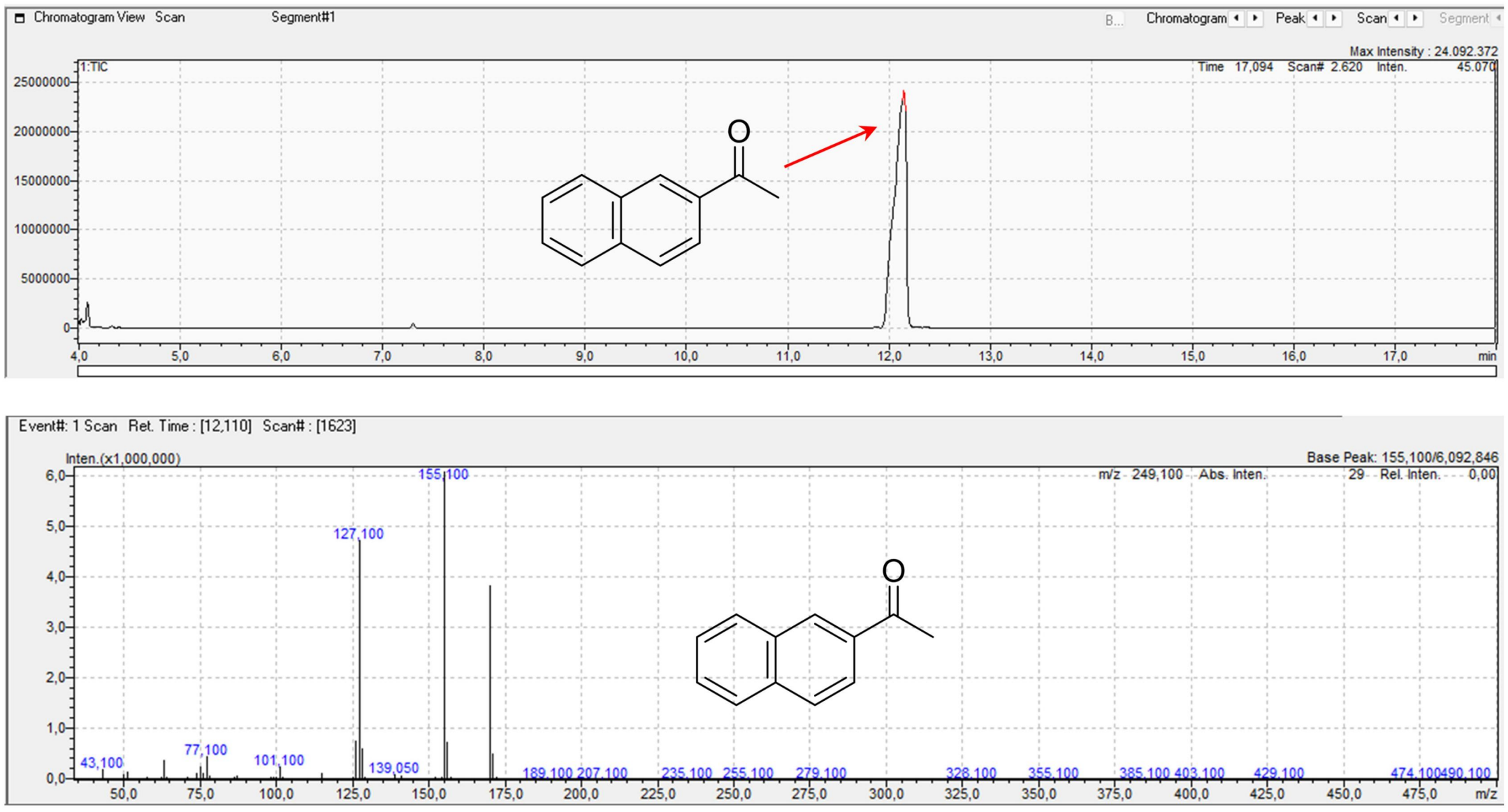

Supplementary Figure 36. GC and MS spectra of single-step catalysis from (*S*)-1-(naphthalen-2-yl)ethanamine to 2-acetonaphthone.

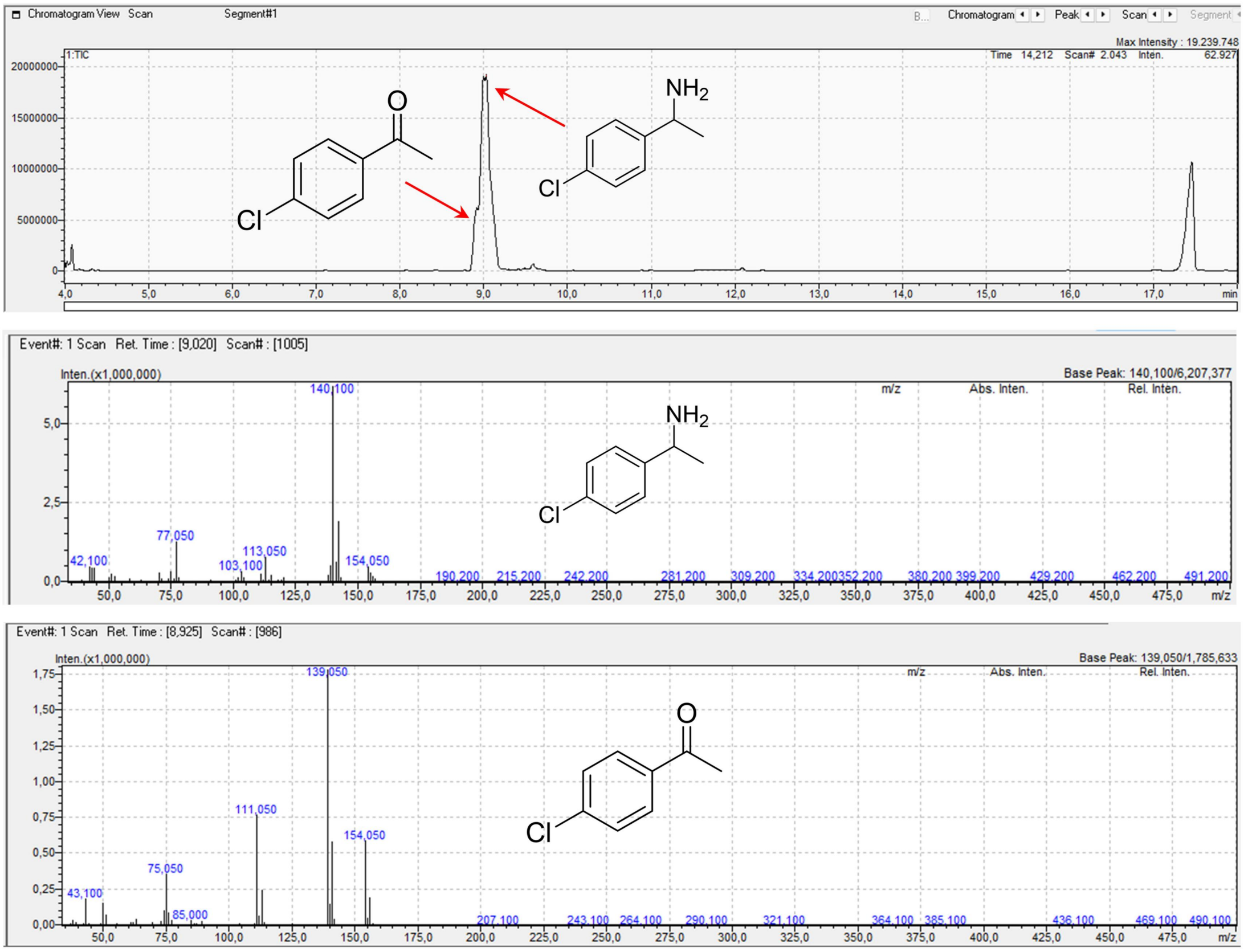

Supplementary Figure 37. GC and MS spectra of single-step catalysis from 1-(4-chlorophenyl)ethylamine to 4′-chloroacetophenone.

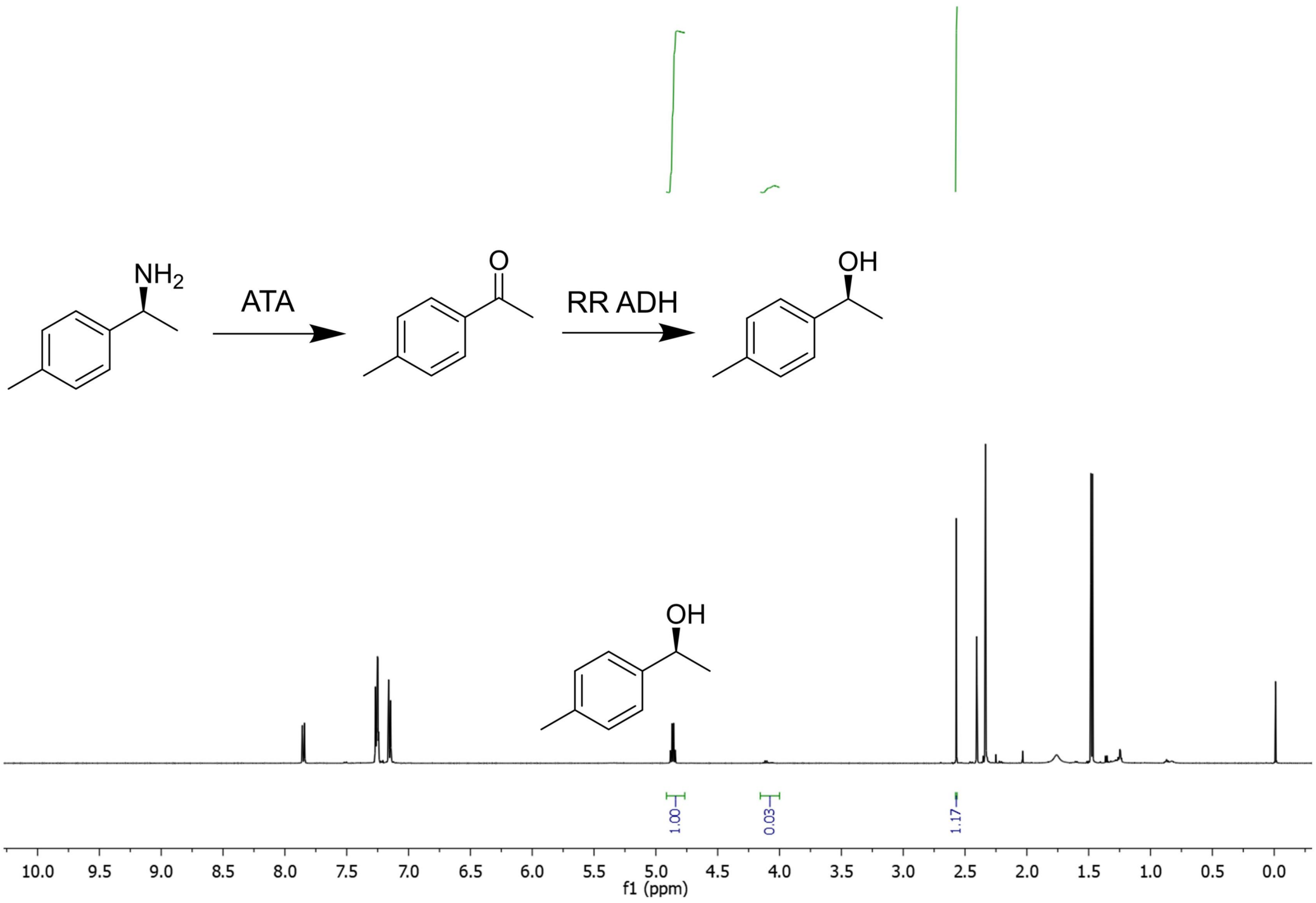

Supplementary Figure 38. ^{1}H NMR spectrum of crude 1-(p-tolyl)ethanol, synthesized by cascade catalysis in *E. coli* overexpressing ATA and RR ADH.

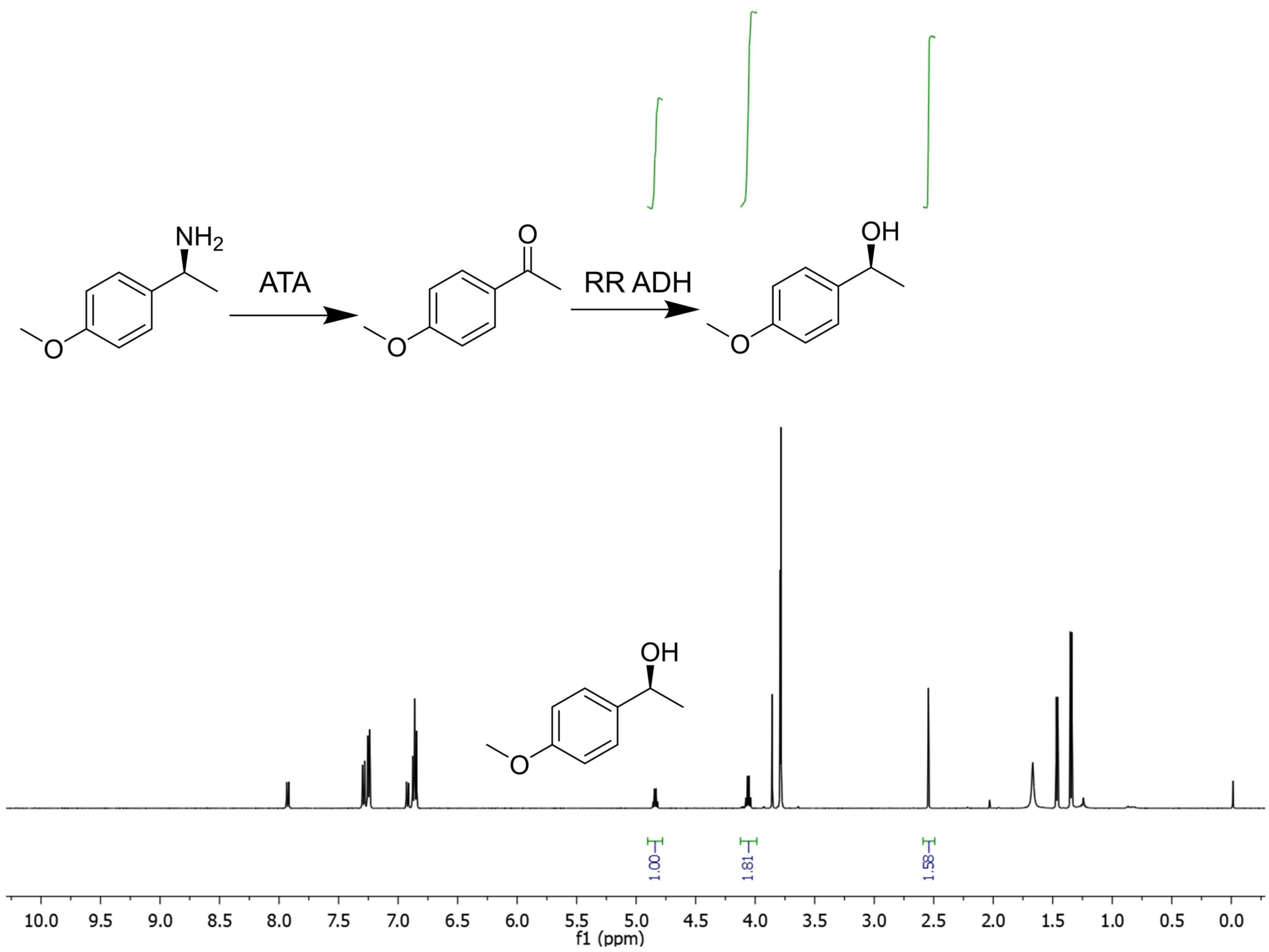

Supplementary Figure 39. ^{1}H NMR spectrum of crude 1-(4-methoxyphenyl)ethanol, synthesized by cascade catalysis in *E. coli* overexpressing ATA and RR ADH.

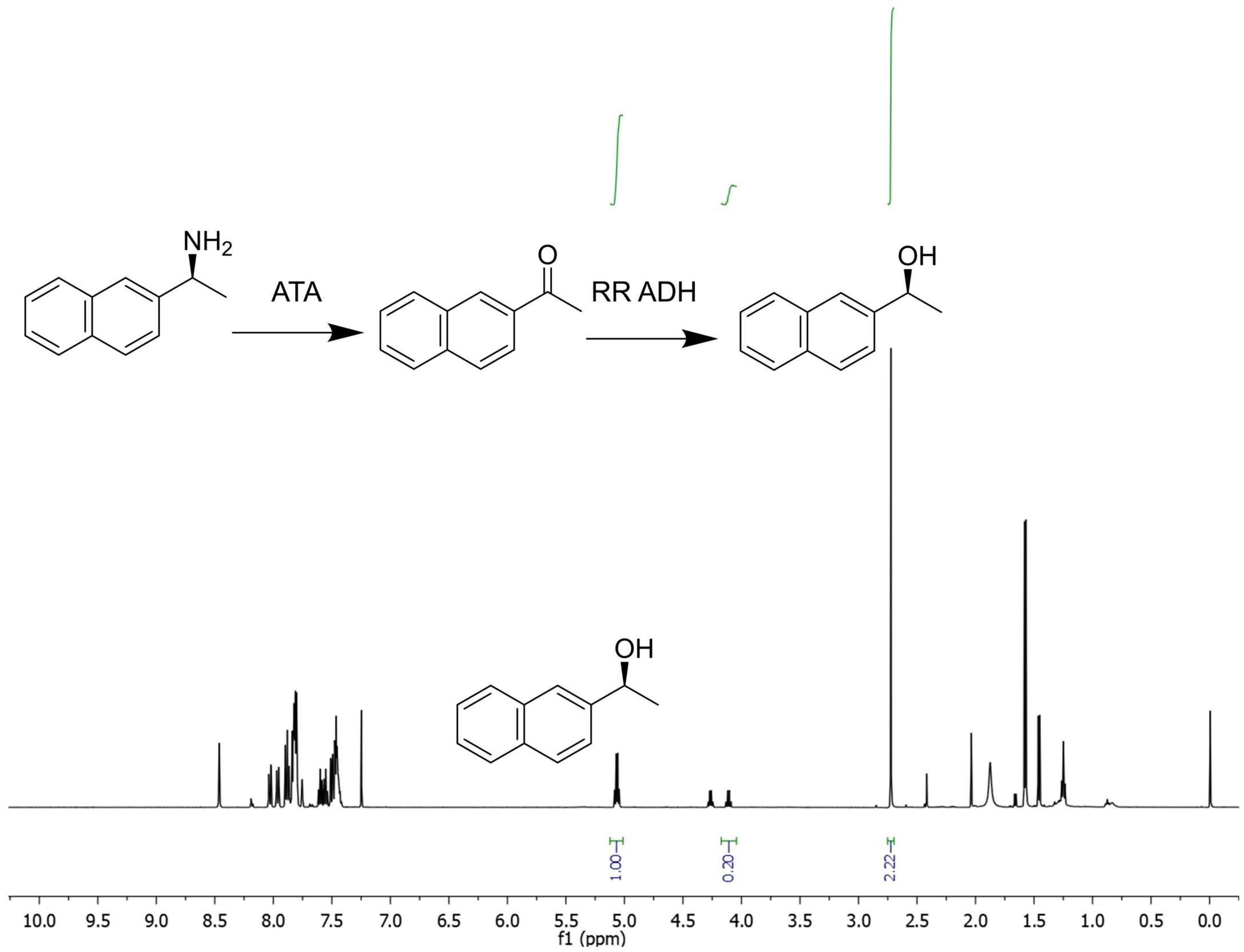

Supplementary Figure 40. ^{1}H NMR spectrum of crude 1-(naphtyl)ethanol, synthesized by cascade catalysis in *E. coli* overexpressing ATA and RR ADH.

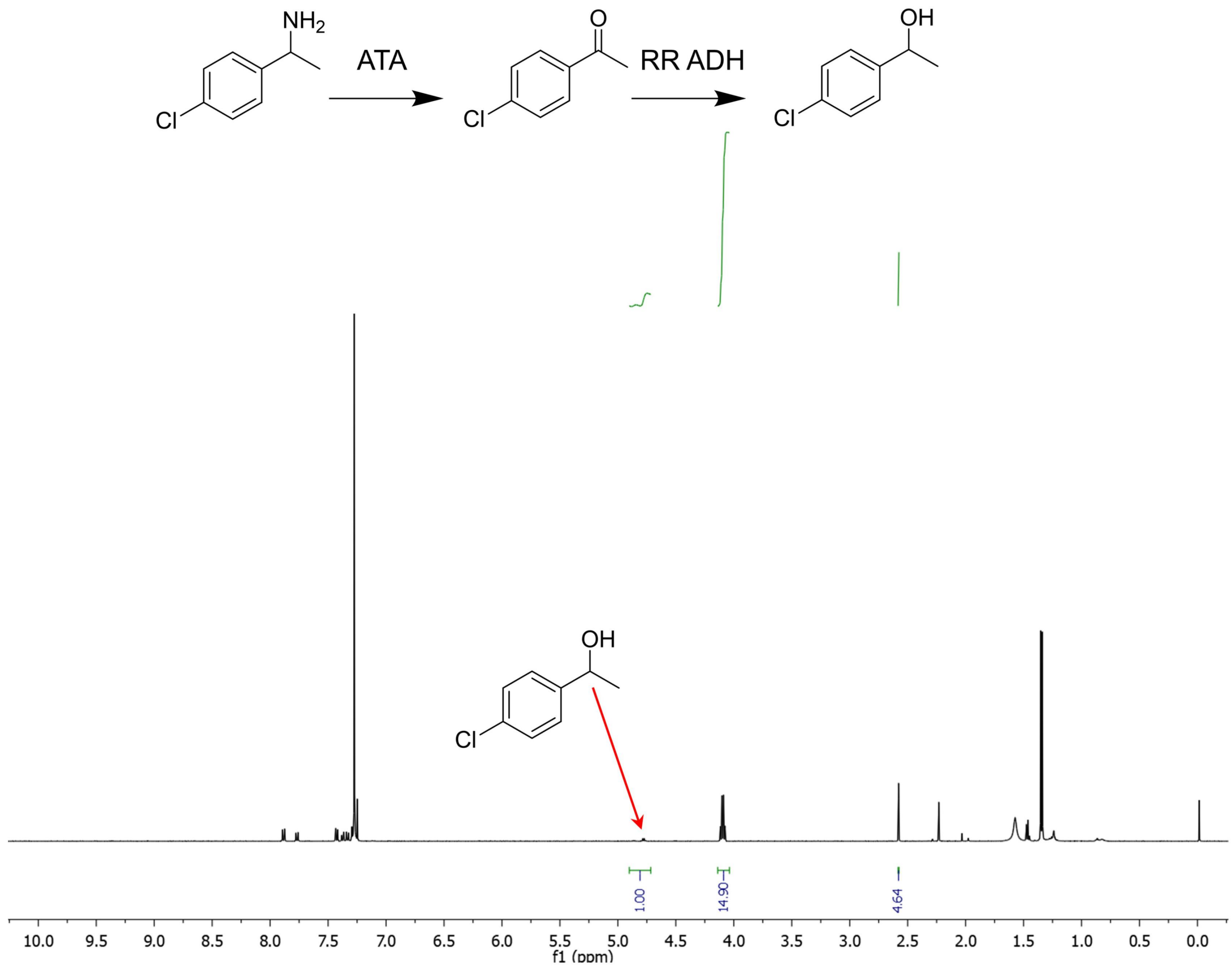

Supplementary Figure 41. ^{1}H NMR spectrum of crude 1-(4-chlorophenyl)ethanol, synthesized by cascade catalysis in *E. coli* overexpressing ATA and RR ADH.

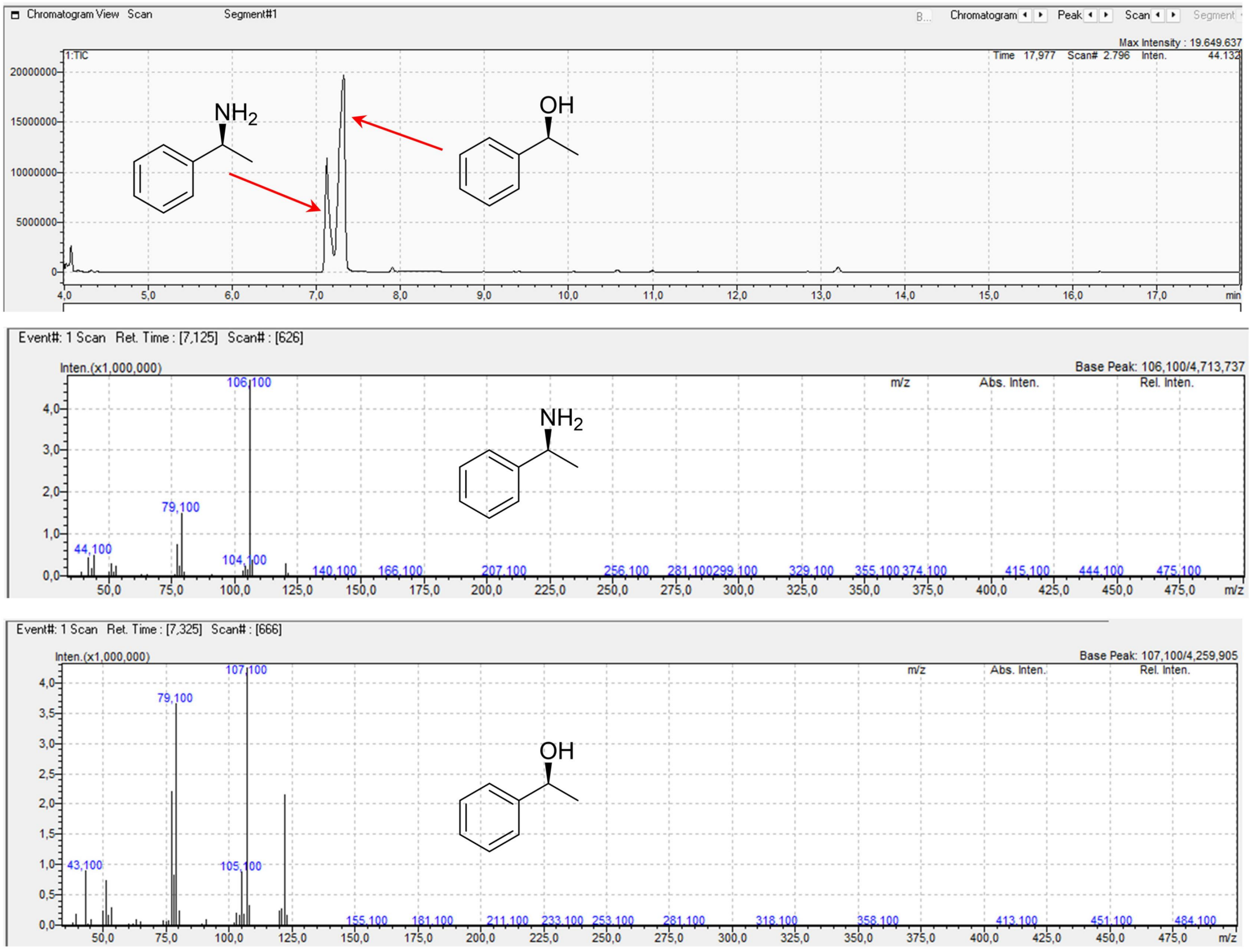

Supplementary Figure 42. GC and MS spectra of cascade catalysis from (*S*)-1-phenylethylamine to (*S*)-1-phenylethanol.

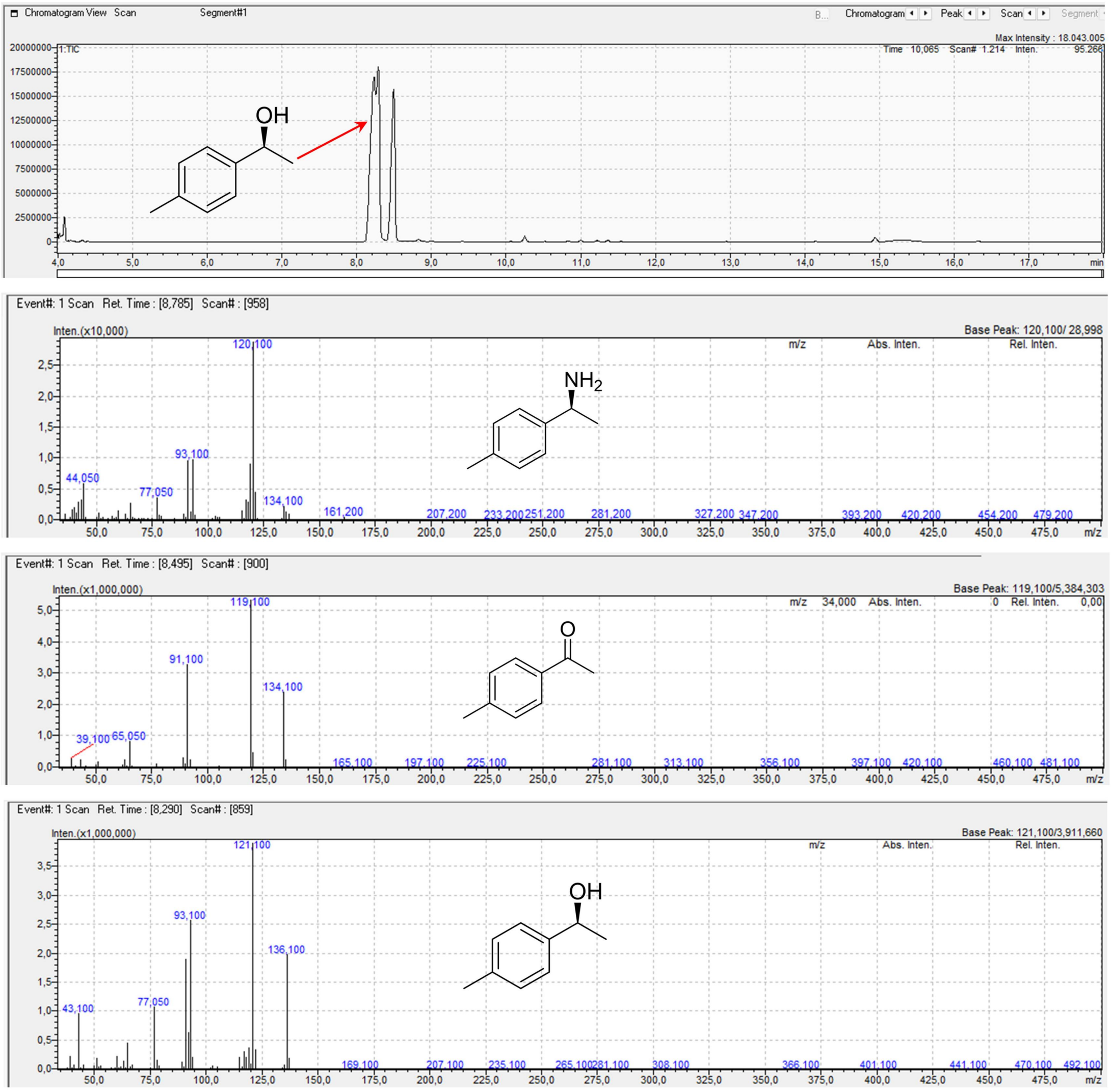

Supplementary Figure 43. GC and MS spectra of cascade catalysis from (*S*)-(-)-1-(p-tolyl)ethylamine to (*S*)-1-(m-tolyl)ethanol.

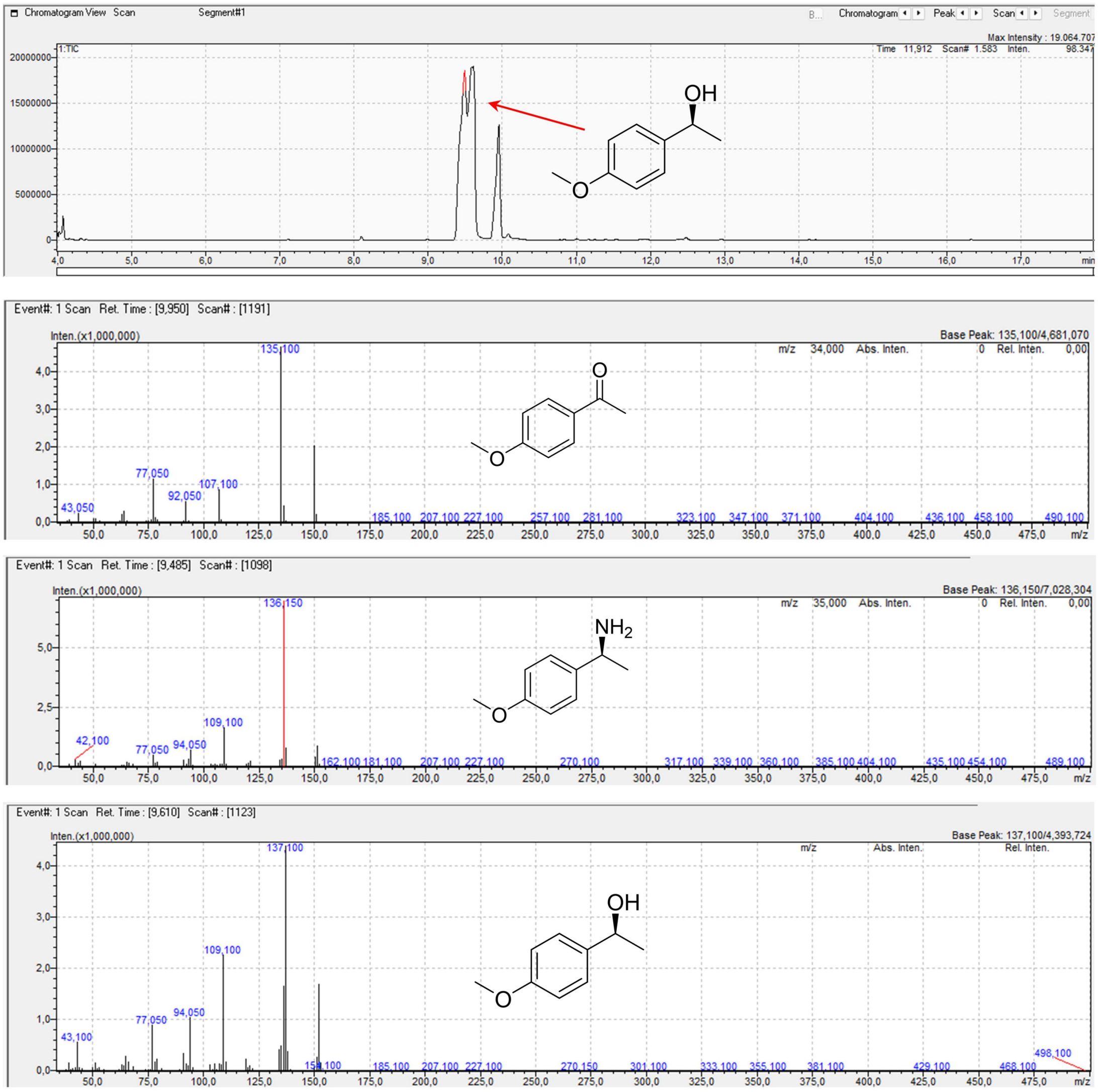

Supplementary Figure 44. GC and MS spectra of cascade catalysis from (*S*)-1-(4-methoxyphenyl)ethylamine to (*S*)-1-(4-methoxyphenyl)ethanol.

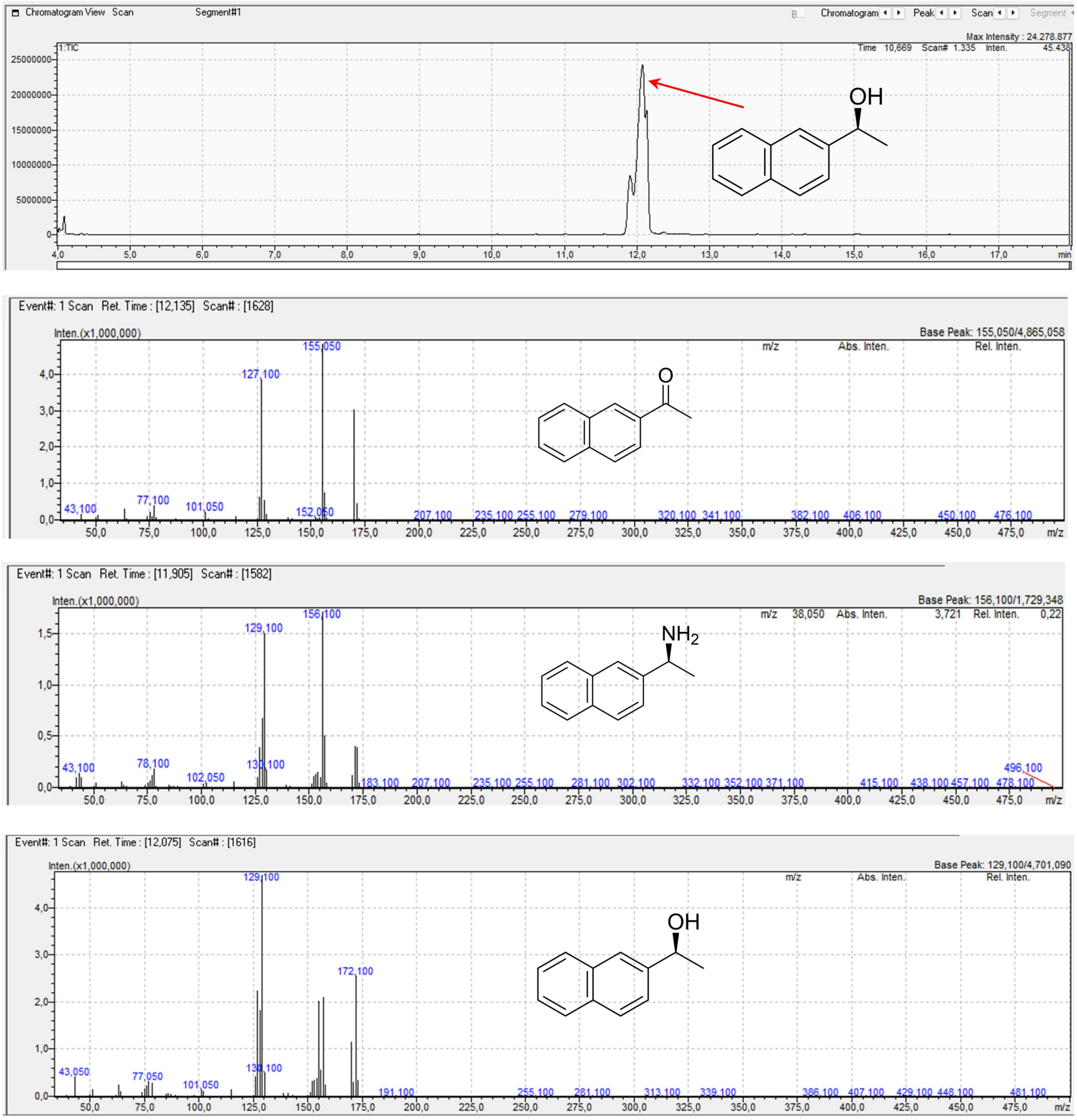

Supplementary Figure 45. GC and MS spectra of cascade catalysis from (*S*)-1-(naphthalen-2-yl)ethanamine to (*S*)-1-(naphthalen-2-yl)ethanol.

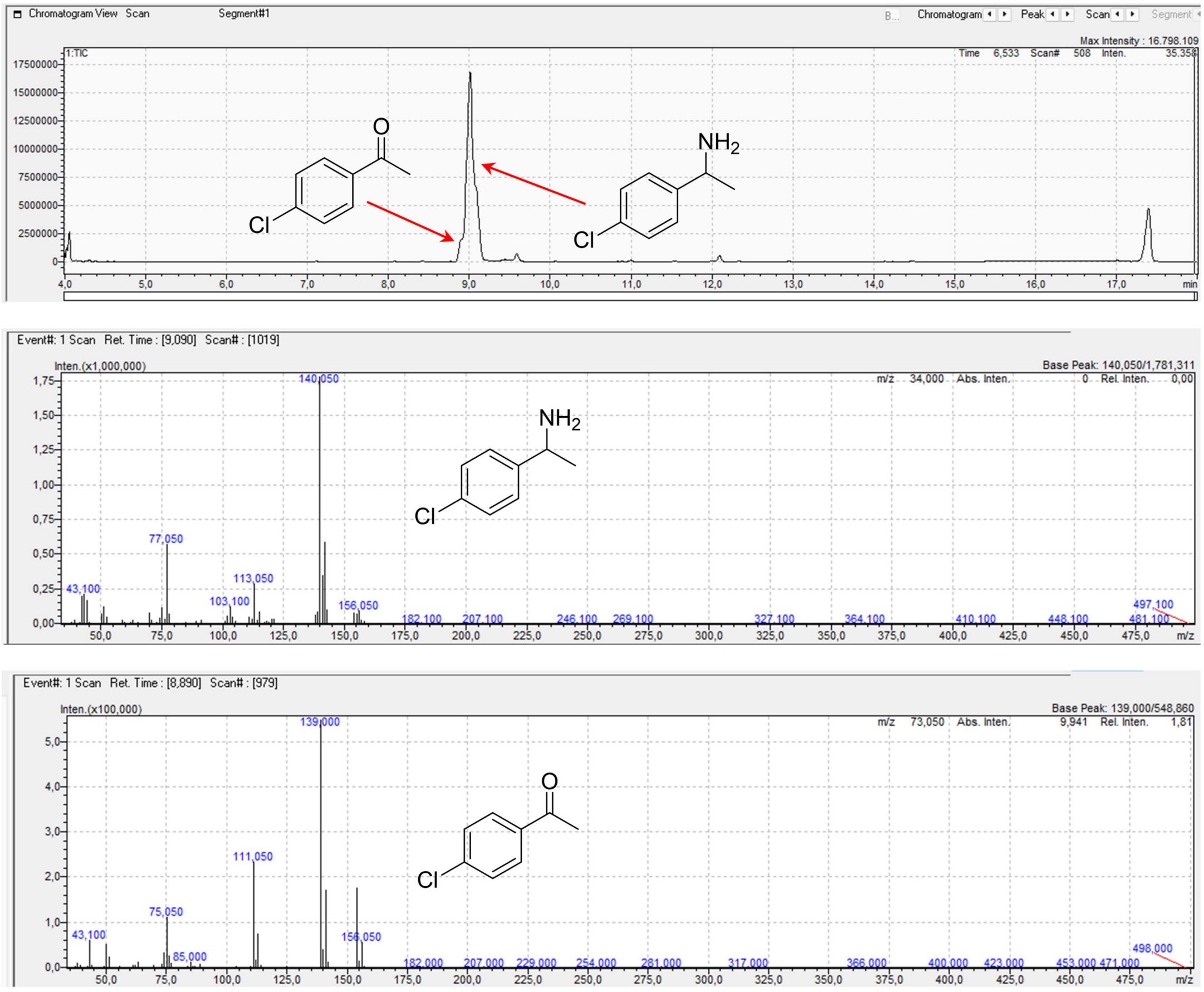

Supplementary Figure 46. GC and MS spectra of cascade catalysis from 1-(4-chlorophenyl)ethylamine to 1-(4-chlorophenyl)ethanol.

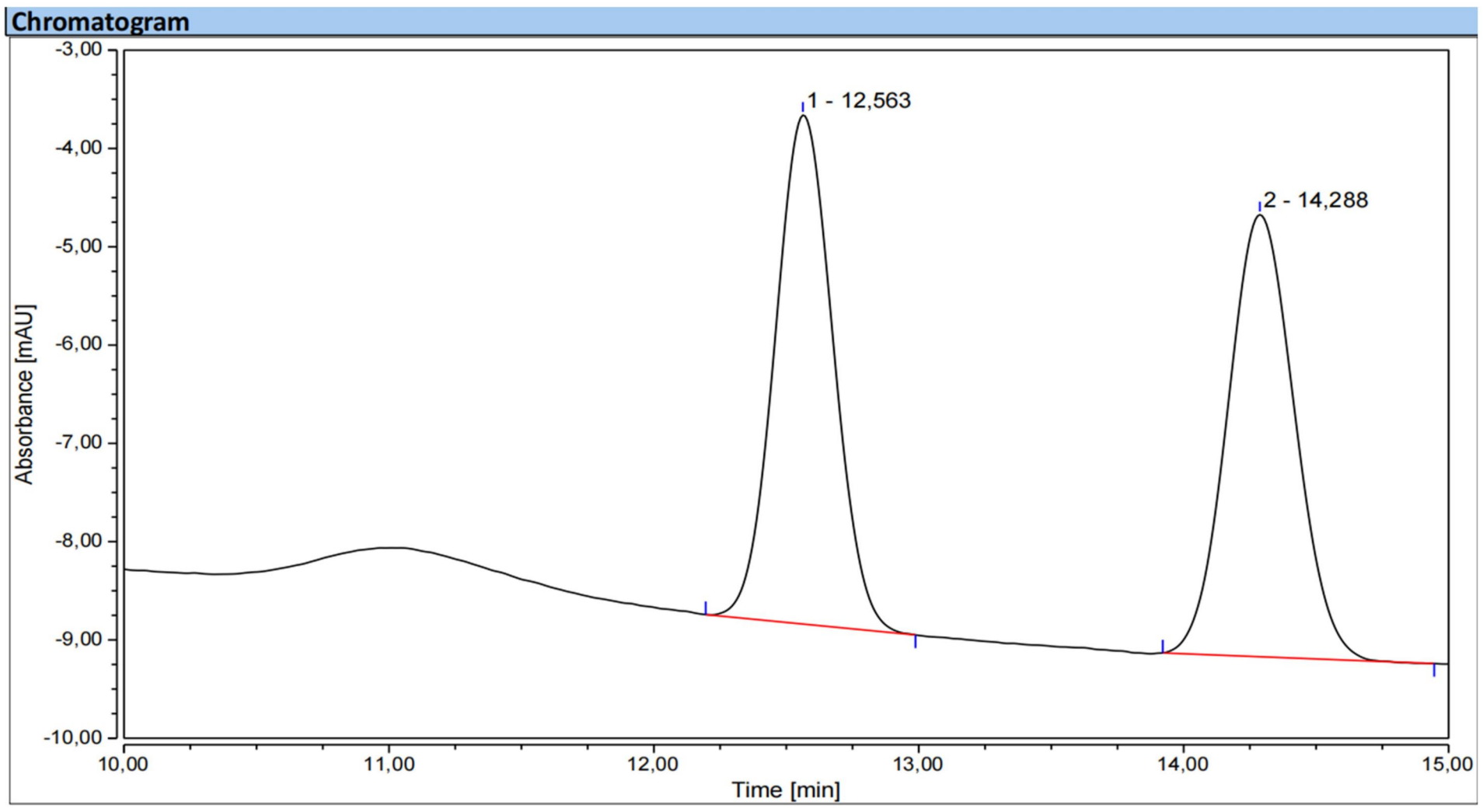

Integration Results

No.	Peakname	RetentionTime min	Area mAU*min	Height mAU	Relative Area %	Rel.Height %	Amount n.a.
1		12,563	1,3189	5,178	50,03	53,52	n.a.
2		14,288	1,3173	4,497	49,97	46,48	n.a.
**Total:**			**2,636**	**9,675**	**100,000**	**100,000**	

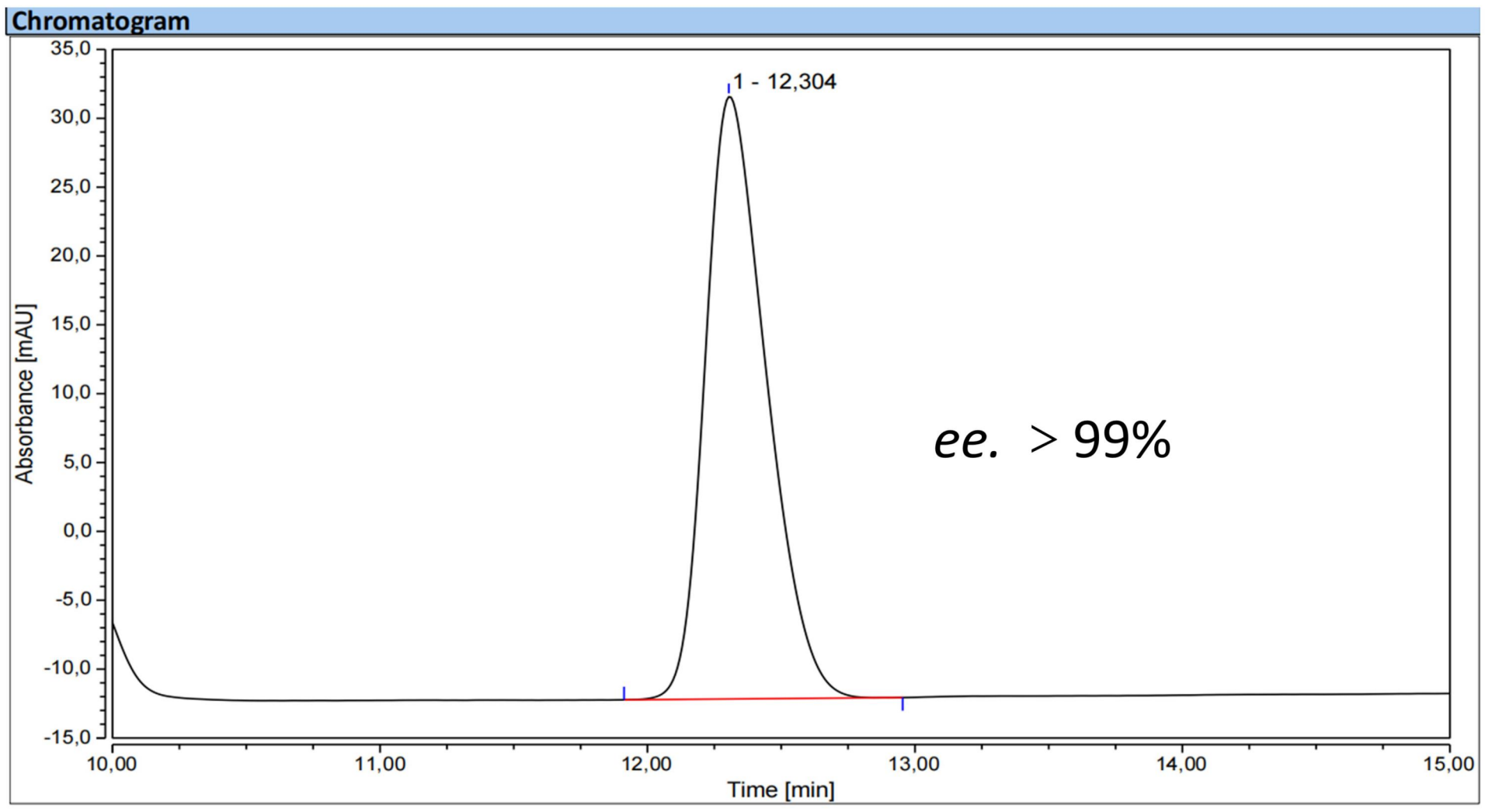

Integration Results

No.	Peakname	RetentionTime min	Area mAU*min	Height mAU	Relative Area %	Rel.Height %	Amount n.a.
1		12,304	11,5886	43,740	100,00	100,00	n.a.
**Total:**			**11,589**	**43,740**	**100,000**	**100,000**	

Supplementary Figure 47. Chiral HPLC chromatogram of crude (*S*)-1-phenylethanol (standard, upper).

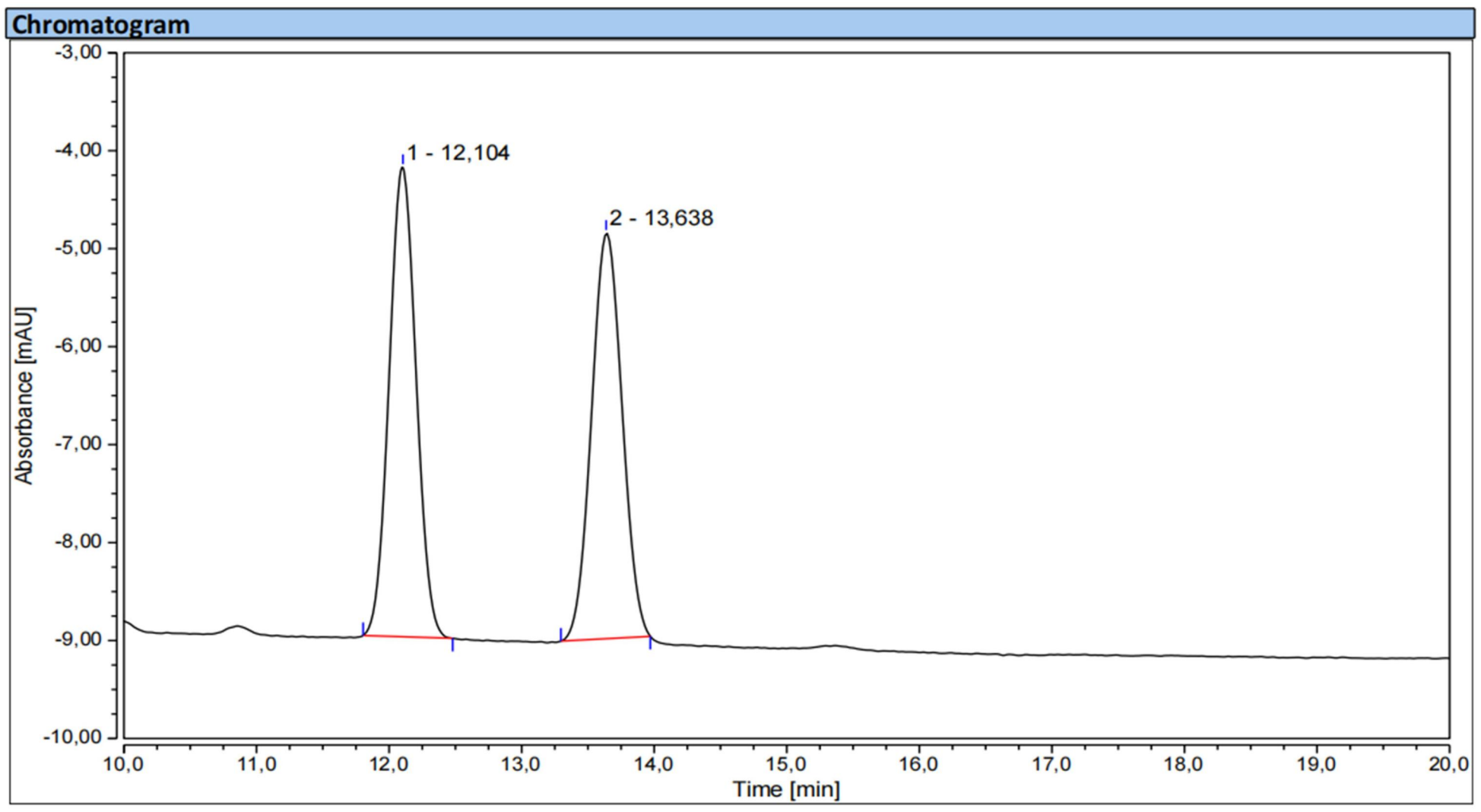

Integration Results

No.	Peakname	RetentionTime min	Area mAU*min	Height mAU	Relative Area %	Rel.Height %	Amount n.a.
1		12,104	1,1276	4,790	50,48	53,65	n.a.
2		13,638	1,1063	4,138	49,52	46,35	n.a.
**Total:**			**2,234**	**8,928**	**100,000**	**100,000**	

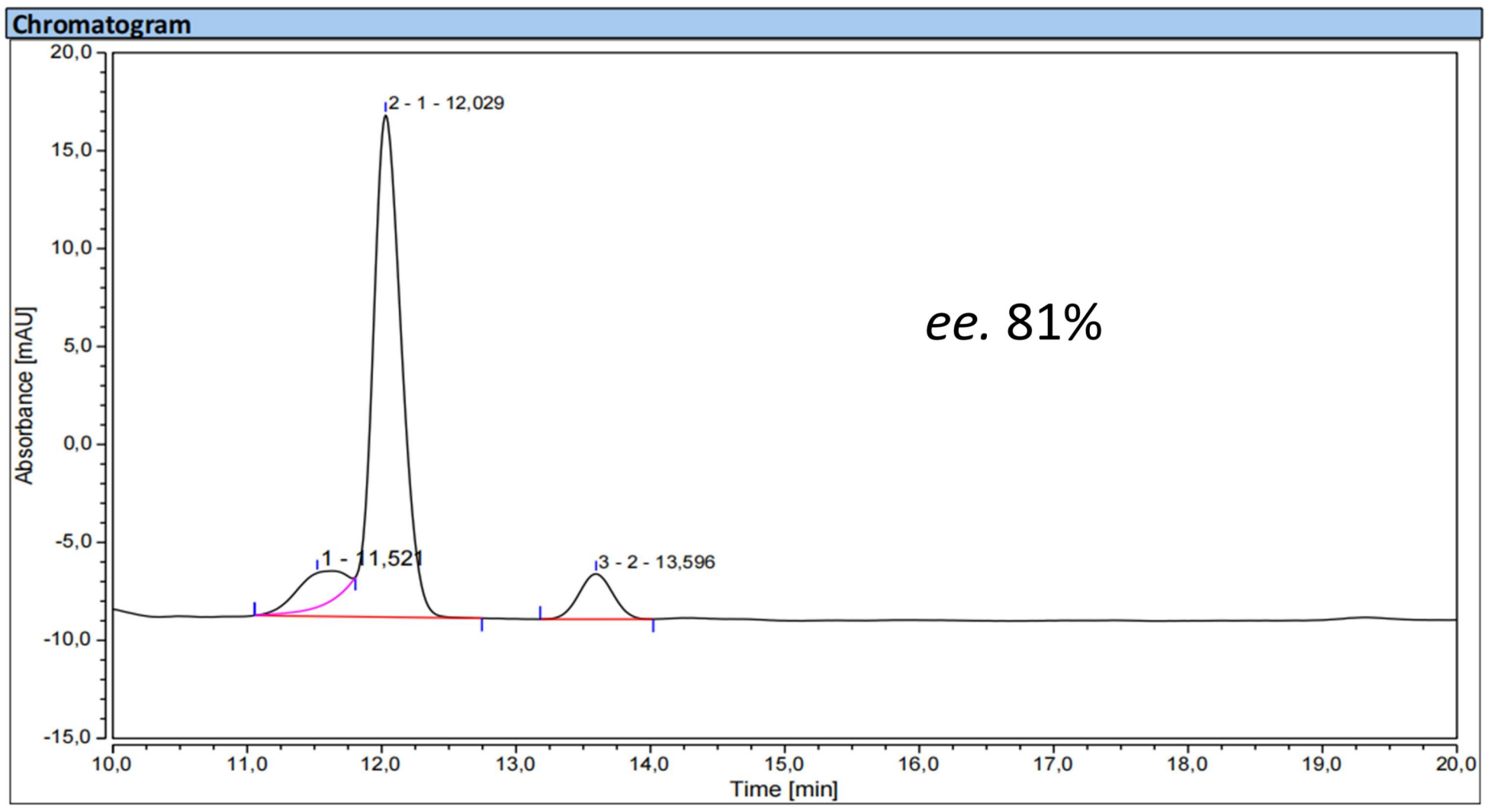

Integration Results

No.	Peakname	RetentionTime min	Area mAU*min	Height mAU	Relative Area %	Rel.Height %	Amount
1		11,521	0,6594	1,744	8,23	5,88	n.a.
2	1	12,029	6,6732	25,623	83,30	86,34	n.a.
3	2	13,596	0,6784	2,311	8,47	7,79	n.a.
**Total:**			**8,011**	**29,678**	**100,000**	**100,000**	

Supplementary Figure 48. Chiral HPLC chromatogram of crude (*S*)-1-(m-tolyl)ethanol (standard, upper).

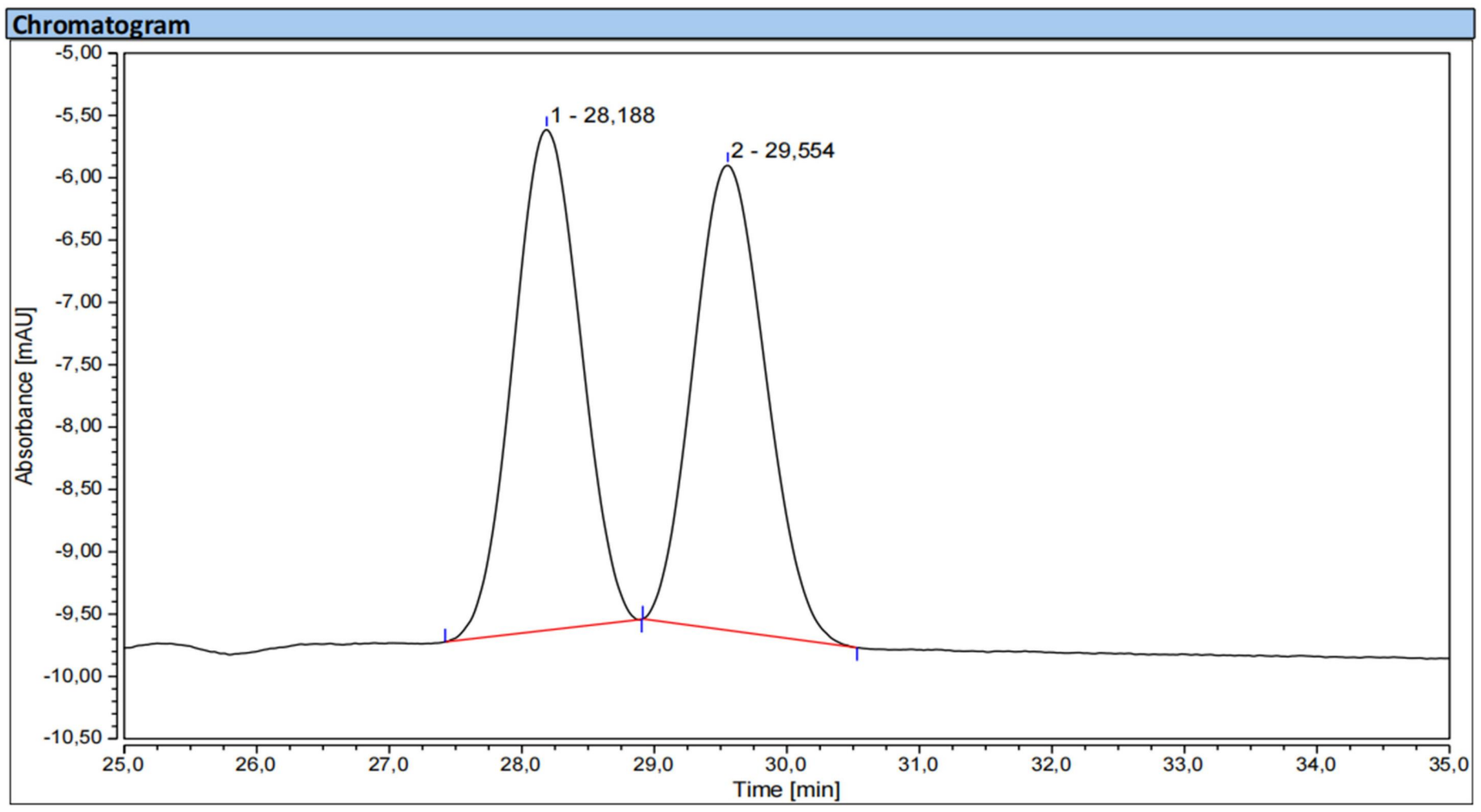

Integration Results

No.	Peakname	RetentionTime min	Area mAU*min	Height mAU	Relative Area %	Rel.Height %	Amount n.a.
1		28,188	2,3666	4,016	50,14	51,85	n.a.
2		29,554	2,3538	3,729	49,86	48,15	n.a.
**Total:**			**4,720**	**7,745**	**100,000**	**100,000**	

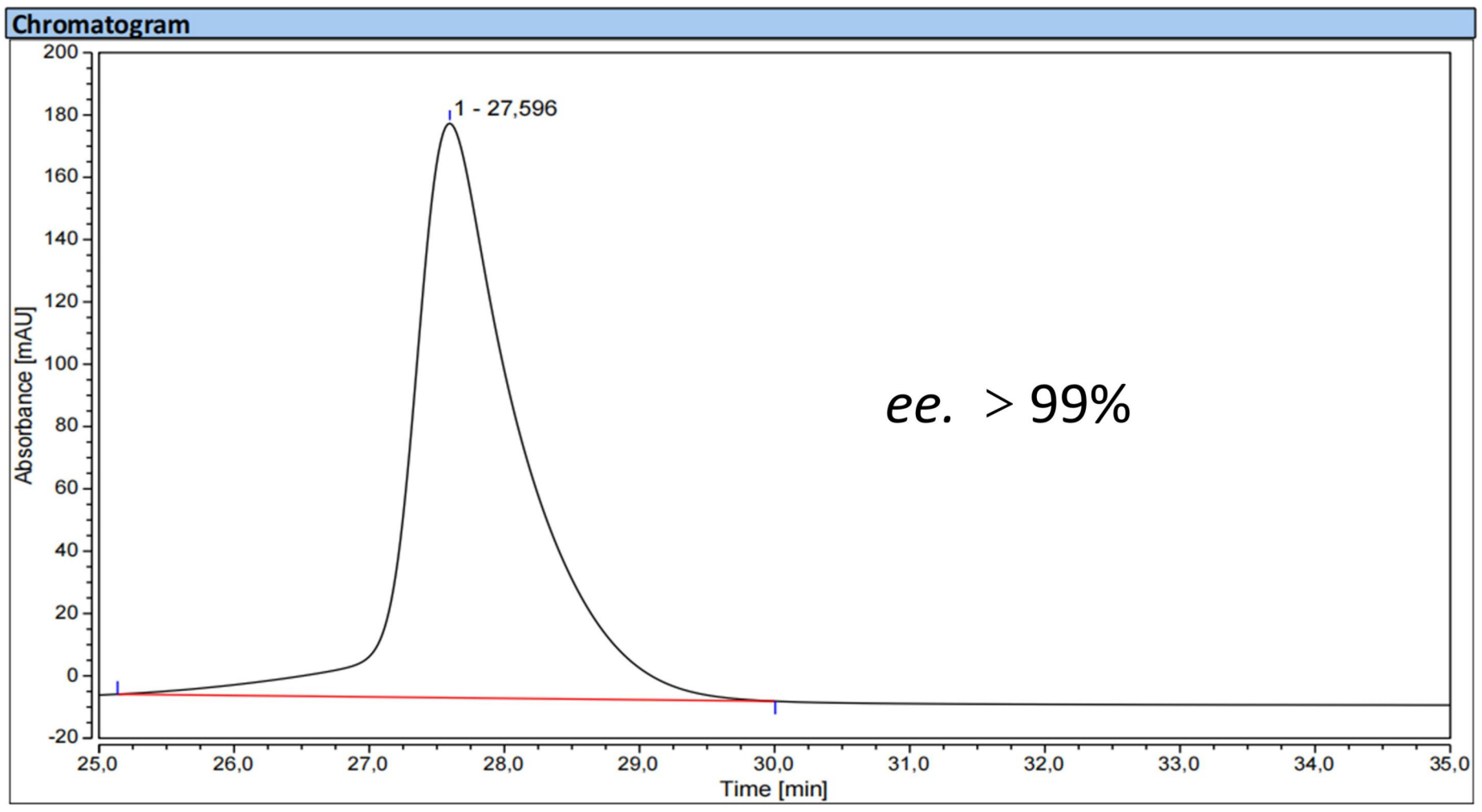

Integration Results

No.	Peakname	RetentionTime min	Area mAU*min	Height mAU	Relative Area %	Rel.Height %	Amount n.a.
1		27,596	169,4502	184,340	100,00	100,00	n.a.
**Total:**			**169,450**	**184,340**	**100,000**	**100,000**	

Supplementary Figure 49. Chiral HPLC chromatogram of crude (*S*)-1-(4-methoxyphenyl)ethanol (standard, upper).

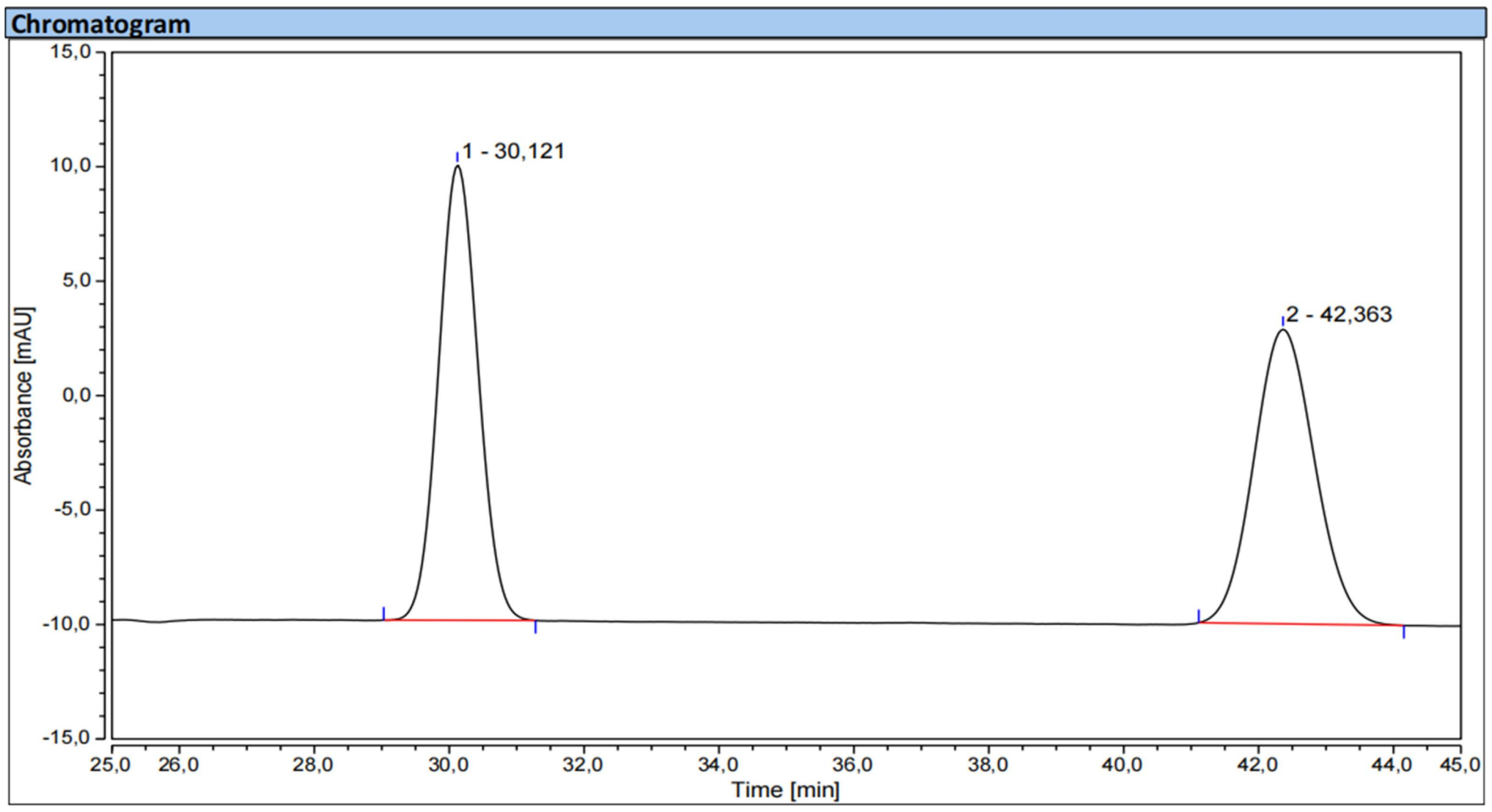

Integration Results

No.	Peakname	RetentionTime min	Area mAU*min	Height mAU	Relative Area %	Rel.Height %	Amount n.a.
1		30,121	13,5399	19,877	50,22	60,71	n.a.
2		42,363	13,4222	12,861	49,78	39,29	n.a.
**Total:**			**26,962**	**32,738**	**100,000**	**100,000**	

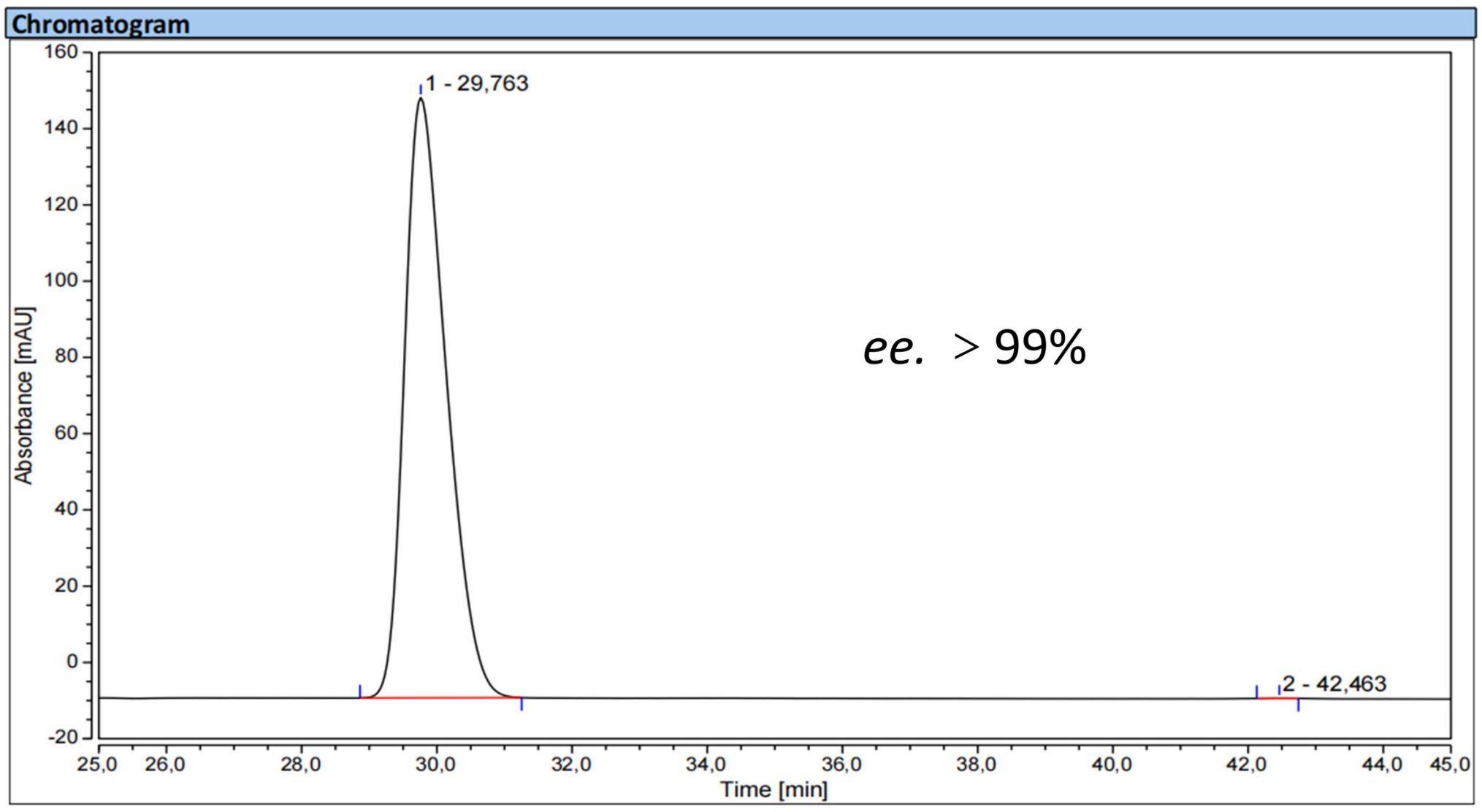

Integration Results

No.	Peakname	RetentionTime min	Area mAU*min	Height mAU	Relative Area %	Rel.Height %	Amount n.a.
1		29,763	112,5268	157,420	99,98	99,97	n.a.
2		42,463	0,0208	0,054	0,02	0,03	n.a.
**Total:**			**112,548**	**157,475**	**100,000**	**100,000**	

Supplementary Figure 50. Chiral HPLC chromatogram of crude (*S*)-1-(naphthalen-2-yl)ethanol (standard, upper).

Chromatogram

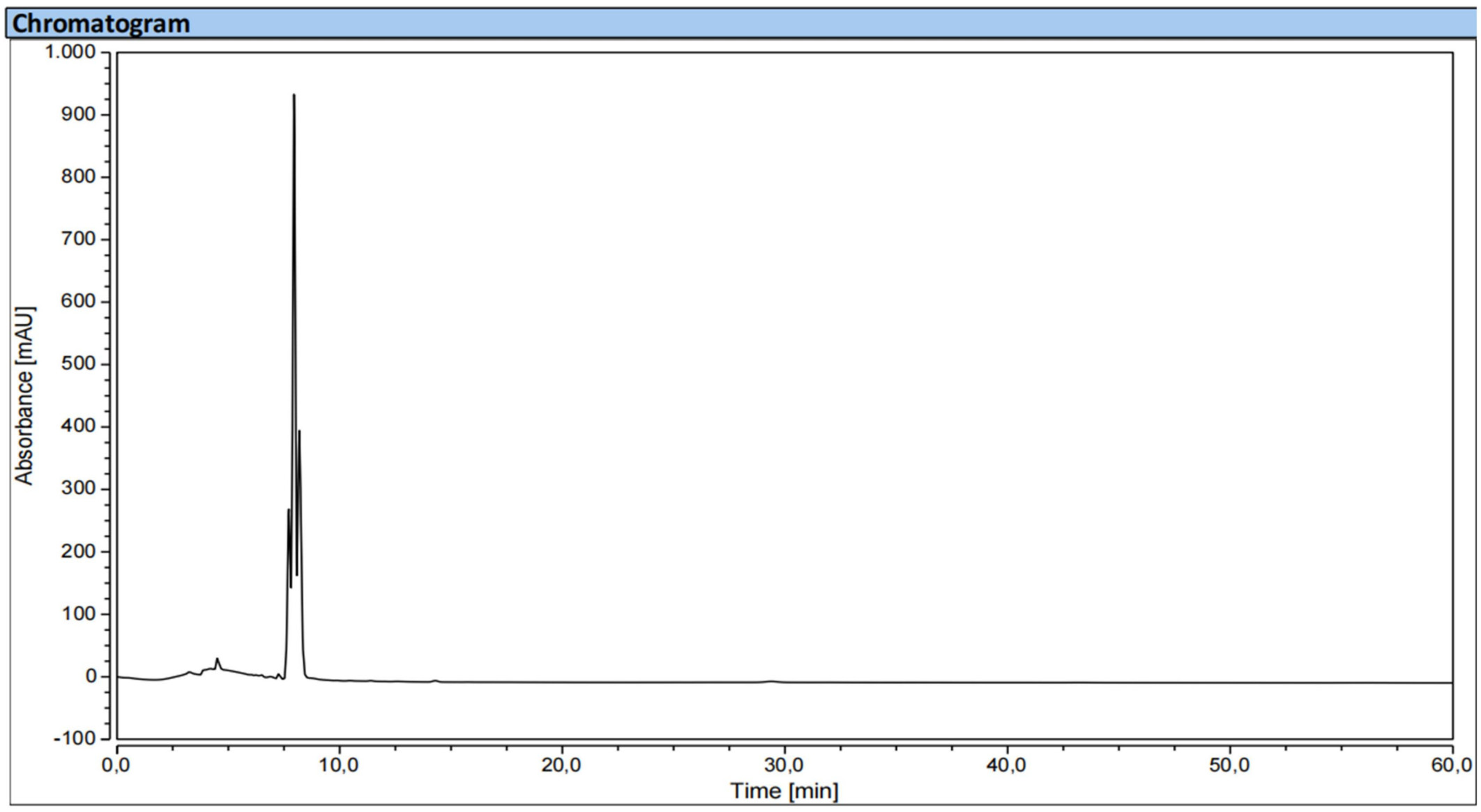

Integration Results

No.	Peakname	RetentionTime min	Area mAU*min	Height mAU	Relative Area %	Rel.Height %	Amount n.a.
**Total:**			**0,000**	**0,000**	**0,000**	**0,000**	

Supplementary Figure 51. Chiral HPLC chromatogram of crude 1-(4-chlorophenyl)ethanol.

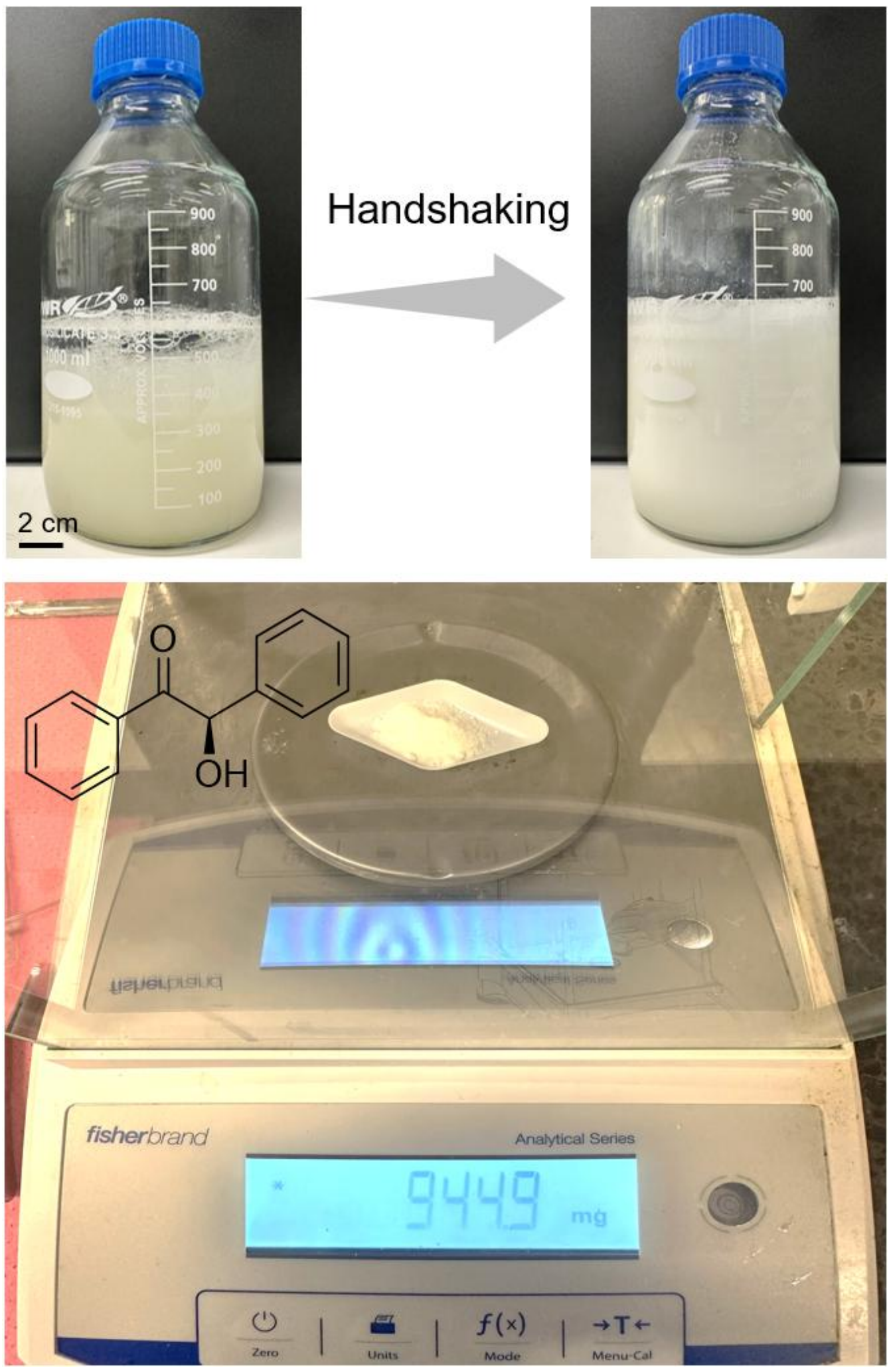

Supplementary Figure 52. A 600mL pre-scale-up experiment was conducted. Approximately 1 g of benzoin was ultimately obtained, providing reliable evidence of the platform's industrialization potential.

a

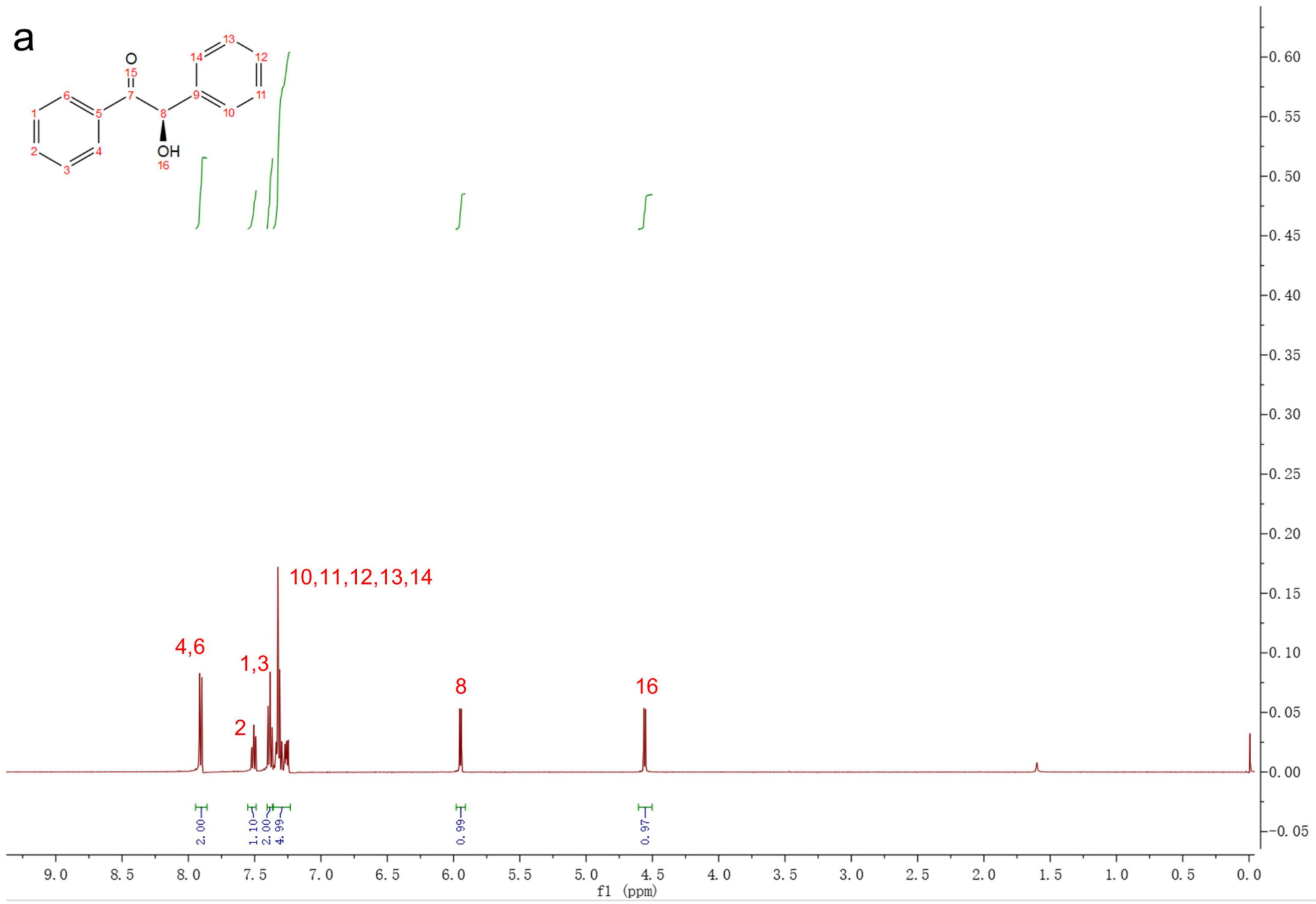

b

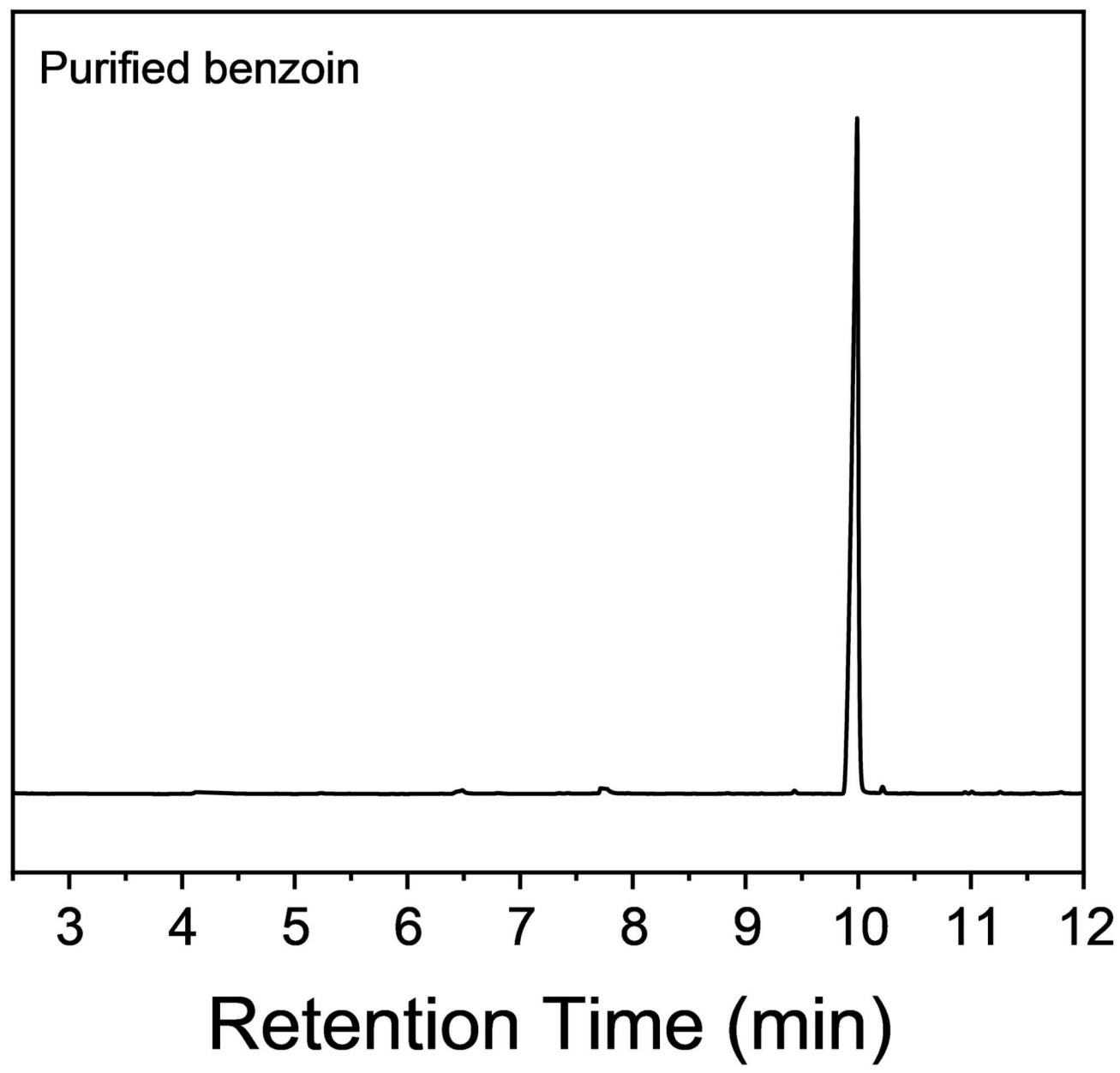

Supplementary Figure 53. (a) ^{1}H NMR spectrum of benzoin catalyzed by a scalable Pickering emulsion system. (b) GC spectrum of purified benzoin.

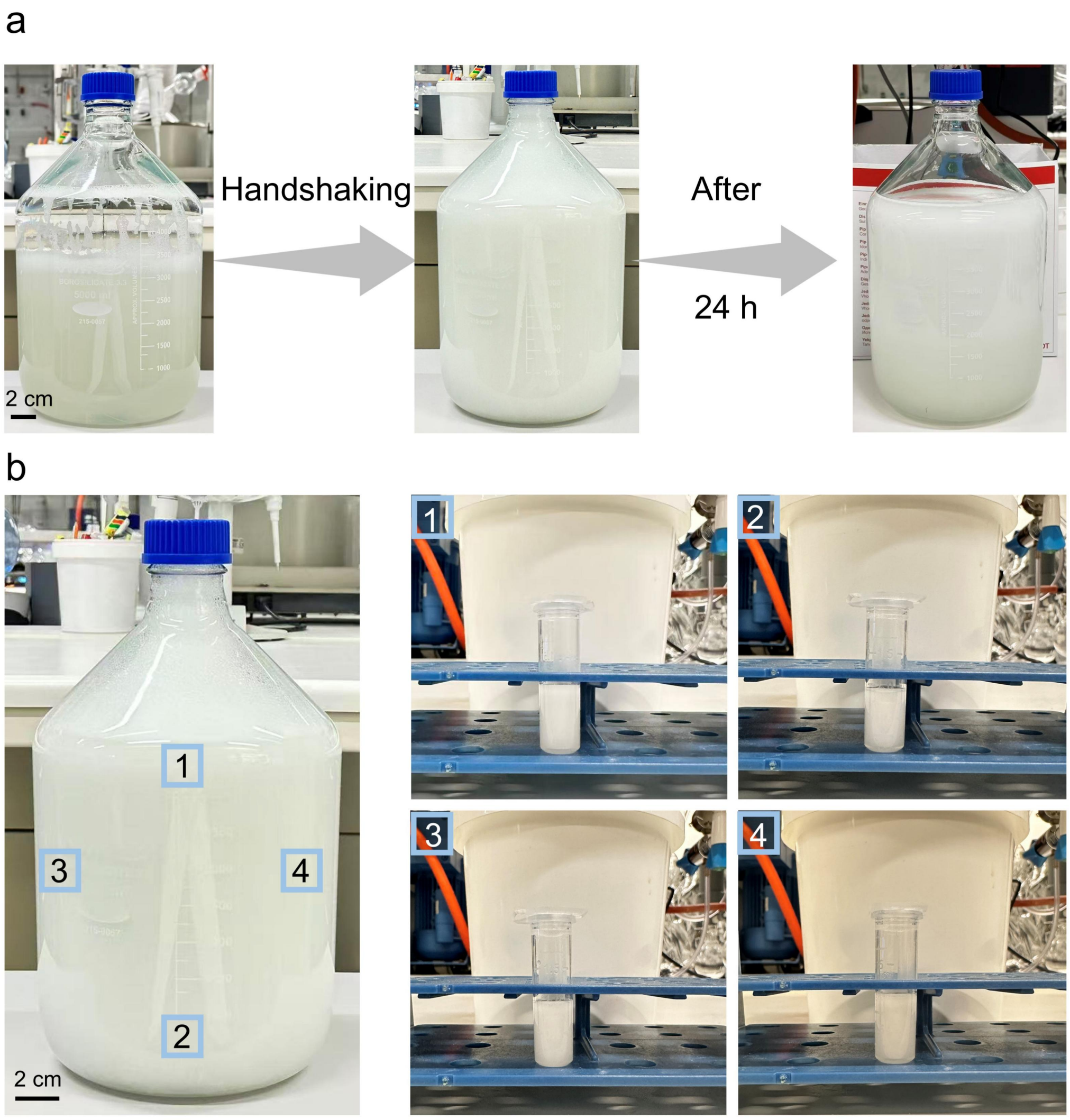

Supplementary Figure 54. (a) The 5 L scale-up experiment was further conducted, though it required a longer handshaking time. (b) Take 1 mL of emulsion from different positions within the 5 L emulsion system to observe the emulsion's spatial stability.

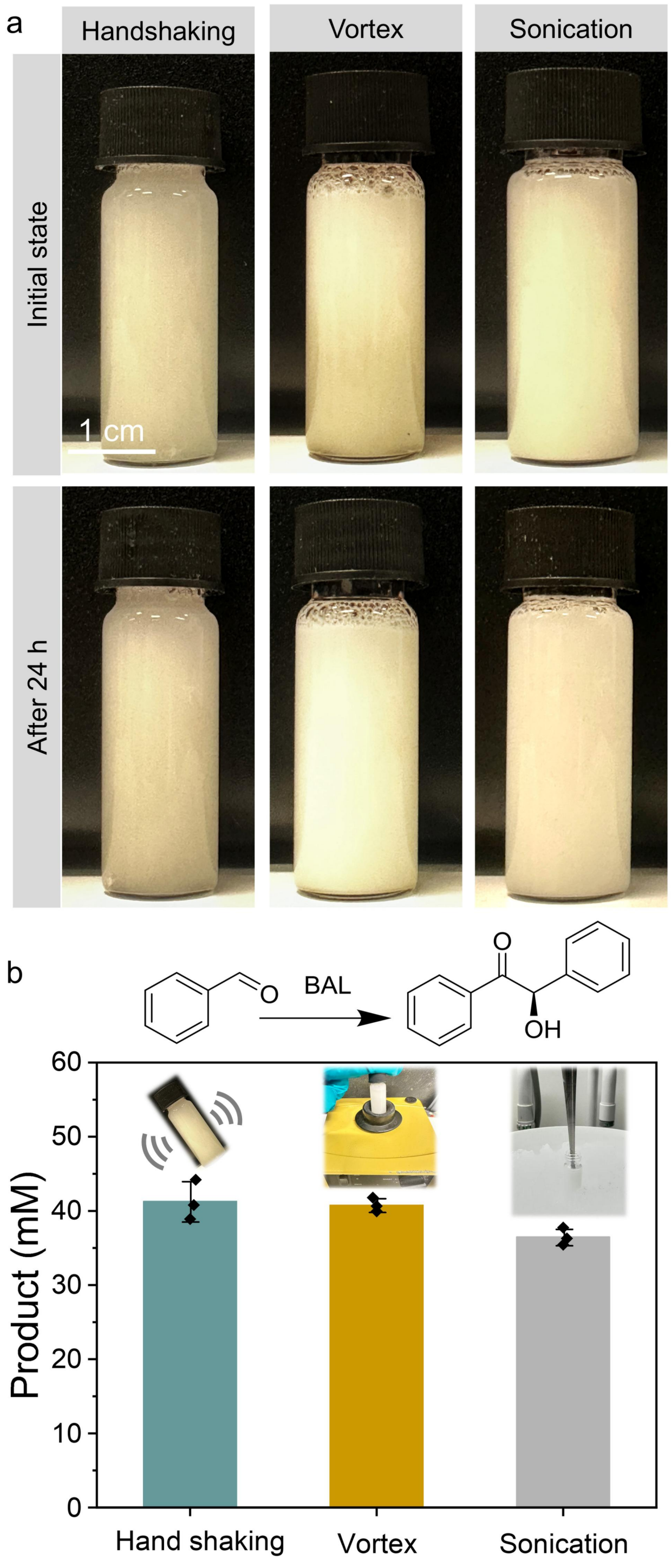

Supplementary Figure 55. (a) Whether using hand shaking, vortexing, or sonication treatment, robust Pickering emulsion systems can be obtained that remain stable for 24 hours. (b) Emulsions prepared by hand shaking, vortexing, and sonication exhibit consistent catalytic activity. Data are presented as mean values +/- SD based on n=3 independent replicates with the error bars representing the SD.

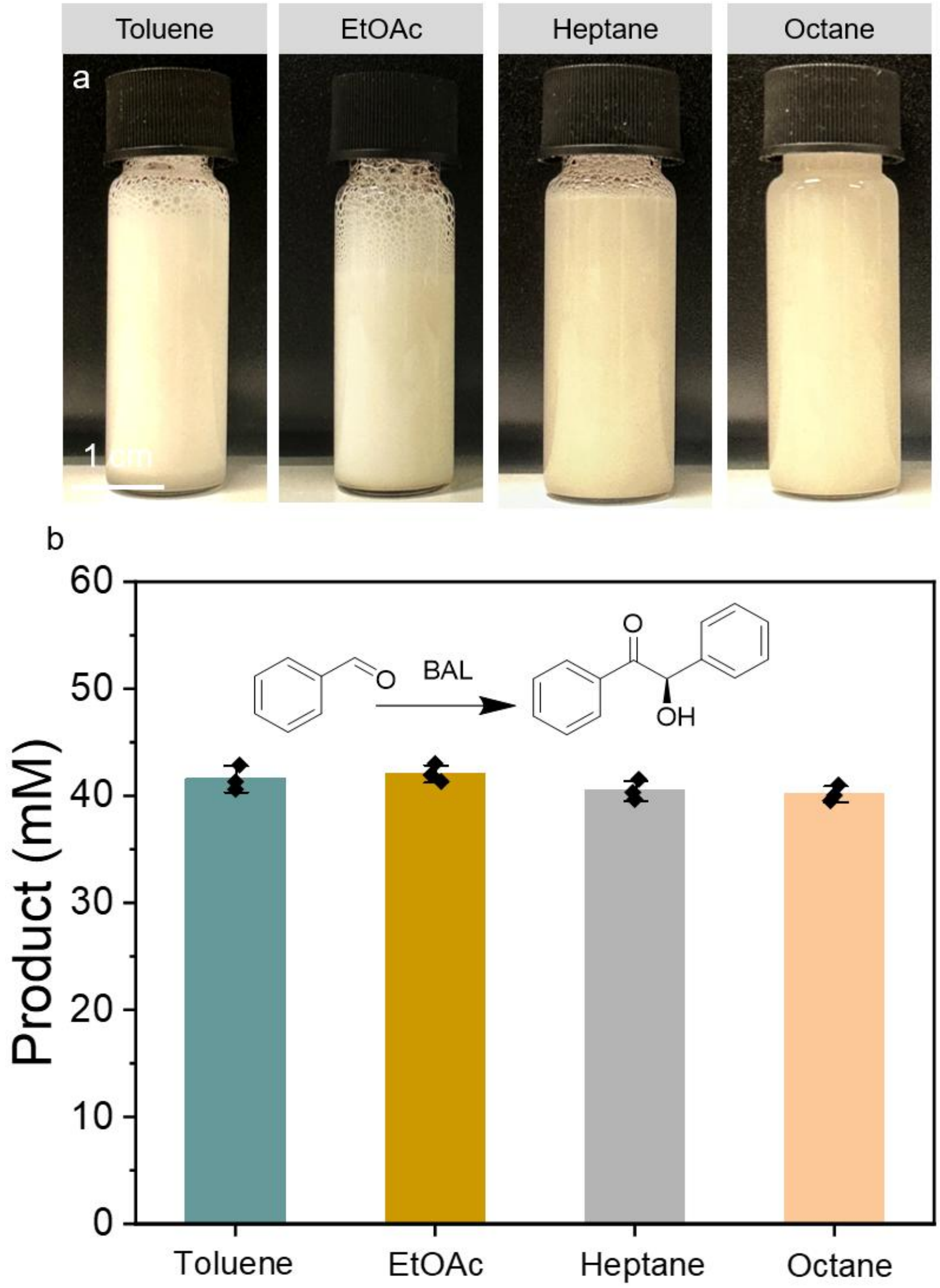

Supplementary Figure 56. (a) Three more eco-friendly solvents (ethyl acetate, heptane and octane) serve as reliable alternatives to toluene, producing emulsions with consistent stability. (b) Pickering emulsions prepared with four different organic phases showed no difference in catalytic activity. Data are presented as mean values +/- SD based on n=3 independent replicates with the error bars representing the SD.

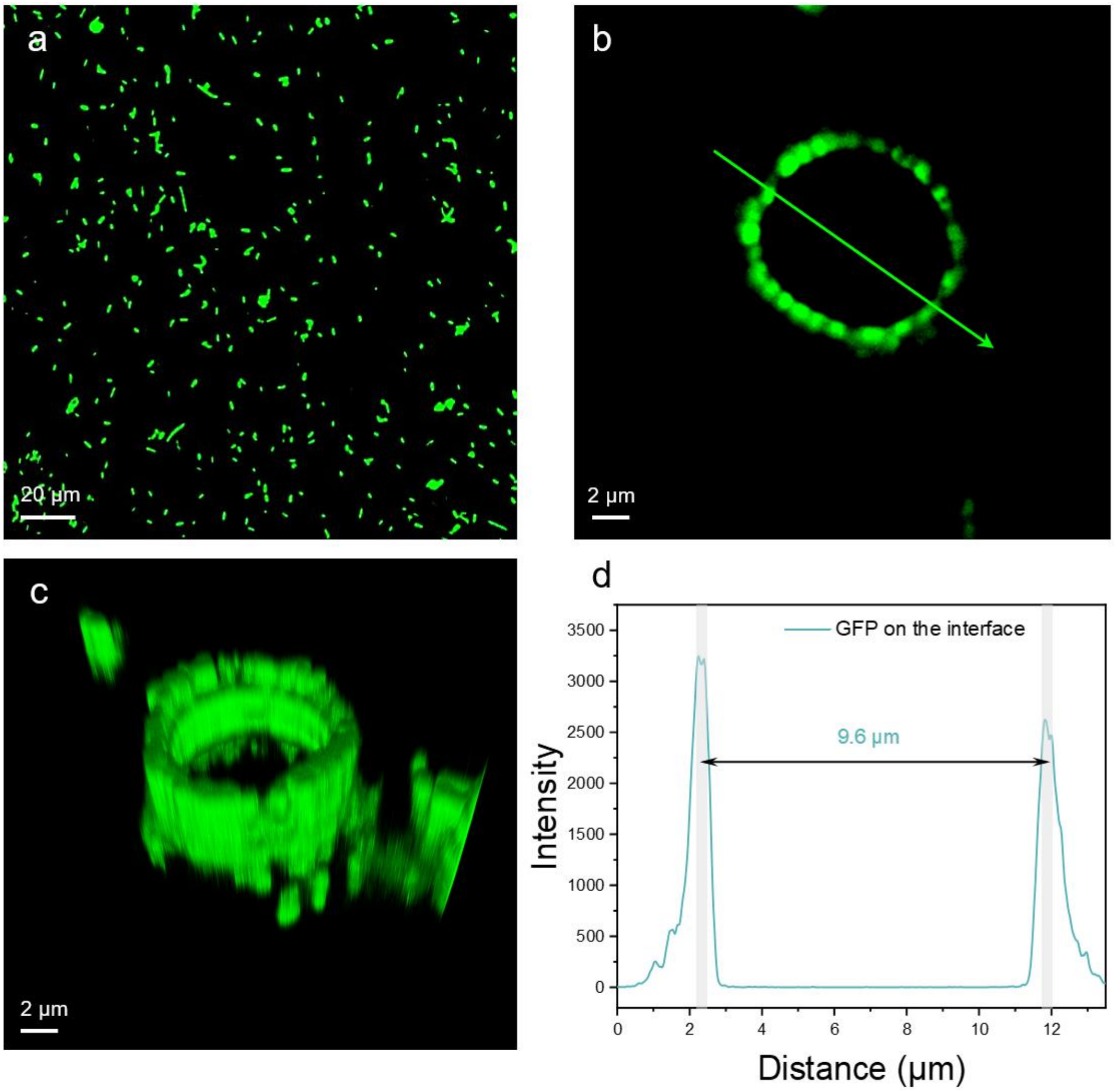

Supplementary Figure 57. (a) Cell@OBC from the emulsion collected by centrifugation were uniformly dispersed in the water phase, confirming the reversible attachment of cell@OBC achieved through handshaking and centrifugation. (b) 2D fluorescence image of GFP-overexpressing cell@OBC stable emulsion after five "centrifugation-emulsification" cycles. (c) The 3D cross-sectional fluorescence image of the stabilized emulsion formed by GFP-overexpressing cell@OBC after five "centrifugation-emulsification" cycles further confirms that cell@OBC are anchored at the water-oil interface. (d) After five "centrifugation-emulsification" cycles, the droplet size closely matches the freshly prepared emulsion. The experiment was repeated three times independently with similar results.

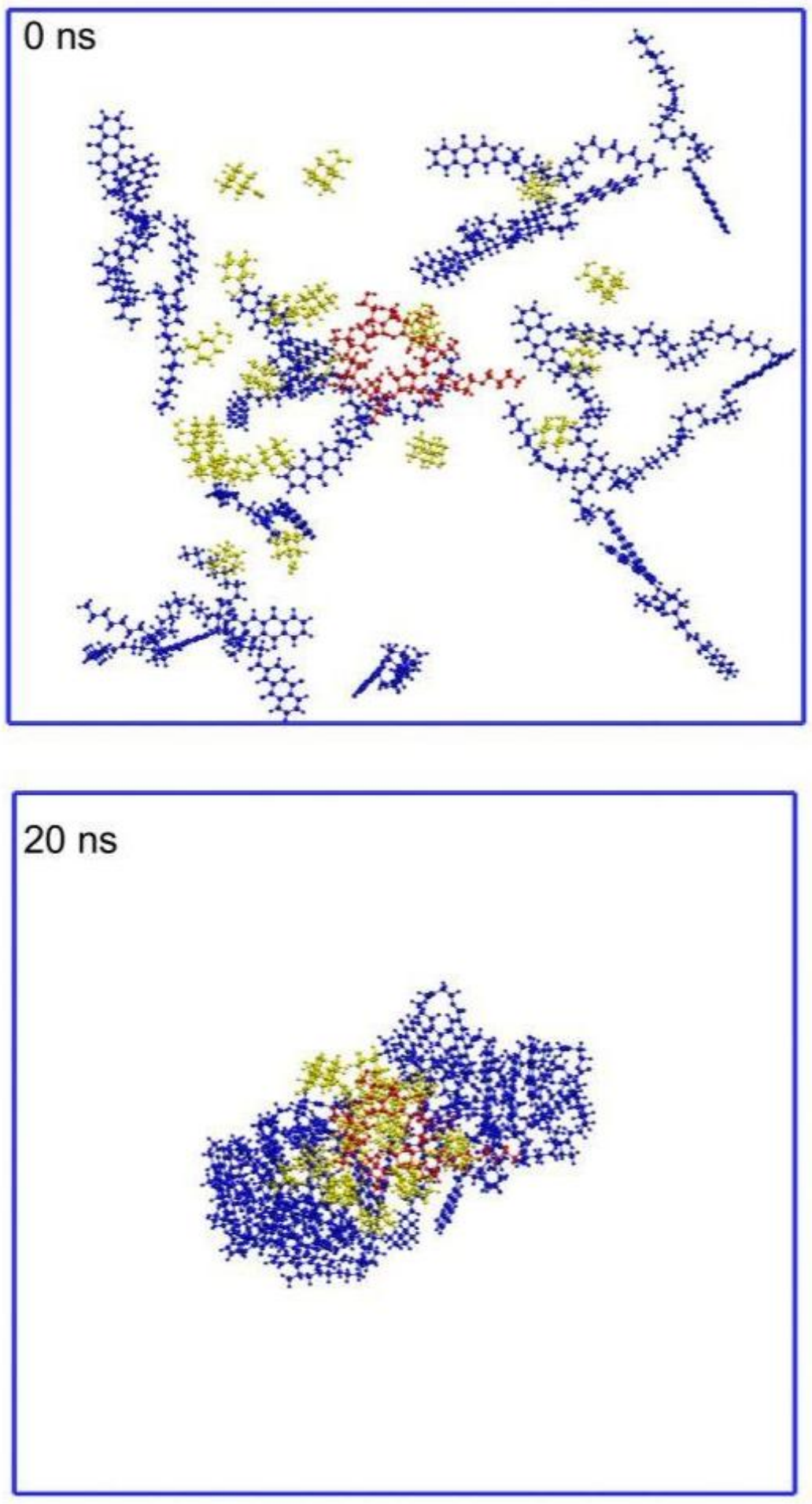

Supplementary Figure 58. Competitive interaction of OBC and adamantane with β-CD from initial state (0 ns) to steady state (20 ns).

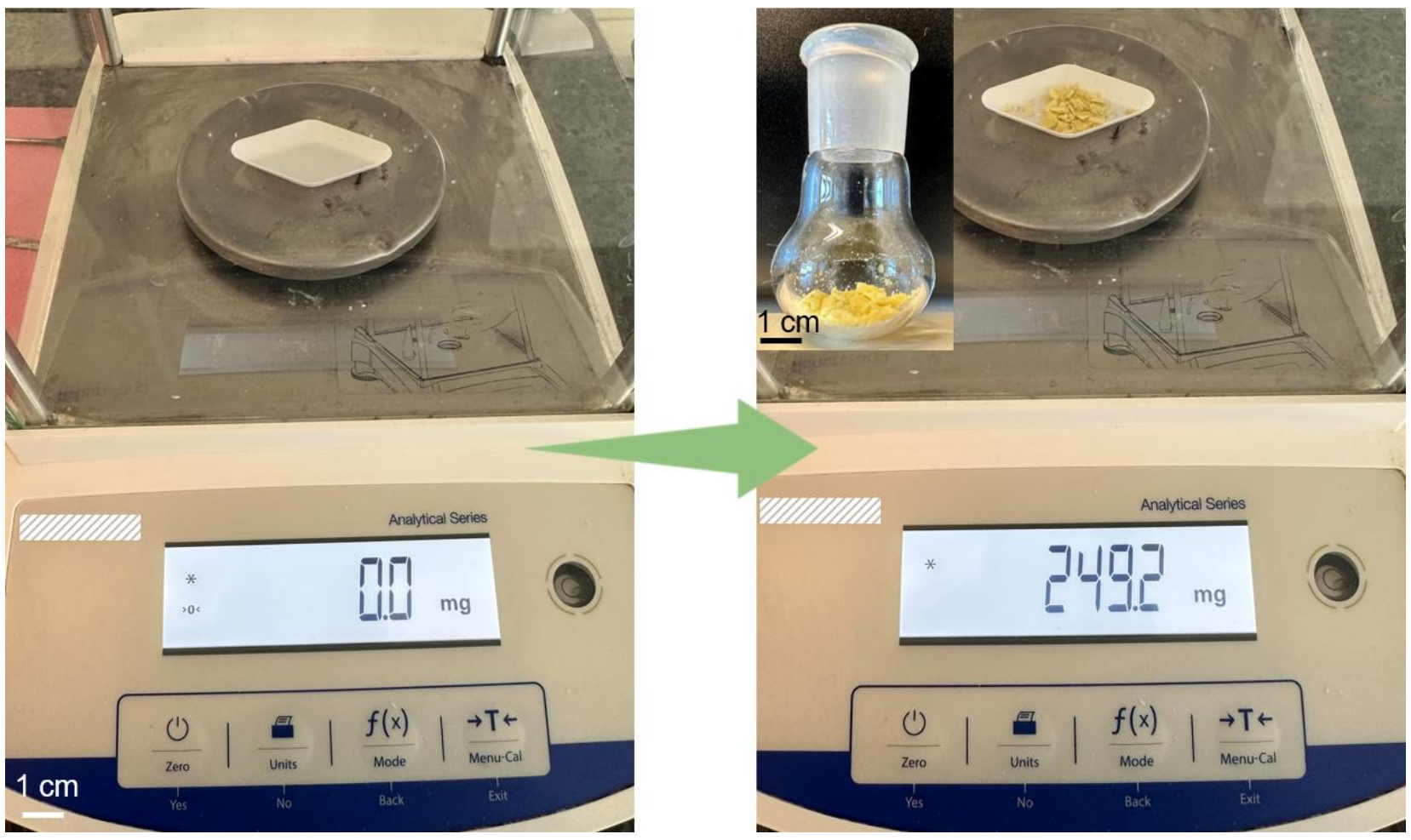

Supplementary Figure 59. Recovered oil-based catalyst (OBC).

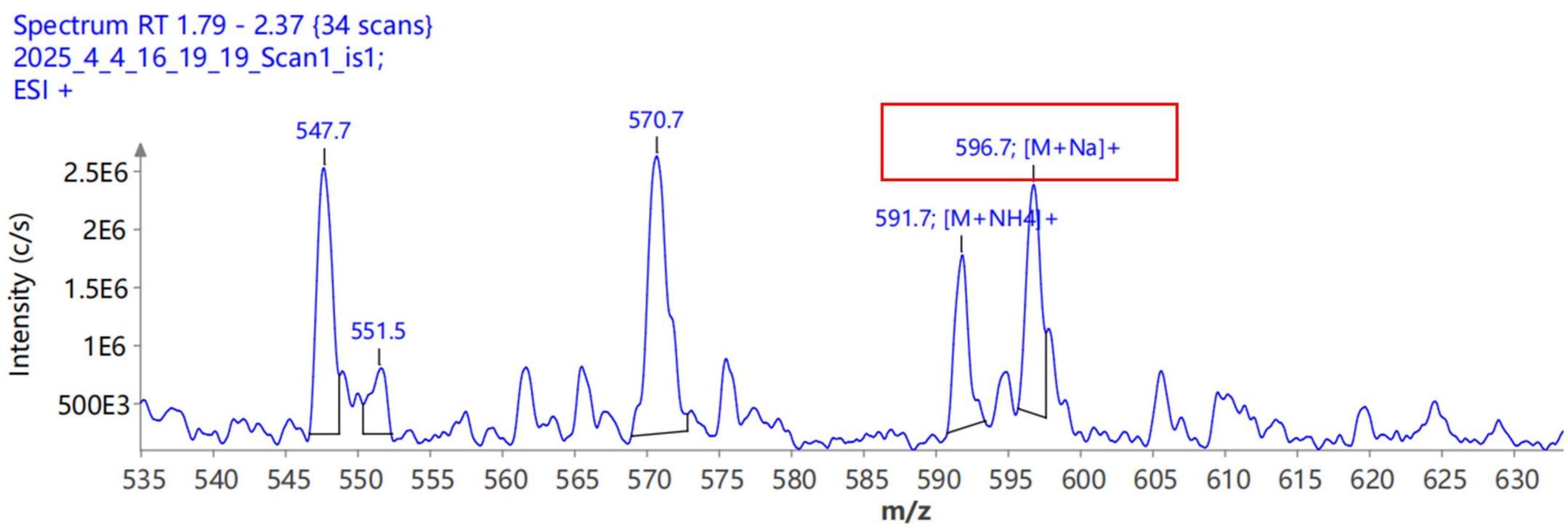

Supplementary Figure 60. TLC-MS spectrum of recovered oil-based catalyst (OBC).

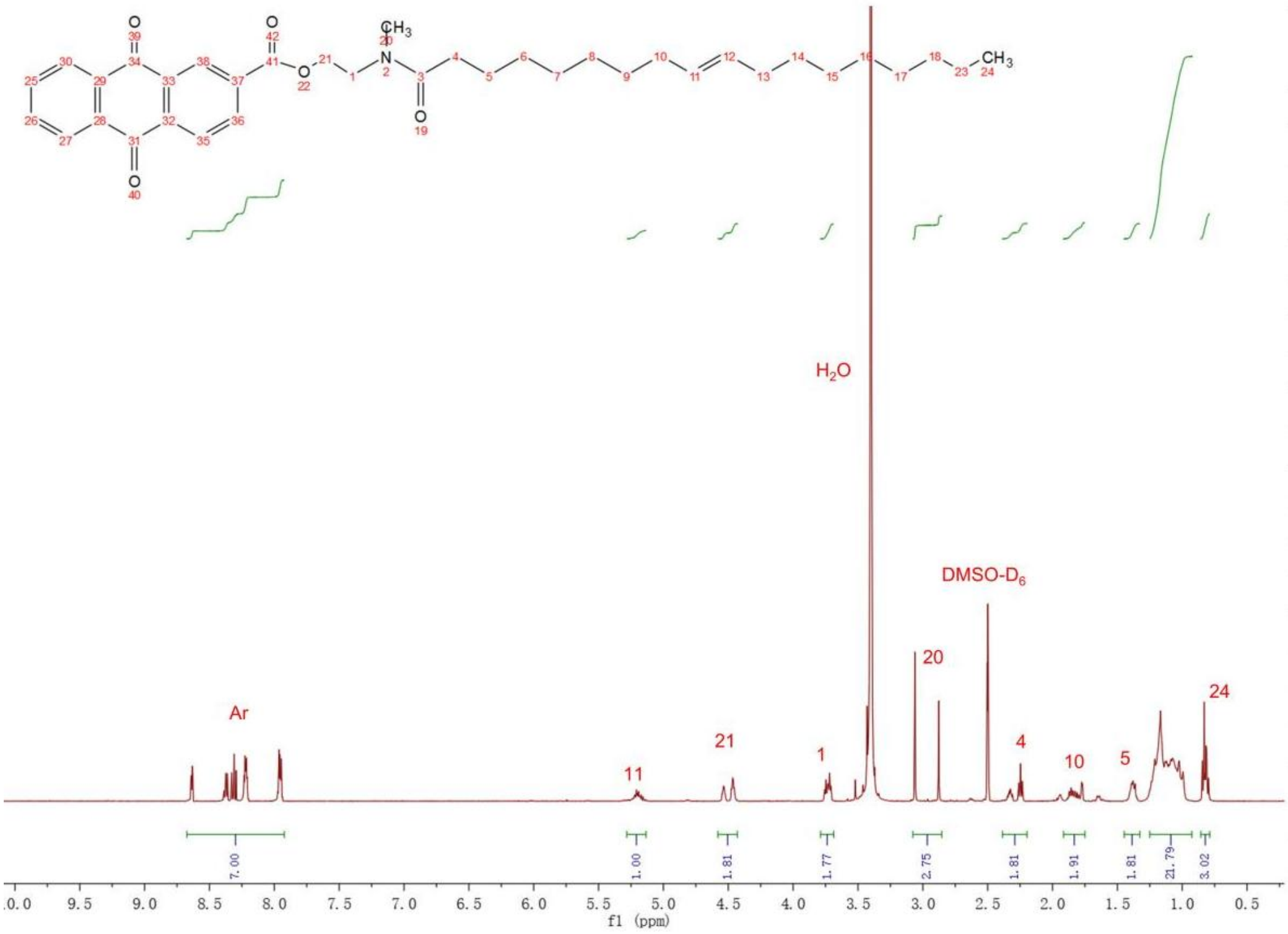

Supplementary Figure 61. ^{1}H NMR spectrum of recycled oil-based catalyst (OBC).

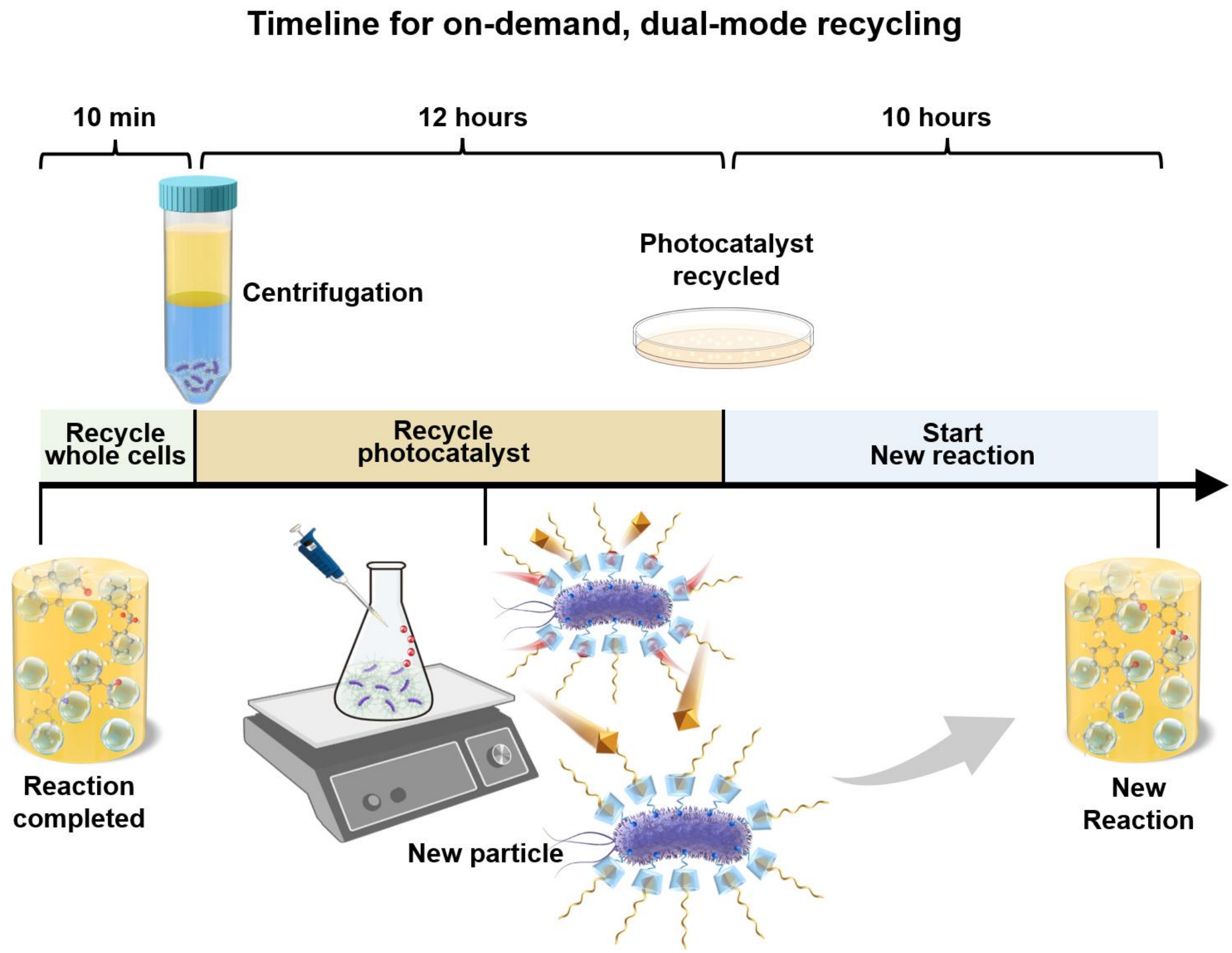

Supplementary Figure 62. Operational workflows and timelines for dual-mode, on-demand recycling.

Supplementary Reference

1 Wu, X., Wang, Z., Li, M., Shao, C. & Wang, Z. Ultrarobust and water-insensitive healable elastomers from hydrophobic multiple phase-locking microstructures. *Chem. Eng. J.* **520**, (2025).

2 Wu, X., Karring, H., Wang, Z. & Wu, C. Protein-cell conjugates as artificial surface display for interfacial biocatalysis. *Chem. Sci.* **16**, 4892-4899, (2025).

3 Wang, S., Hubner, R., Karring, H., Batista, V. F. & Wu, C. A supramolecular approach to engineering living cells with enzymes for adaptive and recyclable cascade synthesis. *Angew. Chem. Int. Ed.*, **e202416556**, (2024).

4 Ning, J., Sun, Z., Hübner, R., Karring, H., Ebbesen, M. F., Dimde, M., & Wu, C. Engineering living cells with polymers for recyclable photoenzymatic catalysis. *Nat. catal.* **7**, 1404-1416, (2024).

5 Sun, Z., Hubner, R., Li, J. & Wu, C. Artificially sporulated Escherichia coli cells as a robust cell factory for interfacial biocatalysis. *Nat. Commun.* **13**, 3142, (2022).

6 Zhang, J., Batista, V. F., Hubner, R., Vogel, S. & Wu, C. Combining liposomal photocatalysts with whole-cell catalysts for one-pot photobiocatalysis. *Small*, **e2408666**, (2024).

7 Sun, Z., Zhao, Q., Haag, R. & Wu, C. Responsive emulsions for sequential multienzyme cascades. *Angew. Chem. Int. Ed.* **60**, 8410-8414, (2021).
