# Supplementary material for: Assembly-Line Biosynthesis in Living-Cell Emulsions via Tunable Supramolecular Surface Chemistry
Source: Nat Commun. 2026 Jun 24;17:7948. doi: 10.1038/s41467-026-74245-z (PMC13448702; doi:10.1038/s41467-026-74245-z)
Supplement: Supplementary file 2 [file 41467_2026_74245_MOESM2_ESM.pdf]

Description of Additional Supplementary Files

Supplementary Movie 1: Formation and stability of the Pickering emulsion stabilized by cell@OBC upon gentle handshaking.

Supplementary Movie 2: Scale-up of the Pickering emulsion system and interfacial biocatalysis process.
