# Supplementary material for: Assembly-Line Biosynthesis in Living-Cell Emulsions via Tunable Supramolecular Surface Chemistry
Source: Nat Commun. 2026 Jun 24;17:7948. doi: 10.1038/s41467-026-74245-z (PMC13448702; doi:10.1038/s41467-026-74245-z)
Supplement: Supplementary file 4 [file 41467_2026_74245_MOESM6_ESM.pdf]

nature portfolio

Peer Review File

Assembly-Line Biosynthesis in Living-Cell Emulsions via Tunable Supramolecular Surface Chemistry

Corresponding Author: Professor Changzhu Wu

This file contains all reviewer reports in order by version, followed by all author rebuttals in order by version.

Version 0:

Reviewer comments:

Reviewer #1

(Remarks to the Author)
This manuscript presents an innovative and conceptually appealing strategy for integrating chemical and biological catalysis within a living, compartmentalized Pickering emulsion system. The use of supramolecular surface chemistry to transform engineered cells into active, recyclable emulsifiers represents a creative advance with clear potential for multienzyme cascades and green chemical synthesis. Overall, the work introduces an imaginative and powerful platform for chemoenzymatic catalysis that is likely to stimulate further research in living materials, multienzyme cascades, and sustainable chemical manufacturing. The study is of broad interest to the catalysis and industrial biotechnology communities and, in my view, is suitable for publication after revision, provided that the points outlined below are carefully addressed.
1. Background and Positioning:
The authors provide a strong biological and conceptual motivation, drawing inspiration from nature's use of spatially organized enzyme cascades. To further strengthen the manuscript, the authors are encouraged to more comprehensively position their work within the existing literature on biocatalysis in emulsions and Pickering systems, which are already known to outperform conventional biphasic systems for certain transformations. Clarifying how the present platform advances beyond prior art—whether through the living emulsifier concept, the dynamic supramolecular linkage, the dual recycling strategy, or their synergistic combination—will help readers more clearly appreciate the novelty.
2. Performance and Experimental Validation:
The reported rate enhancements and the demonstration of gram-scale benzoin synthesis highlight the promise of the system. To fully substantiate these claims, the control experiments should be strengthened by providing detailed experimental procedures, repeating key experiments in triplicate, and including appropriate error bars. This additional rigor will significantly enhance the credibility and reproducibility of the reported performance advantages.
3. Cascade Design and Reaction Choice:
The incorporation of photocatalytic steps into the chemoenzymatic cascades is interesting and expands the scope of the platform. Nevertheless, the manuscript would benefit from a clearer justification for the choice of photocatalysis, particularly in cases where highly efficient ADH-catalyzed reductions are well established. A brief discussion comparing the advantages and limitations of photocatalytic versus purely enzymatic cascades would further strengthen the mechanistic and practical relevance of the work. In addition, a more detailed discussion of the photo–enzymatic cascade reactions, including potential effects of light exposure on enzyme stability and long-term operation, would be valuable.
4. Recycling Strategy and Practical Considerations:
The dual-mode recycling strategy enabled by the dynamic supramolecular linkage is an elegant and appealing feature of the system. At the same time, the authors are encouraged to comment on the practical complexity and time requirements of the recycling procedure and to discuss whether further simplification or process integration may be possible. Such a discussion would help readers assess the real-world applicability of the approach.
5. Quantitative Details and Limitations:
To further enhance the manuscript's impact, the authors should explicitly report and discuss catalyst loading, including comparisons with existing systems. In addition, a clearer treatment of NAD^+/NADH recycling—covering efficiency, stability, and potential limitations during extended operation—would improve the completeness of the catalytic analysis.
6. Overall Limitations:
Finally, while the overall potential of the system is evident, a more balanced discussion of current limitations—such as substrate scope, scalability, light penetration, and long-term cell viability—would provide a fair and realistic perspective and further strengthen the manuscript.

Reviewer #2

(Remarks to the Author)
The authors have good background on chemical modified bacterial cells for biocatatlysis as well as integrating photocatalysis with living cell biocatalyisis. The present manuscript reports a conceptually novel and technically elegant strategy that transforms living Escherichia coli into programmable amphiphilic colloidal catalysts by combining β-cyclodextrin-based surface engineering with oil-based photocatalysts. The resulting hybrid system autonomously self-assembles into Pickering emulsions, creating efficient interfacial microreactors that accelerate a broad range of enzymatic, chemoenzymatic, and photoenzymatic reactions. On-demand, selective recycling of either the living cells or the synthetic photocatalyst—enabled by reversible host–guest interactions—represents a clear advance over existing Pickering emulsion biocatalysis and living-cell immobilization approaches, where co-immobilization typically hinders selective regeneration. Overall, the work establishes a versatile, modular, and sustainable platform with clear implications for biocatalysis, supramolecular chemistry, and engineered living materials.
Please highlight the progresses of the present work comparison to your previous works and check the following points
1. The conclusion on controllable grafting is currently supported only by fluorescence intensity (Figure 2b), which is not fully convincing. Please provide additional data to substantiate this claim.
2. A small amount of DMSO is used during cell surface modification, whereas the organic solvent resistance experiments employ toluene. To validate the general robustness of cell@OBC, please include resistance tests against additional organic solvents.
3. It is unclear whether enrichment of oil-based photocatalysts (OBC) and β-CD on the cell surface affects cell charge properties. This aspect appears to be missing and should be addressed, ideally with supporting data or discussion.
4. The interfacial area is lower at water-to-oil ratios of 2:8, 1:9, and 3:7 compared with other ratios (Figure 3o). Please provide a clearer explanation for this observation.
5. The industrial potential of assembly-line biocatalysis is demonstrated through scale-up experiments, but it is not clear whether stirring or shaking is required in all catalytic reactions, including scale-up. Please clarify the agitation conditions used.
6. The protective coating on the cell surface may introduce an additional mass transfer barrier. Compared with unmodified cells, are substrates or products more difficult to cross the cell membrane? A brief discussion would be helpful.
7. The recycling of oil-based photocatalysts is an attractive feature. Please clarify the rationale for choosing 1-adamantanecarboxylic acid as the competitive guest.
8. The engineered E. coli can be reproduced by cell culture and the co-factor of reductase can be regenerated by utilizing co-substrate, which indicates that the biocatalytic reaction is catalyzed by living cells. Thus, the photocatalytic chemical reaction integrating with biocatalytic reaction catalyzed by living cells have achieved in the present work. However, the replicated cells should have no the modified characteristics (Fig.S10), such as hydrophobic surfaces. Those cells will lose the ability to act as Pickering emulsifiers. Does it influence the recycling of cells as biocatalyst?
9. Chemo-enzymatic cascades, which merge chemical and biological catalysis, are emerging as powerful tools for sustainable chemical production. The biocompatible chemistry has even applied to regulate microbial metabolism (Wallace et al. Using non-enzymatic chemistry to influence microbial metabolism. Curr Opin Chem Biol 2015, 25: 71-79; Xue et al. Driving non-enzymatic reactions towards biocompatible chemistry. Trends in chemistry 2025, 7(2): 66-69). The photo-catalysis of chemical reaction should be immobilized catalyst on the cell surfaces in the present work (Fig.S6) as well as the previous works (Zhou et al. Integrating micelle catalysts with living cells for recyclable photoenzymatic cascades. Chem catalysis 2025, doi.org/10.1016/j.checat.2025.101517). Why did not add the photo-catalyst directly into the oil-water two-phase system (in other words, providing the benefits of integrating of chemical catalyst with biocatalyst) ?
Other minor remarks:
1. In terms of system design, the reason for choosing plant oil is not clearly stated. Please briefly justify this choice.
2. “Superbacteria” are shown in Figure 1 and the manuscript, additional explanation in the text would improve clarity.
3. In the plate assay in Figure 2s (right panel), zones 2 and 3 are not clearly separated. Please repeat the experiment or provide a clearer image.
4. Details of the photoenzyme-catalyzed reactions in Figures 4 and 5 (reaction conditions, etc.) should be provided, preferably in the Supporting Information.
5. The term cell@OBC is used frequently, but it is difficult for readers to recognize it in the figures. Please label cell@OBC explicitly to improve readability.

Reviewer #3

(Remarks to the Author)
The work by Wu et al. presents a nice work of biocatalysis using bacteria-stabilized Pickering emulsions. The work is interesting and the science is correct. Unfortunately, the topic and the work itself does not contain the novelty required for a publication in Nature Communications. Of course the paper is publishable but not in this journal. I recommend a submission to a more specialized journal, e.g., Communication Chemistry. Some additional comments.

-Recent reviews should be included in the reference list: https://doi.org/10.1002/cctc.202400856
-the claim that typically Pickering emulsions rely on inert paritcles like silica or polymers is very strong. THere are many examples of Pickering stabilization.
-Some mention to the alignment of the work to the Green Chemistry Principles would be good
-Using hand shaking as strategy for transporting the stabilizing agent to the interface opens an important question related to the irreversibility of the attachment of the species, and this opens the question about the true Pickering stabilization

-Figure 3h is counterintuitive, CI is defined in the opposite direction expected for creaming. This mean than CI is high when the stabilizing column is big, and the opposite is true for low CI index. I recommend a carful revision.
-The maximum ingerface covering is claimed at water-oil ratio 6:4. However, no explanation about this issue is included.
-It is not clear the ability for scaling in the studied system, some additional discussion and evidences should be included.

Version 1:

Reviewer comments:

Reviewer #1

(Remarks to the Author)
The revised version, with well-justified novelty, clear presentation, thoughtful discussion, expanded supporting information, and new data, has fully addressed my concerns regarding the original manuscript. Based on my evaluation, the manuscript is now suitable for publication.

Reviewer #2

(Remarks to the Author)
The authors have modified the text and clarified my presenting points.
It is OK

Reviewer #3

(Remarks to the Author)
After carefully evaluating the revised version submitted by the authors, I find that the manuscript is now suitable for publication. I appreciate the authors' diligent efforts in addressing the comments and substantially improving the quality of the work.

Open Access This Peer Review File is licensed under a Creative Commons Attribution 4.0 International License, which permits use, sharing, adaptation, distribution and reproduction in any medium or format, as long as you give appropriate credit to the original author(s) and the source, provide a link to the Creative Commons license, and indicate if changes were made.
In cases where reviewers are anonymous, credit should be given to 'Anonymous Referee' and the source.
The images or other third party material in this Peer Review File are included in the article's Creative Commons license, unless indicated otherwise in a credit line to the material. If material is not included in the article's Creative Commons license and your intended use is not permitted by statutory regulation or exceeds the permitted use, you will need to obtain permission directly from the copyright holder.
To view a copy of this license, visit https://creativecommons.org/licenses/by/4.0/

Point-by-point Reply

Reviewer #1:

This manuscript presents an innovative and conceptually appealing strategy for integrating chemical and biological catalysis within a living, compartmentalized Pickering emulsion system. The use of supramolecular surface chemistry to transform engineered cells into active, recyclable emulsifiers represents a creative advance with clear potential for multienzyme cascades and green chemical synthesis. Overall, the work introduces an imaginative and powerful platform for chemoenzymatic catalysis that is likely to stimulate further research in living materials, multienzyme cascades, and sustainable chemical manufacturing. The study is of broad interest to the catalysis and industrial biotechnology communities and, in my view, is suitable for publication after revision, provided that the points outlined below are carefully addressed.

Reply: We appreciate your recognition of our work, and we have revised it to improve the quality based on your suggestions.

Comment 1: Background and Positioning: the authors provide a strong biological and conceptual motivation, drawing inspiration from nature's use of spatially organized enzyme cascades. To further strengthen the manuscript, the authors are encouraged to more comprehensively position their work within the existing literature on biocatalysis in emulsions and Pickering systems, which are already known to outperform conventional biphasic systems for certain transformations. Clarifying how the present platform advances beyond prior art — whether through the living emulsifier concept, the dynamic supramolecular linkage, the dual recycling strategy, or their synergistic combination—will help readers more clearly appreciate the novelty.

Reply: Thank you for your constructive comment. We have followed your suggestion and updated the introduction part accordingly. The revised introduction states that "*we design and construct a*

living interfacial biocatalytic platform that goes beyond conventional Pickering emulsion-based systems. By integrating principles of supramolecular chemistry and interfacial engineering, we introduce an "all-in-one" strategy to transform engineered E. coli cells into dynamic "superbacteria" particles that function simultaneously as emulsifiers and active catalytic microreactors".

First, we acknowledge that Pickering emulsions have been widely used to enhance biphasic reactions by increasing interfacial areas and improving mass transfer. However, most existing Pickering-based interfacial biocatalytic systems remain built on static particulate interfaces, offering limited control over interfacial assembly, reconfiguration, and catalyst recovery. Catalyst deactivation accumulates over repeated cycles, regeneration is difficult, and the integration of mechanistically distinct catalytic functions typically requires additional immobilization layers or separate catalyst entities, thereby increasing system complexity rather than reducing it. These constraints hinder their broader application in complex chemoenzymatic cascades and sustainable, long-term operation.

Second, the central conceptual advance of our work is the use of living cells as the emulsifying and catalytic chassis, transforming *E. coli* into active interfacial microreactors rather than decorating particles. By dynamically programming the cell surface to become amphiphilic, the cells self-assemble at the oil–water interface. Importantly, the catalytic module is introduced via reversible supramolecular grafting instead of permanent covalent immobilization. This design choice is critical: it allows the interfacial architecture to remain reconfigurable and renewable, a feature fundamentally inaccessible to conventional Pickering systems based on static materials. Thus, the novelty of our approach lies not in using a Pickering emulsion itself, but in redefining the interface as a living, programmable, and adaptive catalytic entity.

Third, our system enables functionalities that go beyond prior Pickering biocatalysis. The same interface can host chemoenzymatic and photoenzymatic transformations, without requiring separate catalyst supports or complex assembly steps. Furthermore, the reversible supramolecular

linkage uniquely enables an on-demand, dual-mode recycling strategy, in which either the entire living interfacial particle can be reused or the synthetic oil based photocatalyst can be selectively recycled and re-grafted onto fresh cells. These capabilities arise from the synergistic combination of a living emulsifier, dynamic supramolecular chemistry, and interfacial confinement, rather than from increased interfacial areas alone. We have revised the introduction section accordingly to make these distinctions and the resulting innovation explicit.

In summary, these revisions clearly describe the innovation of our work: building a living, programmable interface with reconfigurable assembly and customizable recycling. We believe this strengthened framing makes the "advance beyond prior art" and the rationale for our all-in-one design immediately apparent to a broad readership.

Comment 2: Performance and Experimental Validation: the reported rate enhancements and the demonstration of gram-scale benzoin synthesis highlight the promise of the system. To fully substantiate these claims, the control experiments should be strengthened by providing detailed experimental procedures, repeating key experiments in triplicate, and including appropriate error bars. This additional rigor will significantly enhance the credibility and reproducibility of the reported performance advantages.

Reply: Thank you for your valuable suggestion. In the revised manuscript and Supporting Information, we have strengthened the experimental validation in the following specific ways:

- We have substantially expanded the Supporting Information (from the previous 30 pages to the current 73 pages) to provide detailed descriptions of cell surface modification, Pickering emulsion formation, photocatalytic reaction setup, and scale-up experiments.
- We have clarified and documented the experimental details of all key reactions, including single-step transformations as well as chemo- and photoenzymatic cascade reactions, and added error bars to all relevant datasets.

We believe that these revisions significantly improve the rigor, credibility, and reproducibility of the study.

Comment 3: Cascade Design and Reaction Choice: the incorporation of photocatalytic steps into the chemoenzymatic cascades is interesting and expands the scope of the platform. Nevertheless, the manuscript would benefit from a clearer justification for the choice of photocatalysis, particularly in cases where highly efficient ADH-catalyzed reductions are well established. A brief discussion comparing the advantages and limitations of photocatalytic versus purely enzymatic cascades would further strengthen the mechanistic and practical relevance of the work. In addition, a more detailed discussion of the photo–enzymatic cascade reactions, including potential effects of light exposure on enzyme stability and long-term operation, would be valuable.

Reply: Thank you for your questions and suggestions. We agree that ADH-catalyzed reduction is indeed a well-established transformation. However, the motivation for introducing photocatalysis reaction in our work is not to replace or outperform ADH catalysis, but rather to address functional limitations that arise in biphasic or multistep cascade systems, and to demonstrate a complementary catalytic modality that offers control and operational flexibility. Specifically,

- **the photocatalysis was selected for the following reasons**:

First, photocatalysis was selected because light represents a clean, traceless, and externally supplied energy input, making it particularly attractive for sustainable catalytic systems. Unlike enzymatic reactions that rely on cofactors as internal energy carriers and require dedicated regeneration cycles, photon energy can be introduced without additional reagents, and without imposing metabolic burden on the biological component. From a process perspective, this feature simplifies system design and facilitates integration with whole-cell biocatalysis.

Second, equally important, light provides unique spatiotemporal controllability that is not readily accessible in enzyme-only systems. The catalytic activity can be precisely initiated, modulated, or terminated by switching the light on or off, enabling direct external regulation of the reaction

progress. This controllability is clearly demonstrated in **Supplementary Figure. 26**, where the photocatalytic reaction proceeds exclusively under UV and is immediately suppressed upon removal of light. Such instantaneous control is difficult to achieve with enzymatic catalysis once substrates and cofactors are present in solution.

Third, in the context of interfacial and cascade catalysis, this externally addressable energy input is particularly advantageous. It allows the photochemical step to be temporally coordinated with enzymatic transformations and provides an additional level of regulation in complex, multi-step reaction networks. These considerations motivated us to incorporate photocatalysis as an integral component of our Pickering interfacial biocatalytic platform. Our work aims to establish living cells as versatile, interface-localized catalytic platforms that can host multiple catalytic modalities. Photocatalysis represents a mechanistically distinct process, making it a stringent test of the robustness and versatility of the engineered whole-cell Pickering interface. Successful operation under photocatalytic conditions strengthens the generality of the platform beyond conventional enzyme-only cascades.

- **Comparison between photocatalysis and enzymatic catalysis**

Photocatalysis and enzymatic catalysis possess complementary strengths as well as inherent limitations. Enzymatic catalysis, such as ADH-mediated reactions, offers exceptional chemo- and stereoselectivity and operates efficiently under mild, aqueous conditions. However, it typically relies on specific cofactors/cosubstrates and regeneration systems and lacks straightforward external control once the reaction is initiated. In contrast, photocatalysis is less selective at the molecular level but provides cofactor-free operation and precise spatiotemporal controllability through light regulation, enabling flexible modulation of reaction rates and timing. Meanwhile, photocatalytic processes may introduce photochemical stress and therefore require careful system design to ensure compatibility with biological components. These differences indicate that the two catalytic modes are not competing but synergistic. Integrating these two methods within a single Pickering

interface platform enables access to more complex cascade reactions while mitigating the individual limitations of each approach.

- **Impact of light exposure on enzyme stability and long-term operation**

We acknowledge your concern that light exposure may affect enzyme and cellular stability during photoenzymatic catalysis. Importantly, **Fig. 3c** indicates that compared to natural cells, cell@OBC exhibits significantly enhanced resistance to UV irradiation, suggesting that the supramolecular coating and interfacial structure offer a protective effect against photodamage. This conclusion is further supported by the live/dead assay shown in **Supplementary Figure 13**. These experiments were designed as comparative stress tests rather than exact representations of reaction conditions, and they highlight the robustness gained through interface engineering. Moreover, from an operational perspective, our system is inherently suited for long-term use because the photocatalyst and the biological component are functionally decoupled. When cellular activity declines, the photocatalyst can be selectively recycled and reassembled onto fresh cells. This design mitigates concerns associated with cumulative light-induced deactivation and enables sustained operation without sacrificing the light-harvesting component.

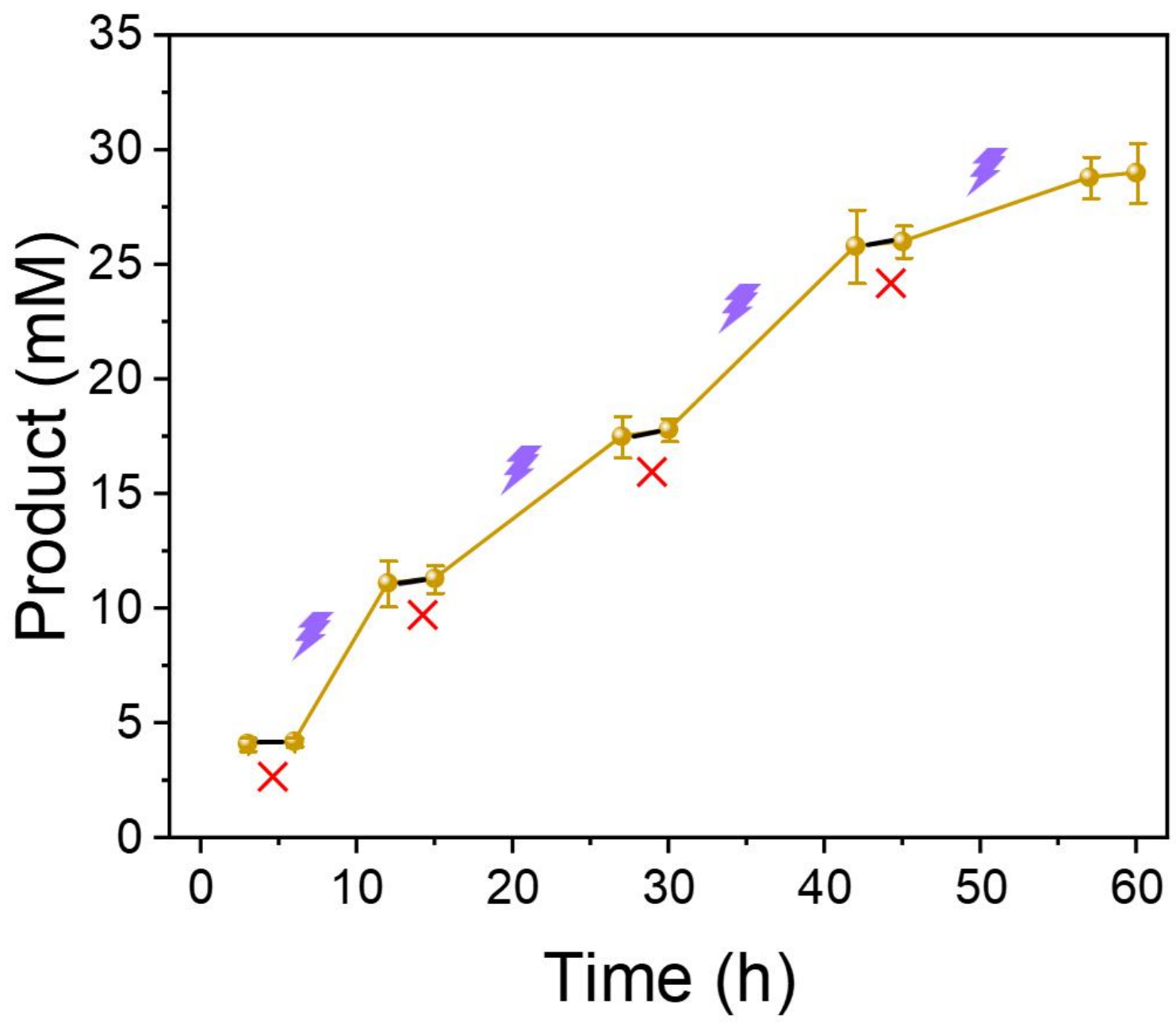

Supplementary Figure 26. Time-dependent benzaldehyde formation under alternating UV light irradiation and dark conditions. The yellow line corresponds to periods of UV irradiation, whereas the black line indicates dark intervals (3 h each). A significant difference in reaction rates is observed between light-on and light-off periods, with faster benzaldehyde accumulation under UV, confirming the controllability of the photocatalysis.

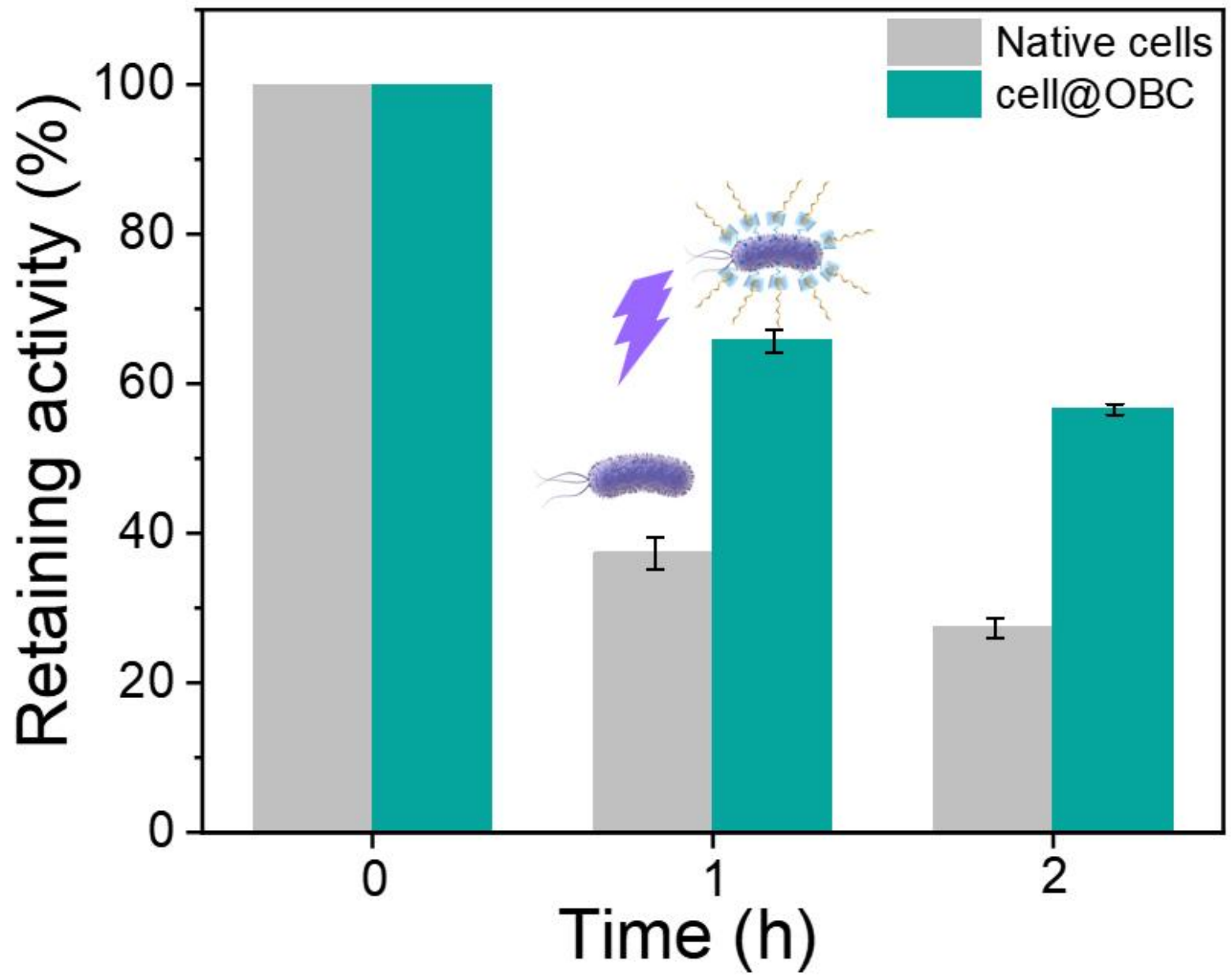

Figure 3c. Retaining activity of native cells (gray) and cell@OBC (green) measured after 0, 1, and 2 h of continuous UV exposure.

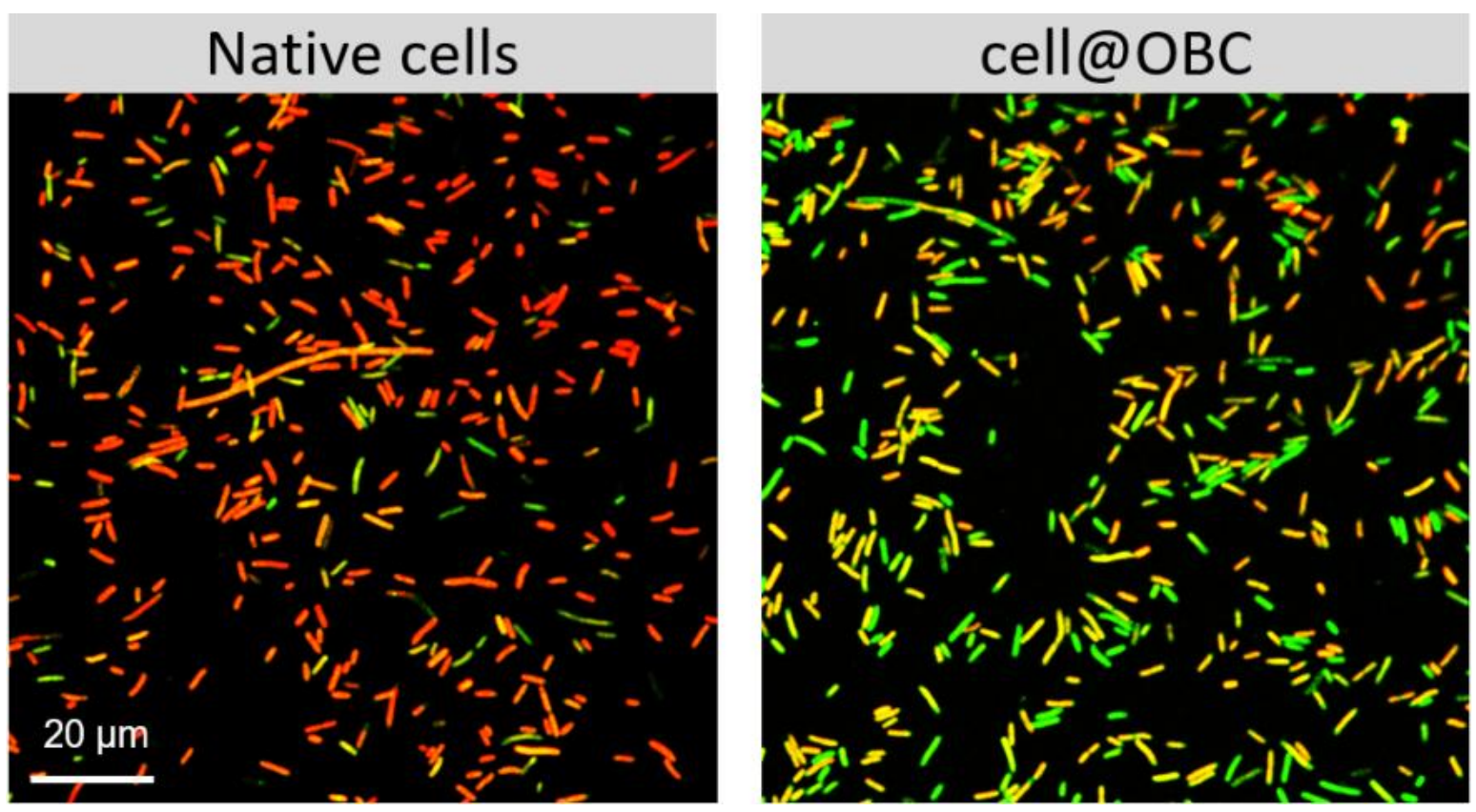

Supplementary Figure 13. Live/dead assay of native cells and cell@OBC after 2 hours of UV exposure.

Comment 4: Recycling Strategy and Practical Considerations: the dual-mode recycling strategy enabled by the dynamic supramolecular linkage is an elegant and appealing feature of the system. At the same time, the authors are encouraged to comment on the practical complexity and time requirements of the recycling procedure and to discuss whether further simplification or process integration may be possible. Such a discussion would help readers assess the real-world applicability of the approach.

Reply: Thank you for your suggestion. We would like to clarify that the current dual-mode, on-demand recycling strategy is intentionally designed as a customized and sufficiently simple solution, rather than a complex multi-step procedure. Specifically, as shown in the newly added schematic timeline (**Supplementary Figure 62**), the system allows two distinct and selectable recycling routes, depending on the desired balance between speed and catalyst renewal:

1) Rapid whole cell recycling (**≈10 min**).

After reaction completion, the Pickering emulsion can be quickly demulsified and the whole cell particles recycled by simple centrifugation within approximately 10 minutes. This mode is intended for short-cycle, high-throughput operation.

2) Photocatalyst-selective recycling and regeneration (**≈12 h**).

Alternatively, the photocatalyst can be selectively separated from the modified cells and reused to build a new reaction interface. Although this way requires a longer processing time (≈12 h), it enables independent renewal of the catalytic interface without discarding functional photocatalyst components. This mode is particularly relevant when photocatalyst stability or long-term performance is a concern, because the photocatalyst represents a high-value synthetic component whose loss would significantly undermine process sustainability and cost-efficiency.

These two recycling modes are decoupled and modular, people can switch between rapid whole-cell reuse and photocatalyst-focused regeneration on demand, depending on process requirements. We have added this timeline schematic and corresponding descriptions to the

revised manuscript to clarify that the dual-mode recycling is a flexible operational option, rather than a compulsory or overly complicated procedure. We believe this clarification substantially improves the practicality and scalability of the system.

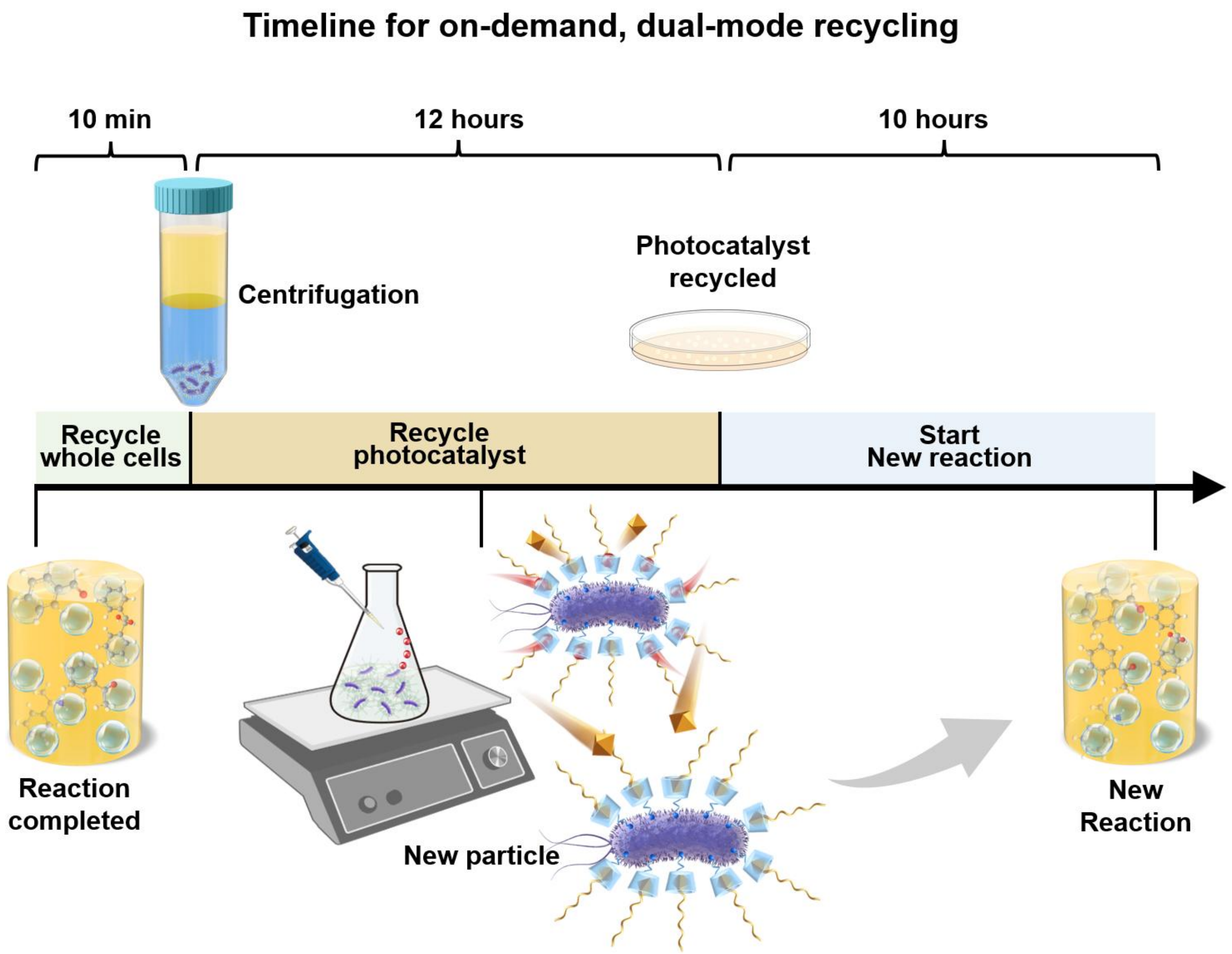

Supplementary Figure 62. Operational workflows and timelines for dual-mode, on-demand recycling.

Comment 5: Quantitative Details and Limitations: to further enhance the manuscript's impact, the authors should explicitly report and discuss catalyst loading, including comparisons with existing systems. In addition, a clearer treatment of NAD^+/NADH recycling—covering efficiency, stability, and potential limitations during extended operation — would improve the completeness of the catalytic analysis.

Reply: Thank you for your valuable suggestion. In response, we performed a dedicated UV–vis–based quantification experiment, and the detailed procedure and calculation are summarized below and illustrated in the newly added **Supplementary Figure 24**.

- UV–vis absorption spectra of the photocatalyst at different known concentrations were recorded under identical solvent and measurement conditions (**Supplementary Figure 24a**). The characteristic absorption maximum at 328 nm was selected for quantification. Based on these data, a calibration curve correlating absorbance at 328 nm with photocatalyst concentration was constructed (**Supplementary Figure 24b**), yielding a linear relationship. To determine the amount of photocatalyst immobilized on the *E. coli* surface, all washing solutions collected after cell@OBC preparation (total volume ≈ 200 mL) were combined, diluted 10-fold, and subjected to UV–vis analysis. The measured absorbance at 328 nm was approximately 0.38, corresponding to a photocatalyst concentration of 0.118 mg mL^{-1} according to the calibration curve. After correcting for the dilution factor (×10) and the total wash volume (200 mL), the total amount of photocatalyst present in the washing solutions was calculated to be 236.8 mg. The initial amount of photocatalyst introduced during cell@OBC fabrication was 252 mg. Therefore, the amount of photocatalyst remaining on the cell surface after washing is 15.2 mg. Thus, 15.2 mg of photocatalyst was successfully immobilized on the *E. coli* surface in a 40 mL system with an OD_{600} of 2.0. Normalized to reaction conditions, this corresponds to around 0.38 mg of photocatalyst per milliliter of catalytic system (OD_{600} = 2.0). Considering that the water phase accounts for approximately 60% of the total reaction volume in the Pickering emulsion, the effective photocatalyst content is approximately 0.228 mg per milliliter of emulsion catalytic system.
- Additionally, in our work, NAD^+ is supplied as a cofactor, and isopropanol (IPA) is added as a cosubstrate to enable *in situ* cofactor regeneration via alcohol dehydrogenase (ADH). This represents a well-established and self-contained ADH-mediated cofactor recycling loop, and no additional regeneration enzymes or auxiliary redox systems are required. Importantly, the

amount of NAD^+ added in our system is deliberately kept at a very low level, sufficient only to sustain enzymatic activity. Under these conditions, NAD^+ does not accumulate, nor does it dominate the reaction environment, which is consistent with standard practices in whole-cell and enzymatic cascade reactions. Therefore, the role of NAD^+/NADH recycling in this work is supportive rather than central: it is introduced solely to ensure continuous enzymatic turnover and to avoid cofactor depletion during catalysis. The focus of our work is not on developing a new recycling chemistry, but on demonstrating that the living cell Pickering interface can spatially confine and sustain coupled enzymatic and photocatalytic reactions under biphasic conditions. Furthermore, the photocatalytic oxidation module operates independently of the NAD^+/NADH redox couple. Its activity is governed exclusively by light input, whereas the enzymatic reduction and cofactor recycling proceed via ADH catalysis. As a result, the two catalytic processes are mechanistically decoupled and complementary.

- We have additionally verified its stability under the reaction conditions (**Supplementary Figure 27**): after dissolving NAD^+ and NADH separately in KPI buffer and incubating for 12 h, no changes in their characteristic UV-vis absorption peaks were observed, indicating that both cofactors remain stable over the relevant timescale. These results confirm that cofactor degradation does not influence the observed catalytic performance. Although the current concentration of NAD+ in this system is extremely low, investigating its behavior at interfaces and in photoenzymatic environments could represent an exciting future direction for our research.

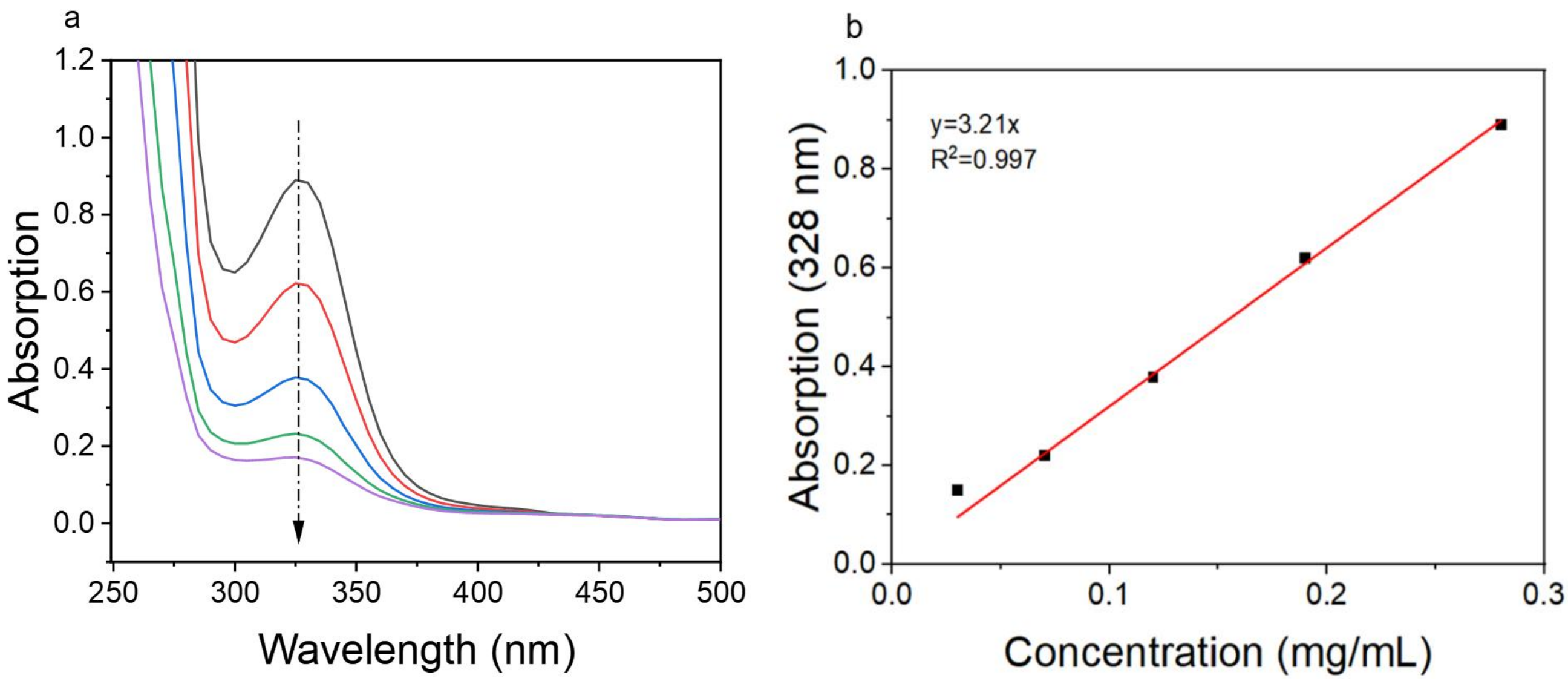

Supplementary Figure 24. UV absorption (a) and standard calibration curve (b) of the photocatalyst.

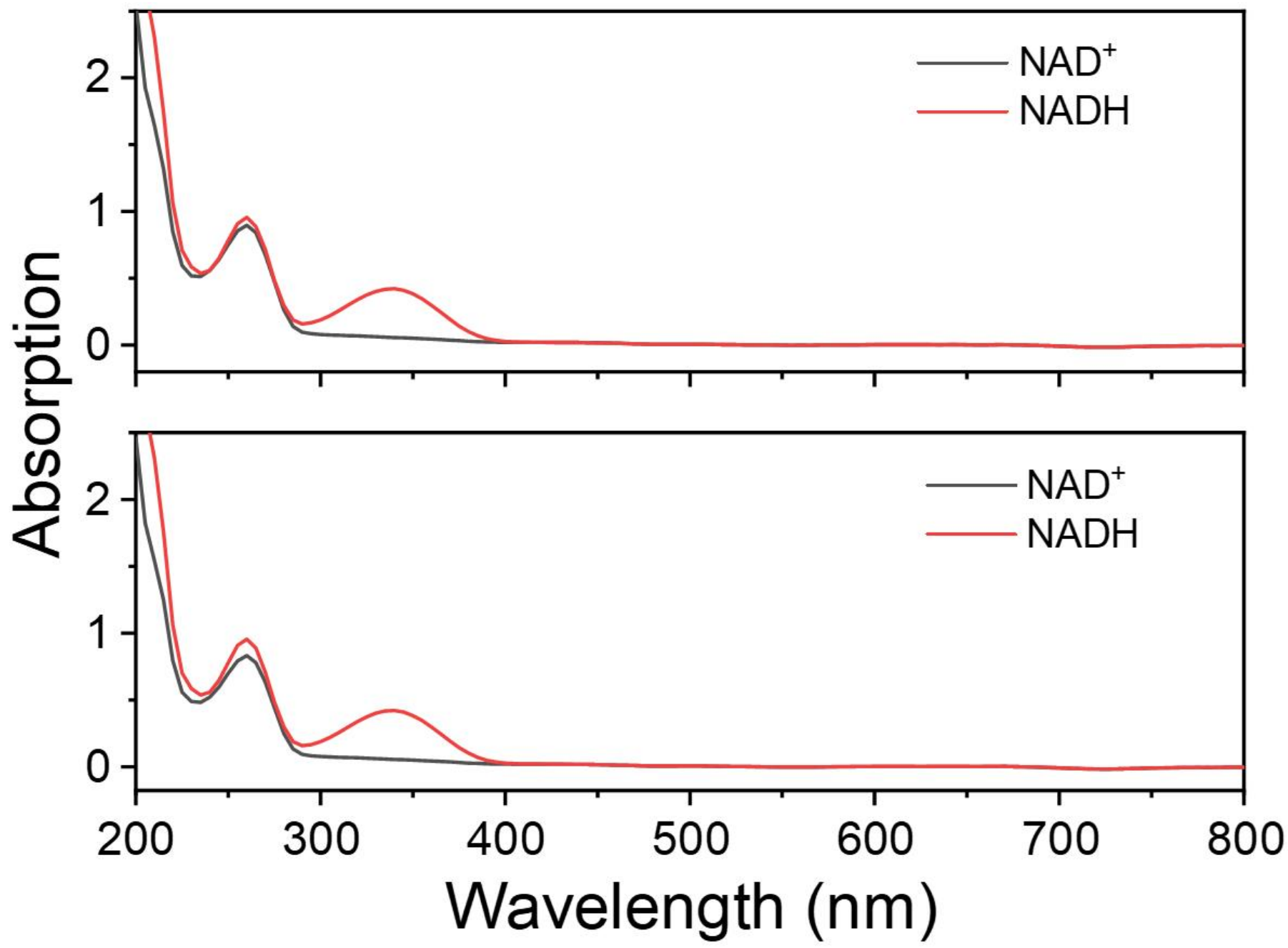

Supplementary Figure 27. The UV-Vis spectra of NAD^+ and NADH before and after 12 hours of incubation in KPI buffer confirm their stability.

Comment 6: Overall Limitations: finally, while the overall potential of the system is evident, a more balanced discussion of current limitations—such as substrate scope, scalability, light penetration,

and long-term cell viability—would provide a fair and realistic perspective and further strengthen the manuscript.

Reply: Thank you for your valuable suggestion. In the revision, we expanded the platform beyond the original model transformation by testing (i) single step interfacial biocatalysis using *E. coli* overexpressing ATA and (ii) multienzyme cascade interfacial biocatalysis using ATA and RR ADH, and we provide full structure confirmation (^{1}H NMR and GC-MS) and enantioselectivity measurements (chiral HPLC) in the revised supplementary information.

First, the ATA single-step module was validated on five structurally distinct amine substrates, giving conversions spanning 28-97% (entries 1-5, **Supplementary table 2**), which directly demonstrates that the living cell Pickering microreactor is not limited to a aromatic substrate but can accommodate substituent variation (electron-donating/withdrawing groups and larger aromatic frameworks). Additionally, the cascade reaction was further evaluated on five substrates, delivering secondary alcohol products with high stereoselectivity in most cases (*ee* value up to 99%) together with conversions from 19-93% depending on substrate structure (entries 1-4, **Supplementary Table 3**), supporting the platform's capability for enantioselective cascade synthesis in the interfacial setting rather than only single-enzyme turnover. However, for the final cascade entry (entry 5, **Supplementary Table 3**), the product was too low to reliably quantify the *ee* value.

Taken together, these additional experiments demonstrate that the proposed living cell Pickering interfacial system is not limited to a single model substrate, but instead represents a general and adaptable catalytic strategy. The successful extension from the initial reaction to both ATA-mediated single-step transformations and ATA/RR ADH cascade reactions, supported by comprehensive structural characterization (^{1}H NMR, GC–MS, and chiral HPLC), shows that the platform can accommodate different enzyme modules and substrate structures while retaining stereochemical control in most cases. Variations in conversion observed for certain substrates

reflect intrinsic substrate-dependent kinetics rather than a fundamental limitation of the interfacial design, and they delineate realistic boundaries for further optimization.

Second, in addition to substrate generality, we also addressed scalability at the process level. By increasing the reaction volume from the milliliter scale to a 5 L Pickering emulsion, we confirmed that the interfacial structure remains stable and operational under substantially enlarged volumes (**Figs. 6i, 6j** and **Supplementary Figure 54**). Using this scaled system, the interfacial biocatalytic conversion of benzaldehyde to benzoin afforded approximately 7.5 g of chiral benzoin, demonstrating that both catalytic performance and stereochemical outcome can be preserved upon scale-up. In summary, the substrate scope and scale-up results support the conclusion that this work introduces a versatile and scalable interfacial biocatalytic platform, rather than a reaction-specific proof of concept.

Third, regarding light penetration, in our design, the UV-active photocatalyst is localized at the water – oil interface via the assembly of amphiphilic cell@OBC particles, rather than being dispersed throughout the bulk phase. This interfacial confinement significantly shortens the effective optical path length and alleviates UV attenuation in the turbid emulsion. Notably, recent studies have shown that UV-driven photocatalysis can be efficiently performed in Pickering/emulsion systems when the photoactive components are concentrated at the interface, despite limited bulk transparency (DOI: 10.1016/S1872-2067(23)64586-3; DOI: 10.1016/j.seppur.2024.131354). Consistent with these reports, the pronounced UV light on/off rate switching and sustained photocatalytic activity observed in our system demonstrate that UV penetration is sufficient to activate the interfacial photocatalyst under the employed conditions.

Fourth, regarding long-term cell viability, we emphasize that the living cells in this system primarily serve as a renewable and functional interfacial particles, rather than as indefinitely stable biocatalysts. Under photocatalytic conditions, gradual loss of cellular activity over extended operation times is expected and is explicitly accommodated in our design. Importantly, the dynamic

supramolecular host–guest linkage enables an on-demand dual-mode recycling strategy, allowing either reuse of intact cell@OBC when activity is retained, or selective recycle of the synthetic photocatalyst and its reassembly onto fresh cells once cellular performance declines. The sustained catalytic activity over multiple cycles and the successful implementation of this recycling strategy demonstrate that long-term productivity of the system does not rely on indefinite cell viability, but instead on the modular and renewable nature of the interfacial architecture.

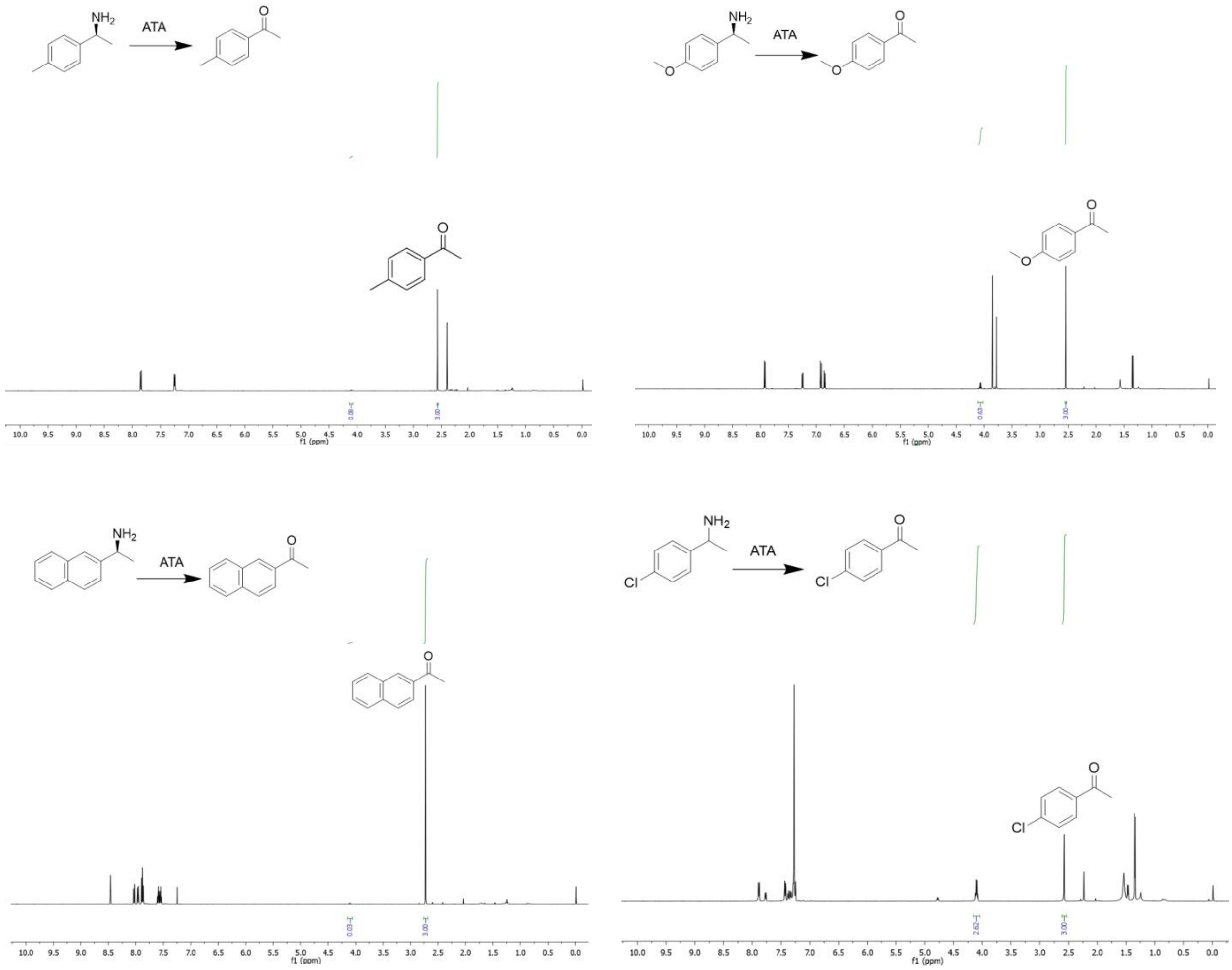

Supplementary Figure 29-Supplementary Figure 32. ^{1}H NMR spectrum of four ketones with different structures, reactions catalyzed by *E. coli* overexpressing ATA.

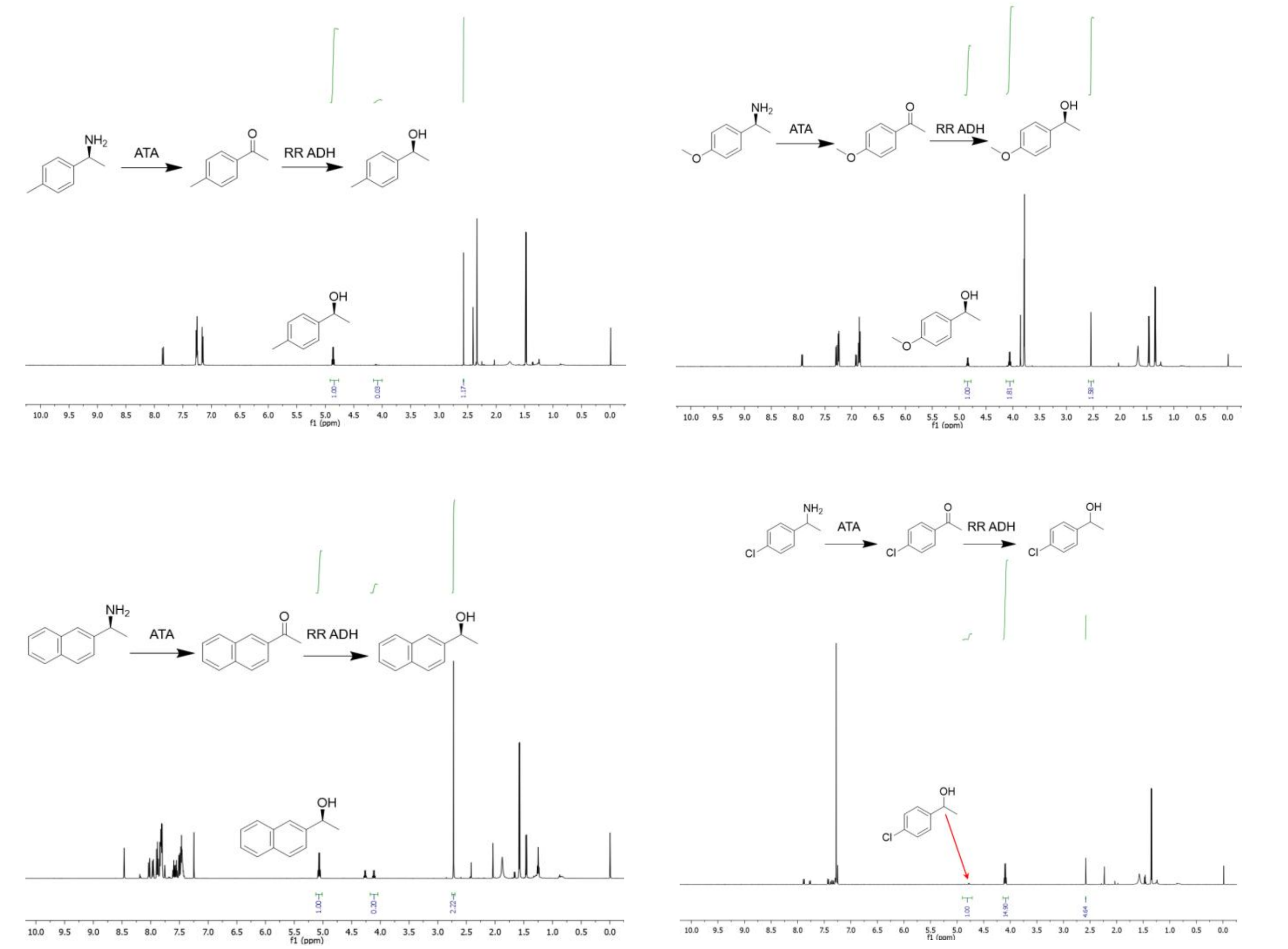

Supplementary Figure 38-Supplementary Figure 41. ^{1}H NMR spectrum of four alcohols with different structures, synthesized by cascade catalysis in *E. coli* overexpressing ATA and RR ADH.

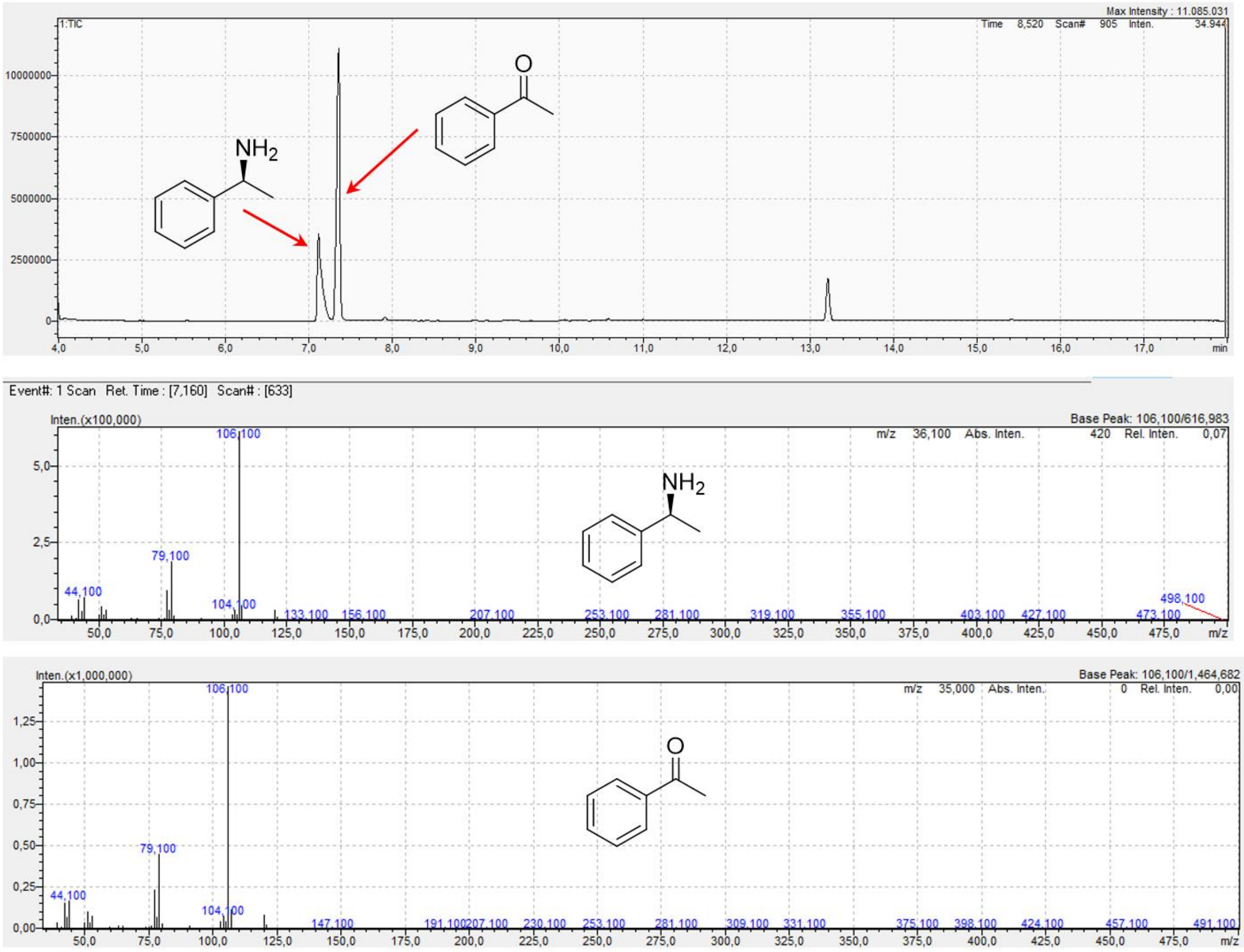

Supplementary Figure 33. GC and MS spectra of single-step catalysis from (*S*)-1-phenylethylamine to acetophenone.

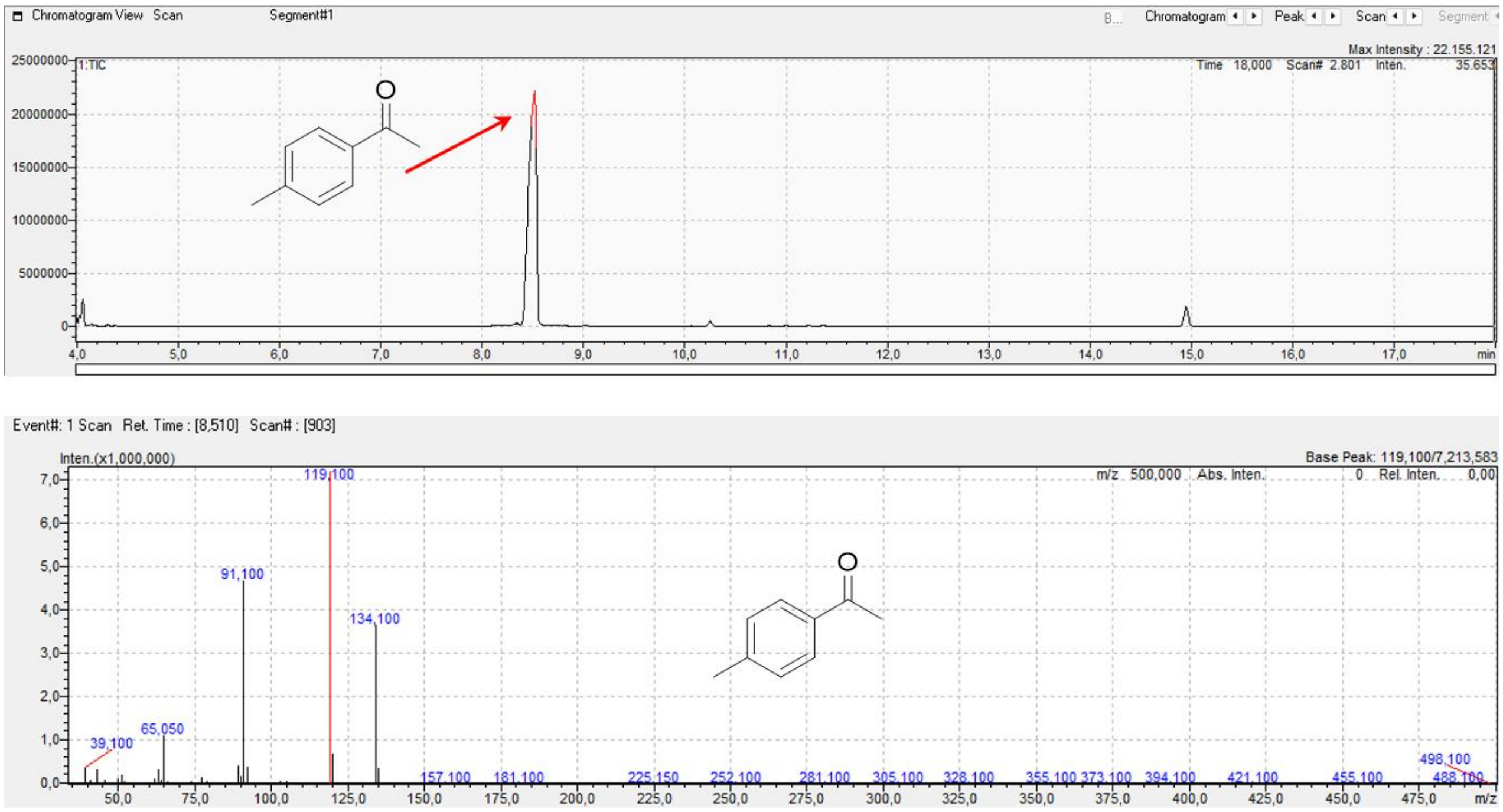

Supplementary Figure 34. GC and MS spectra of single-step catalysis from (*S*)-(-)-1-(p-tolyl)ethylamine to 1-(p-tolyl)ethanol.

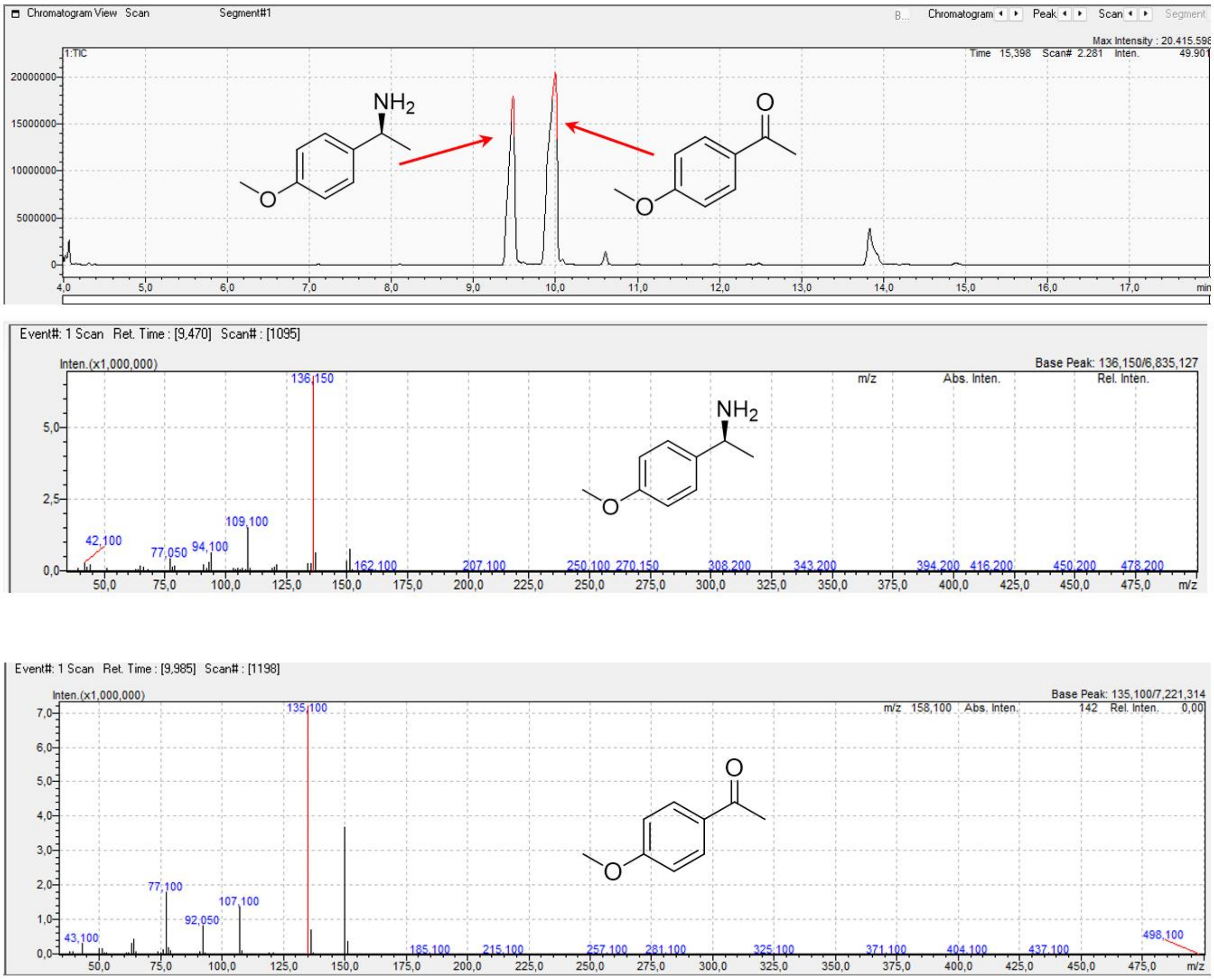

Supplementary Figure 35. GC and MS spectra of single-step catalysis from (*S*)-1-(4-methoxyphenyl)ethylamine to 4'-methoxyacetophenone.

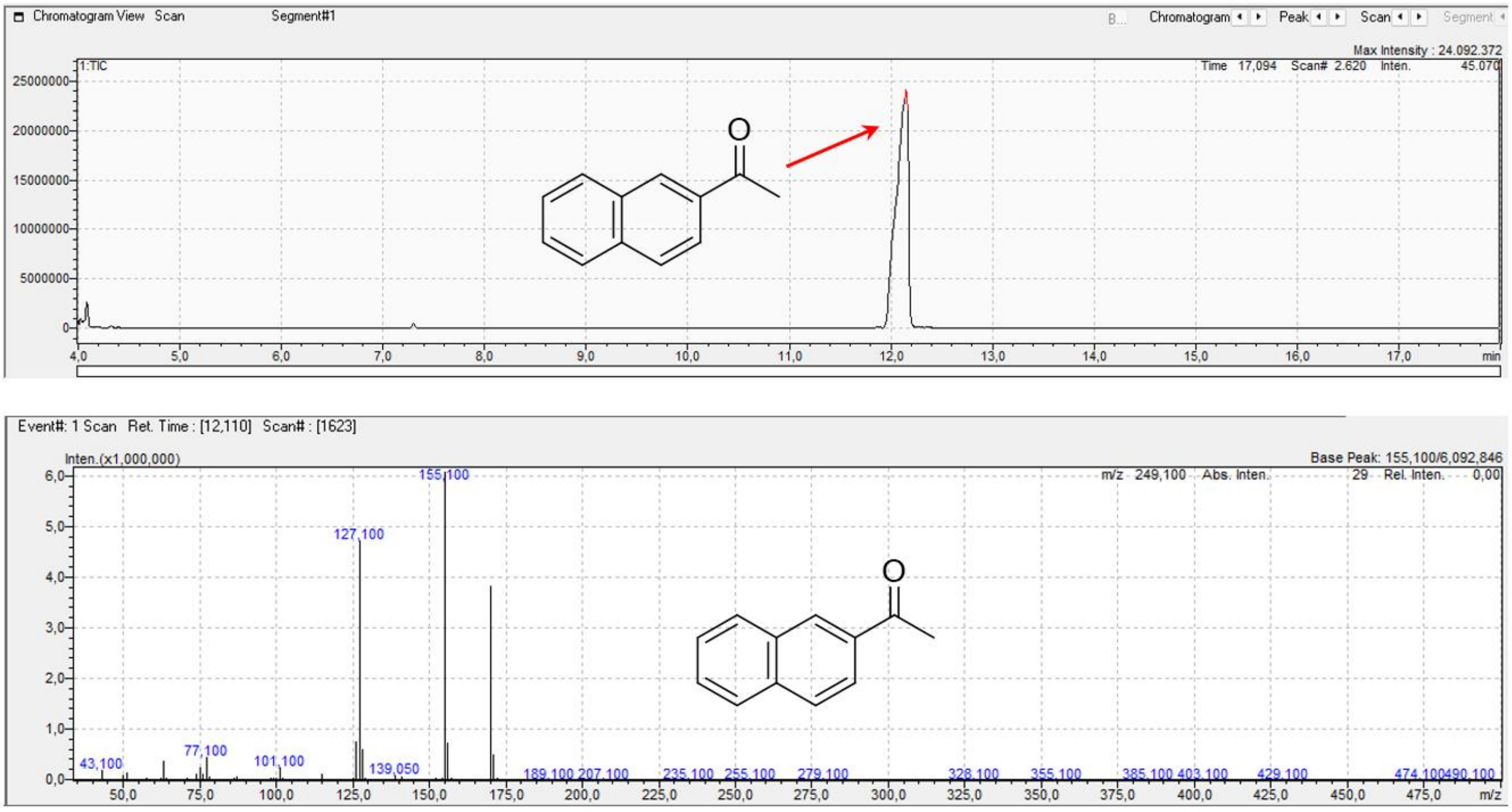

Supplementary Figure 36. GC and MS spectra of single-step catalysis from (*S*)-1-(naphthalen-2-yl)ethanamine to 2-acetonaphthone.

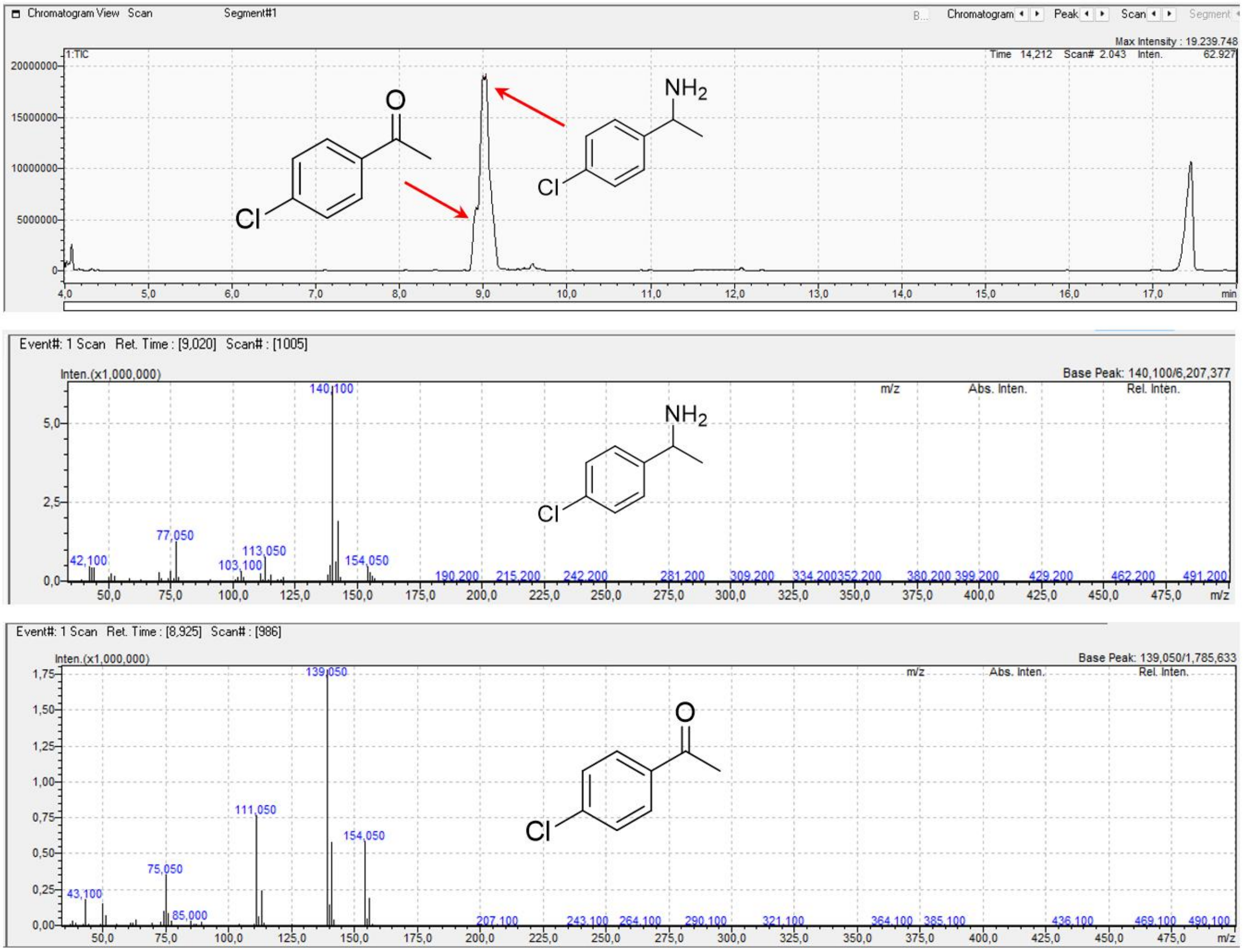

Supplementary Figure 37. GC and MS spectra of single-step catalysis from 1-(4-chlorophenyl)ethylamine to 4′-chloroacetophenone.

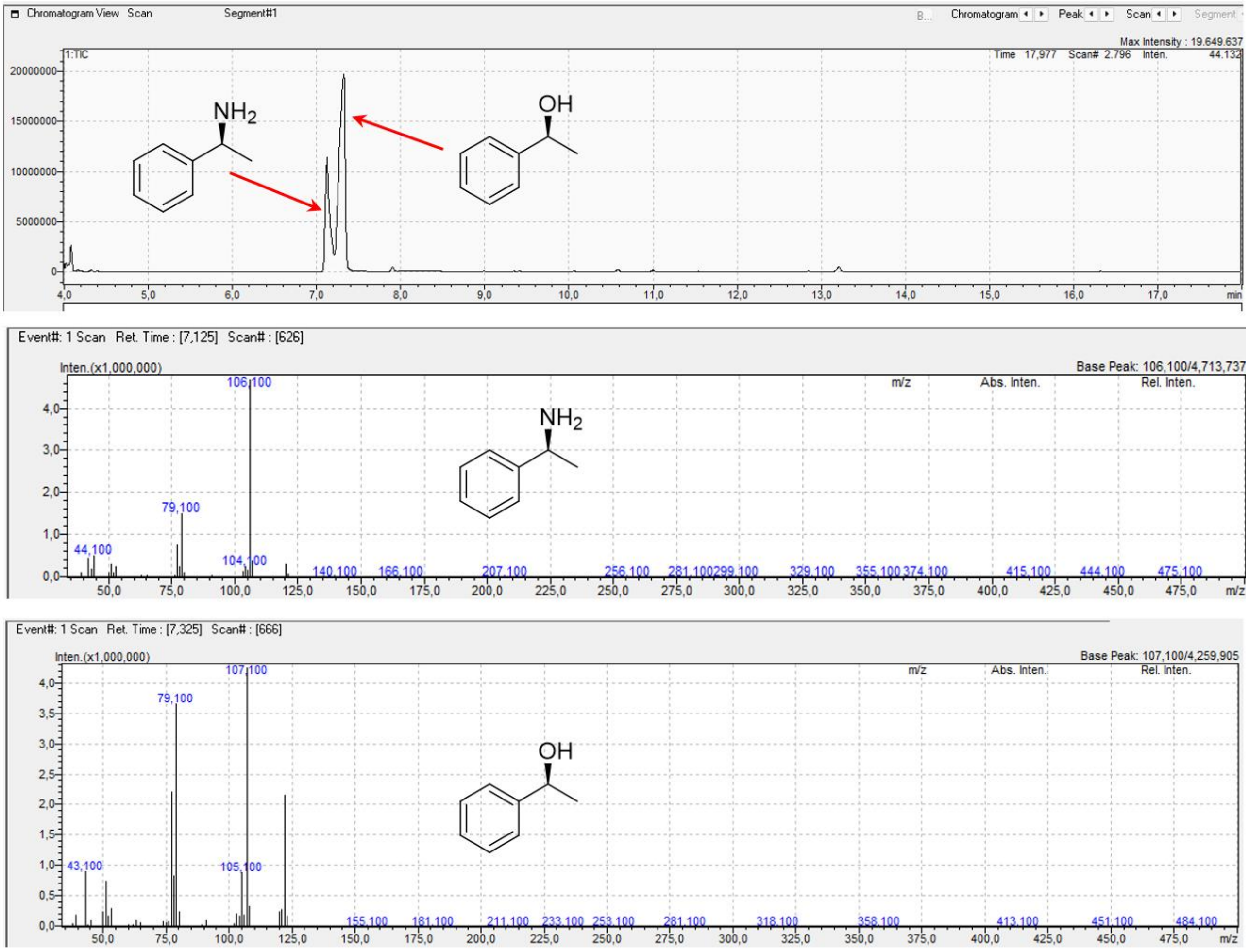

Supplementary Figure 42. GC and MS spectra of cascade catalysis from (*S*)-1-phenylethylamine to (*S*)-1-phenylethanol.

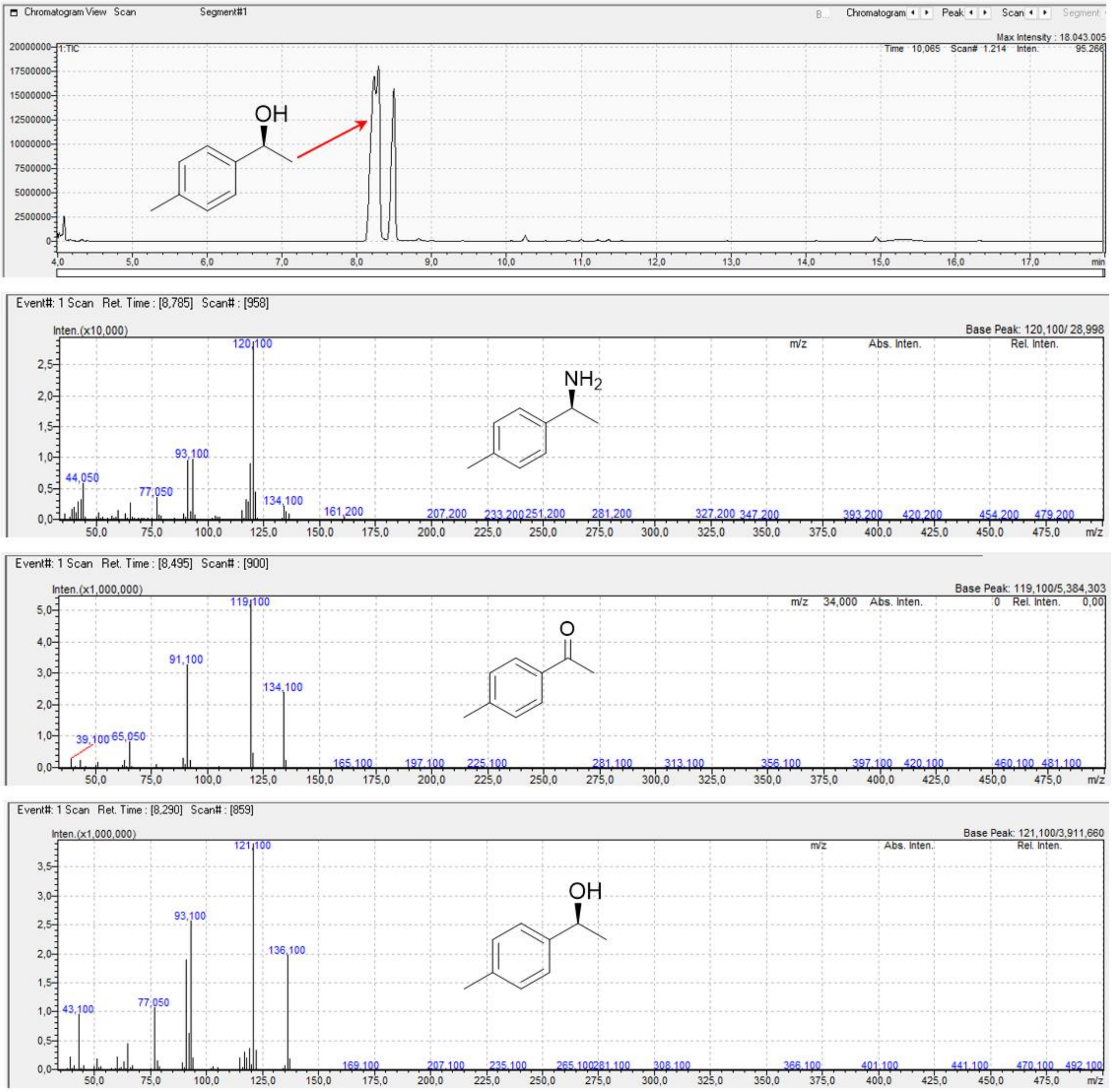

Supplementary Figure 43. GC and MS spectra of cascade catalysis from (*S*)-(-)-1-(p-tolyl)ethylamine to (*S*)-1-(m-tolyl)ethanol.

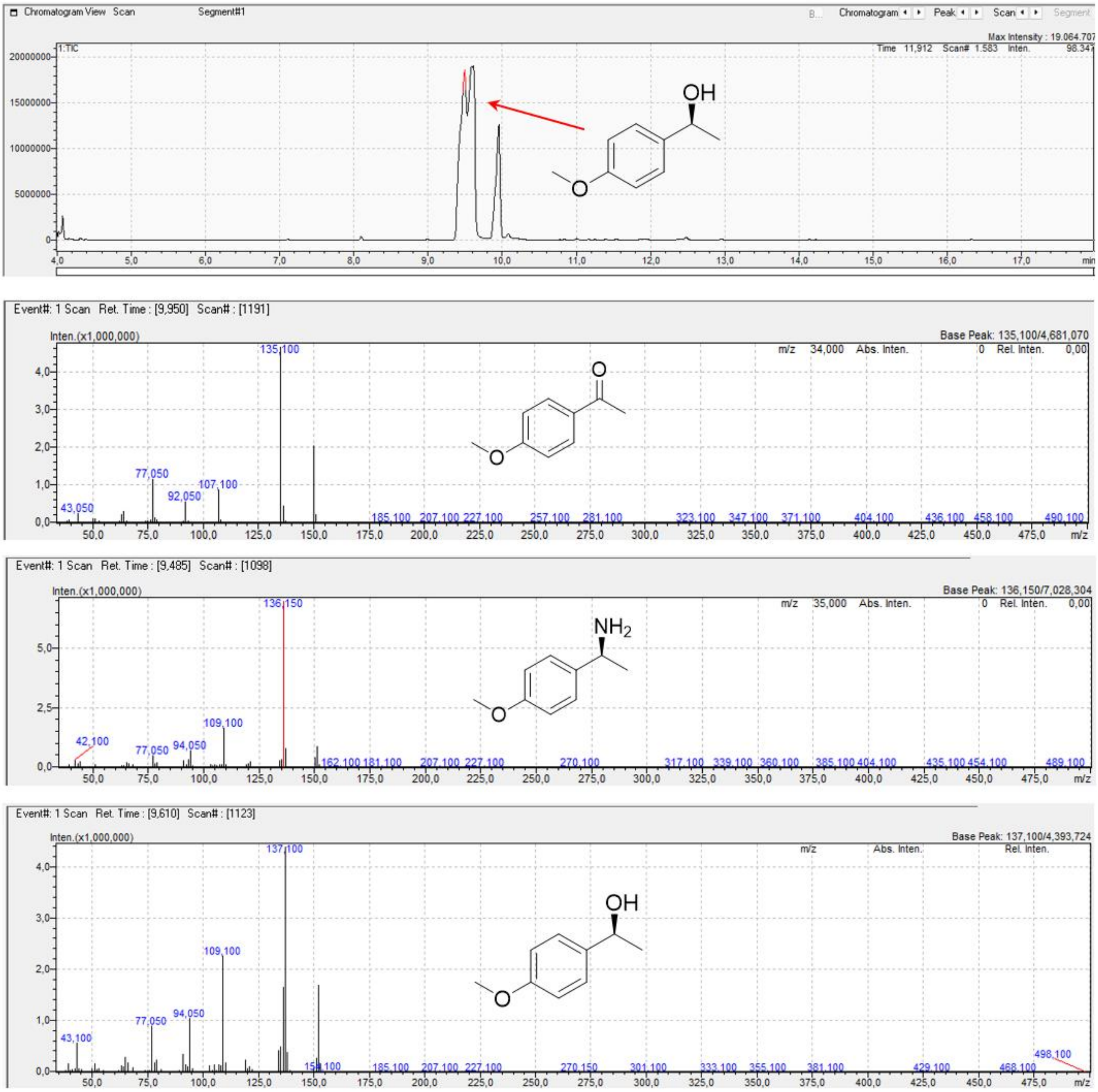

Supplementary Figure 44. GC and MS spectra of cascade catalysis from (*S*)-1-(4-methoxyphenyl)ethylamine to (*S*)-1-(4-methoxyphenyl)ethanol.

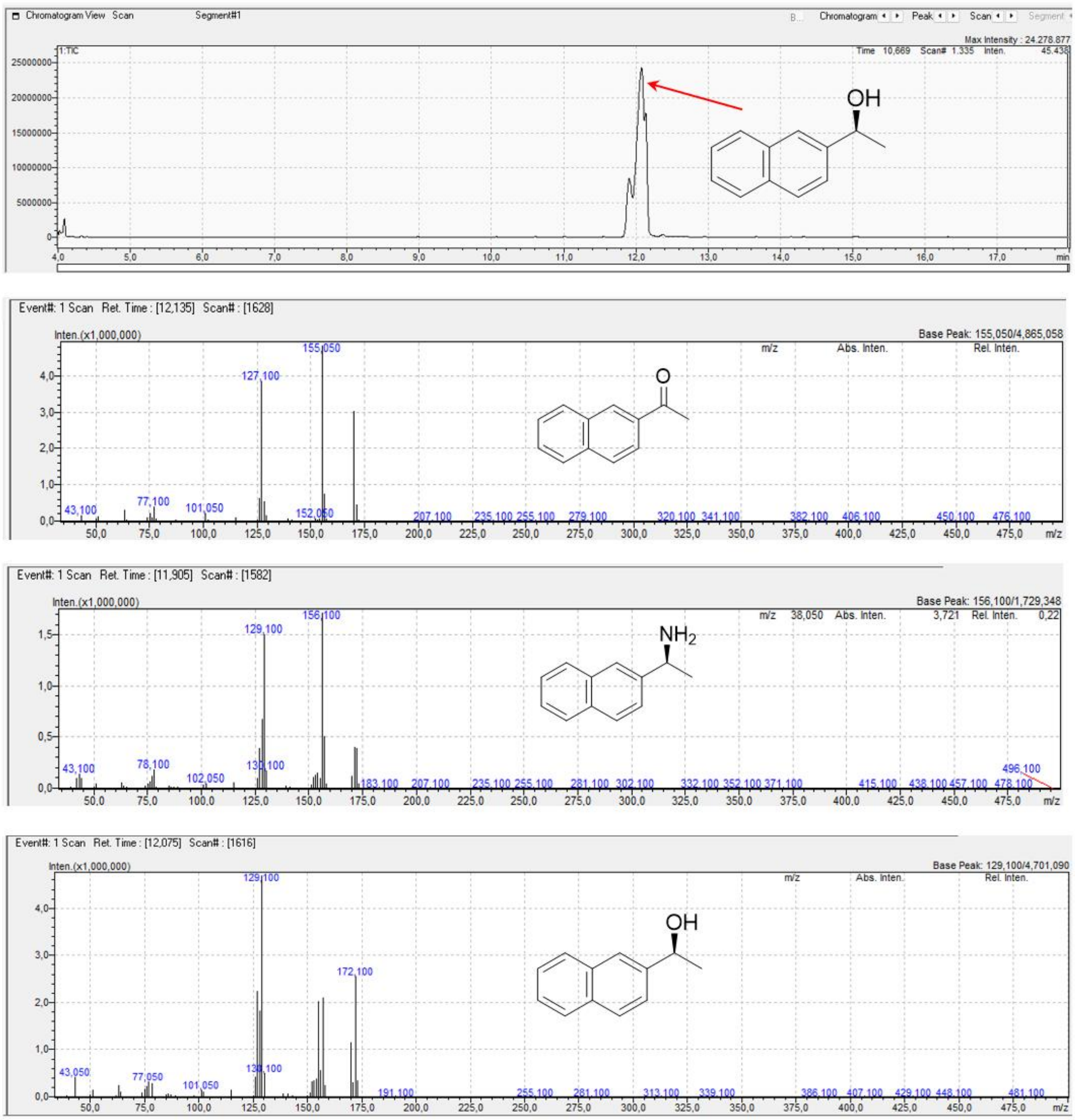

Supplementary Figure 45. GC and MS spectra of cascade catalysis from (*S*)-1-(naphthalen-2-yl)ethanamine to (*S*)-1-(naphthalen-2-yl)ethanol.

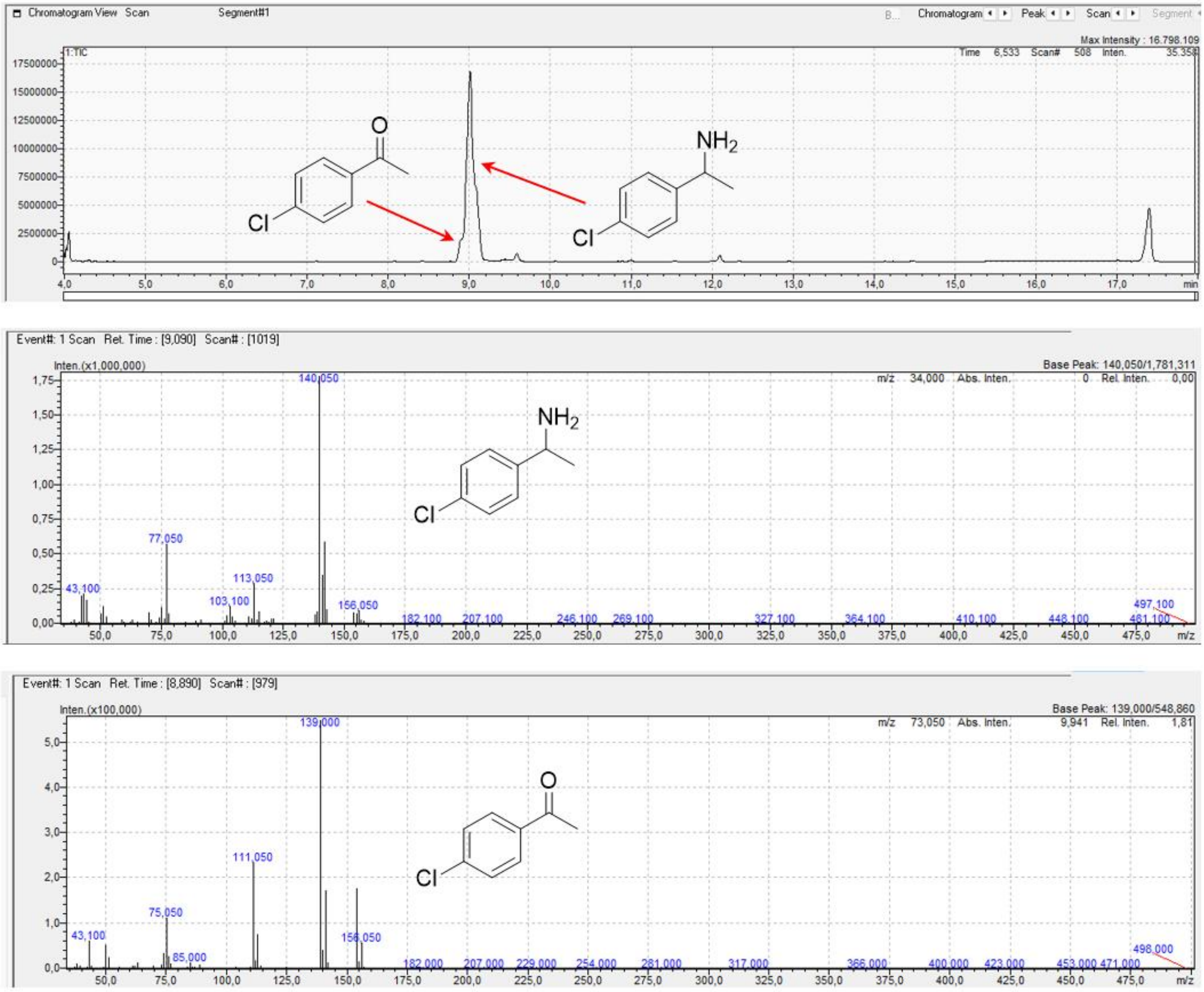

Supplementary Figure 46. GC and MS spectra of cascade catalysis from 1-(4-chlorophenyl)ethylamine to 1-(4-chlorophenyl)ethanol.

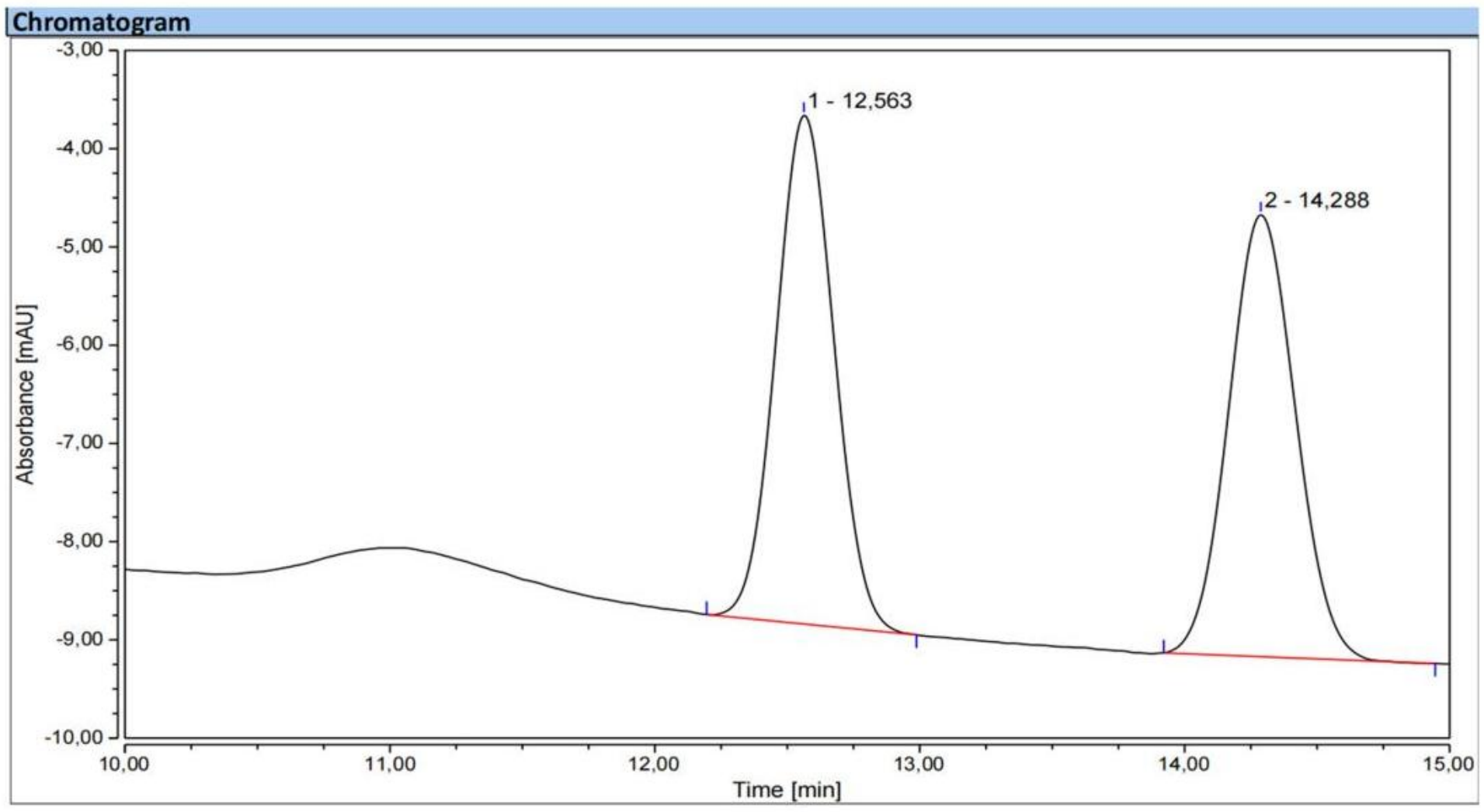

Integration Results

No.	Peakname	RetentionTime min	Area mAU*min	Height mAU	Relative Area %	Rel.Height %	Amount n.a.
1		12,563	1,3189	5,178	50,03	53,52	n.a.
2		14,288	1,3173	4,497	49,97	46,48	n.a.
**Total:**			**2,636**	**9,675**	**100,000**	**100,000**	

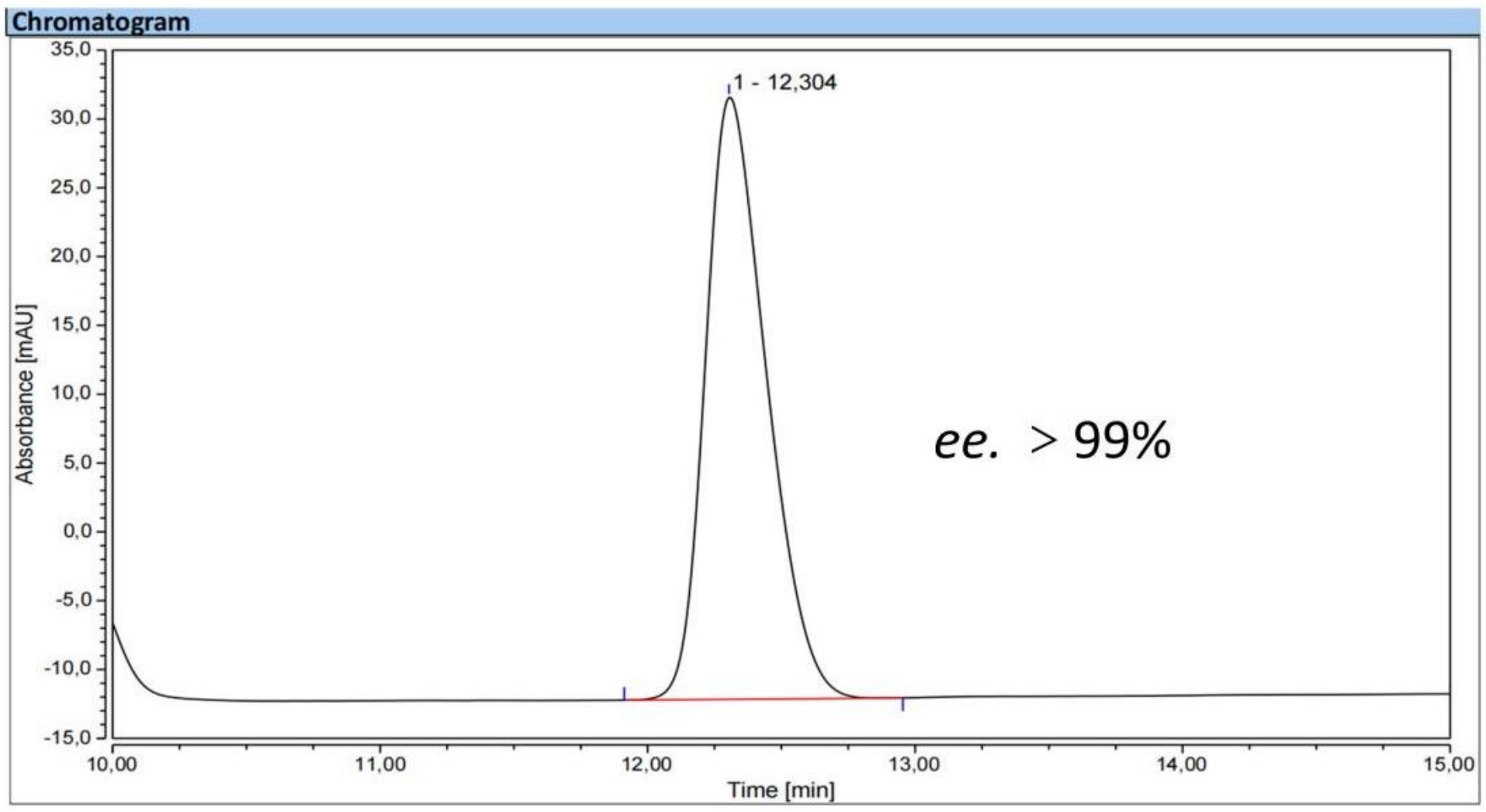

Integration Results

No.	Peakname	RetentionTime min	Area mAU*min	Height mAU	Relative Area %	Rel.Height %	Amount n.a.
1		12,304	11,5886	43,740	100,00	100,00	n.a.
**Total:**			**11,589**	**43,740**	**100,000**	**100,000**	

Supplementary Figure 47. Chiral HPLC chromatogram of crude (*S*)-1-phenylethanol (standard, upper).

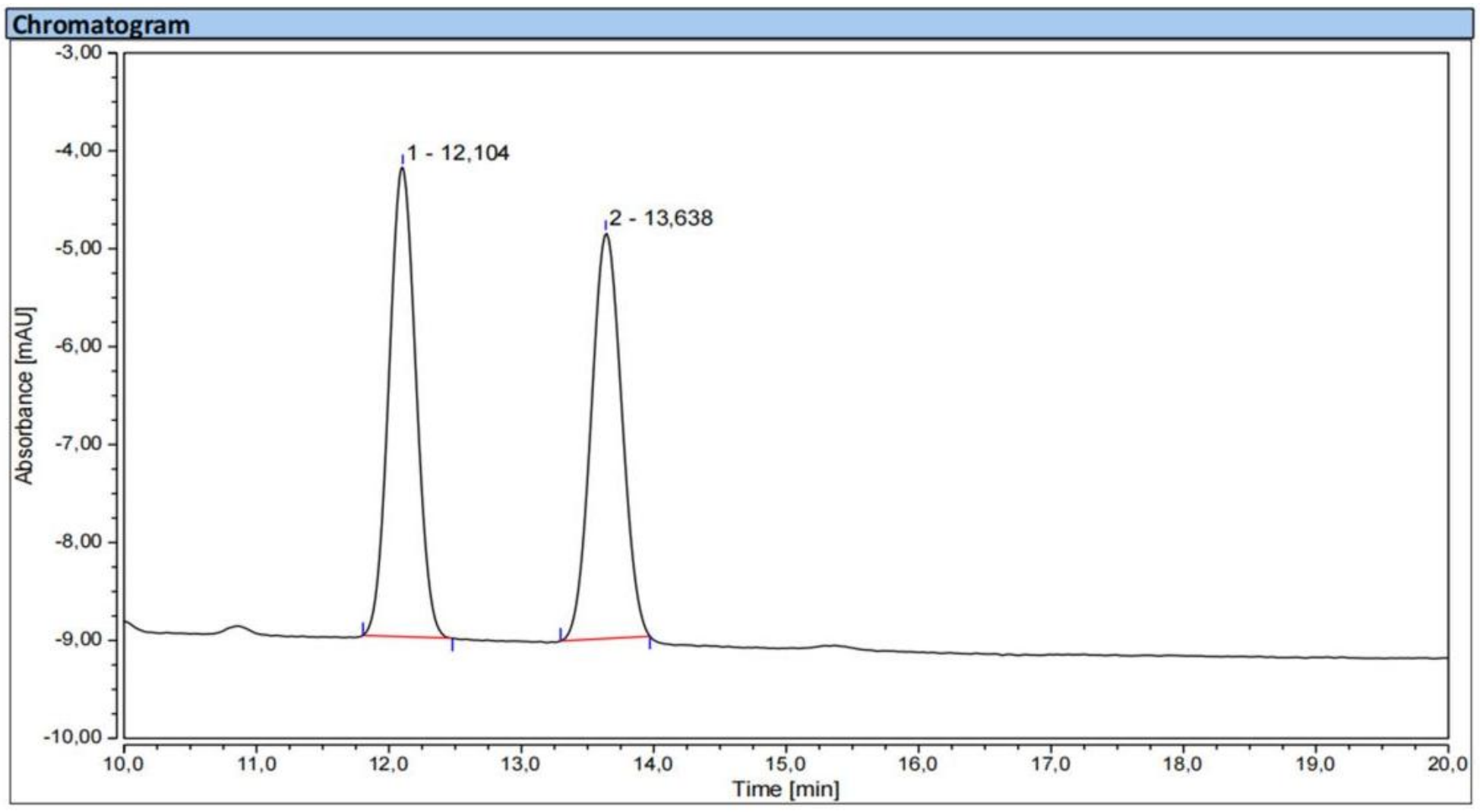

Integration Results

No.	Peakname	RetentionTime min	Area mAU*min	Height mAU	Relative Area %	Rel.Height %	Amount n.a.
1		12,104	1,1276	4,790	50,48	53,65	n.a.
2		13,638	1,1063	4,138	49,52	46,35	n.a.
**Total:**			**2,234**	**8,928**	**100,000**	**100,000**	

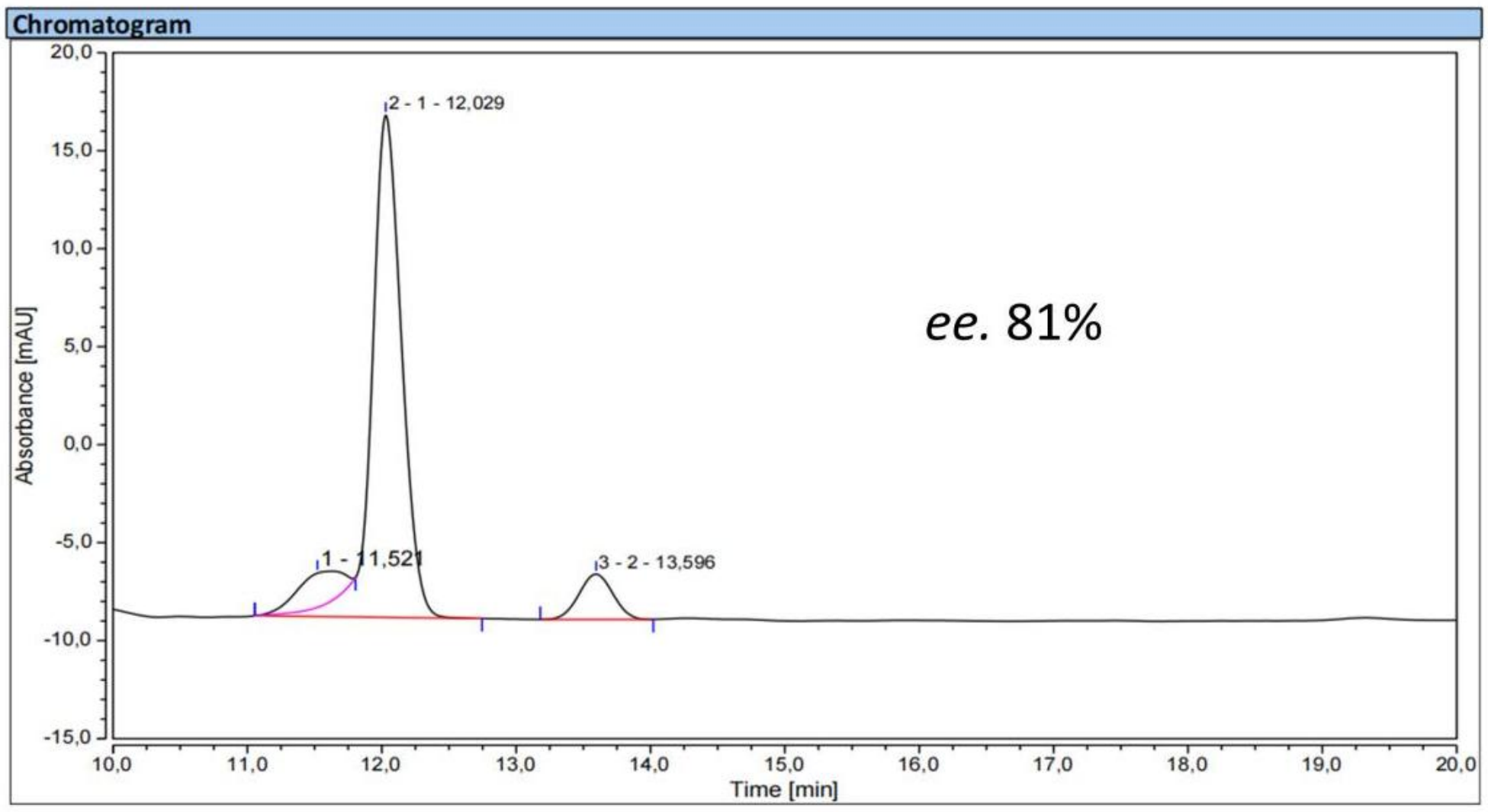

Integration Results

No.	Peakname	RetentionTime min	Area mAU*min	Height mAU	Relative Area %	Rel.Height %	Amount
1		11,521	0,6594	1,744	8,23	5,88	n.a.
2	1	12,029	6,6732	25,623	83,30	86,34	n.a.
3	2	13,596	0,6784	2,311	8,47	7,79	n.a.
**Total:**			**8,011**	**29,678**	**100,000**	**100,000**	

Supplementary Figure 48. Chiral HPLC chromatogram of crude (*S*)-1-(m-tolyl)ethanol (standard, upper).

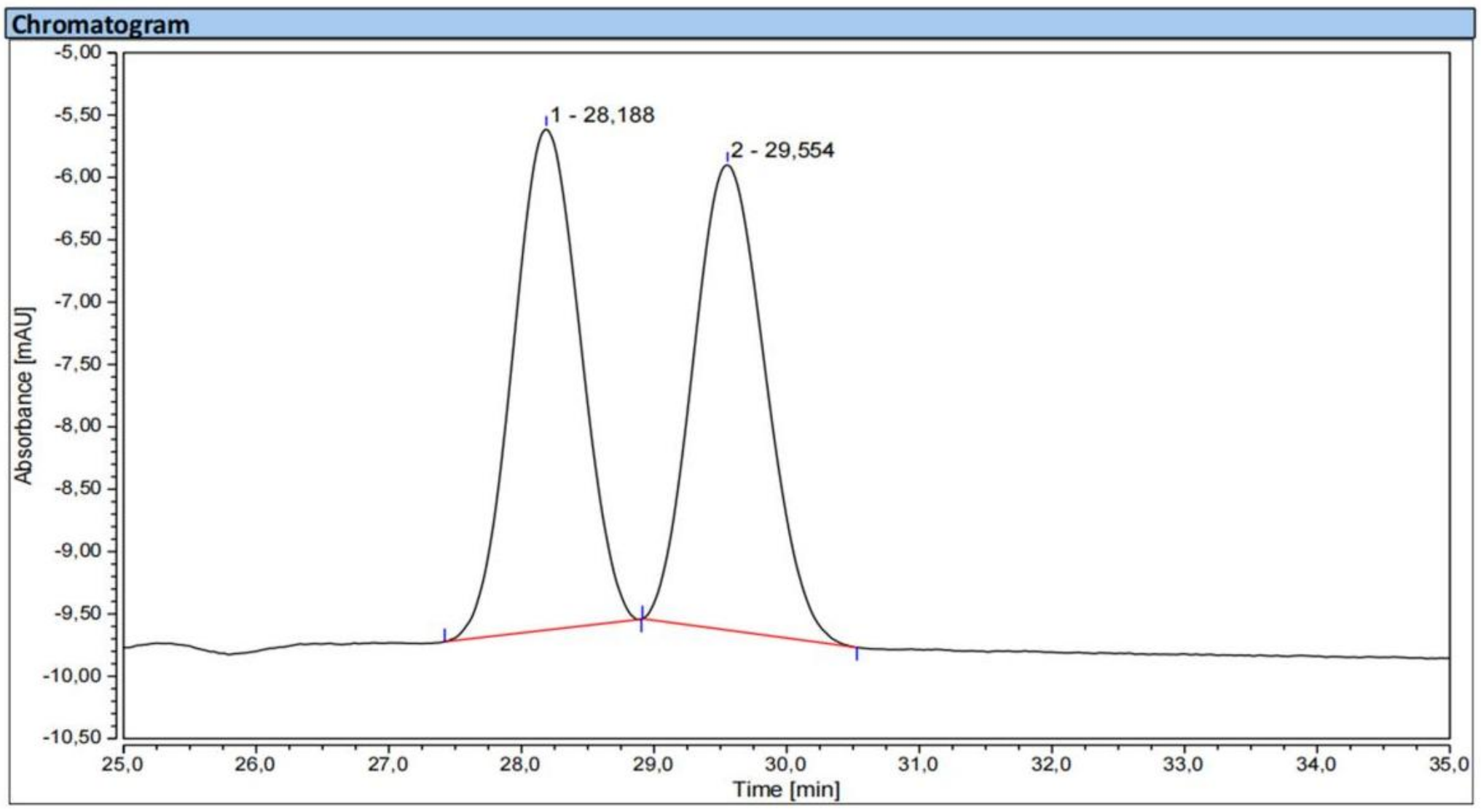

Integration Results

No.	Peakname	RetentionTime min	Area mAU*min	Height mAU	Relative Area %	Rel.Height %	Amount n.a.
1		28,188	2,3666	4,016	50,14	51,85	n.a.
2		29,554	2,3538	3,729	49,86	48,15	n.a.
**Total:**			**4,720**	**7,745**	**100,000**	**100,000**	

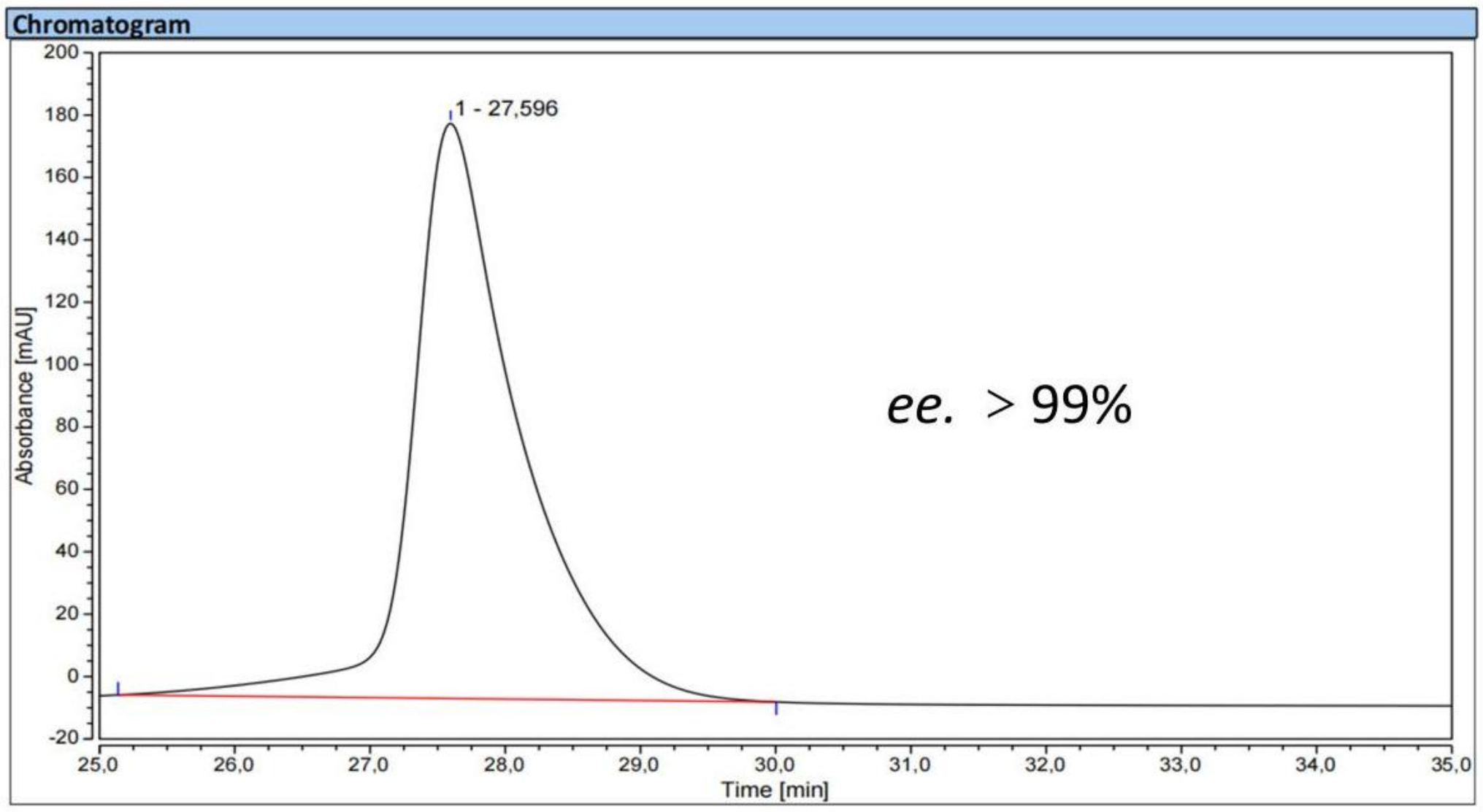

Integration Results

No.	Peakname	RetentionTime min	Area mAU*min	Height mAU	Relative Area %	Rel.Height %	Amount n.a.
1		27,596	169,4502	184,340	100,00	100,00	n.a.
**Total:**			**169,450**	**184,340**	**100,000**	**100,000**	

Supplementary Figure 49. Chiral HPLC chromatogram of crude (*S*)-1-(4-methoxyphenyl)ethanol (standard, upper).

Chromatogram

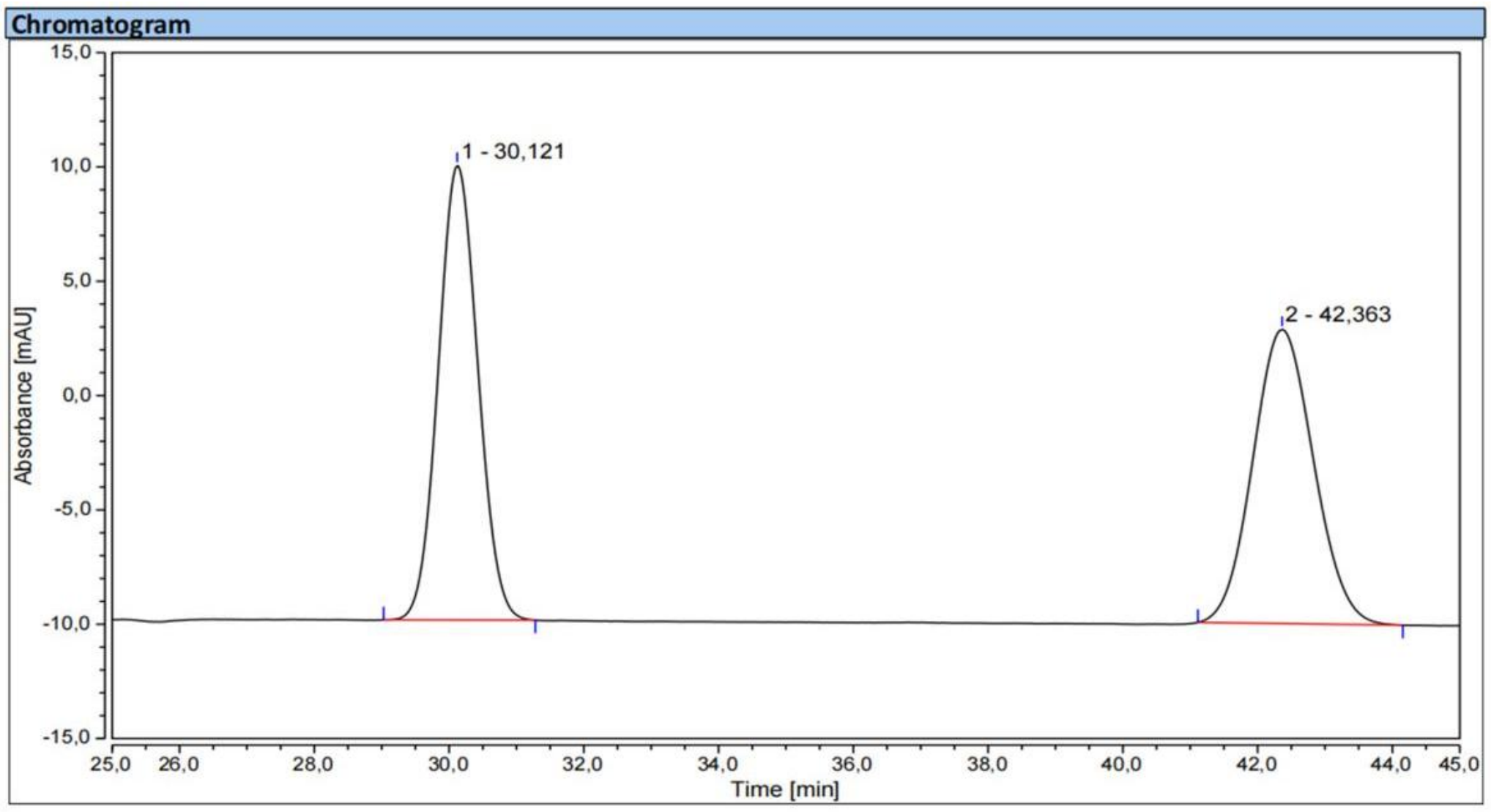

Integration Results

No.	Peakname	RetentionTime min	Area mAU*min	Height mAU	Relative Area %	Rel.Height %	Amount n.a.
1		30,121	13,5399	19,877	50,22	60,71	n.a.
2		42,363	13,4222	12,861	49,78	39,29	n.a.
**Total:**			**26,962**	**32,738**	**100,000**	**100,000**	

Chromatogram

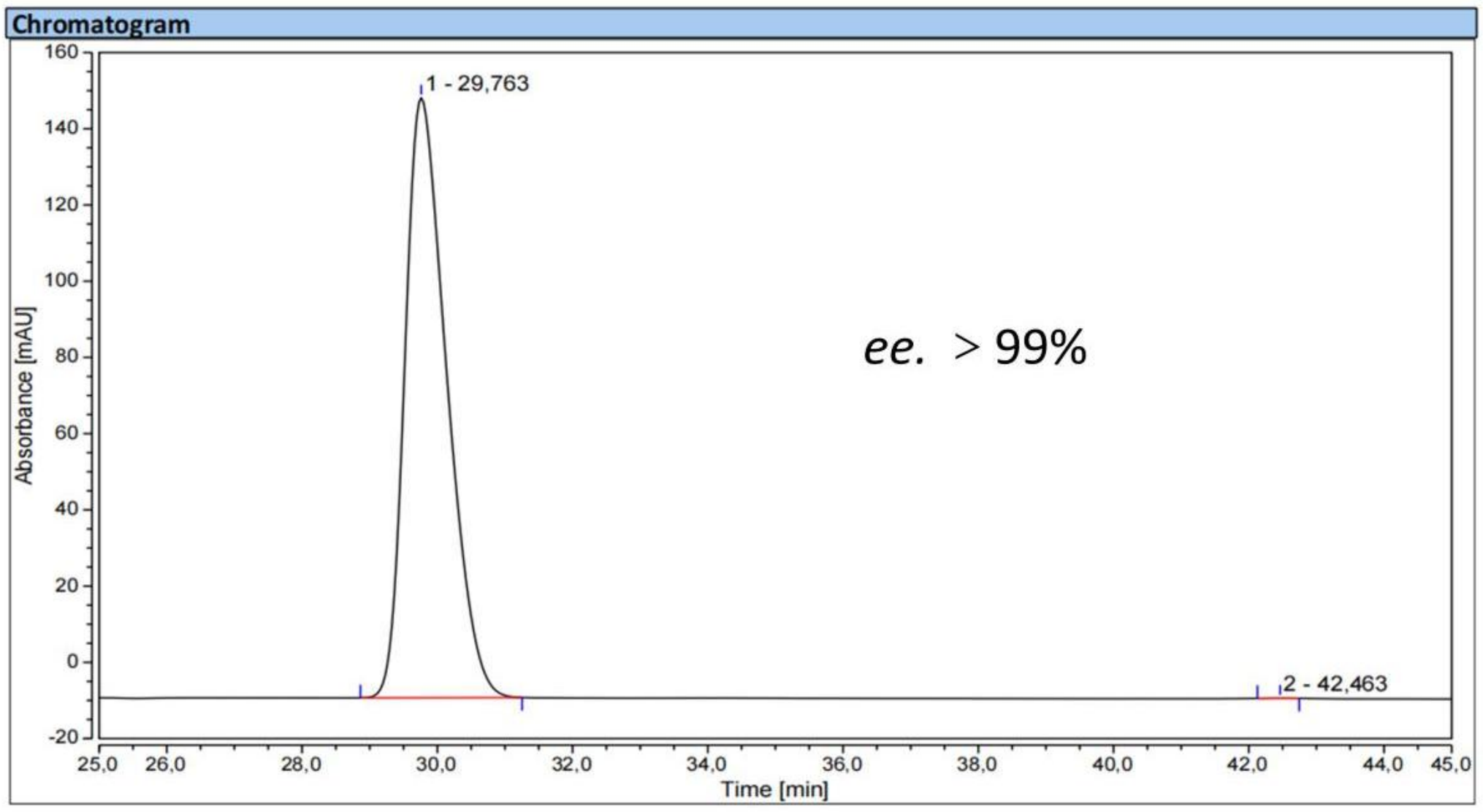

Integration Results

No.	Peakname	RetentionTime min	Area mAU*min	Height mAU	Relative Area %	Rel.Height %	Amount n.a.
1		29,763	112,5268	157,420	99,98	99,97	n.a.
2		42,463	0,0208	0,054	0,02	0,03	n.a.
**Total:**			**112,548**	**157,475**	**100,000**	**100,000**	

Supplementary Figure 50. Chiral HPLC chromatogram of crude (*S*)-1-(naphthalen-2-yl)ethanol (standard, upper).

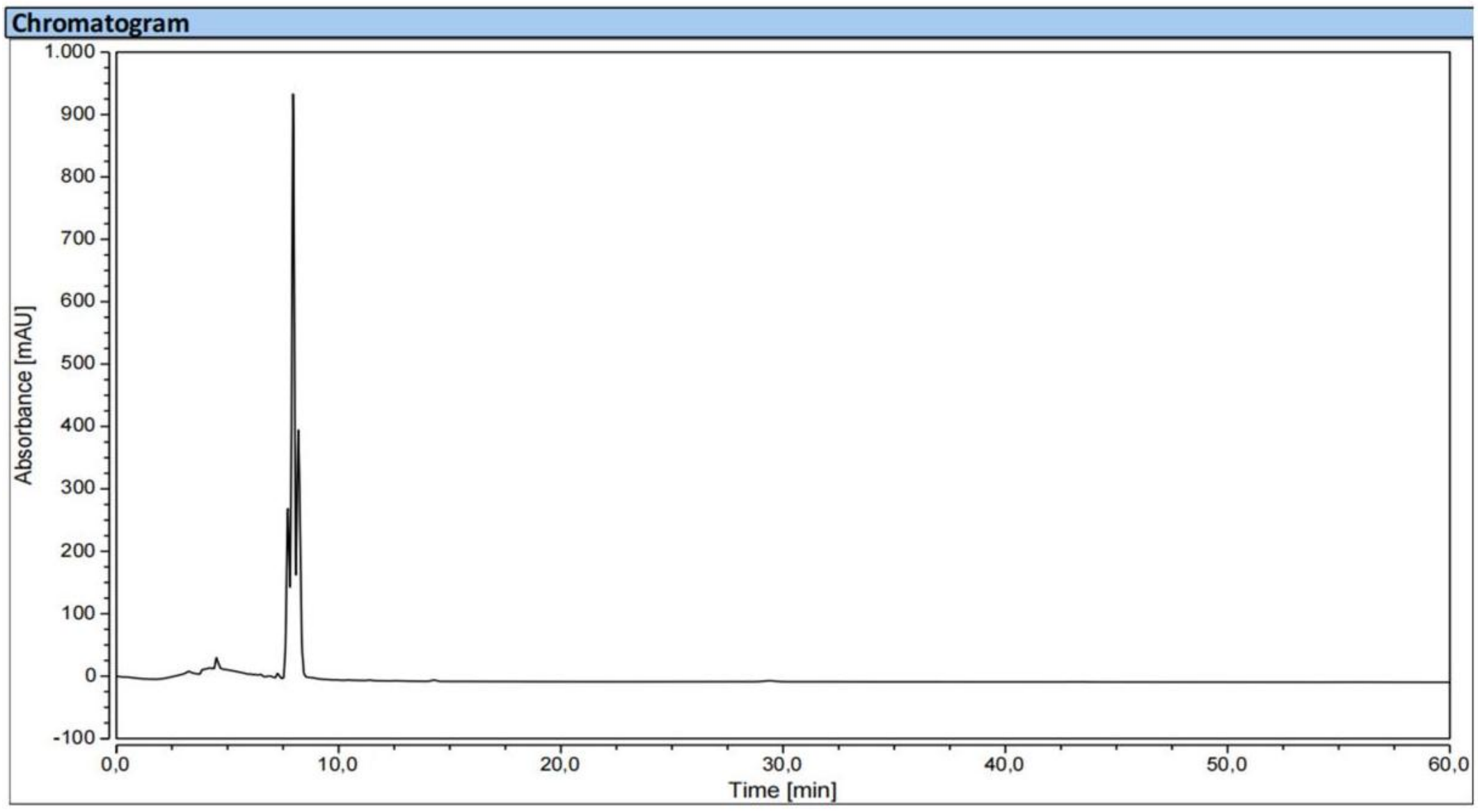

Integration Results

No.	Peakname	RetentionTime min	Area mAU*min	Height mAU	Relative Area %	Rel.Height %	Amount n.a.
**Total:**			**0,000**	**0,000**	**0,000**	**0,000**	

Supplementary Figure 51. Chiral HPLC chromatogram of crude 1-(4-chlorophenyl)ethanol.

Supplementary Table 2. Single step reactions for the production of ketone using the enzyme ATA.

Entry	Substrate	Product	*Conv.* (%)	*ee.* (%)
1	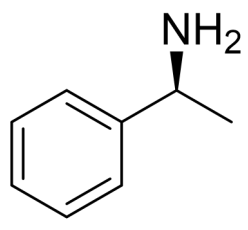	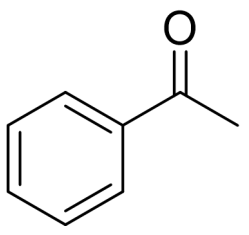	77	-
2	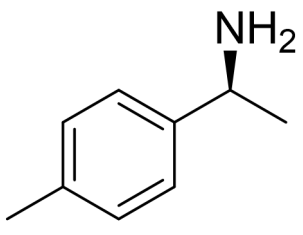	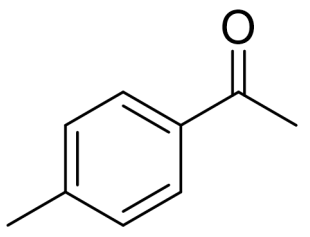	93[a]	-
3	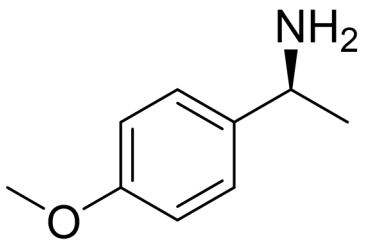	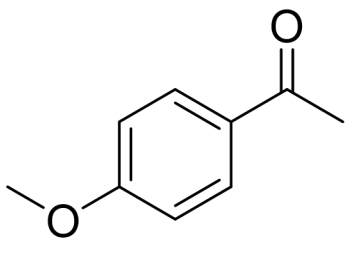	61[a]	-
4	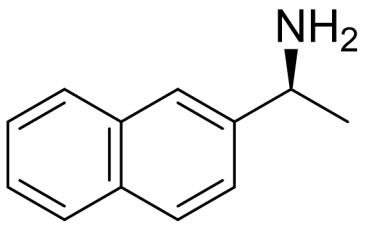	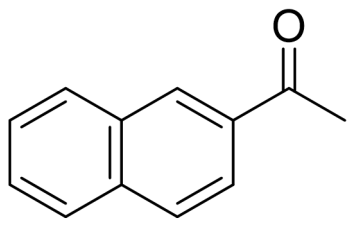	97[a]	-
5	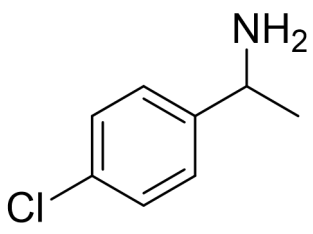	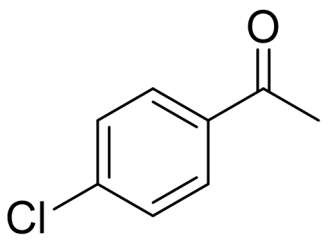	28[a]	-

[a]Conversion determined by ^{1}H NMR. The conversion of acetophenone determined by GC.

Supplementary Table 3. Cascade reactions for the production of secondary alcohols using enzyme ATA and RR ADH.

**Entry**	**Substrate**	**Product**	***Conv.* (%)**	***ee.* (%)**
1	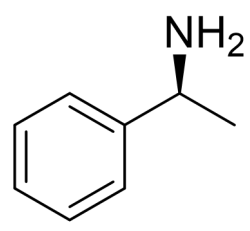	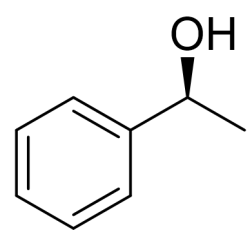	19	99
2	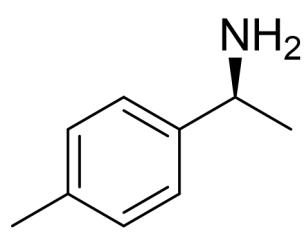	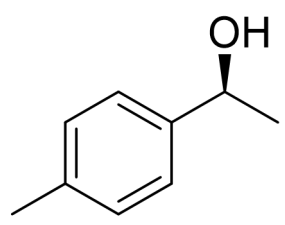	71[a]	81
3	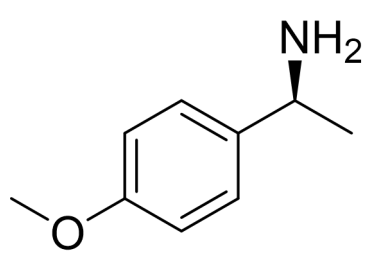	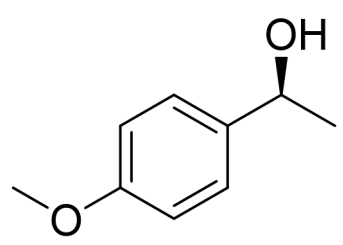	93[a]	99
4	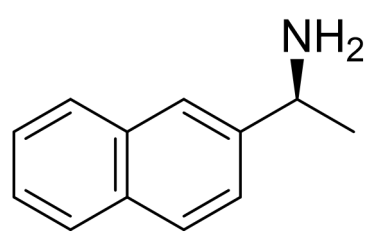	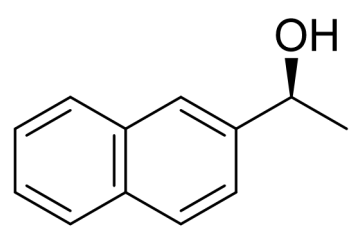	30[a]	99
5	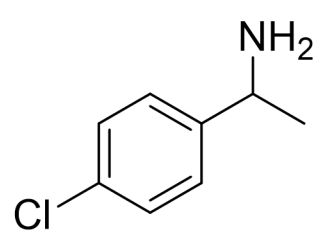	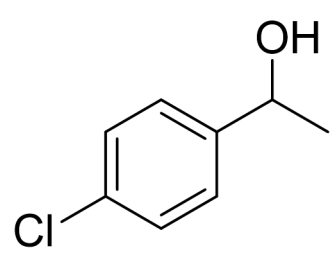	5[a]	-

[a]Conversion determined by ^{1}H NMR. The conversion of (*S*)-1-phenylethanol was determined by GC; *ee* values were determined by chiral HPLC.

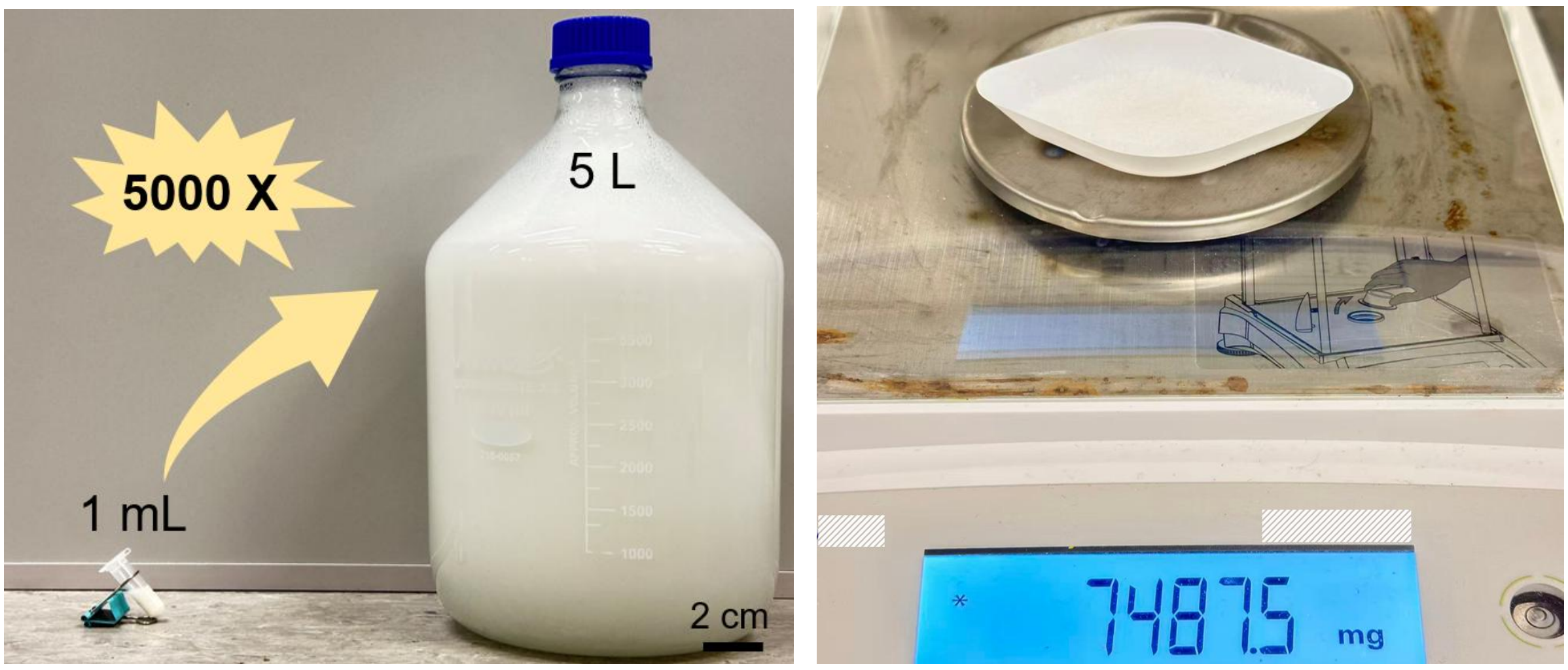

Figure 6. (i) 5 L of the enlarged emulsion was prepared and the catalysis was performed (left). (j) Approximately 7.5 g of purified benzoin was obtained (right).

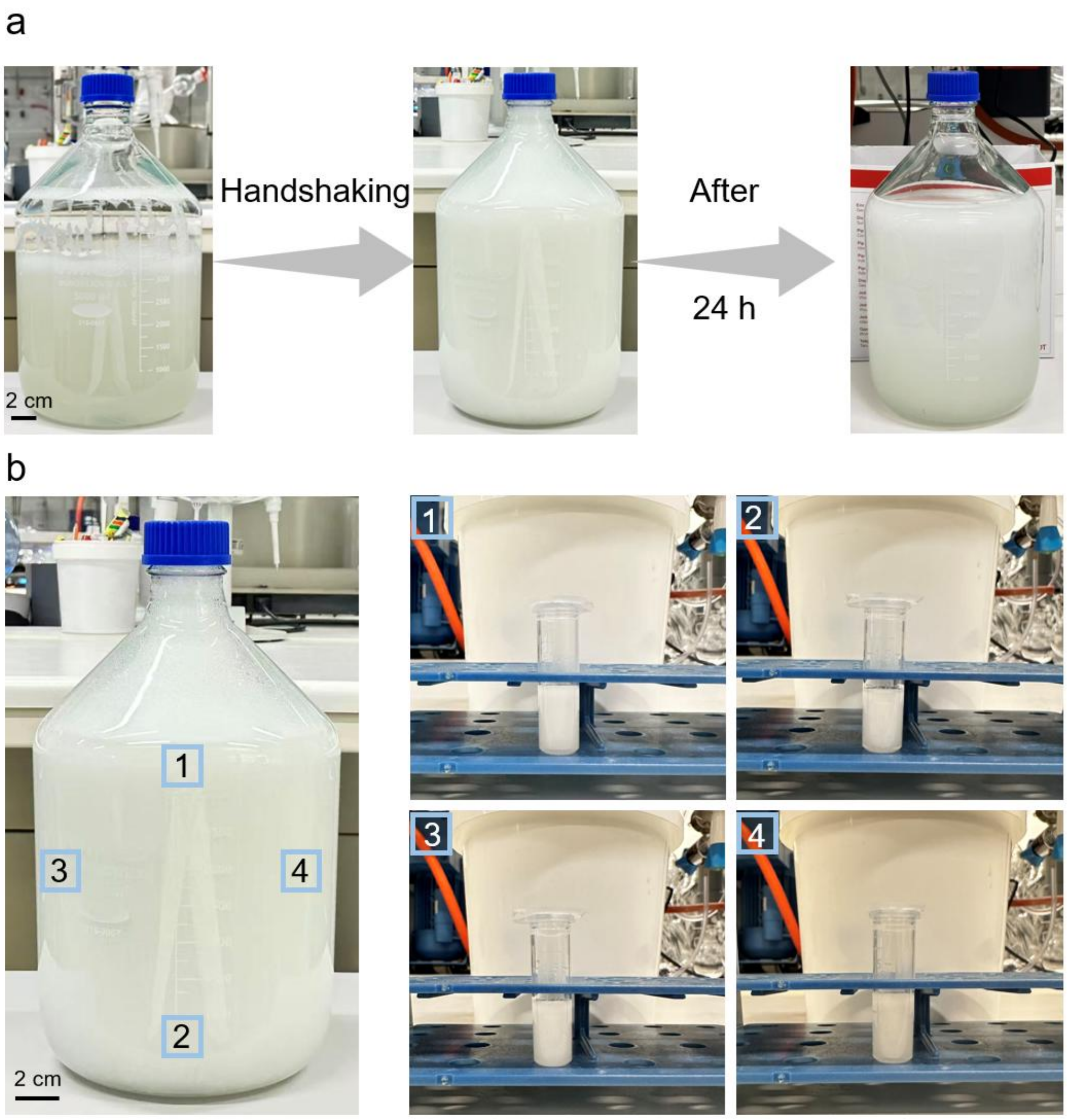

Supplementary Figure 54. (a) The 5 L scale-up experiment was further conducted, though it required a longer handshaking time. (b) Take 1 mL of emulsion from different positions within the 5 L emulsion system to observe the emulsion's spatial stability.

Reviewer #2:

The authors have good background on chemical modified bacterial cells for biocatatlysis as well as integrating photocatalysis with living cell biocatalyisis. The present manuscript reports a conceptually novel and technically elegant strategy that transforms living *Escherichia coli* into programmable amphiphilic colloidal catalysts by combining β-cyclodextrin-based surface engineering with oil-based photocatalysts. The resulting hybrid system autonomously self-assembles into Pickering emulsions, creating efficient interfacial microreactors that accelerate a broad range of enzymatic, chemoenzymatic, and photoenzymatic reactions. On-demand, selective recycling of either the living cells or the synthetic photocatalyst—enabled by reversible host–guest interactions—represents a clear advance over existing Pickering emulsion biocatalysis and living-cell immobilization approaches, where co-immobilization typically hinders selective regeneration. Overall, the work establishes a versatile, modular, and sustainable platform with clear implications for biocatalysis, supramolecular chemistry, and engineered living materials.

Please highlight the progresses of the present work comparison to your previous works and check the following points.

Reply: Many thanks for your positive comments and valuable suggestions on our work. We have made the revisions to improve the manuscript accordingly.

Comment 1: The conclusion on controllable grafting is currently supported only by fluorescence intensity (Figure 2b), which is not fully convincing. Please provide additional data to substantiate this claim.

Reply: Thank you for the constructive comments. We agree that fluorescence intensity alone may not be sufficiently quantitative to support the conclusion of controllable grafting density. Therefore, we have performed additional thermogravimetric analysis (TGA) and zeta potential measurements to provide more quantitative evidence. Specifically, we conducted TGA on native cells and surface-modified cells (cell@OBC) prepared under a series of EDC/NHS coupling conditions (low to high). As shown in **Figure 2d** and **Supplementary Figure 9**, the modified cells exhibit an extra mass loss compared with native cells at high temperature, which can be attributed to the increased amount of grafted organic layer on the cell surface. Importantly, this additional mass loss increases monotonically as the EDC/NHS concentrations are increased, with the net differences (vs. native cells) of 2.23%, 5.47%, 7.41%, 8.99%, and 11.44% for progressively stronger coupling conditions, respectively. This trend provides direct and quantitative support that the grafting amount can be systematically tuned by the coupling agent. Meanwhile, to further confirm controllable surface modification, we measured the zeta potential of native cells and cell@OBC. From the new data (**Figure 2e**), the surface potential shifts from strongly negative (native cells, ~ − 45 mV) to progressively less negative values with increasing coupling strength, approaching near-neutral values at the highest grafting condition. This systematic charge modulation is consistent with increased surface functionalization and provides an extra confirmation of the tunable grafting process. Additionally, we further noticed that simple mixing of cells with the corresponding surface components (without covalent coupling) does not produce a comparable shift in zeta potential across concentrations (**Supplementary Figure 10**), indicating that the observed charge modulation primarily due to actual surface grafting rather than weak, non-specific adsorption.

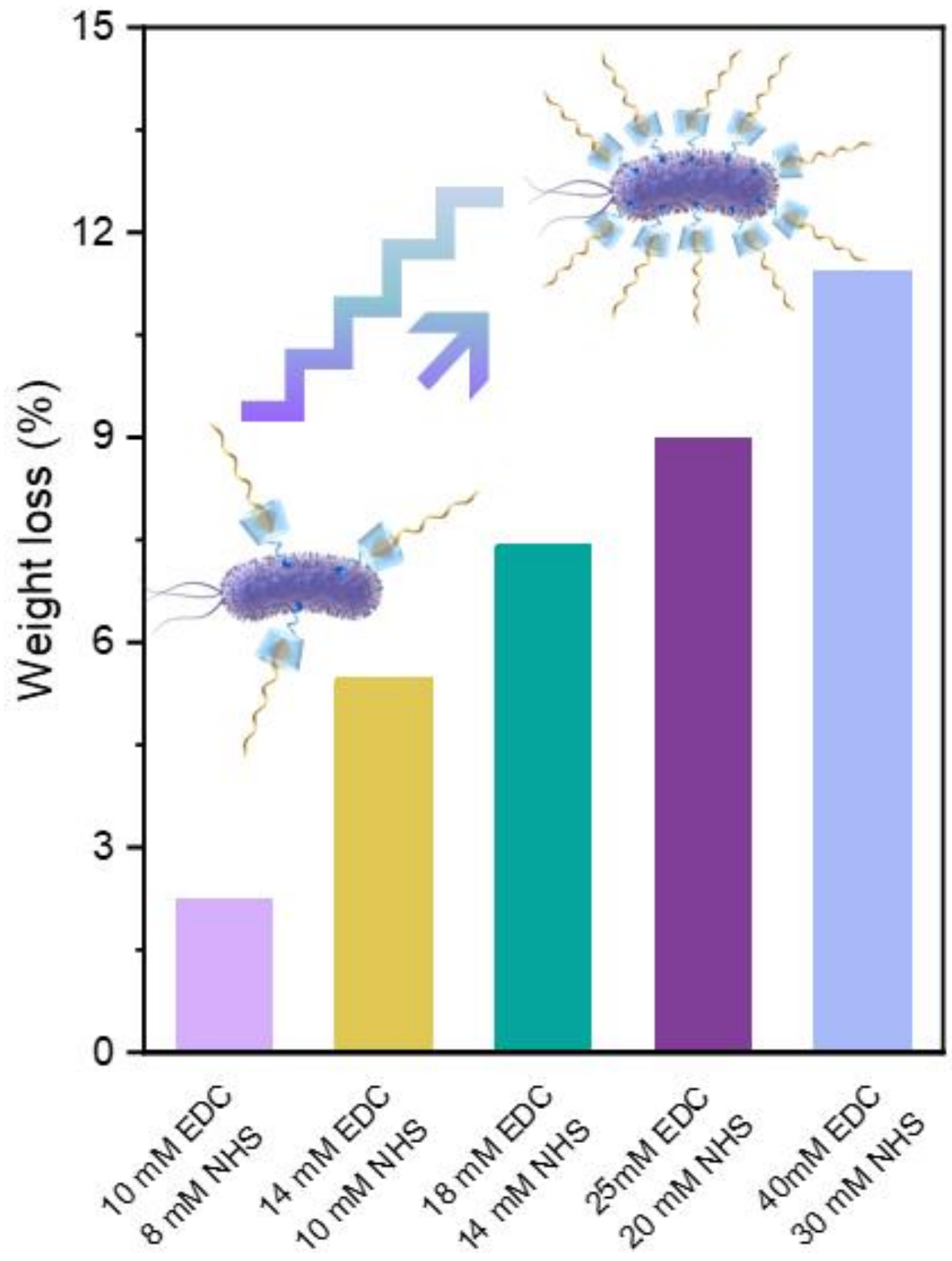

Figure 2d. With increasing amounts of coupling agent, the weight loss of cell@OBC monotonically increased over the 0-800°C range compared to the native cells.

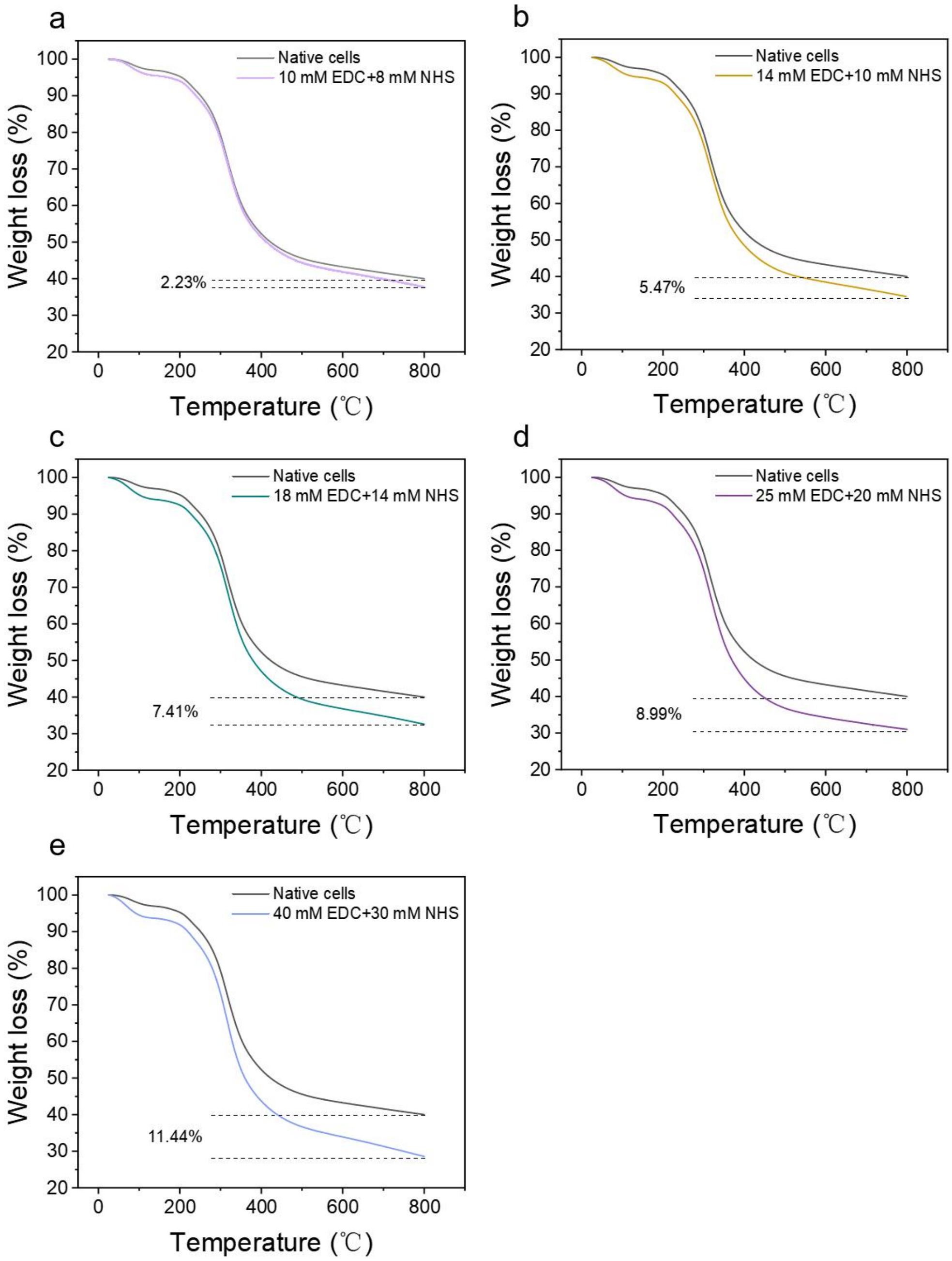

Supplementary Figure 9. TGA curves of (a) native cells and cell@OBC modified with different amount of coupling agent (b-e).

Comment 2: A small amount of DMSO is used during cell surface modification, whereas the organic solvent resistance experiments employ toluene. To validate the general robustness of cell@OBC, please include resistance tests against additional organic solvents.

Reply: Thank you for your valuable suggestion. To address this concern, we have systematically evaluated the stability of cell@OBC in a broad panel of organic solvents with distinct polarity. Native cells and Cell@OBC, which overexpress BAL were first dispersed in KPI buffer and subsequently contacted with an equal volume of organic solvent (water/organic volume ratio = 50:50). The retaining activity was quantified after exposure for 1 h. As shown in **Supplementary Figure 12**, cell@OBC consistently exhibits substantially higher retained activity than native cells in DMF and isopropanol. In contrast, native cells suffer from rapid and severe deactivation under the same exposure conditions, typically retaining only ~20-25% activity after 1 h. These results indicate that the OBC-modified interface provides a protective microenvironment that mitigates solvent-induced deactivation. Notably, the degree of protection depends on solvent properties. In highly polar, aprotic solvents such as DMSO, both cell@OBC and native cells maintain relatively high activity, consistent with the good intrinsic biocompatibility of this solvent. Because DMSO is a known biocompatible solvent for cells (Heri, *et. al*., New J. Chem., 2018,42, 13225-13228; U.S. Department of Health and Human Services Food and Drug Administration (FDA). Q3C—Tables and List Guidance for Industry (2017)). Additionally, unlike most other solvents, methanol leads to similarly low retaining activity for both cell@OBC and native cells after 1 h exposure. This behavior is consistent with the well-documented intrinsic antimicrobial and protein-denaturing properties of methanol. As a highly polar protic solvent, methanol can rapidly penetrate bacterial membranes, disrupt membrane integrity, and directly interfere with intracellular enzyme structures, resulting in non-selective loss of cellular activity (Bennett, R.K. et *al.* Front Microbiol. 12, 638426. 2021). Overall, the observed solvent tolerance demonstrates the robustness of the cell@OBC architecture and supports its suitability for biphasic and emulsion-based biocatalytic applications involving diverse organic media.

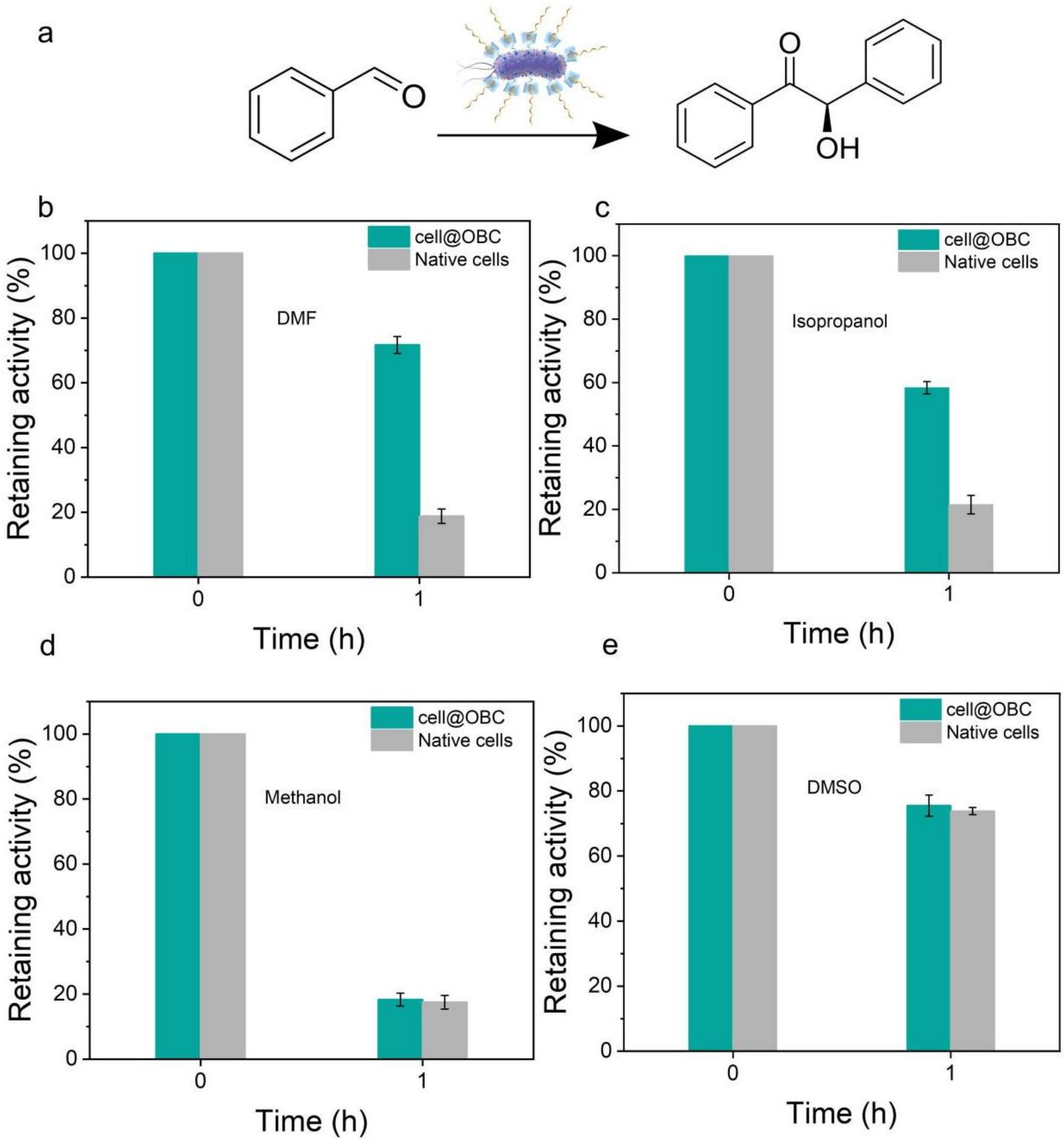

Supplementary Figure 12 General robustness of native cells and cell@OBC in different solvents. The retaining activity of intracellular enzymes is assessed through the BAL-induced benzoin condensation reaction (a). (b) Stability of the intracellular enzyme BAL in a water-DMF mixture (v/v = 50:50). (c) Stability of the intracellular enzyme BAL in a water-isopropanol mixture (v/v = 50:50). (d) Stability of the intracellular enzyme BAL in a water-methanol mixture (v/v = 50:50). (e) Stability of the intracellular enzyme BAL in a water-DMSO mixture (v/v = 50:50). The error bars represent the standard deviations of three parallel measurements, n = 3.

Comment 3: It is unclear whether enrichment of oil-based photocatalysts (OBC) and β-CD on the cell surface affects cell charge properties. This aspect appears to be missing and should be addressed, ideally with supporting data or discussion.

Reply: Thank you for your questions and suggestions. To clarify this, we have performed additional zeta potential measurements under varying modification conditions. As shown in **Figure 2e**, native cells exhibit a strongly negative surface potential (~ −45 mV). After EDC/NHS-mediated coupling, the zeta potential of cell@OBC becomes progressively less negative as the coupling strength increases. This shift indicates a gradual and controllable alteration of the cell surface physicochemical environment, consistent with an increased surface coverage by the grafted organic layer. To exclude the possibility that the observed zeta-potential change originates from non-specific adsorption or simple physical association, we carried out an extra control experiment in which native cells were mixed with OBC at different concentrations without EDC/NHS coupling. Under these conditions (**Supplementary Figure 10**), the zeta potential remains essentially unchanged (still around −40 to −45 mV) across the tested OBC concentrations, showing no trend comparable to that observed in **Figure 2e**. This result indicates that simple mixing is insufficient to induce the obvious surface-charge change.

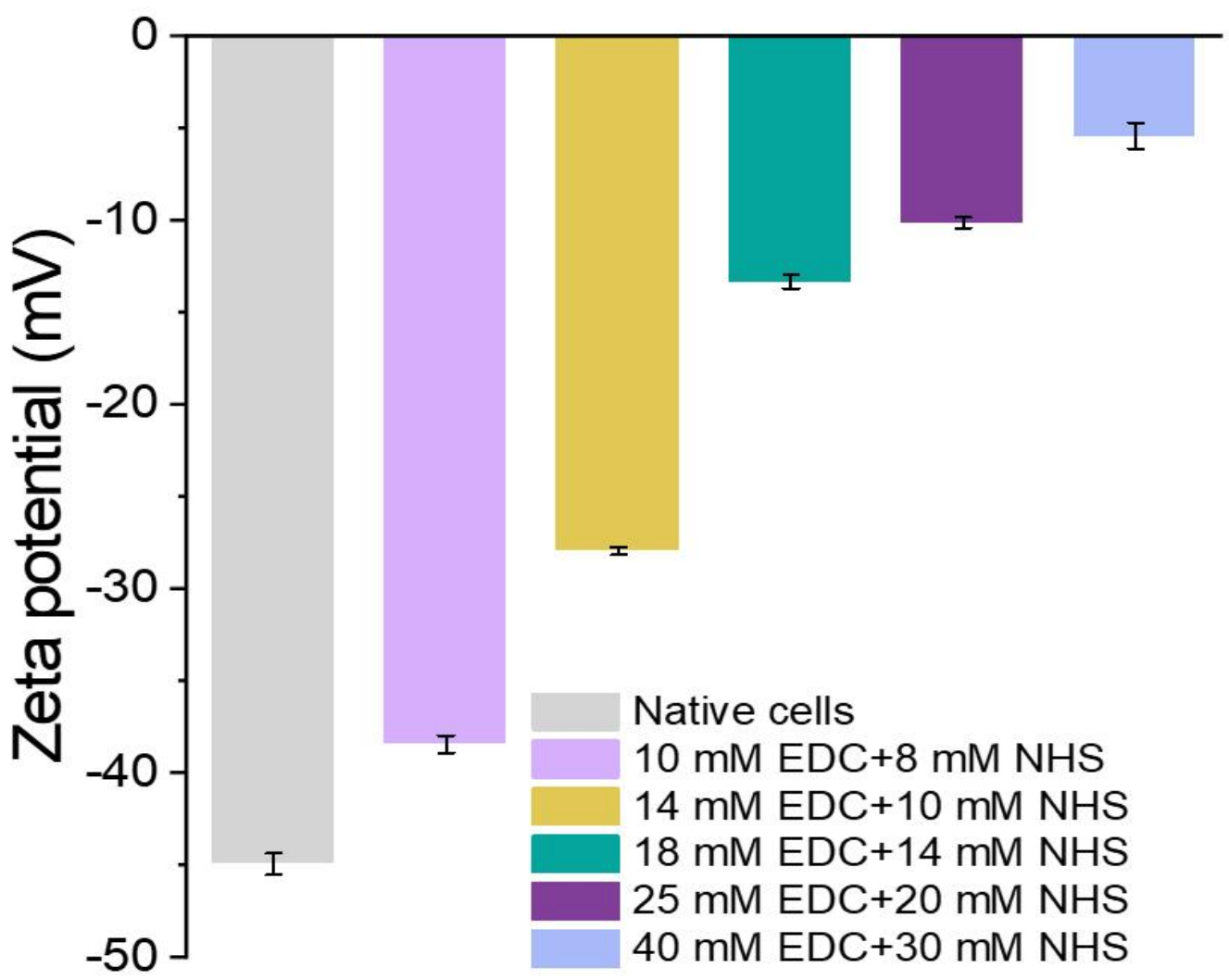

Figure 2e. Zeta potential of native cells and cell@OBC modified with different amounts of coupling agent. Data are presented as mean values +/- SD based on n=3 independent replicates with the error bars representing the SD.

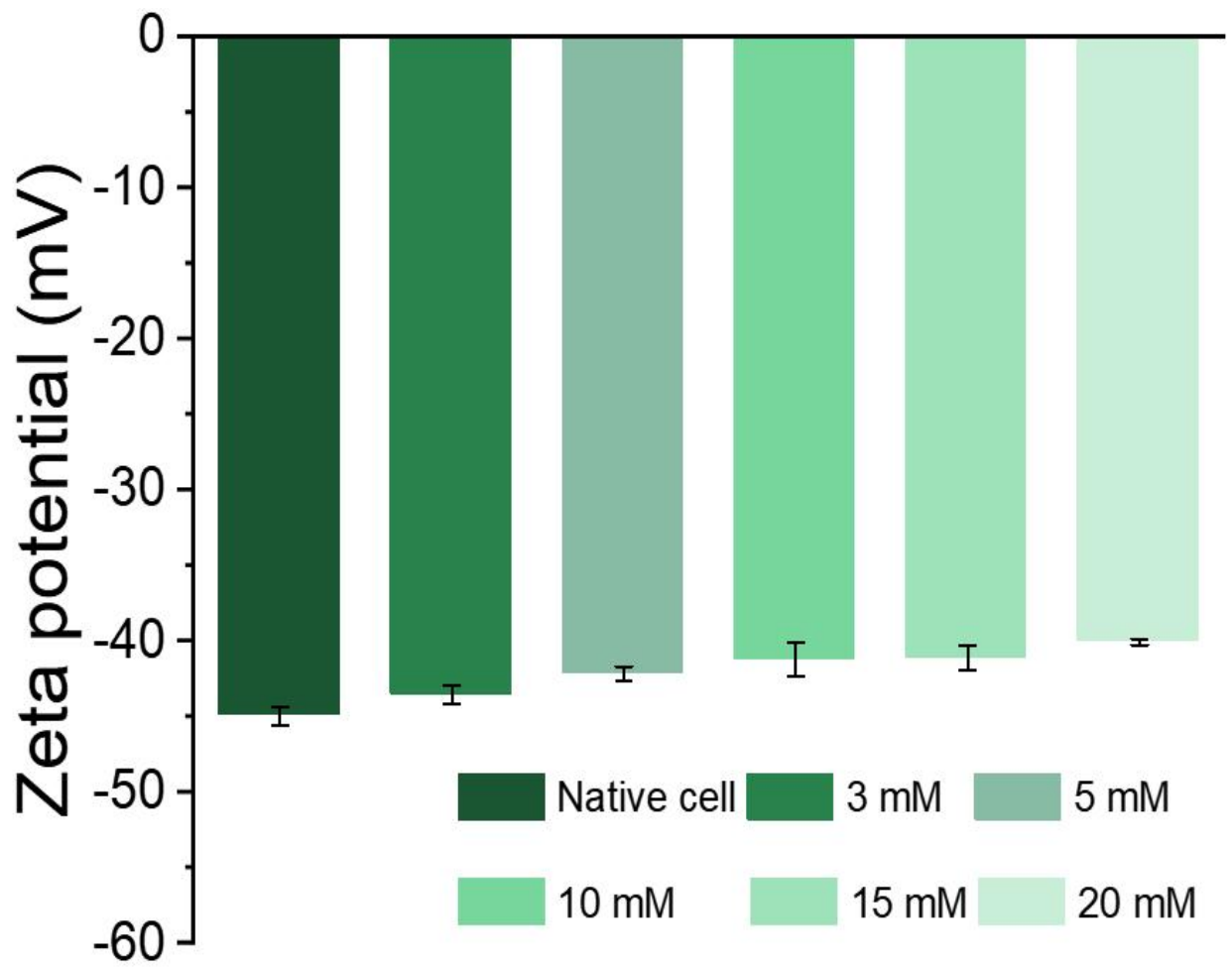

Supplementary Figure 10. Zeta potential of native cells and native cells mixed with OBC solutions at different concentrations. Data are presented as mean values +/- SD based on n=3 independent replicates with the error bars representing the SD.

Comment 4: The interfacial area is lower at water-to-oil ratios of 2:8, 1:9, and 3:7 compared with other ratios (Figure 3o). Please provide a clearer explanation for this observation.

Reply: We thank the reviewer for this helpful request and agree that a clearer mechanistic explanation is needed. The total interfacial area in a water-in-oil Pickering emulsion is jointly determined by the dispersed-phase volume fraction and the droplet size distribution. When the water content is very low (e.g., water-to-oil ratios of 1:9, 2:8, and 3:7), the limited water phase is insufficient to generate a large number of droplets under the same emulsification conditions. In addition, early-stage fusion of droplets is more pronounced when the internal phase is lacking, resulting in relatively larger droplets. The combination of a low dispersed-phase fraction and an increased mean droplet diameter therefore leads to a reduced total interfacial area. In contrast, at intermediate water-to-oil ratios such as 6:4, the system reaches an optimal balance between

droplet generation and interfacial stabilization. The water phase fraction is high enough to form a large number of droplets, while the water-in-oil architecture remains intact and the available cell@OBC stabilizers can adequately cover newly created interfaces. This results in the smallest average droplet size, the highest droplet number density, and consequently the maximum total interfacial area observed in Fig. 4o. We have revised the manuscript to clarify this when discussing the interfacial area data.

Comment 5: The industrial potential of assembly-line biocatalysis is demonstrated through scale-up experiments, but it is not clear whether stirring or shaking is required in all catalytic reactions, including scale-up. Please clarify the agitation conditions used.

Reply: Thank you for this important comment regarding agitation conditions. We would like to clarify that in all catalytic experiments reported in this work, including the 5 L scale-up experiment, no additional stirring or shaking was applied during the reaction. Gentle handshaking was only used initially to form the emulsion and distribute cell@OBC at the oil–water interface. Once formed, the Pickering emulsion remained stable throughout the reaction without external agitation, and the catalytic reactions proceeded under static conditions. This behavior reflects the inherent stability of the particle-stabilized interface and indicates that continuous mechanical energy input is not required.

Comment 6: The protective coating on the cell surface may introduce an additional mass transfer barrier. Compared with unmodified cells, are substrates or products more difficult to cross the cell membrane? A brief discussion would be helpful.

Reply: Thank you for your valuable suggestion. Whole-cell biocatalysis is well established and widely applied for the synthesis of value-added chemicals, where native cell membranes generally do not constitute a prohibitive diffusion barrier for small-molecule substrates. Consistent with this understanding, we observed that native cells catalyze the model reaction efficiently under our experimental conditions. We fully understand your concern that the additional OBC layer on the

cell surface might introduce an extra diffusion barrier. To address this, we conducted a new experiment in which the catalytic performance of native cells, cell@OBC, and free enzymes (BAL) released from lysed cell@OBC were compared under the same conditions. As shown in **Figure 2g** and **Supplementary Figure 11**, all three systems produced comparable amounts of product within 10 minutes, with no statistically significant differences observed. This result indicates that substrate diffusion to intracellular enzymes is not measurably hindered by the OBC coating. Taken together with the efficient cascade reactions shown in the emulsion system, these results demonstrate that the surface modification does not impose a diffusion limitation on biocatalysis, while enabling the additional interfacial functionalities reported in this work. To this end, we have included additional discussion in the manuscript to strengthen the content.

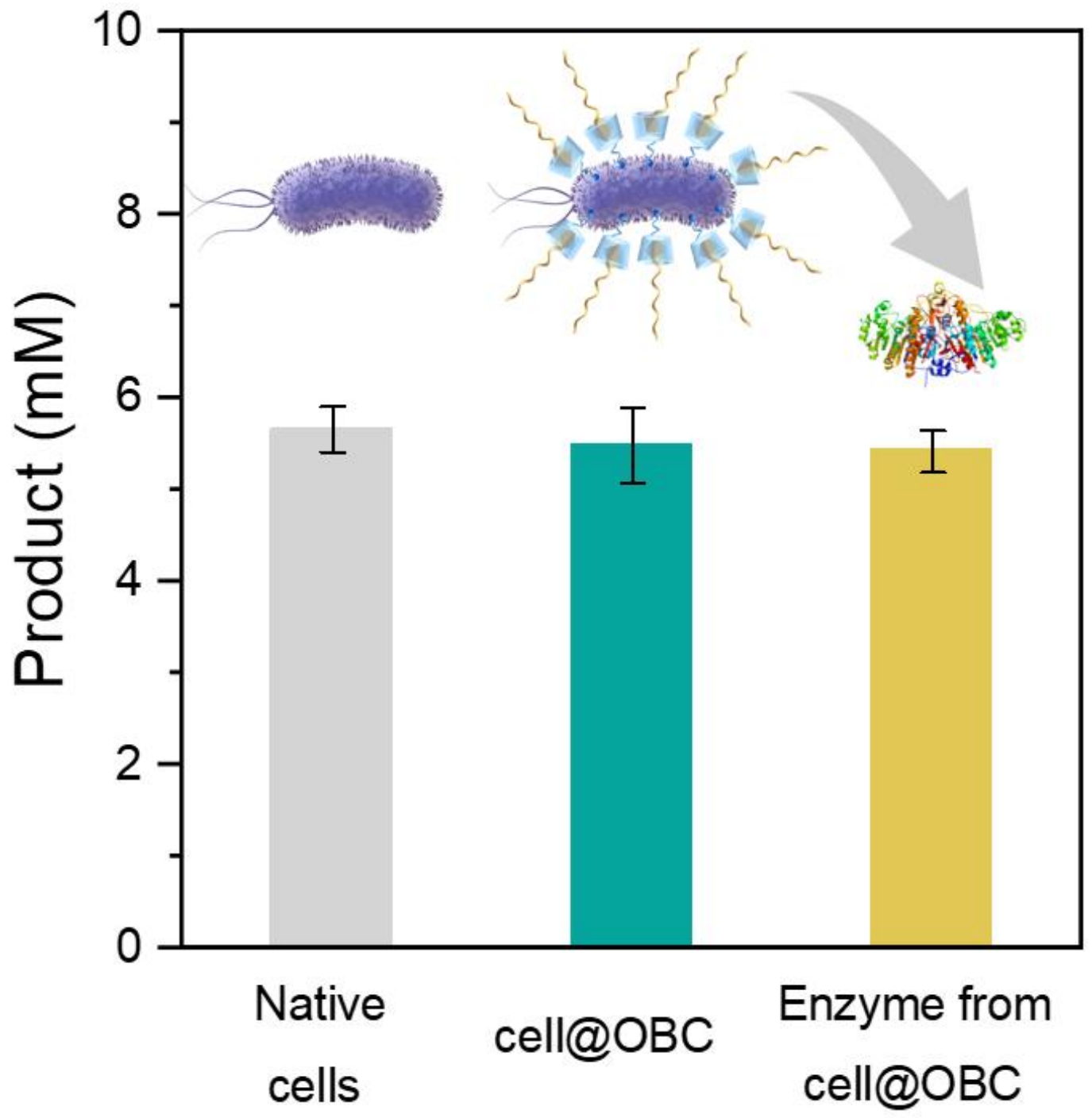

Figure 2g. The catalytic activity of native cells, cell@OBC, and the enzyme lysed from cell@OBC.

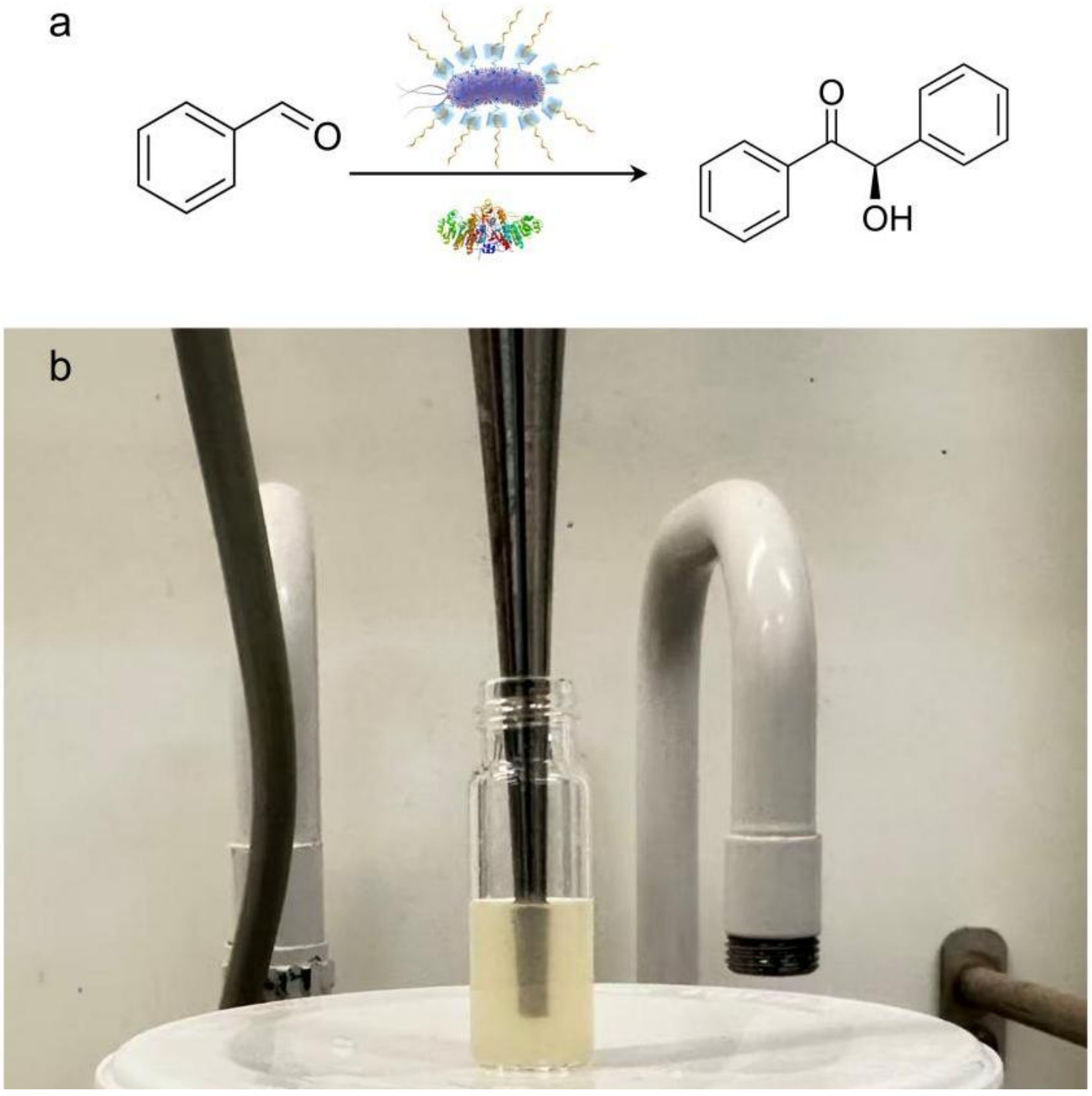

Supplementary Figure 11. (a) The catalytic activity is assessed through the BAL-induced benzoin condensation reaction. (b) Enzyme lysis is achieved by sonication.

Comment 7: The recycling of oil-based photocatalysts is an attractive feature. Please clarify the rationale for choosing 1-adamantanecarboxylic acid as the competitive guest.

Reply: Thank you for the question. We selected 1-adamantanecarboxylic acid as the competitive guest for two complementary reasons that are both mechanistically and practically important for the selective recycling of the oil-based photocatalyst (OBC). First, the adamantane/β-cyclodextrin pair is a well-known high-affinity host–guest interaction, and in our system, this higher binding energy provides a clear thermodynamic driving force to displace the OBC guest from the β-CD host on the cell surface. This design principle is further supported by our molecular dynamics analysis in the manuscript, where adamantane preferentially occupies the β-CD cavity and expels OBC, consistent with the stronger host–guest interaction predicted for adamantane–CD compared to OBC-CD. Second, beyond binding energy, 1-adamantanecarboxylic acid was chosen specifically to simplify downstream purification and enable a truly practical recycling workflow.

Unlike non-functionalized adamantane, 1-adamantanecarboxylic acid can be readily deprotonated and dissolved in alkaline aqueous solutions, which is highly advantageous when separating products and recovering OBC. In our recycling procedure, after competitive displacement in DMSO and solvent removal, the crude product contains recovered OBC along with residual 1-adamantanecarboxylic acid. We then perform simple alkaline water washing to selectively extract 1-adamantanecarboxylic acid into the water phase, leaving behind the hydrophobic OBC without additional chromatographic purification.

Comment 8: The engineered *E. coli* can be reproduced by cell culture and the co-factor of reductase can be regenerated by utilizing co-substrate, which indicates that the biocatalytic reaction is catalyzed by living cells. Thus, the photocatalytic chemical reaction integrating with biocatalytic reaction catalyzed by living cells have achieved in the present work. However, the replicated cells should have no the modified characteristics (Fig. S10), such as hydrophobic surfaces. Those cells will lose the ability to act as Pickering emulsifiers. Does it influence the recycling of cells as biocatalyst?

Reply: Thank you for your valuable suggestion. We found that when the recycled cells were re-cultured and allowed to proliferate, the newly generated cells were no longer able to reform a Pickering emulsion upon handshaking (**Supplementary Figure 17a**). This behavior is expected because the Pickering emulsification capability in our system is due to the engineered surface amphiphilicity introduced by the OBC-related assembly rather than from genetically encoded cell properties. During cell division, this non-covalently assembled amphiphilic layer is diluted or shed, causing the replicated cells to revert toward a more hydrophilic, native-like surface state and thereby lose the ability to function as Pickering emulsifiers. Importantly, the loss of emulsification ability in re-cultured cells does not hinder cell recovery or the overall recycling strategy. Even when a stable emulsion cannot be re-formed, the cells in the mixed system can still be efficiently recovered by centrifugation, as shown in **Supplementary Figure 17b**. This distinction highlights that the Pickering function is associated with the engineered surface state of the original

cell@OBC particles rather than with cell proliferation itself. In practical operation, this behavior is fully compatible with our proposed dual-mode recycling strategy, where engineered cells can be directly reused for multiple cycles, while re-cultured cells can be re-functionalized if needed to restore surface amphiphilicity and interfacial activity.

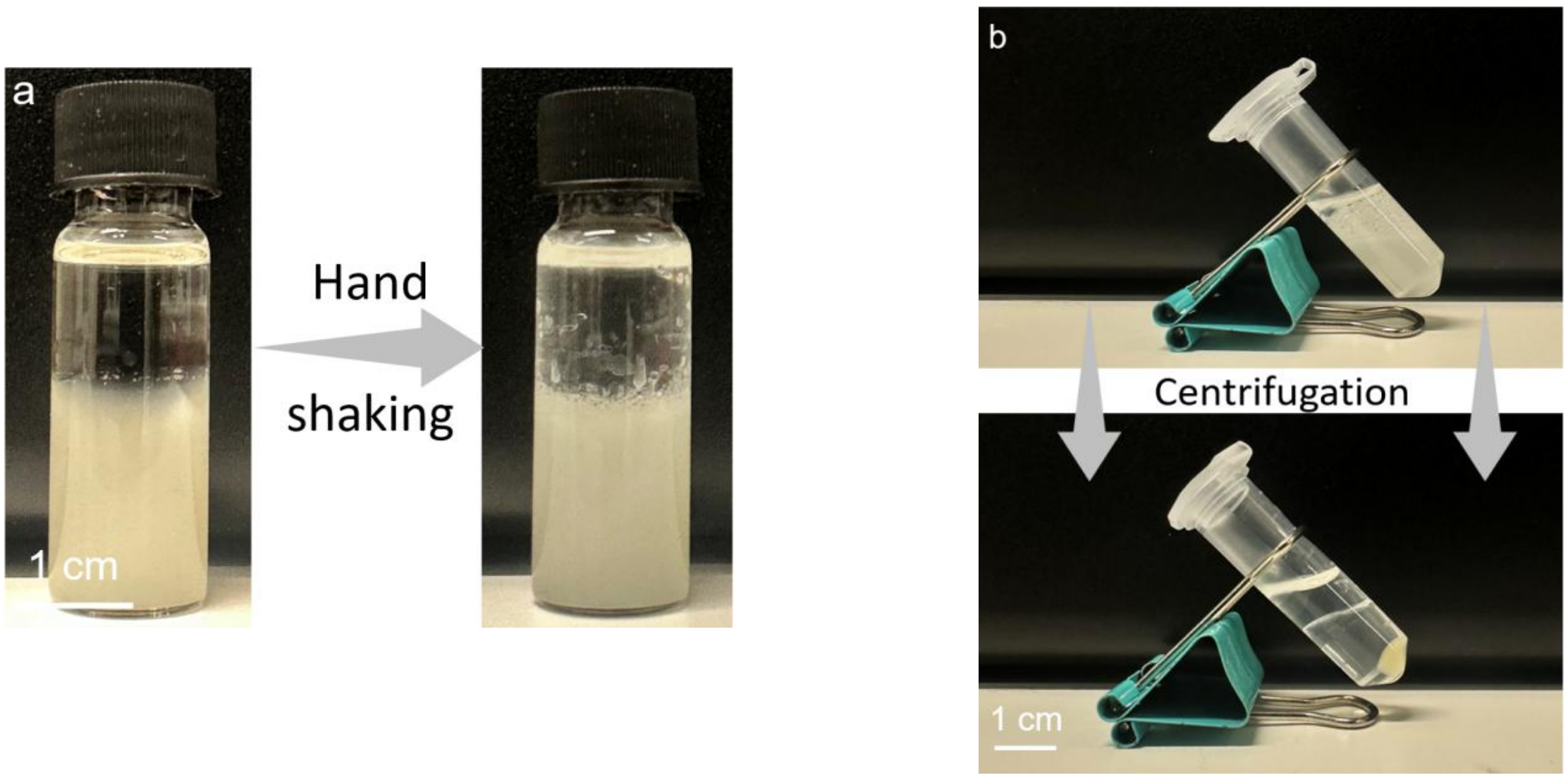

Supplementary Figure 17. (a) The replicated cells lose the ability to emulsify as the hydrophobic properties on the surface are lost. (b) The replicated cells can still be recycled by centrifugation, despite the loss of the functionalized interface.

Comment 9: Chemo-enzymatic cascades, which merge chemical and biological catalysis, are emerging as powerful tools for sustainable chemical production. The biocompatible chemistry has even applied to regulate microbial metabolism (Wallace, *et al*. Using non-enzymatic chemistry to influence microbial metabolism. Curr Opin Chem Biol 2015, 25: 71-79; Xue, *et al*. Driving non-enzymatic reactions towards biocompatible chemistry. Trends in chemistry 2025, 7(2): 66-69). The photo-catalysis of chemical reaction should be immobilized catalyst on the cell surfaces in the present work (Fig.S6) as well as the previous works (Zhou, *et al*. Integrating micelle catalysts with living cells for recyclable photoenzymatic cascades. Chem catalysis 2025, doi.org/10.1016/j.checat.2025.101517). Why did not add the photo-catalyst directly into the oil-

water two-phase system (in other words, providing the benefits of integrating of chemical catalyst with biocatalyst) ?

Reply: Thank you for your question. We would like to clarify that directly adding the oil-based photocatalyst (OBC) into an oil–water biphasic system is fundamentally different from the design concept and practical objective of our platform. OBC is hydrophobic and readily dissolves in the organic phase (toluene in this work). As a result, when OBC is freely dispersed in a biphasic system, it coexists with the product and unreacted substrate in the same organic phase after the reaction. Under such conditions, selective recycling of the photocatalyst becomes impractical, as separation from chemically similar organic components would require additional chromatographic or solvent-intensive purification steps, which undermines the sustainability and recyclability goals of the system. More importantly, in our design, OBC is not introduced merely as a freely diffusing photocatalyst, but as a multifunctional interfacial component. By anchoring OBC onto the cell surface via host–guest interactions, it simultaneously serves two critical roles: (i) providing localized photochemical activity at the oil–water interface, and (ii) modulating the surface amphiphilicity of *E. coli* to enable the formation of stable Pickering emulsions. This dual function cannot be achieved by simply dissolving OBC in the oil phase. The surface-engineered cell@OBC particles act as both the catalytic scaffold and the Pickering stabilizer, creating an enlarged and persistent reactive interface while allowing the photocatalyst to be selectively displaced and recycled after reaction. We have clarified this conceptual distinction in the revised manuscript to emphasize that interfacial immobilization of OBC is essential not only for cascade efficiency, but also for emulsion construction and catalyst recyclability.

Other minor remarks:

Remark 1**:** In terms of system design, the reason for choosing plant oil is not clearly stated. Please briefly justify this choice.

Reply: Thank you for your valuable suggestion. We have included relevant descriptions in the revised manuscript to explain the reasons for selecting plant oils.

Remark 2**:** "Superbacteria" are shown in Figure 1 and the manuscript, additional explanation in the text would improve clarity.

Reply: Thank you for your valuable suggestion. The concept of "Superbacteria" has been added to the manuscript.

Remark 3**:** In the plate assay in Figure 2s (right panel), zones 2 and 3 are not clearly separated. Please repeat the experiment or provide a clearer image.

Reply: Thank you for your valuable suggestion. Additional experiments were conducted to replace the previous version.

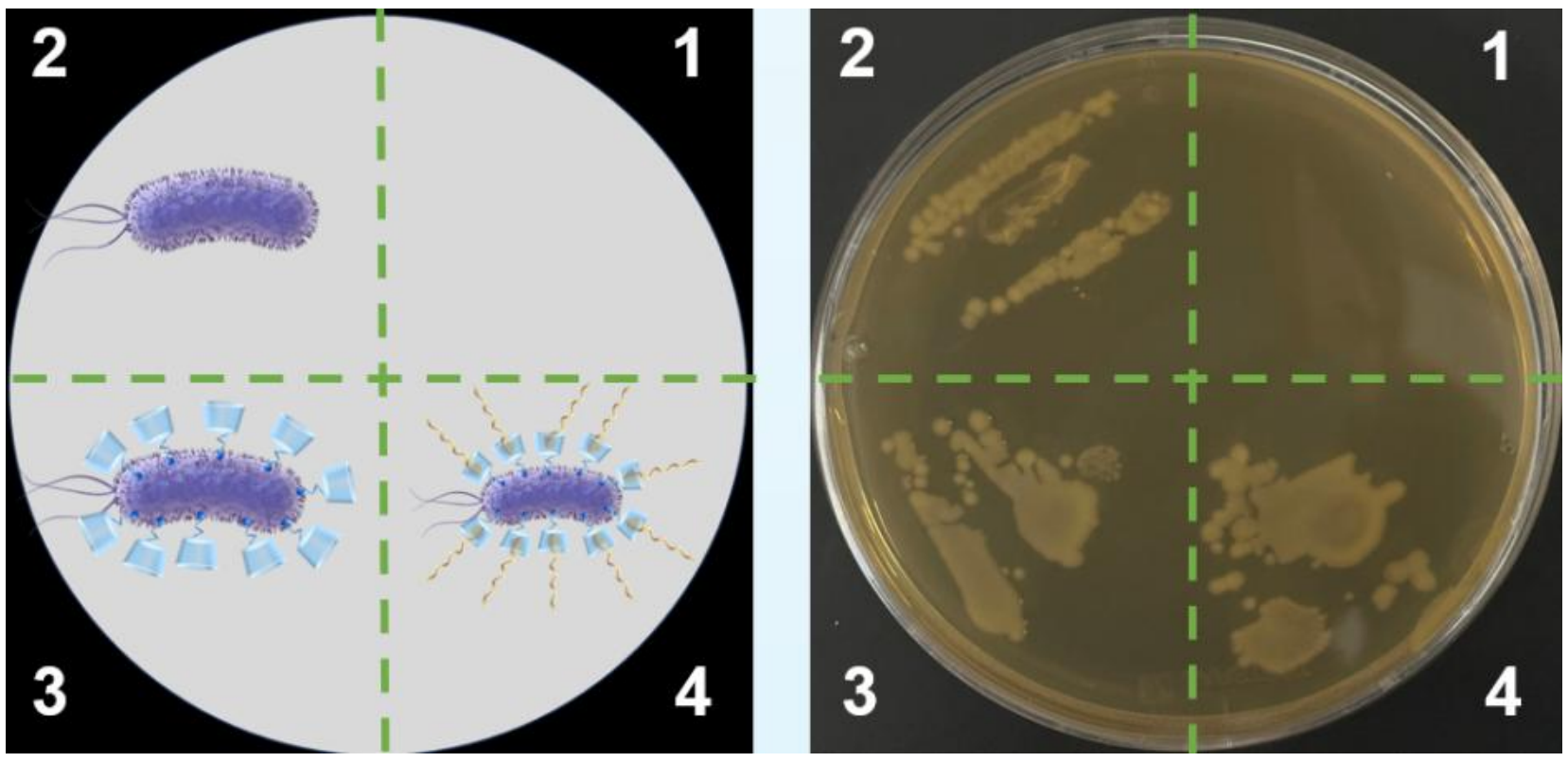

Figure 3q. Schematic representation of the plating assay and the image of the plating assay, showing native cells (section 2), cell@CD (section 3) and cell@OBC (section 4), section 1 is blank control.

Remark 4**:** Details of the photoenzyme-catalyzed reactions in Figures 4 and 5 (reaction conditions, etc.) should be provided, preferably in the Supporting Information.

Reply: Thanks for the comment. All details regarding the photochemical catalysis mentioned in the manuscript have been supplemented in the supporting information.

Remark 5: The term cell@OBC is used frequently, but it is difficult for readers to recognize it in the figures. Please label cell@OBC explicitly to improve readability.

Reply: Thank you for your valuable suggestion. Information about cell@OBC has been added to some figures in the manuscript to enhance readability.

Reviewer #3:

The work by Wu et al. presents a nice work of biocatalysis using bacteria-stabilized Pickering emulsions. The work is interesting and the science is correct. Unfortunately, the topic and the work itself does not contain the novelty required for a publication in Nature Communications. Of course the paper is publishable but not in this journal. I recommend a submission to a more specialized journal, e.g., Communication Chemistry. Some additional comments.

Reply: We thank you for the thorough evaluation of our manuscript and the encouraging remarks regarding the interest and correctness of the work. We have used this feedback to substantially improve the manuscript. In particular, we have conducted numerous new experiments and added 43 new figures (in both the main text and Supplementary Information) to address each comment in detail. We believe these revisions highlight the **novelty, broad impact, and suitability** of our study for *Nature Communications*.

Regarding your comments on overall novelty and suitability for Nature Communications, we respectfully emphasize that our work indeed contains significant novel contributions and broad impact that make it well-suited for a general-interest and high-standard journal of *Nature Communications*. We have explicitly clarified the unique aspects of our platform and why it is different from prior studies. Key points highlighting the **novelty and importance** of this work are outlined below:

- **Dual Catalyst Recycling (Novel Functionality):** To our knowledge, this is the first report of an on-demand, component-selective dual recycling of catalysts in a chemoenzymatic cascade system. We demonstrate that both the whole-cell biocatalyst and the synthetic photocatalyst can be recovered and reused in a tunable fashion. This goes beyond conventional Pickering emulsions (which are often "reusable" in a generic sense) by introducing catalyst recovery as a designable element. It directly addresses a long-standing challenge in biocatalysis – the difficulty of recycling multiple catalysts with different stabilities in one pot. Our system allows one to replace or refresh each catalyst independently as needed, **saving cost and simplifying processes** that involve multi-step catalysis.

- **Broad Scope and Scalability:** The platform's versatility and robustness are evidenced by a broad reaction scope and the ability to scale the system from milliliters to liters. In the revised manuscript, we showcase diverse applications ranging from single-step biotransformations to multi-step chemoenzymatic cascade reactions, all accelerated by the Pickering emulsion interface. We also demonstrate a 5000-fold scale-up (from 1 mL to 5 L) culminating in gram-scale production of an enantioenriched product (R-benzoin) with retained efficiency and selectivity (see our response to Comment 7 and Figs. 6i, j in the revised paper). This scalability and generality underscore the potential of our approach for industrial biocatalysis, going well beyond a single specialized reaction and mL-scale experiment.

- **Sustainability and Green Chemistry Alignment:** Our strategy is firmly rooted in green chemistry principles. The dynamic catalyst recycling mentioned above maximizes catalyst utilization and minimizes waste. Moreover, the photocatalyst (OBC) is synthesized from a low-value, non-edible plant oil feedstock (a renewable resource), rather than from petrochemical precursors, thereby connecting catalytic function with renewable resource utilization. We have expanded the manuscript's discussion (see response to Comment 3) to explicitly highlight these sustainable aspects, including new experiments demonstrating energy-efficient

emulsification and the use of greener solvents without performance loss (Supplementary Figs. 54 and 55). Collectively, these features position our work at the nexus of biocatalysis, interfacial engineering, and sustainable chemistry, which we believe is well aligned with the scope of Nature Communications.

In light of the above points, the extensive new data and clarifications provided, and our great efforts made, we hope that you can recognize that the revised manuscript meets the novelty and significance criteria for Nature Communications. We are grateful for your suggestion and have carefully improved our work to emphasize its unique contributions.

Comment 1: Recent reviews should be included in the reference list: https://doi.org/10.1002/cctc.202400856.

Reply: We appreciate this suggestion. We have added the recommended recent review (Guzmán et al., **ChemCatChem** 2024) to our reference list in the revised manuscript. In addition, we have included several other up-to-date review articles relevant to Pickering interfacial catalysis and biocatalysis to strengthen the context of our work. These new references include:

1) ChemCatChem 16, e202400856, (2024) (doi.org/10.1002/cctc.202400856);

2) Adv. Colloid. Interfac. 302, 102620, (2022). (doi.org/10.1016/j.cis.2022.102620);

3) J. Mol. Liq 425, 127259, (2025). (doi.org/10.1016/j.molliq.2025.127259);

4) Catal. Today. 462, 115534, (2026). (doi.org/10.1016/j.cattod.2025.115534);

5) Curr. Opin. Colloid. In. 71, 101804, (2024). (doi.org/10.1016/j.cocis.2024.101804).

By incorporating these references, we have ensured that the introduction and discussion properly acknowledge the current state of the field.

Comment 2: The claim that typically Pickering emulsions rely on inert particles like silica or polymers is very strong. There are many examples of Pickering stabilization.

Reply: We agree with you for this point and have revised the wording in the introduction to avoid any overgeneralization. The revised text in Introduction is now expressed as "In many established systems, Pickering emulsions are stabilized by solid particles such as inorganic, polymeric, and bio-derived, which can be strongly anchored at the interface. This not only maintains a large interfacial surface area but also effectively prevents coalescence and promotes interfacial mass transfer".

Comment 3: Some mention to the alignment of the work to the Green Chemistry Principles would be good.

Reply: We highly appreciate your suggestion and have significantly expanded our discussion to explicitly illustrate how our work aligns with multiple principles of green chemistry. In response to this comment, we performed new experiments and added a schematic summary to underscore the sustainability advantages of our system. These additions are described below, corresponding to several core green chemistry principles:

- **Design for Energy Efficiency**: We evaluated low-energy emulsification methods. Remarkably, simply hand-shaking the mixture is sufficient to form a stable Pickering emulsion that supports efficient catalysis. In fact, we compared three methods – hand shaking, vortex mixing, and sonication – and observed that all produced equally stable emulsions (no phase separation for 24 h) and yielded nearly identical product conversions (**Supplementary Figure. 55**). This result indicates that high mechanical energy input is not required; the mildest method (manual shaking) works just as well, highlighting the energy-efficient nature of our system.

- **Safer Solvents:** To minimize the use of hazardous organic solvents, we tested greener solvent alternatives in place of toluene for the organic phase. In the revised **Supplementary Figure 56**, we show that solvents such as ethyl acetate, heptane, and octane can all form stable Pickering emulsions with our cells, and the catalytic performance in these emulsions remains comparable to that in toluene. This demonstrates that our interfacial biocatalysis

system is compatible with eco-friendly solvents, allowing users to choose solvents based on sustainability without sacrificing efficacy.

- **Use of Renewable Feedstocks:** We introduced a timeline schematic for an on-demand, dual-mode recycling strategy (**Supplementary Figure 62**), which highlights the efficiency and selectivity of catalyst reuse. Whole-cell particles can be rapidly recovered within approximately 10 min for short-cycle reuse, while the photocatalyst can alternatively be selectively separated and regenerated for longer-term operation. This modular recycling design minimizes waste generation and maximizes catalyst use, further strengthening the sustainability profile of the system.
- **Designing Safer Chemicals:** In addition, it is worth noting that the photocatalyst (OBC) employed in this work is derived from non-edible and non-toxic plant oil, a resource commonly regarded as agricultural or forestry waste. The use of such renewable and low-value feedstocks contributes to a reduced environmental footprint and aligns with the principle of using renewable raw materials.
- **Atom Economy and Catalysis:** Beyond sustainability at the process level, we would also like to emphasize the advantages of this system in specific reactions. For example: the enantioselective conversion of benzaldehyde to benzoin. Conventional benzoin condensation reactions, typically catalyzed by N-heterocyclic carbenes (NHCs), can achieve asymmetry only through the introduction of specially designed chiral catalysts/ligands (e.g., chiral triazolium/NHC precatalysts or other tailored chiral organocatalysts), whose preparation is often multistep, time-consuming, and resource-intensive (Enders. D. *et. al.*, Angew. Chem. Int. Ed. 2002, 41, 1743-1745; Huang. X. L. *et al.*, Chinese Sci Bull, 2010, 55, 1753 – 1757). Moreover, high enantioselectivity commonly requires stringent reaction conditions (e.g., anhydrous environments, low temperatures, inert atmospheres) and is frequently accompanied by laborious purification to remove chiral catalysts and side products, increasing

solvent usage, energy input, time, and overall economic cost. In contrast, the living cell Pickering interfacial system reported here enables the highly selective synthesis of chiral benzoin derivatives directly from benzaldehyde under mild conditions, without the need for externally synthesized chiral ligands or complex catalyst architectures, thereby integrating stereochemical control with green chemistry principles in a fundamentally different and more sustainable manner.

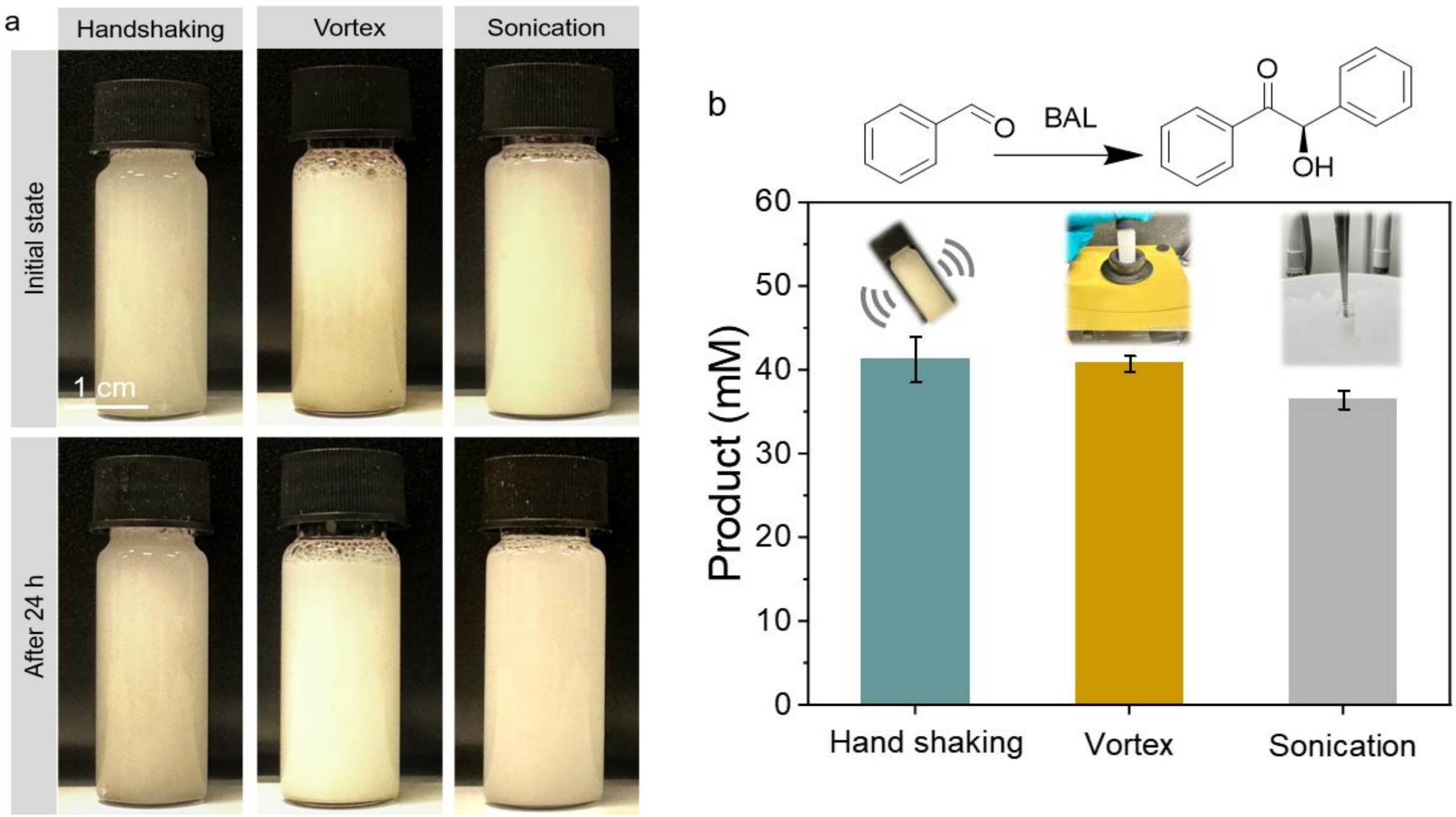

Supplementary Figure 55. (a) Whether using hand shaking, vortexing, or sonication treatment, robust Pickering emulsion systems can be obtained that remain stable for 24 hours. (b) Emulsions prepared by hand shaking, vortexing, and sonication exhibit consistent catalytic activity.

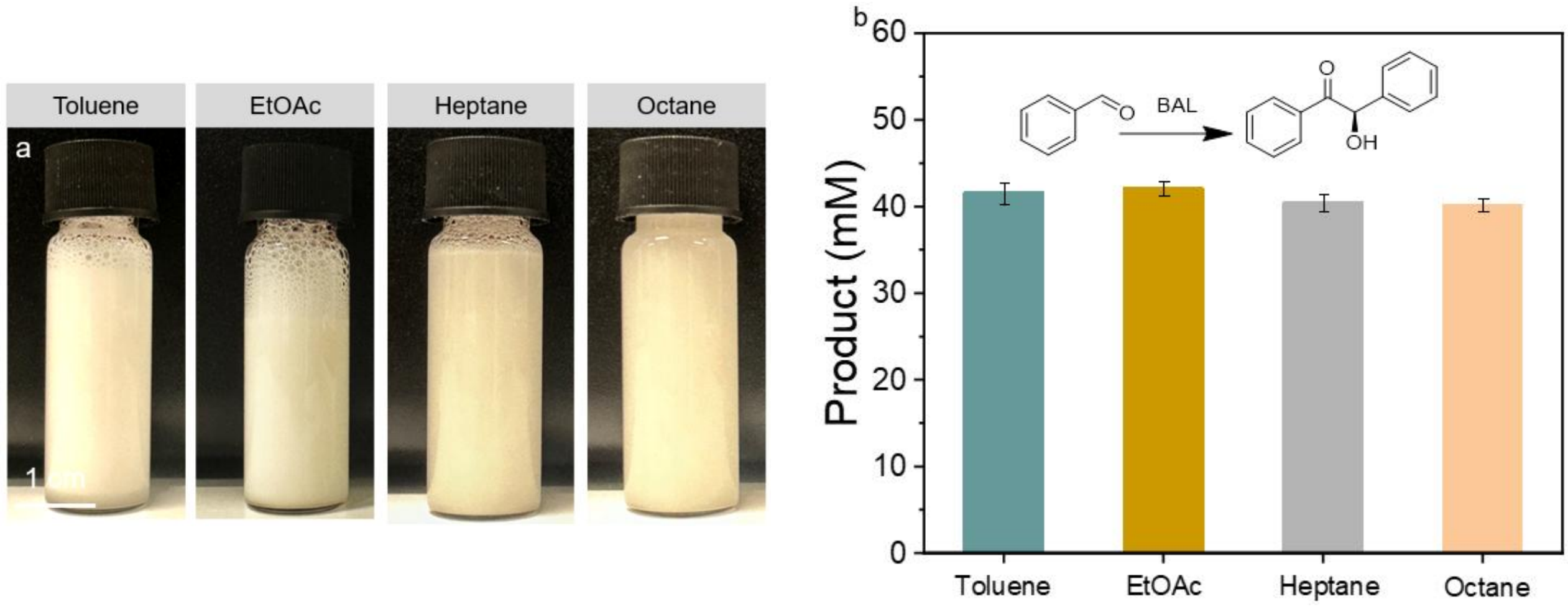

Supplementary Figure 56. (a) Three more eco-friendly solvents (ethyl acetate, hexane and octane) serve as reliable alternatives to toluene, producing emulsions with consistent stability. (b) Pickering emulsions prepared with four different organic phases showed no difference in catalytic activity.

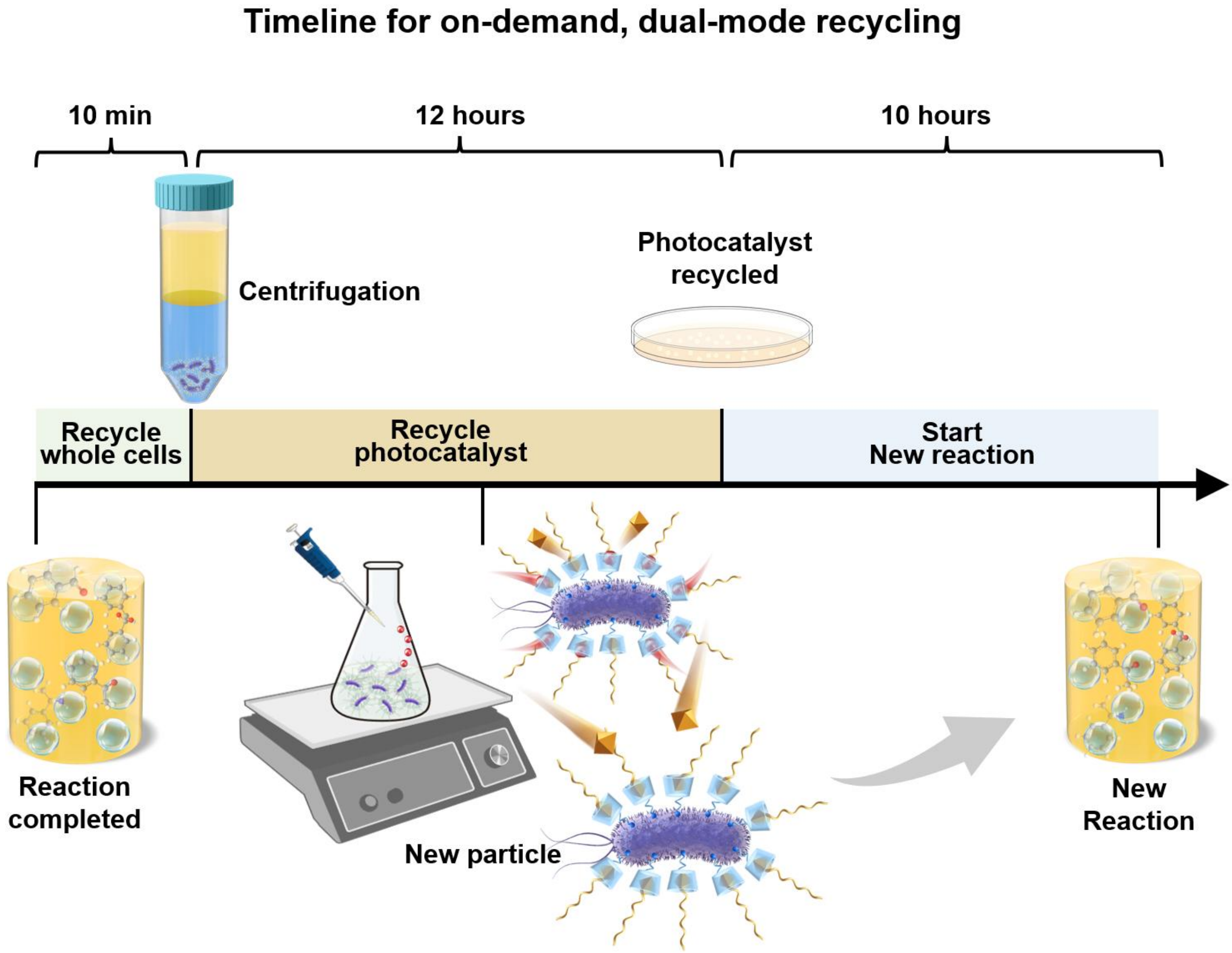

Supplementary Figure 62. Operational workflows and timelines for dual-mode, on-demand recycling.

By adding the above results and discussion (see the "sustainable catalysis via on-demand dual-mode recycling" section of the revised manuscript and **Supplementary Figures. 55, 56 and 62**), we have made it clear that our work is firmly aligned with green chemistry ideals. We thank you for prompting us to strengthen this important aspect of the manuscript.

Comment 4: Using hand shaking as strategy for transporting the stabilizing agent to the interface opens an important question related to the irreversibility of the attachment of the species, and this opens the question about the true Pickering stabilization.

Reply: Thanks for this comment. We recognize your concern and have addressed it through additional discussion and new experimental evidence. The question essentially has two parts: (i) Does gentle hand-shaking truly produce a Pickering-stabilized emulsion (with particles irreversibly anchored at the interface), or is the stability questionable? (ii) How do we reconcile the reversible nature of our supramolecular cell–catalyst attachment with the notion of a stable Pickering interface (which is typically considered irreversible on experimental timescales)? We have clarified both points as follows:

1. Low-Energy Emulsification and Pickering Theory: Forming a stable Pickering emulsion by hand shaking is feasible in our system because of the optimized particle properties (appropriate size, wettability, etc.). We note that Pickering emulsion stability is governed by well-established factors – as described by Pieranski's classic equation (see the below equation 1. Pawel. *Phys. Rev. Lett.* 1980, *45*, 569), the attachment energy of particles at an interface can be very high, so even gentle mixing can suffice if particles have the right wettability and size.

$$\Delta E = -\frac{\pi r^2}{\gamma_{OW}}[\gamma_{OW} - (\gamma_{PW} - \gamma_{PO})]^2 \qquad \text{(Eq. 1)}$$

Where γ_{OW}, γ_{PW} and γ_{PO} represent interfacial energy from interface of oil/water, particle/water, and particle/oil, respectively, and r is particle radius.

In other words, once our cell@OBC conjugates reach the interface, they spontaneously anchor and lower the interfacial energy, leading to stable droplets. Hand shaking is merely a convenient, low-shear way to disperse the phases; it does not imply that the particles are weakly attached. In fact, literature provides many examples of Pickering emulsions (including those stabilized by bacteria) being prepared with simple handle-shaking rather than high-shear homogenization. For instance: Jiang, H. et al., *Chem. Sci.*, 2022, *13*, 39-43; Liu, L. et al., *J. Coll. Inter. Sci.*, 2023, *629*, 559–568; Wu, X et al., *Chem. Sci.*, 2025, *16*, 4892-4899.

We have added references to such studies in the revised manuscript to reassure that our method is in line with established practice.

2. New Experiments – Microscopic Evidence of Interfacial Anchoring: To directly demonstrate true Pickering stabilization in our hand-shaken emulsions, we are very happy to perform new fluorescence microscopy experiments with green fluorescent protein (GFP)-labeled cells. We engineered *E. coli* to express GFP and prepared GFP-tagged cell@OBC particles. These fluorescent cells were used to stabilize emulsions, which we then observed under a confocal laser scanning microscope (CLSM). As shown in **Supplementary Figure. 57**, the GFP-labeled cells form a bright green ring at the water–oil interface of each droplet, rather than dispersing in the bulk phases. A 2D cross-sectional image (**Supplementary Figures. 57b**) and a 3D reconstruction (**Supplementary Figure. 57c**) both clearly show the cells localized at the droplet interface, confirming that the bacteria indeed reside at and stabilize the interface. We also plotted the fluorescence intensity across a droplet diameter (**Supplementary Figure. 57d**): the intensity profile exhibits sharp peaks at the interface and negligible signal in the interior, which is the hallmark of particles adsorbed to the interface (as opposed to a soluble surfactant which would distribute throughout). These data provide visual, direct evidence that our emulsions are stabilized by a particulate interfacial layer, fulfilling the criteria of Pickering emulsions.

3. Interfacial Stability under Cycling: We further added new experiment and tested the robustness of this interfacial anchoring over multiple demulsification/emulsification cycles. In the experiment, we deliberately broke the emulsion by centrifugation (which forces droplets to coalesce and separates the phases), then re-shook it to re-form an emulsion – and repeated this for five cycles. The fluorescent images after five cycles (**Supplementary Figures. 57b, c**) showed the same ring-like distribution of GFP–cells at the interface, and the droplet size after five cycles remained comparable to that of a freshly made emulsion (**Supplementary Figure. 57d** and caption). In other words, even after repeatedly disrupting the emulsion and reforming it, the cells consistently re-adsorb at the interface and stabilize the droplets. This confirms that the attachment of cell@OBC to the interface is not a one-time fluke of the initial shaking, but a repeatable and inherent behavior – i.e., the emulsions truly rely on Pickering stabilization by the particles.

4. Clarifying "Reversibility" vs. Interfacial Stability: We have revised the manuscript to clarify the different contexts of "reversibility" in our system, to avoid any confusion. The supramolecular host–guest interaction between β-CD on the cell and the OBC catalyst is intentionally reversible (dynamic) so that the catalyst can be detached when we choose (for recycling purposes). However, this does not mean that the particles spontaneously detach from the interface during normal operation. In fact, once the amphiphilic cell@OBC particles are formed, their adsorption at the water–oil interface is effectively irreversible on the timescale of the reaction. The emulsion can be broken only by applying an external force (e.g., centrifugation or dilution), not by the particles simply falling off the interface. We demonstrate this in **Supplementary Figure. 57a**: after centrifugal demulsification, the recovered cell@OBC particles can be redispersed in water, showing that the cell–OBC linkage permits them to be handled and recycled. But during catalysis (without centrifugation), they remain at the interface, ensuring a stable emulsion. Thus, the "reversible" attachment refers to the chemical modifiability of the cell–catalyst conjugate, whereas the Pickering stabilization is maintained as long as the emulsion is in use. We have explicitly noted this distinction in the revised text. In summary, dynamic supramolecular grafting gives us recyclability, while Pickering anchoring provides operational stability – these two aspects operate at different levels and are not in conflict.

By adding the above explanations and the new Supporting Information data (**Supplementary Figure. 57** and associated description), we believe we have fully addressed your concern. The revised manuscript now provides a clear rationale and experimental proof that hand-shaking is sufficient to achieve a genuine Pickering emulsion in our system, and it explains how our reversible surface chemistry is compatible with stable interfacial adsorption. We thank you for prompting us to fortify this important point.

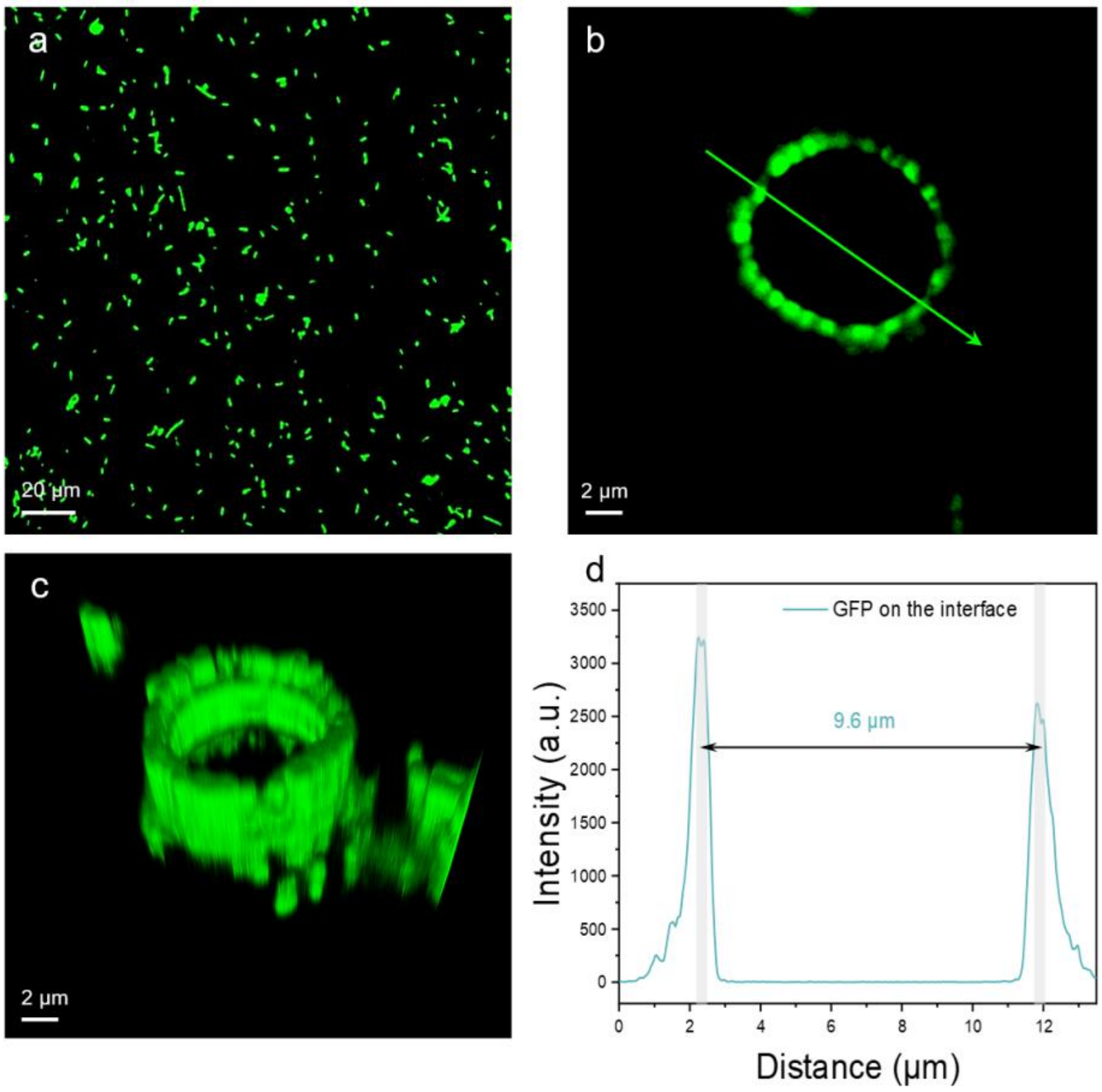

Supplementary Figure 56. (a) Cell@OBC from the emulsion collected by centrifugation were uniformly dispersed in the water phase, confirming the reversible attachment of cell@OBC achieved through handshaking and centrifugation. (b) 2D fluorescence image of GFP-overexpressing cell@OBC stable emulsion after five "centrifugation-emulsification" cycles. (c) The 3D cross-sectional fluorescence image of the stabilized emulsion formed by GFP-overexpressing cell@OBC after five "centrifugation-emulsification" cycles further confirms that cell@OBC are anchored at the water-oil interface. (d) After five "centrifugation-emulsification" cycles, the droplet size closely matches the freshly prepared emulsion.

Comment 5: Figure 3h is counterintuitive, CI is defined in the opposite direction expected for creaming. This mean than CI is high when the stabilizing column is big, and the opposite is true for low CI index. I recommend a careful revision.

Reply: Thank you for your valuable suggestion. We have corrected Fig. 3h by repeated the creaming index (CI) test. The updated results are shown in the revised manuscript (**Fig. 4h**). The

new CI clearly indicates that the CI remains high during the initial period and gradually decreases after prolonged standing, with a pronounced change observed at 48 h, where the emulsion reaches a stable state accompanied by visible phase separation, as confirmed by the newly added photos.

Beyond reporting a single "creaming index," it is important to directly visualize how the droplet evolves to assess whether stability loss originates from coalescence/Ostwald ripening or other processes. Therefore, we performed a CLSM characterization of the cell@OBC-stabilized Pickering emulsion at 0, 6, 12, 24, and 48 h, and the results are shown in the **Supplementary Figure 22**. The emulsion exhibits a uniformly dispersed droplet population with moderate and relatively consistent droplet sizes from 0 to 24 h, with no obvious signs of macroscopic phase separation or localized instability. This directly supports that the cell@OBC particles provide robust interfacial stabilization in our catalysis experiments. In contrast, after 48 h of static standing, we observe clear evidence of droplet growth and merging. The droplet size distribution shifts toward larger droplets and coalescence becomes visible. Therefore, the loss of stability at extended times arises from droplet coalescence rather than an immediate failure of particle adsorption.

Taken together, the added CLSM data and the creaming index provide mutually reinforcing evidence that the cell@OBC Pickering emulsion is stable up to at least 24 h. Droplet coarsening becomes noticeable only upon much longer standing. We now explicitly state as a practical stability boundary in the revised Supporting Information and manuscript.

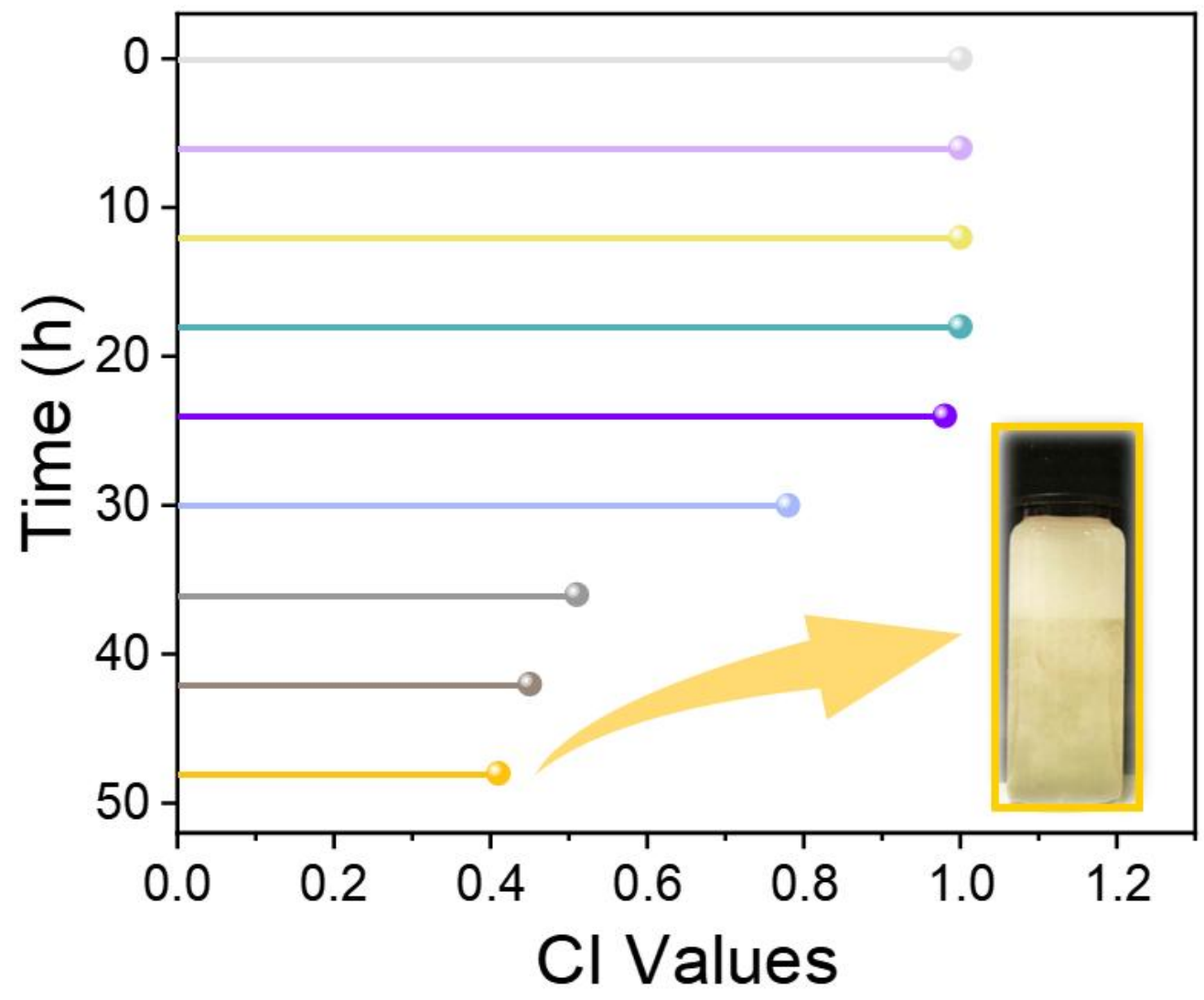

Fig. 4h CI values of emulsion with different storage times; After 48 hours of storage, the emulsion tends to the final state (inset).

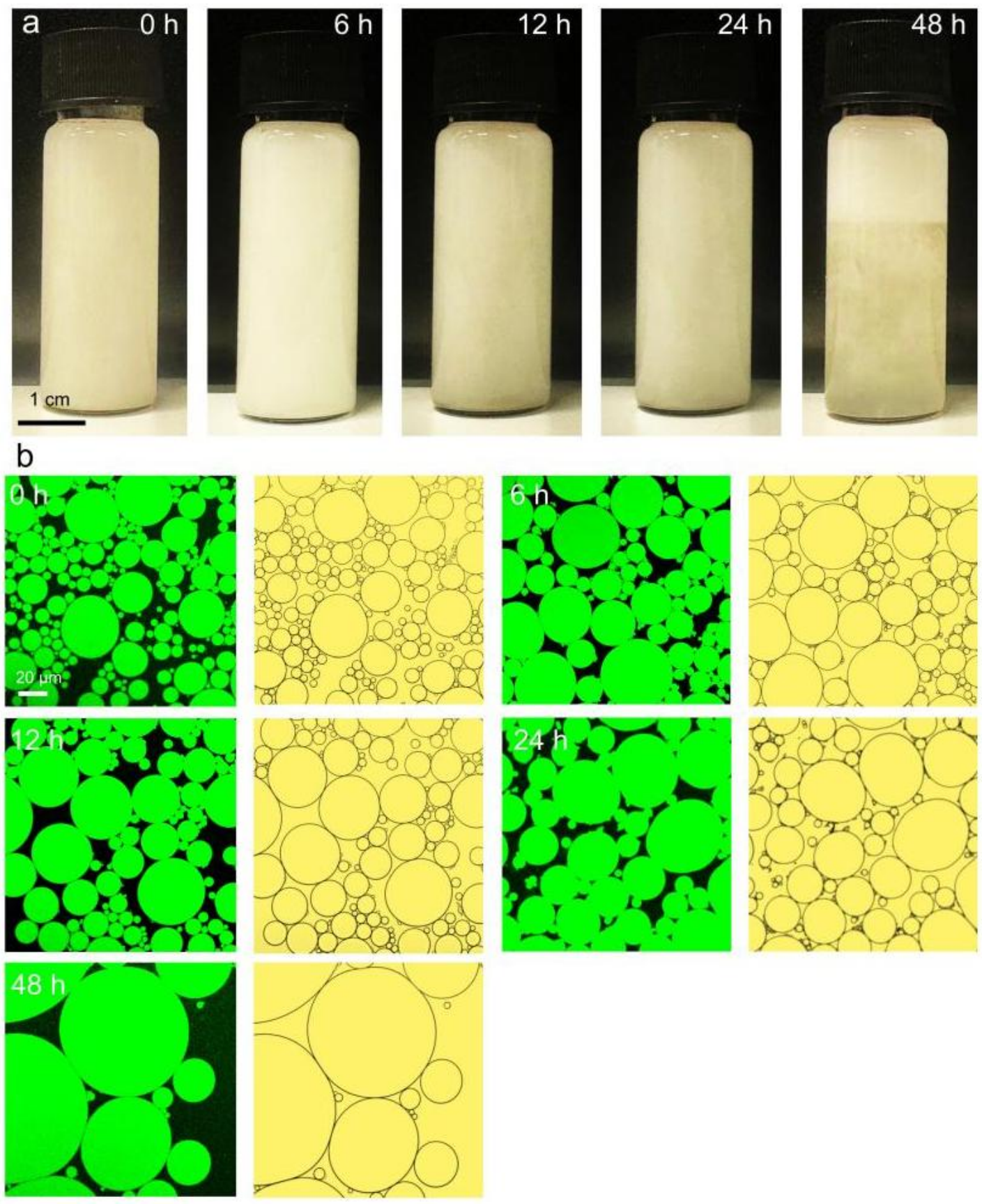

Supplementary Figure 22. (a) Macroscopic photos of the emulsion after standing for different periods of time. (b) The morphology of cell@OBC stabilized Pickering emulsions was recorded by CLSM at 0, 6, 12, 24 and 48 hours. At 48 hours, coalescence of droplets was observed.

Comment 6: The maximum interface covering is claimed at water-oil ratio 6:4. However, no explanation about this issue is included.

Reply: We have added a detailed explanation in the manuscript to rationalize why the 6:4 water-to-oil ratio provides the maximum interfacial area (and thus optimal biocatalytic performance) in our system. In brief, our reasoning is based on **classical emulsion theory** and the concept of **limited coalescence** in Pickering emulsions: For emulsions composed of approximately spherical droplets, the total interfacial area per unit emulsion volume (A_N) is determined by two key parameters: the dispersed-phase volume fraction (ϕ) and the droplet size, commonly represented by the Sauter mean diameter (d_{32}). According to classical emulsion theory, these quantities are related by:

$$A_N = \frac{6\phi}{d_{32}} \quad \text{(Eq. 2)}$$

This expression is well established and widely used to estimate the available interfacial area in emulsions (e.g., doi.org/10.3390/chemengineering2020016; McClements, Food Emulsions, Eq. 1.14). In Pickering emulsions, d_{32} is not solely controlled by the emulsification method, but is strongly influenced by particle-limited interfacial stabilization. As described by the concept of limited coalescence, droplets coalesce until sufficient particle coverage is achieved; therefore, the final droplet size reflects a balance between newly generated interfacial area and the available stabilizing particles. Based on this framework, the maximum interfacial area observed at a water:oil ratio of 6:4 can be rationalized as follows: At 6:4, the system reaches an optimal balance where a sufficiently high ϕ is combined with the smallest mean droplet diameter, thereby maximizing the ratio ϕ/d_{32} and, consequently, the total interfacial area. This interpretation is fully consistent with our experimental data, where the 6:4 formulation exhibits the smallest d_{32}, the highest droplet number density, and the largest calculated interfacial area.

Comment 7: It is not clear the ability for scaling in the studied system, some additional discussion and evidences should be included.

Reply: Thank you for your valuable suggestion. We agree that demonstrating scalability is important for highlighting the practical potential of our platform. In response, we have added new **experiments at significantly larger scale** and expanded the discussion of scalability. In the revised manuscript, we report a **5-liter scale-up** of our living Pickering emulsion system, accompanied by a preparative-scale biotransformation, to show that the approach remains effective when scaled by several orders of magnitude.

Key results and additions regarding scalability are as follows:

- **5 L Emulsion Formation:** We prepared a **5 L batch** of the Pickering emulsion (compared to 1 mL in typical lab tests, this is a 5000× scale increase). This was achieved simply by proportionally enlarging the components while using the same hand-shaking method. We were able to form a stable emulsion at this liter scale without issues. A photograph of the entire 5 L emulsion is now included in **Fig. 6i** of the revised manuscript, illustrating the macroscopic appearance of the emulsion in a large container.

- **Emulsion Uniformity Test:** To ensure that the emulsion was uniformly stabilized throughout such a large volume, we drew samples from different regions of the 5 L reaction vessel (specifically from the top, bottom, left, and right positions) after the emulsion had been sitting for a while. Each 1 mL sample was observed for any signs of local creaming or breakage (**Supplementary Figure. 54b**). We found that all samples appeared identical – no detectable phase separation or creaming differences, indicating that the emulsion's stability was homogeneous throughout the bulk. This suggests that our hand-shaking approach, even at 5 L scale, produces a consistent emulsion (though we note it required more effort/time to shake such a large volume, which is expected).

- **Gram-Scale Product Synthesis:** We then carried out the benzaldehyde-to-benzoin reaction in the 5 L emulsion to test catalytic performance upon scale-up. The reaction proceeded efficiently in the large reaction system, and after completion, we isolated approximately 7.5 g of enantiopure *R*-benzoin (yielding the same enantiomeric excess as in small-scale runs). A photograph of the isolated product (a substantial quantity of solid benzoin) is shown in **Fig. 6j** of the revised manuscript. This result demonstrates that the **catalytic activity and selectivity are preserved at liter scale**, and that the product can be obtained in preparative amounts without any loss of performance.
- **Discussion of Results:** We have added a dedicated paragraph in the discussion to highlight these scalability findings. We emphasize that the interfacial area and benefits of the Pickering emulsion can be translated to much larger scales – the living-cell emulsion maintained its structural integrity (no collapse or instability at scale), catalytic efficiency, and stereochemical outcome even when moving from lab scale to pilot scale. This strongly supports the idea that our platform could be used in realistic manufacturing settings. By contrast, many novel catalytic systems are demonstrated only on the milliliter scale; our additional evidence fills this gap for our work.

By providing the new data, we address the your concern directly: the revised manuscript now contains explicit evidence of the system's scalability and a discussion of its implications. We believe this strengthens the manuscript by showcasing that our method is not only scientifically interesting but also practically viable at scale. Thank you for suggesting this addition.

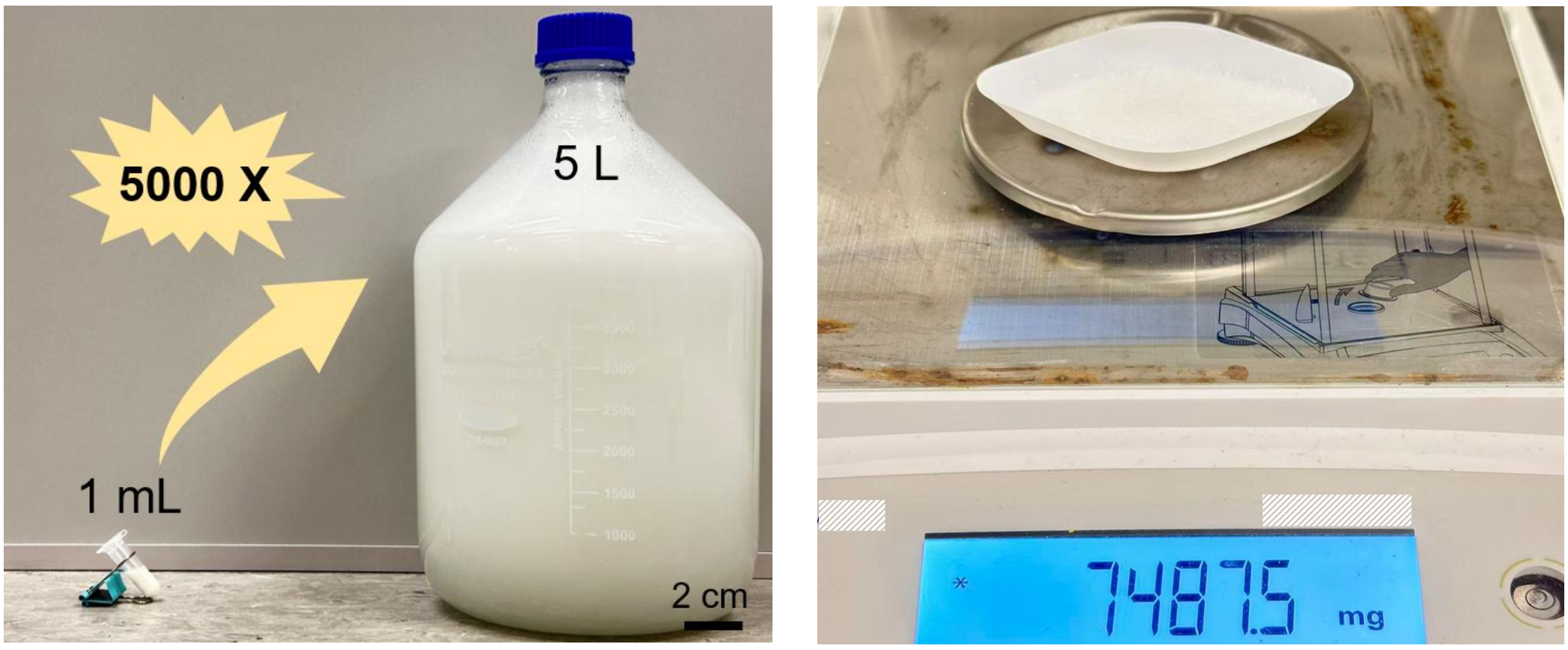

Figure 6. (i) 5 L of the enlarged emulsion was prepared and the catalysis was performed (left). (j) Approximately 7.5 g of purified benzoin was obtained (right).

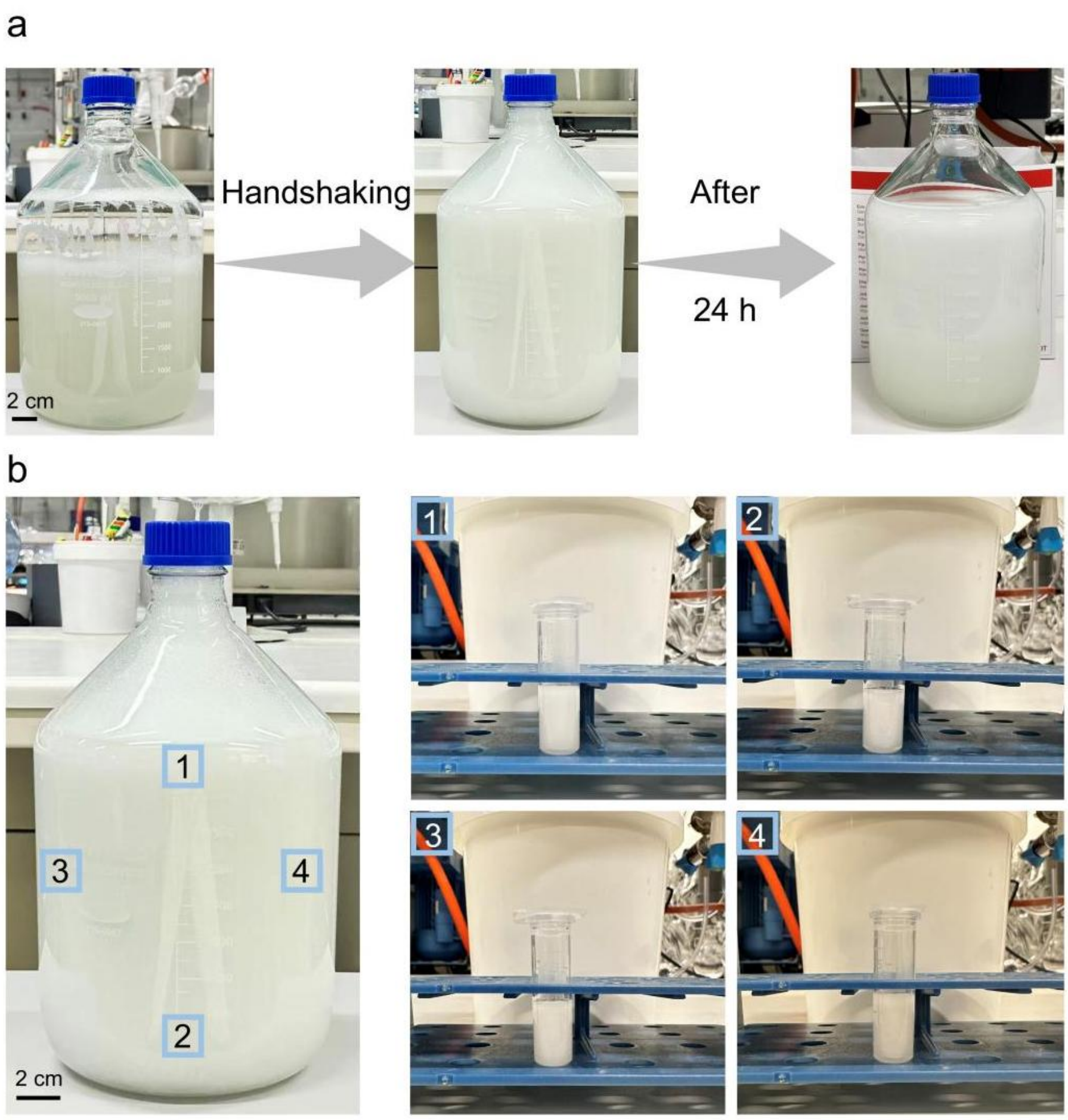

Supplementary Figure 54. (a) The 5 L scale-up experiment was further conducted, though it required a longer handshaking time. (b) Take 1 mL of emulsion from different positions within the 5 L emulsion system to observe the emulsion's spatial stability.

Point-by-point Reply

Reviewer #1:

The revised version, with well-justified novelty, clear presentation, thoughtful discussion, expanded supporting information, and new data, has fully addressed my concerns regarding the original manuscript. Based on my evaluation, the manuscript is now suitable for publication.

Reply: Thank you for recommending our manuscript for publication in *Nature Communications*. We greatly appreciate your constructive comments.

Reviewer #2:

The authors have modified the text and clarified my presenting points. It is OK.

Reply: Thank you for your constructive comments and valuable suggestions throughout the review process.

Reviewer #3:

After carefully evaluating the revised version submitted by the authors, I find that the manuscript is now suitable for publication. I appreciate the authors' diligent efforts in addressing the comments and substantially improving the quality of the work.

Reply: Thank you for recommending our manuscript for publication in *Nature Communications*. We greatly appreciate your constructive comments.
